# Synergistic Ground and Orbital Observations of Iron Oxides on Mt. Sharp and Vera Rubin Ridge

**DOI:** 10.1029/2019JE006294

**Published:** 2020-09-23

**Authors:** A. A. Fraeman, J. R. Johnson, R. E. Arvidson, M. S. Rice, D. F. Wellington, R. V. Morris, V. K. Fox, B. H. N. Horgan, S. R. Jacob, M. R. Salvatore, V. Z. Sun, P. Pinet, J. F. Bell, R. C. Wiens, A. R. Vasavada

**Affiliations:** ^1^ Jet Propulsion Laboratory California Institute of Technology Pasadena CA USA; ^2^ Johns Hopkins University Applied Physics Laboratory Laurel MD USA; ^3^ Department of Earth and Planetary Sciences Washington University St. Louis MO USA; ^4^ Geology Department, Physics and Astronomy Department Western Washington University Bellingham WA USA; ^5^ School of Earth and Space Exploration Arizona State University Tempe AZ USA; ^6^ NASA Johnson Space Center Houston TX USA; ^7^ Division of Geological and Planetary Sciences California Institute of Technology Pasadena CA USA; ^8^ Department of Earth, Atmospheric, and Planetary Sciences Purdue University West Lafayette IN USA; ^9^ Department of Astronomy and Planetary Science Northern Arizona University Flagstaff AZ USA; ^10^ Institut de Recherche en Astrophysique et Planétologie Université de Toulouse, CNRS, UPS, CNES Toulouse France; ^11^ Los Alamos National Laboratory Los Alamos NM USA

**Keywords:** Mars, spectroscopy, hematite, curiosity, CRISM<

## Abstract

Visible/short‐wave infrared spectral data from the Compact Reconnaissance Imaging Spectrometer for Mars (CRISM) show absorptions attributed to hematite at Vera Rubin ridge (VRR), a topographic feature on northwest Mt. Sharp. The goals of this study are to determine why absorptions caused by ferric iron are strongly visible from orbit at VRR and to improve interpretation of CRISM data throughout lower Mt. Sharp. These goals are achieved by analyzing coordinated CRISM and in situ spectral data along the Curiosity Mars rover's traverse. VRR bedrock within areas that have the deepest ferric absorptions in CRISM data also has the deepest ferric absorptions measured in situ. This suggests strong ferric absorptions are visible from orbit at VRR because of the unique spectral properties of VRR bedrock. Dust and mixing with basaltic sand additionally inhibit the ability to measure ferric absorptions in bedrock stratigraphically below VRR from orbit. There are two implications of these findings: (1) Ferric absorptions in CRISM data initially dismissed as noise could be real, and ferric phases are more widespread in lower Mt. Sharp than previously reported. (2) Patches with the deepest ferric absorptions in CRISM data are, like VRR, reflective of deeper absorptions in the bedrock. One model to explain this spectral variability is late‐stage diagenetic fluids that changed the grain size of ferric phases, deepening absorptions. Curiosity's experience highlights the strengths of using CRISM data for spectral absorptions and associated mineral detections and the caveats in using these data for geologic interpretations and strategic path planning tools.

## Key Points

- Areas on Vera Rubin ridge with deep ferric absorptions from orbit also have deep ferric absorptions in Curiosity spectral data sets
- Ferric phases are more widespread on Mt. Sharp than originally reported. Diagenesis deepened ferric absorptions in several locations
- Combining orbital and in situ observations enhances planetary exploration

## 1 Introduction

**Figure 1:**
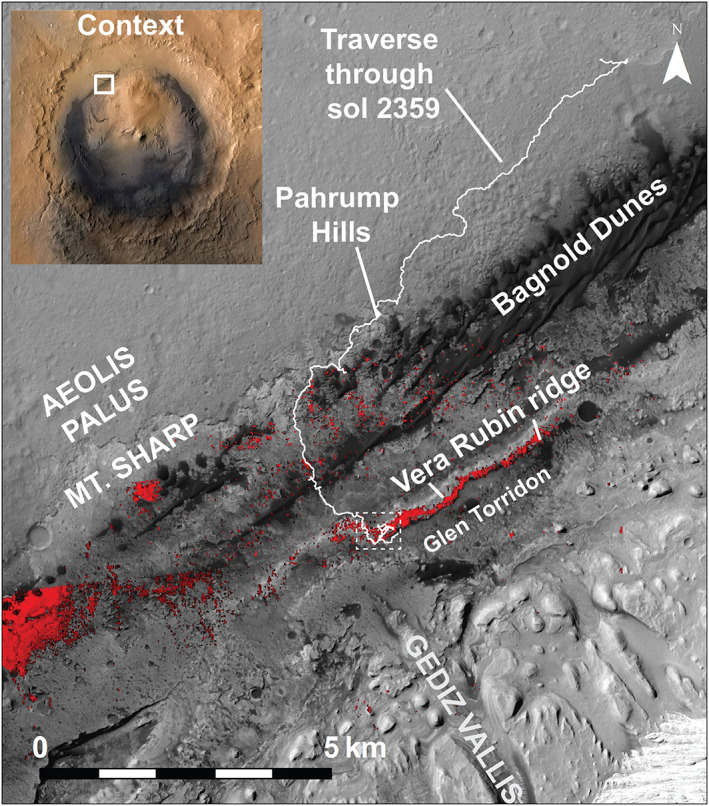
HiRISE mosaic showing the northwest quadrant of Mt. Sharp. The CRISM 860 nm band depth map from Fraeman et al. (2016) are also indicated in red. Curiosity's traverse through Sol 2,359 and key locations are labeled. Dotted box shows location of Figure 2. A context map in the upper left shows the location of this area with respect to the ~150 km diameter Gale Crater, which is located near 4.49°S, 137.42°E.

**Figure 2:**
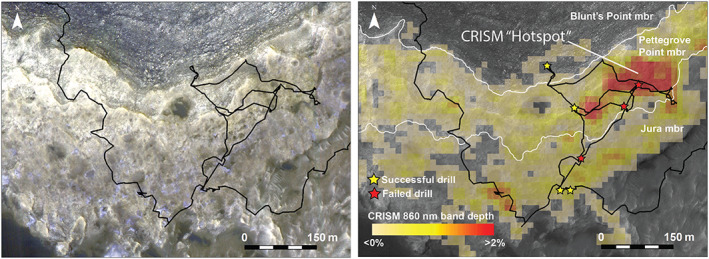
(left) Color stretched HiRISE mosaic from Fraeman et al. (2016) showing detail of Vera Rubin ridge. Specific HiRISE image from that mosaic shown here is ESP_021610_1755. (right) The 860 nm band depth map from CRISM scene ATO00021C92 that shows variability of 860 nm signature across VRR and drilled locations.

Vera Rubin ridge (VRR) is a topographic rise on the northwest flank of Mt. Sharp (formally named Aeolis Mons). In reflectance spectral data collected by the orbiting Compact Reconnaissance Imaging Spectrometer for Mars (CRISM), VRR is associated with an inflection centered at ~535 nm, absorption at 860 nm, and a local reflectance maximum at 750 nm, all of which are consistent with Fe^3+^ in red crystalline hematite (Figures 1 and 2) (Fraeman et al., 2013). Based on the putative CRISM hematite detection at VRR and lack of similar strong absorptions in strata immediately adjacent to the ridge, Fraeman et al. (2013) hypothesized that VRR could be a unique hematite‐rich interval that marked a site of localized iron oxidation and potential past habitable environment. As part of its ongoing mission in Gale Crater, the Mars Science Laboratory Curiosity was sent to explore the area and document its geologic history.

Curiosity's Chemistry and Mineralogy (CheMin) X‐ray diffraction (XRD) instrument detected crystalline hematite in VRR bedrock (Rampe et al., 2020), validating the interpretation from CRISM data that VRR contained hematite. However, the CRISM‐based interpretation that VRR was a unique hematite‐bearing interval in Mt. Sharp was not correct. Samples of bedrock from lower on Mt. Sharp analyzed by CheMin also contain hematite (Achilles, 2018; Bristow et al., 2018; Morrison et al., 2018; Rampe et al., 2017), but these areas are not associated with strong hematite‐absorptions in CRISM data.

This study addresses three major questions: (1) Why is VRR associated with deeper ferric spectral absorptions from orbit relative to underlying areas? (2) What does the answer to (1) imply about orbital evidence for the presence/absence of hematite throughout lower Mt. Sharp? (3) What do answers to these questions tell us about the geologic history of Mt. Sharp? We answer these questions by comparing the high‐spatial resolution spectral data Curiosity collected from Pahrump Hills at the base of Mt. Sharp through VRR (Sols 758–2,300) with corresponding CRISM observations to understand how surface characteristics influence how hematite manifests in orbital spectral data over lower Mt. Sharp. In addition to answering these questions, we also include a discussion about lessons learned for the strengths and caveats in applying CRISM data for geologic interpretations. CRISM data will continue to assist Curiosity as the rover climbs Mt. Sharp and traverses strata associated with orbital spectral signatures that are consistent with phyllosilicates and sulfates, which may reflect changing Martian environments (Milliken et al., 2010). Future mobile missions, such as NASA's Mars 2020 rover and ESA's ExoMars rover, will also rely on CRISM data to find and access key outcrops. We therefore also provide recommendations for using CRISM data in strategic path planning for Curiosity and future rovers.

## 2 Background

### 2.1 Geologic Setting of Mt. Sharp and Vera Rubin Ridge

Mt. Sharp is a ~5 km high mound of stratified material in the center of the ~150 km diameter Gale Crater. Curiosity is currently climbing the mound to document evidence of the changing, potentially habitable environments that are preserved in its strata. To date, the MSL science team has defined two group‐level stratigraphic units within Mt. Sharp using a combination of orbital mapping and fine‐scale sedimentologic indicators documented by Curiosity's instruments. These units are the Mt. Sharp group and the Siccar Point group (Banham et al., 2018; Fraeman et al., 2016; Grotzinger et al., 2015). The team also defined a third stratigraphic group on the floor of Gale Crater named the Bradbury group (Grotzinger et al., 2015). The Bradbury and Mt. Sharp groups are a package of fluvio‐deltaic and lacustrine deposits, and the Siccar Point group unconformably overlies the Mt. Sharp group (Banham et al., 2018; Grotzinger et al., 2015). The Murray formation is the only formation‐level subdivision thus far within the Mt. Sharp group, and it is further divided into five lithostratigraphic members below VRR (Edgar et al., 2020). In order of increasing elevation, the members are the Pahrump Hills member, Hartmann's Valley member, Karasburg member, Sutton Island member, and Blunts Point member. The Pahrump Hills, Blunts Point, and parts of the Karasburg members are comprised of finely laminated mudstones, while Hartmann's Valley and parts of the Karasburg member contain mud to sand stones with decimeter‐ to meter‐scale cross bedding. The Sutton Island member is a heterolithic mudstone and sandstone.

Before Curiosity's in situ exploration, orbital data were used to create “orbitally defined units” in Mt. Sharp that were distinguished based on mineralogy inferred from CRISM data as well as textural and physical properties (e.g., thermal inertia) (Anderson & Bell, 2010; Fraeman et al., 2016; Le Deit et al., 2013; Milliken et al., 2010; Thomson et al., 2011). The Murray formation was initially mapped as a single orbitally defined unit starting at Pahrump Hills and ending at VRR (Grotzinger et al., 2015; Stack et al., 2019). It was demarcated by its distinctive expression in High Resolution Imaging Science Experiment (HiRISE) data and by its association with a medley of weak orbital spectral features matched to library data for phases that include sulfates, phyllosilicates, mafic sands, hydrated silica, and iron oxides (Fraeman et al., 2016; Milliken et al., 2010; Thomson et al., 2011).

VRR was a separate orbitally defined unit in large part because of its association with strong features at 535 and 860 nm in CRISM data, in addition to its distinctive texture and topography (Anderson & Bell, 2010; Fraeman et al., 2013) (Figures 1 and 2). No other spectral absorptions are present at longer wavelengths in CRISM data over the ridge. VRR has a positive topographic expression with respect to underlying strata and is composed of light toned, well consolidated bedrock (Figure 2). The ridge is ∼200 m wide and extends ∼6.5 km northeast‐southwest, approximately parallel with the base of Mt. Sharp. VRR is also located near the mouth of the Gediz Vallis channel, a ~10 km long channel that is one of the youngest geomorphic features on Mt. Sharp (Figure 1).

Despite being mapped as a separate orbitally defined unit, stratigraphic analyses using Mastcam and MAHLI images acquired from Curiosity demonstrate that VRR strata are still part of the Murray formation (Edgar et al., 2020). The rocks within VRR are primarily thinly laminated mudstones and are interpreted to have been deposited in a fluvial‐lacustrine setting, consistent with the underlying strata (Edgar et al., 2020). VRR is composed of two stratigraphic members, the Pettegrove Point member and the Jura member (Edgar et al., 2020). The Pettegrove Point member is characterized by fine‐grained, thinly laminated, parallel stratified bedrock (Edgar et al., 2020). The Jura member is characterized by fine‐grained, thinly laminated mudstone to fine sandstone, and it is distinguished from the Pettegrove Point member because it occasionally has decimeter to meter‐scale inclined strata that dip in multiple directions (Edgar et al., 2020). The two stratigraphic members within VRR are distinguishable from orbit (Figure 2). Qualitatively, the Jura member is darker and more heavily cratered than the Pettegrove Point member, and stratification in the Jura member is not readily apparent (Edgar et al., 2020).

### 2.2 Identifying Hematite on Mars With Reflectance Spectroscopy

Spectral data, including data provided by CRISM, can be used to remotely discriminate spectral absorptions consistent with crystalline red hematite and other crystalline high‐spin ferric oxides/oxyhydroxides (e.g., Murchie et al., 2009 and references therein) using four electronic absorptions between ~400 and 1,000 nm (e.g., Y. He et al., 2005; Morris et al., 1985; Sherman, 1985; Sherman et al., 1982; Sherman & Waite, 1985). Three are single‐electron transitions (cubic symmetry notation): (1) (^6^A_1_ ➔ ^4^A_1_,^4^E_1_) between ~400 and ~415 nm, (2) ^6^A_1_➔^4^T_2_ between ~650 and 710 nm, and (3) ^6^A_1_ ➔ ^4^T_1_ between ~840 and ~910 nm. The fourth is a pair transition 2(^6^A_1_)➔2(^4^T_1_) between ~485 and ~550 nm. The transitions are spin and Lapotre allowed because of magnetic coupling between adjacent Fe^3+^ cations (e.g., Y. He et al., 2005; Rossman et al., 1996; Sherman, 1985; Sherman & Waite, 1985). Furthermore, a manifestation of the face‐sharing arrangement of FeO_6_ polyhedra in hematite as opposed to other ferric oxides/oxyhydroxides is a strongly enhanced absorption near 535 nm resulting from intensification of its ^6^A_1_ ➔ ^4^A_1_,^4^E_1_ and pair (2(^6^A_1_) ➔ 2(^4^T_1_)) transitions (Sherman & Waite, 1985).

**Figure 3:**
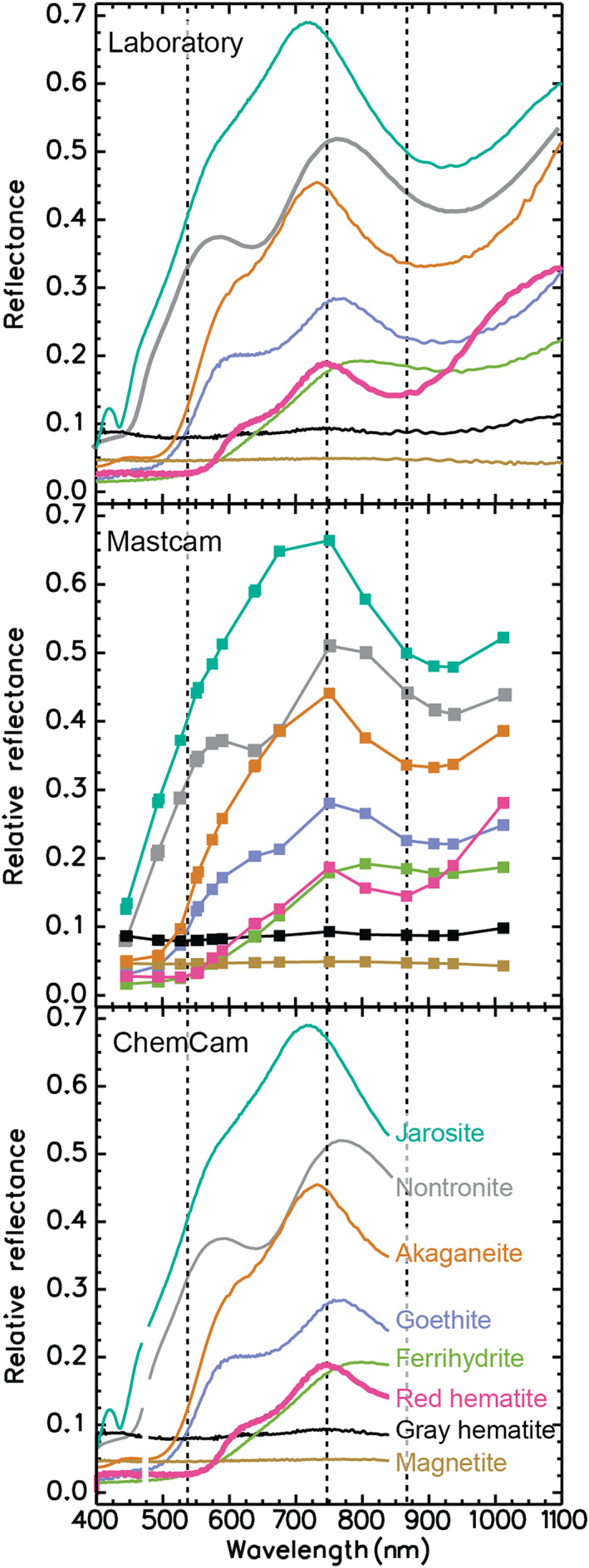
(top) Laboratory spectra of ferric materials convolved to Mastcam (middle) and ChemCam passive spectral (bottom) wavelengths. Hematite can be uniquely identified in Mastcam spectra based on its absorption near 860 nm and also has an absorption near 530 nm and a peak at 750 nm. Laboratory spectra sources: USGS spectral library (all Kokaly et al., 2017)—Goethite WS222 Medium Gr. [W1R1Ba], ematite WS161 [W1R1Bb], hematite WS161 [W1R1Bb], ferrihydrite GDS75 Syn; F6 [W1R1Bb], magnetite HS78.3B [W1R1Bb], and Jarosite JR2501 (K) [W1R1Bb AREF]. PDS spectral library (https://speclib.rsl.wustl.edu/)—Akaganeite [AKS1_FP_0_C4S47398_0101] (Morris et al., 2000) and Nontronite [NBJB26] (Pieters, 1983).

Ferric (oxyhydr)oxides are observable in reflectance spectra based on these absorptions when present in pigmentary form (Figure 3). “Pigmentary form” is defined as having color at visible wavelengths (a positive‐sloped absorption edge in the red between ~530 and 760 nm; Figure 3), which implies the phase is crystalline and has a small particle diameter. Identifying which specific ferric oxide/oxyhydroxide is present can be problematic because the position of the absorptions described above from these phases are nonunique strongly overlap and have large linewidths (e.g., Morris et al., 2000; Scheinost et al., 1998). Hematite is an exception because the 535 nm spectral inflection is enhanced relative to other ferric (oxyhydr)oxides. The position of the ^6^A_1_ ➔ ^4^T_1_ transition also occurs at the shortest wavelength (~860 nm) for ferric (oxyhydr)oxides (e.g., Morris et al., 2000; Scheinost et al., 1998; Sherman et al., 1982), so that detection of both ~535 and ~860 nm spectral features is spectral evidence that (red) pigmentary hematite is present.

The preceding paragraphs pertain to spectral observations of hematite‐bearing geologic materials where the oxide is distributed throughout (at least in part) as small particles of pigmentary (i.e., red) hematite. For sufficiently large hematite particles (specular hematite), the ferric absorption edge is weak or not present (Lane et al., 2002; Morris et al., 2020), meaning it is approximately spectrally neutral over visible wavelengths, imparting a black to gray color when mixed with other spectrally neutral but higher albedo materials. When present, specular hematite will reduce the contrast of spectral features from other phases compared to how they would appear if the oxide were absent as shown, for example, by Singer (1981) for mixtures of olivine and magnetite and Clark and Lucey (1984) for mixtures of ice and charcoal where magnetite and charcoal are spectral surrogates for specular hematite. The particle‐diameter transition from pigmentary to specular behavior is empirically defined at ~3–5 μm (Catling & Moore, 2003; Lane et al., 2002; Morris et al., 2020).

Over the region 400 to 1,000 nm, global Martian surface dust is characterized by a ferric absorption edge between ~400 and ~750 nm, as well as a broad, shallow band centered near 850 nm consistent with contributions from multiple ferric phases (including ferric oxides/oxyhydroxides; Figure 3) and even possibly ferrous silicate phases (Bell et al., 1990, 2000; Morris et al., 2000; Mustard & Bell, 1994). The spectral properties of the global dust are distinct from pigmentary hematite described above. They are instead approximated by palagonitic tephra from Maunakea volcano where nanoscale ferric particles are the pigment (Morris et al., 1993, 2000) and by physical mixtures of X‐ray amorphous and crystalline hematite particles embedded in a glassy SiO_2_ matrix (Morris et al., 1989). Although reflectance spectra of hematite by itself distributed as nanophase (XRD amorphous) and crystalline particles in a spectrally neutral matrix can mimic the spectral properties of Martian dust, there is not sufficient information to exclude or include other ferric (oxyhydr)oxides as contributory pigments. Because of the spectral ambiguity, Morris et al. (2000) used the generic name “nanophase ferric oxide” for the dust ferric pigment.

CRISM data show several large, isolated areas in lower Mt. Sharp that are associated with spectra that have diagnostic ferric features at 535 and 860 nm (Figure 1), as a well a local reflectance maximum near 750 nm (Fraeman et al., 2016; Milliken et al., 2010; Seelos et al., 2019). These spectra are consistent with hematite for reasons presented above (Fraeman et al., 2013). Similar absorptions are not present in CRISM data over Glen Torridon, the trough immediately south of VRR (Figure 1). There are a few pixels immediately to the north of VRR that are associated with CRISM spectra which also have weak 860 and 535 nm features, but these absorptions are often on the same order of magnitude as instrument noise and the individual pixels are not clearly associated with specific geologic features. The contrast between the strong absorptions interpreted to indicate hematite that are associated with VRR versus the scattered, weak ferric absorptions in the strata to the north and south led to the interpretation that VRR was an isolated hematite‐rich interval within the Mt. Sharp sedimentary sequence. Fraeman et al. (2013) hypothesized the VRR preserved a redox interface based on this geologic inference.

## 3 Data Sets and Methods

**Figure 4:**
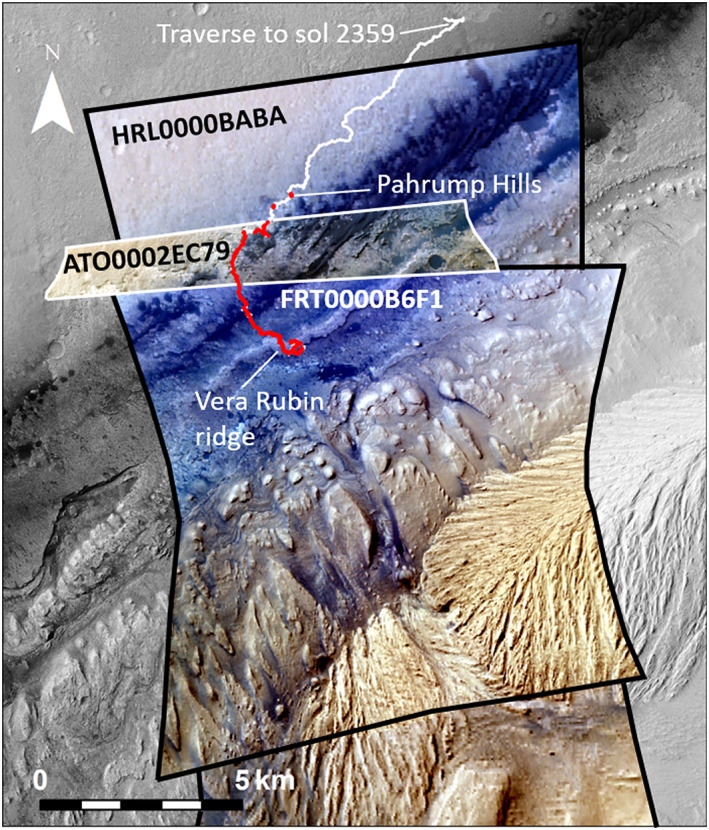
Location of CRISM scenes and section of Curiosity traverse (red) analyzed in this paper.

Curiosity has two instruments that are capable of collecting spectral information: Mast Camera (Mastcam) and Chemistry and Camera (ChemCam) (Bell et al., 2017; Malin et al., 2017;Maurice et al., 2012 ; Wiens et al., 2012). We examined spectral data from Mastcam and ChemCam collected between Sols 758 and 2,360 of the mission (September 2014 to March 2019), which correspond to the traverse from the base of Mt. Sharp in the Pahrump Hills through VRR and a small portion of Glen Torridon. We also examined coordinated CRISM scenes over the same area (Figure 4). The specifics of these instruments and data products used are described below.

### 3.1 Orbital Data Sets

CRISM measures reflected radiance from the Martian surface and atmosphere at 544 discrete visible short‐wave infrared (VSWIR) wavelengths between 362 and 3,920 nm (Murchie et al., 2007). CRISM is a line scanning (cross track) spectrometer that is mounted on a gimbal that slews in the along‐track direction. In full resolution targeted (FRT) mode, CRISM has a spatial resolution of 18 m/pixel. CRISM also can collect half resolution long (HRL) scenes that cover greater areas than FRTs but provide half the spatial resolution (36 m/pixel). A third observing mode, along‐track oversampled (ATO), is achieved by adjusting the angular velocity of CRISM's gimbal to collect 18 m/pixels at intervals <18 m in the along‐track direction. ATO data can be processed to an along‐track resolution of 9–12 m/pixel using an iterative maximum‐log likelihood (MLM) algorithm, which retrieves the best estimate of scene radiance in the presence of noise (L. He et al., 2019; Kreisch et al., 2017). The MLM processing technique can also be applied in the spectral dimension for HRL, FRT, and ATO images to de‐noise spectra (He et al., 2019).

We used MLM‐processed CRISM ATO (used to enhance both the spectral and spatial dimension), FRT, and HRL (spectral only) scenes that had also been atmospherically corrected using the Discrete Ordinates Radiative Transfer Program for a Multi‐Layered Plane‐Parallel Medium (DISORT) (L. He et al., 2019; Kreisch et al., 2017). Using DISORT, we modeled the joint radiance contributions from the Martian surface and atmosphere, including the scene‐dependent effects of aerosols. Integrating the photometric properties of the Martian surface allowed us to model surface radiance as single scattering albedo (SSA), a measure of reflectance that describes the ratio of scattering to scattering absorption efficiencies for a single event and that is independent of viewing geometry (Arvidson et al., 2006; Johnson et al., 2006). Hematite within each CRISM scene was mapped using the 860 nm band depth parameter optimized for CRISM and defined in Viviano‐Beck et al. (2014). Spectra from pixels with high 860 nm band depths were then manually inspected to ensure that they had all three diagnostic hematite spectral features detectable by CRISM (features at ~535 and 860 nm, local maximum at ~750 nm).

The three main CRISM scenes used in this work are HRL0000BABA, FRT0000B6F1, and ATO0002EC79 (Figure 4). Some portions of the traverse through Bradbury group units are not covered by these scenes, but those areas were not part of the analyses here. The image HRL0000BABA has the lowest spatial resolution but provides full coverage of Curiosity's entire traverse through the Mt. Sharp and Siccar Point group units within a single scene. FRT0000B6F1 has higher spatial resolution but only covers half of the rover's traverse. HRL0000BABA and FRT000B6F1 were acquired within 1 week of each other, under very similar and clear atmospheric conditions. An initial estimate for atmospheric opacity due to dust, tau_dust, was derived using techniques described in Wolff et al. (2009; 2019) and then iterated with that starting value until models converged on single tau_dust value that resulted in similar SSA spectra from the two frames where they overlapped. For both scenes, tau_dust was estimated to be 0.33 at 900 nm. ATO0002EC79 is the highest resolution scene but has the lowest spatial coverage. This scene was acquired several years later than the other two CRISM scenes, in similarly clear atmospheric conditions. Here, tau_dust was again initially estimated using the techniques of Wolff et al. (2009; 2019), and tau_dust estimates were iterated until model results produced SSA values that were similar for overlapping regions for HRL0000BABA. For this scene, tau_dust was estimated to be 0.4 at 900 nm. All CRISM data were registered to a HiRISE base map using hand selected ground control points focused near Curiosity's traverse, and the data were warped using the first‐order polynomial function available in the georeferencing toolkit that is included in ArcMap software by Esri (www.esri.com). Small offsets in registration remain and are a source of error in the Curiosity/CRISM comparisons presented later.

### 3.2 Curiosity Spectral Instruments

Mastcam is a pair of science cameras on Curiosity's remote sensing mast (Bell et al., 2017; Malin et al., 2017). The left Mastcam (M34) has an instantaneous field of view (IFOV) of 0.22 mrad, providing a sampling of 450 μm/pixel for targets 2 m away. The right Mastcam (M100) has an IFOV of 0.074 mrad, equivalent to a three times better spatial sampling of 150 μm/pixel for targets 2 meters away. Each Mastcam is equipped with a filter wheel that has a set of seven filters, plus an eighth broadband infrared cutoff filter used in combination with the RGB Bayer pattern bonded directly to the detectors. Combined, the single wavelength camera filters and RGB patterns provided by the Bayer filters can resolve 12 unique bands from 445 to 1,013 nm, which can be used to distinguish many iron‐bearing minerals (Figure 3) (Bell et al., 2003; Malin et al., 2017). Further details about the uses of narrow‐band filters and Bayer filters can be found in Wellington et al. (2017).

We calibrated Mastcam observations to radiance (W/m^2^/nm/sr) using preflight calibration coefficients and to I/F values (scene radiance/(π*solar irradiance)) using in situ observation of the Mastcam radiometric calibration target that is mounted to the rover's deck. We calculated relative reflectance values by dividing I/F by the cosine of the solar incidence angle (Wellington et al., 2017). The full details of the calibration process are available in Wellington et al. (2017) and Bell et al. (2017). We used Mastcam multispectral landscape images to map materials that had spectral properties consistent with hematite using the 527 and 867 nm band depth parameters developed in Wellington et al. (2017) and defined in Table 1. We also examined decorrelation stretch (DCS) images created using filters centered near the 867 nm feature (R3 [805 ± 10 nm], R4 [908 ± 11 nm], and R6 [1,031 ± 21 nm] or L3 [751 ± 10 nm], L5 [867 ± 10 nm], and L6 [1,012 ± 21 nm]) to highlight variability of ferric absorption features within a scene.

**Table 1:** Equations Used for Spectral Parameters in This Paper

Instrument	Parameter^a^	Equation^b^	Reference
Mastcam	BD527	1‐R_527_/[0.648R_446_ + 0.352R_676_]	Wellington et al. (2017)
Mastcam	BD867	1‐R_867_/[0.556R_751_ + 0.444R_1012_]	After Bell et al. (2000)
ChemCam/CRISM	BD535	1‐R*_535_/[0.65 R*_500_ + 0.35 R*_600_]	Johnson et al. (2015)
ChemCam/CRISM	S750:840	(R*_750_ − R*_840_)/(750 − 840)	Johnson et al. (2015)
CRISM	BD860_2	1‐R*_860_/[0.527 R*_755_ + 0.473 R*_977_]	Viviano‐Beck et al. (2014)

^a^ BD### = band depth centered at ### nm; S840:750 = slope between 840 and 750 nm.

^b^ R_###_ = reflectance at ###; R*_###_ = average of five wavelengths centered at ### nm.

In addition to landscape images, we calculated the 527 and 867 nm band depth of spectra along the traverse from Mastcam multispectral observations of rock targets that we have identified as belonging to the Murray formation (sandstones belonging to the Stimson formation and anomalous float rocks were excluded) (Table S1 in the supporting information). We also excluded photometry sequences and other observations acquired with large incidence angles (acquired more than 90 min before or after local noon) or large emission angles (e.g., observations of distant targets) because the Lambertian scatting assumptions in the Mastcam I/F calibration break down at large phase angles (Bell et al., 2017). We extracted spectra from Mastcam images by selecting common regions of interest (ROIs) within the left and right Mastcams, following the methodology described by Starr et al. (2019) and Rice et al. (2019). ROI averages from each camera were scaled to their average value at 1,012 nm (L6 and R6 filters) with the other overlapping filter positions averaged. Errors in Mastcam spectra were taken as the variance of the pixels averaged in each ROI, rather than instrumental noise (which is typically much smaller; Bell et al., 2017).

ChemCam is a laser‐induced breakdown spectrometer (LIBS) that nominally operates by measuring elemental emission lines in laser‐generated plasma (Maurice et al., 2012; Wiens et al., 2012). The violet (382–469 nm) and visible/near‐infrared (474–906 nm) spectrometers on ChemCam have sufficient sensitivity to collect reflectance spectra from surfaces without the use of the laser (passive mode) (Johnson et al., 2015, 2016). After every LIBS point, ChemCam acquired a 3 msec exposure passive measurement that was converted to target radiance and relative reflectance using methods developed and described in Johnson et al. (2015). Resulting ChemCam passive spectra have useable data from 400 to 840 nm with spectral sampling 0.04–0.2 nm depending on wavelength and spatial resolutions that range from 1.3 to 4.5 mm for typical targets distances of 2–5 m, respectively (Johnson et al., 2015). The wavelength range and sampling can detect absorptions caused by iron in minerals (Johnson et al., 2016). Notably, however, absorptions consistent with hematite are challenging to uniquely distinguish from absorptions caused by other iron oxides because the diagnostic location of the minimum of the 860 nm hematite absorption is uncertain because spectral coverage is truncated at the longest wavelength (Figure 3). We calculated the 535 nm band depth and slope of reflectance values between 750 and 840 nm using equations developed in Johnson et al. (2015) and defined in Table 1.

Passive ChemCam observations are made after the LIBS observations to take advantage of the dust removal provided by the shock wave from the plasma. The laser pulses used for LIBS are known to cause phase transitions, especially for metal oxides (e.g., Fau et al., 2019). However, the focused laser beam diameter (~0.4 mm at 2.5 m; Maurice et al., 2012) is much smaller than the field of view of the spectrometers (0.74 mrad). The ratio of areas of the spectrometer field of view to the laser beam footprint is ~20 at 2.5 m, an approximate mean distance for all ChemCam observations, so the area of altered material in the laser crater is very minor (~5%) compared to the total area observed by ChemCam. The credibility of making passive reflectance observations with ChemCam was established by Johnson et al. (2015).

**Figure 5:**
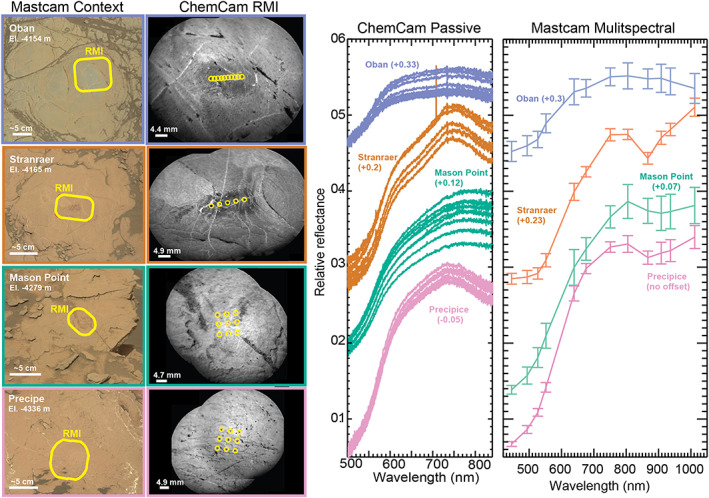
(left) Context Mastcam and associated RMI images for ChemCam observations showing typical bedrock targets along Curiosity's traverse through Mt. Sharp. (right) ChemCam passive spectra and Mastcam spectra associated with DRTed targets demonstrating the spectral variability of the Murray formation. Both Stranraer and Oban were located on VRR and represent end‐members in the area; Stranraer had some of the deepest ferric absorptions along the traverse while Oban was a member of the “gray” class of rocks that contains coarse grained gray hematite. Observation IDs are Oban—Mastcam: *Sol 1,905, product identifiers 0099940(08–21)*, RMI: *CRO_566517863PRC_F0671358CCAM01904L1, CRO_566518772PRC_F0671358CCAM01904L1*; Stranraer—Mastcam: *Sol 2,007, product identifiers 01056000(1–7)*, RMI: *CRO_575483031PRC_F0690408CCAM01005L1*, *CRO_575483650PRC_F0690408CCAM01005L1*; Mason point—Mastcam: *Sol 1,696, product identifiers 0088450(01–14)*, RMI: *CRO_547960268PRC_F0630346CCAM01695L1, CRO_547961029PRC_ F0630346CCAM01695l*; Precipice—Mastcam: *Sol 1,532, product identifiers 00780900(1–7),* RMI: *CRO_533402746PRC_F0592830CCAM01531L1, CRO_533404002PRC_ F0592830CCAM01531.*

All ChemCam targets are associated with a corresponding image from ChemCam's Remote Micro‐Imager (RMI) that provides textural context for the point spectra (Figure 5) (Le Mouélic et al., 2015; Maurice et al., 2016; Wiens et al., 2012). For this work, we used RMIs to select only ChemCam passive spectra from sunlit bedrock surfaces within the Mt. Sharp group; we excluded shadowed spectra or spectra that covered sands, soils, veins, other diagenetic features, drill tailings, or float rock (Table S2). We also excluded ChemCam passive data from Sols 2,076–2,215 because their calibration was uncertain due the large amount of scattered light from during the 2018 planetary encircling dust event.

The ChemCam and Mastcam data sets are complementary to one another. As a multispectral imager, Mastcam provides low spectral resolution information for every pixel across an entire image, which can be used to understand geologic relationships between materials with distinct spectral properties. ChemCam is a hyperspectral point spectrometer, meaning it collects higher spectral resolution data than the multispectral Mastcam, but spectra are restricted to <5 mm‐size‐targeted points on a single nearby target. ChemCam is also sensitive to a more limited spectral range than Mastcam (Figure 3). Mastcam data can be heavily affected by dust cover, whereas ChemCam spectra are less affected by dust contamination because the prespectra collection LIBS laser shots ablate much of the surface dust (Johnson et al., 2015, 2016). Therefore, for the most direct comparisons to ChemCam spectra, the full Mastcam spectra analyzed here are taken from targets that have been partially cleared by Curiosity's Dust Removal Tool (DRT).

Previous studies have validated the ability for these methods to detect ferric phases. Seven Mt. Sharp rocks below VRR that have crystalline ferric phases measured by Curiosity's CheMin XRD are also associated with ferric absorptions in ChemCam and Mastcam spectral data sets (Jacob et al., 2020; Johnson et al., 2016; Wellington et al., 2017). Conversely, ferric absorption features were consistently weak or absent in ChemCam data and in Mastcam spectral data in six samples from Mt. Sharp that do not have crystalline ferric phases in CheMin XRD data (Johnson et al., 2016; Wellington et al., 2017).

## 4 Results

### 4.1 Curiosity Maps of Ferric Absorptions in Murray Formation Bedrock

**Figure 6:**
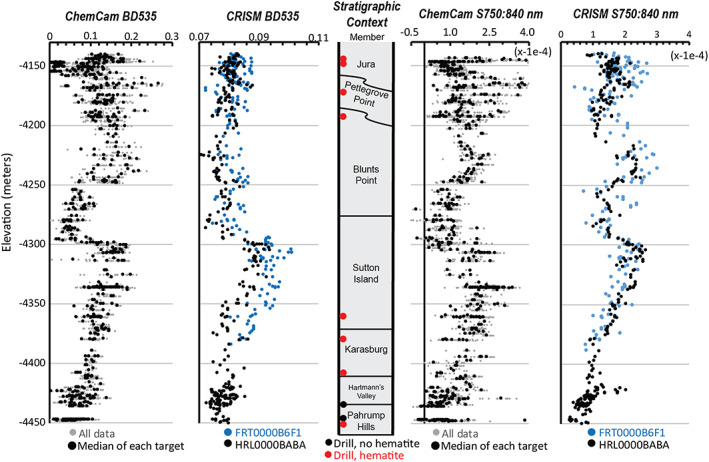
(left) Variability of 535 nm band depth versus elevation seen in ChemCam passive bedrock observations compared with CRISM data from two scenes along Curiosity's traverse. (center) Stratigraphic members versus elevation for context. (right) Variability of 750:840 nm slope from ChemCam passive and CRISM spectra. CRISM and ChemCam parameters were calculated using the same formulas.

We calculated the spectral parameters that describe absorption features near 535 and 860 nm (defined in Table 1) for all ChemCam passive spectral targets acquired on sunlit bedrock surfaces and Mastcam DRT surfaces in the Murray formation. Almost all ChemCam spectra from Pahrump Hills through the top of the Blunts Point member have a 535 nm absorption and 840 nm downturn, indicating they contain crystalline ferric components (Figure 6). A notable exception is spectra collected in the elevation interval near the Buckskin drilled rock sample, around ~ −4,430 m, which was enriched in silica (Hurowitz et al., 2017; Rampe et al., 2017). Although ferric absorptions are almost always present, the depth of the 535 nm band and the magnitude of the 840 to 750 nm slope varied along the traverse. Both parameters increased through the first ~150 m of elevation (Pahrump Hills member through middle of Sutton Island member), dropped near elevation −4,300 m (top of the Sutton Island member) and then increased for the remaining ~100 m climb toward VRR (top of Sutton Island and Blunts Point member).

**Figure 7:**
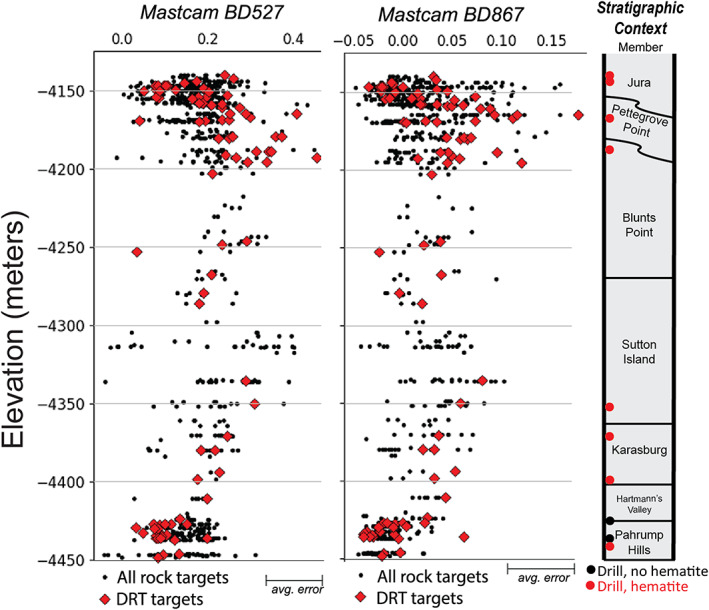
(left) Variability of 527 nm band depth versus elevation and (center) variability of 867 nm band depth versus elevation in Mastcam bedrock observations. (right) Stratigraphic context.

Almost all Mastcam spectra of DRT targets from Pahrump Hills through the top of the Blunts Point member are similarly consistent with ferric‐bearing materials. The patterns of varying depths of the 527 and 867 nm absorptions are also consistent with trends in similar ChemCam spectral parameters (Figure 7). Specifically, the 527 and 867 nm band depths increase from the Pahrump Hills member through the base of the Sutton Island member, although the continuation of this trend at higher elevations within the Sutton Island member is difficult to follow given a lack of DRT target observations between −4,250 and −4,300 m elevation.

Within VRR, the Pettegrove Point and Jura members have many rock targets that are associated with 535 nm absorptions and 840 nm downturns in ChemCam data as well as 527 and 860 nm bands in Mastcam data, although some targets are not associated with these spectral features. Overall, VRR rocks exhibit a wider range of spectral parameters values than targets from all of the underlying strata in both ChemCam and Mastcam data (Figures 6 and 7). While generally the depth of the 535 nm band and magnitude of the 840 to 750 nm slope from these targets are within the same range with the previous values, select targets do have slightly deeper bands than anywhere else along the traverse. Similarly, the 527 and 867 nm band depths in Mastcam spectra are generally within the range of previous values, with some targets exhibiting stronger absorptions. The bedrock targets that do not have ~530 and ~860 nm absorptions also have a less steep ~400–600 nm slope, making them appear gray in color compared to the typical red or purple rocks that contain stronger ferric spectral bands (Figure 5). Gray targets with weaker ferric spectral absorptions were more commonly observed in the Jura member, although a few are also observed in the Pettegrove Point member.

**Figure 8:**
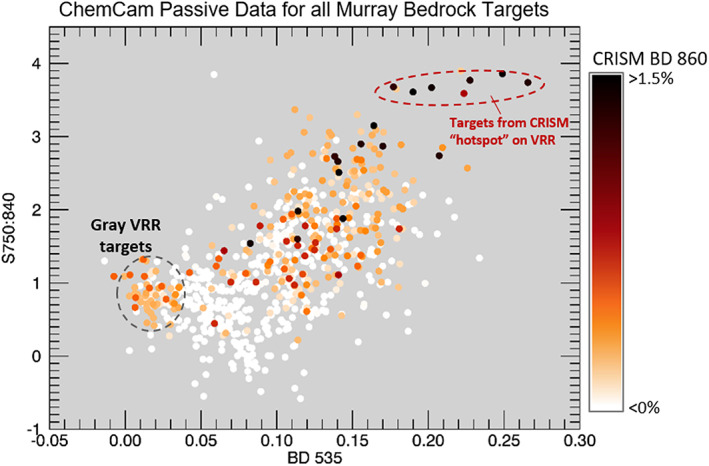
Median 535 nm band depth versus median 750:840 nm ratio for each ChemCam target from all bedrock observations in the Murray formation collected between Sols 758 and 2,300. Points are colored based on the 860 nm band depth of the CRISM pixel over location where target was located. The targets with the combined deepest 535 nm band depth and 750:840 nm ratio from ChemCam passive spectra correspond to regions on VRR with the deepest CRISM 860 nm bands. Targets with low 535 and 750:840 nm depths associated with moderate 860 nm CRISM absorptions are “gray targets” from VRR that are smaller than spatial resolution of CRISM (see example “Oban” in Figure 5). Location of CRISM “hot spot” is labeled in Figure 2. CRISM parameters were calculated from FRT0000B6F1 and HRL0000BABA for areas of traverse not covered by FRT0000B6F1.

Around Sol 2,004, Curiosity was directed to a portion of the ridge that is associated with CRISM pixels that have some of the deepest 860 nm absorptions observed in the CRISM data, nicknamed the CRISM “hot spot” (Figure 2). ChemCam and Mastcam data show some rocks in that area have the deepest combined ~530 nm absorptions and strongest 840 nm downturns/860 nm band of any targets observed by Curiosity through its entire traverse through the Murray formation (Figure 8).

Where they are present, ferric spectral absorptions are intimately associated with fine‐grained bedrock targets, and they are visible throughout drilled and freshly fractured bedrock not just the rocks' surfaces. On a few occasions, Mastcam images show 530 and 860 nm absorptions consistent with red hematite are present in diagenetic features. Dark zones within calcium sulfate veins found in the Jura member of VRR have strong 530 and 860 nm absorptions. Using ChemCam LIBS elemental data, L'Haridon et al. (2020) also report small nodules with nearly pure iron oxide compositions are found within gray materials in the Jura member, although these nodules do not have any spectral absorptions in ChemCam passive spectra or Mastcam multispectral data (L'Haridon et al., 2020; Horgan et al., 2020). They hypothesize these nodules are spectrally neutral gray hematite.

**Figure 9:**
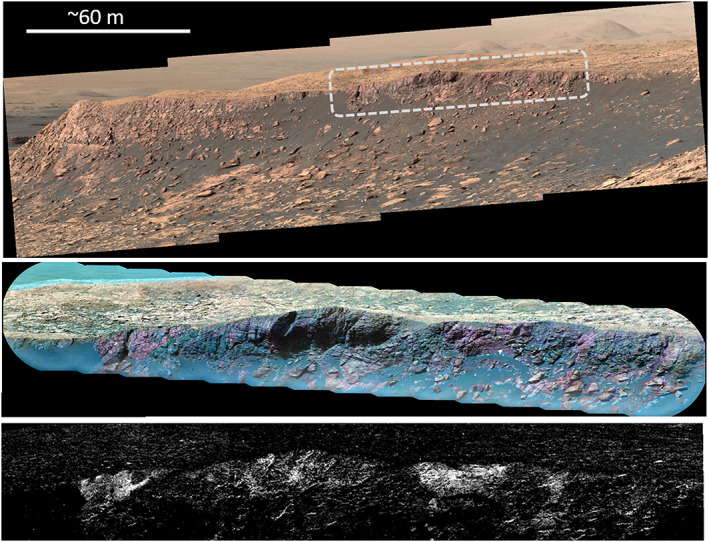
(top) Mastcam R0 mosaic of Red Cliff area, Sol 2,038, product identifiers 01076900(1–4) (middle) ChemCam RMI mosaic (CR0_578416634PRC _F0700240CCAM04038L1) of portion of Red Cliff area colorized with Mastcam false color R612 (R: 1013 nm, G: 527 nm, B: 447 nm) to emphasize spectral differences are not controlled by stratigraphy. Multispectral image product identifiers are 0107620(01–21). (bottom) Mastcam R6/R4 (1,013:908 nm) ratio that with 3 × 3 median filtered applied to reduce noise. Pixels with deep ~900 nm absorptions are white, pixels without absorptions (ratio values <1) are black.

Mastcam landscape images show spectral variations cross primary bedding. In particular, the Mastcam multispectral mosaic of the Red Cliffs area within the Pettegrove Point member shows a vertical cut through VRR. Here the transition between gray material without 527 and 867 nm absorptions and red material with deep 527 and 867 nm absorptions cross cuts primary depositional bedding (Figure 9).

### 4.2 Curiosity Comparison With CRISM

Using the same equations from Table 1, we calculated the ChemCam ferric spectral parameters for CRISM spectra along Curiosity's traverse from FRT0000B6F1 and HRL0000BABA. The CRISM data show a much smaller range of absolute variation than the in situ data, but when the *x* axis of the CRISM data is enlarged, there is a clear similarity in trends of spectral parameters versus elevation between the CRISM and Curiosity data sets (Figure 6).

In the CRISM spectra over Curiosity's traverse, both the 535 nm band depth and 750:840 nm slope increase from Pahrump Hills through the Sutton Island member. Both parameters decrease abruptly around elevation −4,300 m. Trends in the CRISM and ChemCam spectral diverge during the interval from −4,250 to −4,200 m. Here the 535 nm band depth in CRISM remains low while it increases in the ChemCam data. CRISM and ChemCam do follow each more closely in the behavior of the 750:840 nm slope in this interval, with both parameters increasing and decreasing leading up to VRR. CRISM data do not show as a wide of a spread of parameters values within VRR as ChemCam data for both parameters.

## 5 Discussion

### 5.1 Surface Characteristics That Affect Spectral Properties of Mt. Sharp Viewed From Orbit

Curiosity's in situ observations reveal at least three factors that influence how bedrock spectral properties are seen from orbit: (1) inherent bedrock variability, (2) subpixel mixing of multiple phases, and (3) dust. The evidence for each is discussed in detail below. All three contribute to why CRISM data show deep ferric absorptions at VRR.

#### 5.1.1 Inherent Bedrock Variability

The trends in the depth of ferric‐related absorption features versus elevation measured by CRISM along Curiosity's traverse are remarkably similar to analogous trends in ChemCam passive spectra from bedrock targets (Figure 6). The similarity is significant because it indicates that the large areas sampled by CRISM are generally representative of the bedrock “ground truth” provided by ChemCam. The correlation between spectral absorption depth versus elevation is less clear in comparisons between CRISM and Mastcam data (Figure 7), which we hypothesize is due to less frequent sampling with elevation. ChemCam and Mastcam data both show rocks on VRR are associated with the deepest ~535 and ~860 nm absorptions seen anywhere along Curiosity's traverse. These rocks are located within the region informally designated as the CRISM “hot spot,” where CRISM spectral data also have deep ~535 and ~860 nm absorptions (Figures 2 and 8). Combined, these observations demonstrate a major reason VRR displays deep ~535 and ~860 nm absorptions from orbit is that the VRR bedrock itself is associated with deeper ~535 and ~860 nm absorptions compared to elsewhere in Mt. Sharp.

**Figure 10:**
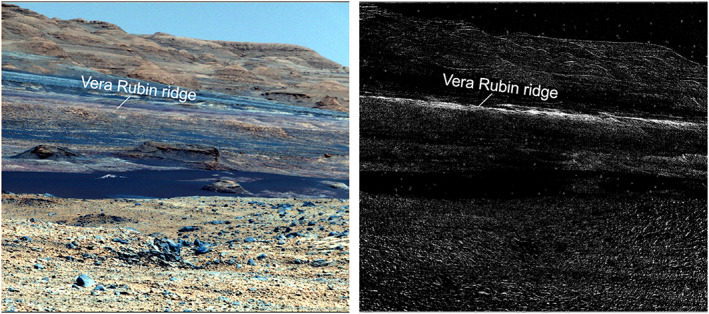
(left) Mastcam false color from Sol 468, product identifiers 00186400(0–6), showing VRR from several kilometers away. (right) R6 to R4 (1,013 to 900 nm) slope map showing the deep ferric absorptions (white) associated with the bedrock on VRR compared with surrounding Murray formation. After Johnson et al. (2016) and Wellington et al. (2017).

The first indication that VRR had unique spectral properties was actually observed on Sols 467–475 (November/December 2013), when the rover acquired long distance Mastcam multispectral and ChemCam observations of the ridge from ~5 to 6 km away. Locations from ChemCam passive observations that fell on VRR exhibit flatter spectra in the ~660 nm region, a deeper ~535 nm band than surrounding points, and a steeper downturn longward of a relative reflectance peak near 750 nm (Johnson et al., 2016). Mastcam multispectral observations also show the ridge has a distinctly deep and persistent absorption near 867 nm and a weak inflection near 527 nm (Wellington et al., 2017). From this distance, other strata on the mound do not show such distinguishable spectral features (Figure 10). The causes and implications for the deep spectral absorptions on VRR are discussed in section 5.2.

#### 5.1.2 Subpixel Mixing of Multiple Phases

Almost all ChemCam and Mastcam spectra from Murray formation bedrock observed during Curiosity's traverse below VRR have ferric absorptions. These absorptions are present but much shallower in corresponding areas in CRISM observations (Figure 6). Consequently, hematite was not interpreted to be present based on CRISM data within areas of Curiosity's traverse below VRR.

We hypothesize that mixing of multiple materials within a single CRISM pixel is one factor that influences the ability to map ferric absorptions in lower Mt. Sharp. CRISM's normal sampling of 18 m/pixel or even its best‐case along‐track sampling of 9 m/pixel means that each spectrum collected by the instrument almost always includes contributions from multiple materials. The spectral remote sensing community has addressed the challenge of understanding how constituents contribute to spectral mixtures by developing spectral unmixing algorithms (e.g., Keshava & Mustard, 2002, among many). These algorithms range from relatively straightforward linear checkerboard mixing assumptions to more complicated nonlinear models. We applied these mixing models to test whether mixing of multiple phases could explain the weaker spectral features in CRISM data.

**Figure 11:**
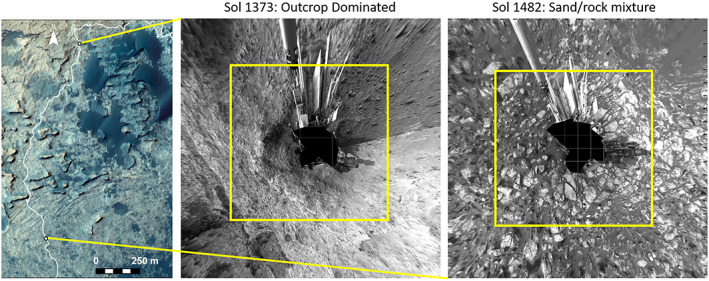
(left) HiRISE images ESP_035772_1755 and ESP_021610_1755 showing a portion of Curiosity's traverse near the Murray Buttes area. (middle) Orthorectified Navcam mosaic of a rare outcrop dominated area from Sol 1,373. (right) Orthorectified Navcam mosaic of a more typical mixed sand and bedrock area from Sol 1,482. The yellow boxes are 18 m on a side and represent the area covered by a single CRISM pixel.

The terrain properties observed on the ground guided model and end‐member selection. Below VRR, a large portion of Curiosity's traverse was dominated by a mixture of bedrock “plates” and dark basaltic sands. Orthorectified Curiosity Navcam mosaics show a single CRISM spectrum covering an 18 × 18 m area over large portions of Curiosity's traverse often contains contributions from both sand and bedrock (Figure 11). We therefore modeled CRISM pixels using simple linear unmixing models that had dark sand and hematite‐rich bedrock as end‐members. This model assumes the total surface covered by one CRISM pixel can be considered to be divided proportionally based on the fractional abundances of the sand and bedrock end‐members. This assumption is valid as long as there is not multiple scattering between components, which is reasonable in the case of the approximately meter‐scale‐sized dark sand and bedrock patches.

**Figure 12:**
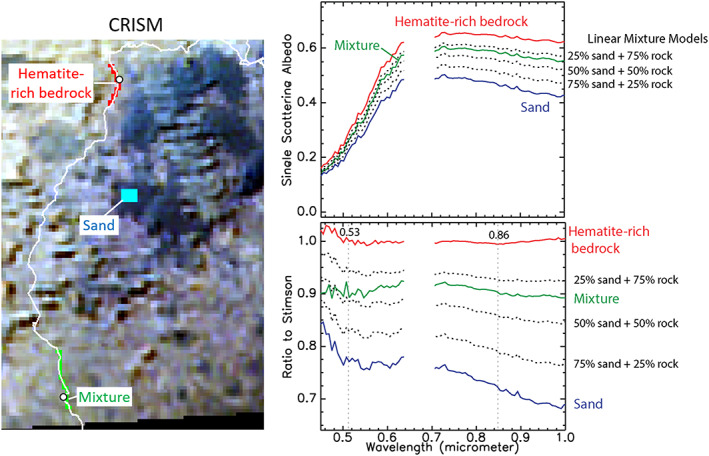
Example a of linear mixing model that simulates checkerboard mixing. (left) Regions of interest on CRISM ATO0002EC79 processed to 12 m/pixel using MLM methods and covering locations from Figure 10 as well as a nearby sand dune. (right top) Spectra from the sand area (blue), hematite‐bearing bedrock (red, hematite confirmed via the Oudam drill sample), and typical terrain along Curiosity's traverse to VRR (green). Linear mixing models (dotted lines) using the sand (blue) and bedrock (red) end‐members show the typical terrain (green) can be well modeled by using proportions that are similar to what was observed on the ground (Figure 11). (right bottom) Same as above but ratioed to a spectrum over the Stimson formation to emphasize ferric absorption features. CheMin data showed Stimson did not contain any hematite.

Figure 12 shows an example model result for one area. Sand and bedrock spectral end‐members were selected from CRISM ATO0002EC79, which had been processed to 12 m/pixel using MLM methods. The bedrock end‐member was selected from an area in orbital data where corresponding ground‐based images showed very little sand contributions. Spectra from other areas along Curiosity's traverse were successfully reproduced by linearly mixing sand and bedrock end‐member spectra in proportions that were consistent with the aerial proportion of sand cover seen on the ground in orthorectified Navcam images from those same locations (Figures 11 and 12). This finding justified selection of the two end‐members and confirmed the validity of this model.

**Figure 13:**
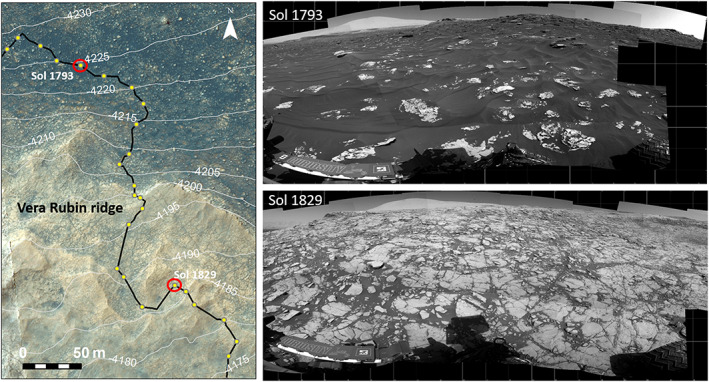
(left) Color HiRISE image ESP_021610_1755 showing show elevation interval were CRISM and ChemCam spectral parameters do not track, likely due to large amount of sand compared with bedrock. (top right) Navcam image showing typical sand‐dominated terrain in this elevation interval from Sol 1,793, elevation −4,225 m. (bottom right) Navcam showing typical bedrock‐dominated terrain for VRR from Sol 1,829, elevation −4,187 m.

These models demonstrated that only a small aerial proportion of dark sand within a CRISM pixel can dramatically reduce the apparent depth of bedrock‐ferric absorptions (Figure 12). Interestingly, the CRISM spectral parameter trends deviate from corresponding ChemCam passive trends strongly in the ~ −4,250 to −4,200 m elevation region (Figure 6), and this interval corresponds to a portion of Curiosity's traverse that was particularly sandy (Figure 13). We conclude aerial mixing effects between dark sand and bedrock strongly weaken the bedrock's contribution to the spectral signature in this particular region. Conversely, decreased sand cover on VRR (Figure 13) makes the 530 and 860 nm absorption features associated with bedrock in that area more detectable from orbit.

Although not explicitly modeled here, subpixel mixing likely also explains the divergence between CRISM and ChemCam spectral trends on VRR. The spectral variability observed on VRR, particularly the appearance of the gray materials concentrated in the Jura member, is over areas that are smaller than the spatial resolution of CRISM. The largest gray areas are only 10s of meters large, which at best is a single CRISM pixel.

#### 5.1.3 Dust

The penetration depth of light is on the order of ~10 times its wavelength (e.g., Ciani et al., 2005), so spectra collected in the VSWIR are only sensitive to the upper few micrometers of the surface. Thin layers (~5s to ~100s of micrometers depending on wavelength region) of Martian dust can therefore obscure information from underlying material in the VSWIR wavelength range (e.g., Johnson & Grundy, 2001; Johnson et al., 2003, 2004; Kinch et al., 2015). Martian dust is composed of an assortment of minerals that could include olivine, pyroxene, feldspar, carbonates, sulfates, zeolites, phyllosilicates, and poorly crystalline or nanophase iron oxides (e.g., Bandfield et al., 2003; Bell et al., 2000; Hamilton et al., 2005; Morris et al., 2000; Ruff, 2004). The spectral signature of Martian dust is relatively featureless in the VSWIR other than a steep ferric absorption from ~400 to 750 nm and a very shallow absorption near 850 nm. As described in section 2.2, the spectral properties of pigmentary (red crystalline) hematite are distinct from Martian dust.

**Figure 14:**
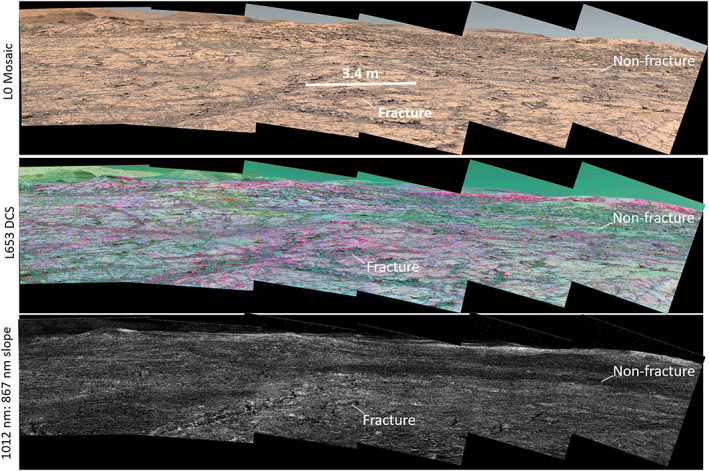
(top) Mastcam L0 Sol 1,814, product identifiers 009360(00–23), (middle) decorrelation stretch from L653 filters. Areas that are more purple have deeper ferric absorptions, (bottom) 1,012–867 nm slope map. Curiosity investigated a workspace near the labeled fracture and labeled “nonfracture” but found no chemical or spectral differences once dust was removed from the outcrops.

**Figure 15:**
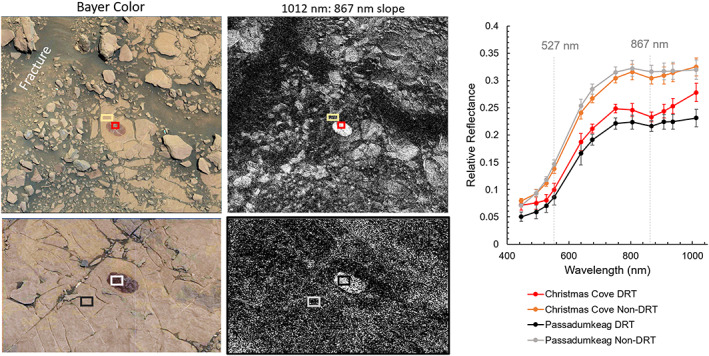
Mastcam L0 images and 1,012–867 nm slope maps, where areas in white are associated with negative slopes from 1,012 to 867 nm. Spectra (right) collected from boxes show in regions that have been cleaned with the DRT compared with typical dusty regions show the spectral properties of rocks near and away from fractures in Figure 14 are similar once some dust is removed. Christmas cove is the target near a fracture (top images, Sol 1,819, product identifiers 0093950(08–21)) and Passadeumkeag is the target far from a fracture (bottom images, Sol 1,822, product identifiers 00941100(1–7)). Location of spectra is show by boxes of corresponding colors.

Meter‐scale fractures that dissect the Pettegrove Point member of VRR show the effects of dust on surface spectral properties at the rover scale (Figure 14). These fractures are bounded by upturned centimeter‐sized blocks that create rougher surfaces compared to surrounding flat bedrock. Mastcam landscape spectral images show the rough areas near fractures are often associated with deeper 867 nm bands than smooth areas away from fractures (Figure 14), so Curiosity was sent to investigate both a fracture and an adjacent smooth area in detail. APXS, ChemCam, and Mastcam analyses of smooth surfaces that had been partially cleaned of dust using the rovers' DRT or LIBS laser shots demonstrated these areas are spectrally and chemically identical to fractured surfaces (Figure 15). We conclude the reason for the apparently stronger 867 nm absorption associated with fractures in the landscape images is because less dust has accumulated on fractures than in the smooth areas between fractures, possibly due to the near‐vertical surfaces of the fractures.

The Mastcam multispectral images demonstrated that dust masks the 867 nm absorption associated with rocks at some places on VRR. More broadly, this example emphasizes that ~860 nm absorptions can be reduced or completely obscured where dust cover is thick, so the presence of dust is another factor that influences spectral properties measured from orbit. It is not possible to determine whether there is more or less dust on VRR compared to other areas on Mt. Sharp using rover data sets, but the less‐dusty fracture edges may factor into the ability to measure an absorption 860 nm from orbit in VRR's Pettegrove Point member.

### 5.2 What Causes the Deep Absorptions in VRR Bedrock?

As demonstrated above, CRISM 860 nm band depth maps highlight VRR partially because many of the rocks within the ridge display deeper ~860 nm absorptions compared to underlying strata. What is the underlying cause of these deep absorptions?

The strength of absorptions in VSWIR reflectance data are controlled by the abundances and grain sizes of the absorbing materials (e.g., Clark, 1999). There is no evidence that VRR rocks have a greater abundance of hematite than other Mt. Sharp rocks. ChemCam LIBS and Alpha‐Particle X‐ray Spectroscopy (APXS) measurements do not show any major chemical differences between VRR rocks and rocks lower on Mt. Sharp (Frydenvang et al., 2020; Thompson et al., 2020). The crystalline mineralogy of VRR measured in drilled samples from three locations on the ridge with the CheMin XRD instrument are also very similar to mineralogy measured by CheMin from samples lower on Mt. Sharp (Rampe et al., 2020). One of the drilled samples from the ridge, “Stoer,” did contain the most hematite seen in any drilled sample from Mt. Sharp, but it was only a few weight % greater than the total hematite seen in the “Oudam” drilled sample, which was collected near the Pahrump Hills area (Rampe et al., 2020). It is worth noting that Curiosity's attempts to drill rocks with the deepest ferric absorptions within the CRISM “hot spot” were unsuccessful because the rocks there were too hard, and the drill did not reach sufficient depth to collect a sample.

In contrast, there is evidence for hematite grain size variations on VRR. “Highfield,” a gray target from the Jura member, had only very shallow absorptions at 535 and ~860 nm, yet this sample contained almost as much hematite in CheMin analyses as the nearby red “Stoer” target, which did have 535 and ~860 nm absorptions (Rampe et al., 2020). The presence of hematite in Highfield measured by CheMin and its lack of hematite‐related absorptions in ChemCam passive and Mastcam multispectral observations lead to the conclusion that this target contained larger‐grained, “gray” hematite (Rampe et al., 2020). By extrapolation, all of the gray material on the top of VRR (Figure 5) may be gray rather than red because it contains larger‐grained gray hematite. We note, magnetite, which is also gray, lacks absorption features, and was found lower on Mt. Sharp (Rampe et al., 2017), was not detected in CheMin data of Highfield (Rampe et al., 2020). We also note that although gray hematite often turns red when it is finely ground or run across a streak plate, the drilled powder from the Highfield drill sample did not turn red because the fines created by Curiosity's drill were not small enough (Rampe et al., 2020).

Given the evidence of hematite grain size variations on VRR by the presence of both red and gray hematite in the bedrock, the simplest explanation for the deep spectral absorptions in bedrock in VRR is that they are also related to grain size variations. Laboratory investigations have shown the depths of hematite absorption features are strongly dependent on grain size, and these absorptions increase and then decrease as grain sizes increase (e.g., Bell et al., 1990; Johnson et al., 2019; Morris et al., 1989; Morris et al., 2020). Alternative interpretations, however, cannot be ruled out. Jacob et al. (2020) demonstrate that the presence of Fe^3+^‐phyllosilicates also influences the depth of the 867 nm absorption measured by Mastcam multispectral data on VRR. Importantly, the types and abundance of ferric materials in the X‐ray amorphous component of the drilled samples is also largely unknown (Rampe et al., 2020), and these materials could have a strong influence on bedrock spectral properties. For example, variations in the abundance of nanophase hematite (or other nanophase ferric oxides) could be responsible for a general lack of correlation between 860 and 530 nm band depths across most of VRR (Horgan et al., 2020), as nanophase hematite does not exhibit a strong 860 nm band (Morris et al., 1989; Morris & Lauer, 1990). In reality, the deep spectral absorptions at VRR are likely due to a combination of several of these factors.

### 5.3 A Revised Orbital View of Hematite in Lower Mt. Sharp, and Associated Implications for Mt. Sharp History

We reexamined CRISM hematite maps over Mt. Sharp in light of our improved understanding of surface properties that contribute to orbital data. Despite the fact that CRISM spectra along Curiosity's traverse appeared to have weak to no hematite‐related absorptions on Mt. Sharp, CheMin detected more than 2 wt. % hematite along with other oxidized phases in seven of the nine drilled rock samples collected from Mt. Sharp below VRR (Achilles, 2018; Bristow et al., 2018; Morrison et al., 2018; Rampe et al., 2017). In addition to oxidized phases in the crystalline component, Fe‐phases are also present in the ubiquitous X‐ray amorphous materials from these drilled samples, although the oxidation state of these material is not known (Achilles, 2018). The bedrock along Curiosity's path up Mt. Sharp is almost always associated with ferric absorptions (Figures 6 and 7), but mixing with dark sand occurs often in corresponding CRISM observations (Figures 12 and 13).

**Figure 16:**
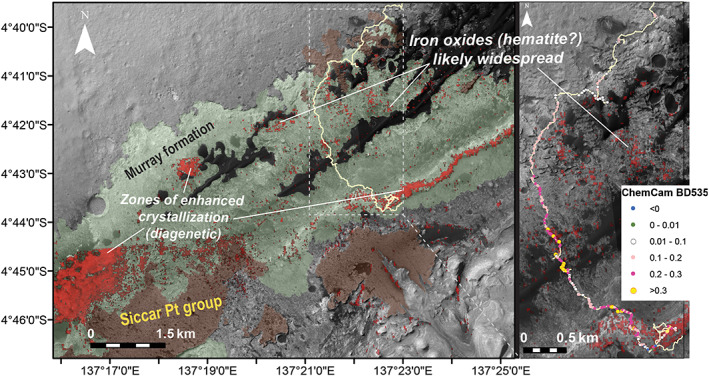
(left) Annotated HiRISE mosaic of Mt. Sharp showing likely extent of the Murray formation based on rover observations to date (green) and Siccar Point group units (brown). CRISM hematite map from Fraeman et al., 2016 shown in red. (right) Graphical representation of ChemCam 535 nm band depths from rocks along Curiosity's traverse.

Based on this finding and the role of sand and dust in reducing apparent depths of ferric absorptions described above, we argue that weak ferric absorptions in CRISM spectra of the Murray formation that were initially dismissed as noise (band depth 0.01–1%) (Figure 16) are attributable to absorptions from rocks in those areas. If true, hematite or other ferric oxides would be much more pervasive throughout lower Mt. Sharp that previously reported. We do caution that hematite is not present throughout the entirety of the Murray formation. For example, Curiosity observed one interval near the Buckskin drill hole (elevation −4,447 m) that CheMin data showed was dominated by high concentrations of silica accompanied by magnetite rather than hematite (Rampe et al., 2017).

In addition to VRR, several other areas within orbital‐based maps of the Murray formation in the northwest portion of Mt. Sharp are associated with contiguous CRISM pixels that have deep 535 and 860 nm absorptions and a peak at 750 nm (Figure 16) (Fraeman et al., 2016; Milliken et al., 2010). Seelos et al. (2019) additionally detected several strong ferric absorptions attributed to hematite in the lower elevations (also likely Murray formation) of the western and southern portion of the mound. By analogy with VRR, we assume that these areas also exhibit deeper ferric absorptions within the bedrock, as well as less sand and dust. If we use elevation as a proxy for stratigraphic position (Grotzinger et al., 2015), we conclude the process(es) that caused the deepening of the ferric spectral absorptions are not confined to a specific elevation interval. This observation is consistent with meter‐scale Mastcam landscape images from VRR that show strong hematite‐related spectral variations cross‐cut primary strata within the ridge (Figure 8).

We propose a model where late‐stage diagenetic fluids affected the grain size of ferric phases in Mt. Sharp, which lead to the deep spectral absorptions and presence of red and gray hematite observed at VRR. Intriguingly, many of the areas in orbital data with the deepest hematite signatures are close to exposures of Siccar Point group rocks (Figure 16; Seelos et al., 2019). Rocks within the Siccar Point group unconformably overlie Mt. Sharp group rocks, and they have been mapped as orbitally defined units referred to as the mound skirting unit, washboard terrain, lower mound marginal unit, and high thermal inertia unit (Anderson & Bell, 2010; Buz et al., 2017; Fraeman et al., 2016; Milliken et al., 2014; Thomson et al., 2011). Diagenetic fluids that formed the patches of the Murray formation that have particularly strong ferric absorptions, such as VRR, may have been focused by the contact between the Murray formation and Siccar Point group. Similar scenarios have been proposed in Kronyak et al. (2019) and Rampe et al. (2020).

### 5.4 Lessons Learned About CRISM Data Interpretation

Curiosity's campaign at VRR adds to a list of examples that demonstrate the power of coordinated CRISM and rover investigations in Mars exploration. Previously, Curiosity's observations of the Bagnold dune field were used to test hypotheses about eolian grain density sorting and the accuracy of spectral abundance models (Bridges & Ehlmann, 2018; Lapotre et al., 2017; Seelos et al., 2014). The Mars Exploration Rover (MER) Spirit's in situ discovery of carbonates in Columbia Hills motivated a close examination of CRISM data over the area, which resulted in discoveries of additional carbonate‐bearing outcrops, new detections of phyllosilicates, and expanded our knowledge of the aqueous history of the area (Carter & Poulet, 2012; Morris et al., 2010). At MER Opportunity's landing site in Meridiani Planum, CRISM data were used to document the full extent of the sulfates associated with the Burns formation, providing further evidence to support the hypothesis that they were formed through regional‐scale processes (Arvidson et al., 2015; Poulet et al., 2008; Wiseman et al., 2010). Finally, the Opportunity rover's exploration of the rim of Endeavour crater was guided by CRISM detections of Fe/Mg phyllosilicates in specific outcrops. Opportunity's in situ measurements at Cape York and Marathon Valley revealed that the phyllosilicates detected by CRISM formed post impact by fracture‐driven fluids (Arvidson et al., 2014; Fox et al., 2016; Wray et al., 2009).

This study demonstrates that interpretation from CRISM data can be validated through knowledge of surface properties from in situ observations. Curiosity confirmed hematite was present at VRR as predicted by CRISM. To first order, this provides continued confidence in the accuracy of mineral identifications based on spectral absorptions viewed from orbit. Additionally, with some knowledge of surface properties from in situ observations, CRISM can be extrapolated to regions beyond a narrow traverse with much more confidence. In return, CRISM data successfully highlight outcrops with scientifically important compositions for rovers to study in situ.

The experiences at VRR also emphasize caveats that should be taken when making geologic interpretations based on the apparent lack of mineral spectral absorptions. The original hypothesis that VRR was an isolated oxidized interval in Mt. Sharp was not correct (Fraeman et al., 2013), as Curiosity observations have demonstrated that crystalline ferric phases are present north of VRR (Achilles, 2018; Bristow et al., 2018; Morrison et al., 2018; Rampe et al., 2017). In addition to the complicating factors of dust and subpixel mixing described here, previous investigations have also shown that the surface textures, photometric angles, and atmospheric contribution have a significant influence on the strength and interpretation of spectral signatures (e.g., Fernando et al., 2013, 2015; Jehl et al., 2008; Johnson et al., 2008; Pinet et al., 2014). Synergistic studies on Earth combining airborne and field work have demonstrated (1) the complexity of the spectral signal linked to the photometric variability associated with the observing geometry conditions across the field of view (e.g., Pinet et al., 2017) and (2) the significant effort which may be needed to be able to identify in situ the precise geomorphological source (outcrops, bedrocks, sands) of the material responsible for the unambiguous spectroscopic signature detected by airborne (orbital) assets (e.g., Clénet et al., 2010). In situ observations are critical for refining orbital interpretations because they provide the high‐spatial resolution information necessary to resolve geologic setting and context, including what other phases are or are not present.

Curiosity will continue to use CRISM data for path planning as the rover climbs Mt. Sharp, as will future landed missions to Mars. When utilizing CRISM data to determine where to drive rovers, it is important to continue to understand that orbital observations are influenced by a wide variety of physical and compositional properties, any of which can have major spectral influences. The strategy of driving rovers to the exact CRISM pixel with the deepest absorption feature is key to understanding the source of orbital signatures, but does not guarantee that this area is a compositional end‐member or most important for understanding geologic history. For strategic path planning, CRISM data are most valuable when used in coordination with geologic maps derived from high resolution orbital images and reconnaissance images from the rover itself.

## 6 Summary

We used in situ spectral, chemical, and mineralogical analyses by Curiosity in lower Mt. Sharp to refine interpretations about the distribution of hematite through the mound along Curiosity's traverse as well as throughout the mound, which provided new insight into regional scale processes in lower Mt. Sharp. Key findings are as follows:

1. Multiple in situ instruments and CRISM data converged on the same interpretation that crystalline red hematite is present in VRR.
2. CRISM 860 nm band depth maps highlight VRR mainly because many of the rocks within the ridge are associated with deeper ~860 nm absorptions compared to underlying strata. Dust and soil coverage also influence the visibility of spectral absorptions at VRR and throughout the Murray formation.
3. The deeper spectral absorptions at VRR are likely caused by variations in grain sizes. The deep ferric absorptions at VRR are not caused by more abundant crystalline ferric phases.
4. Hematite and other ferric phases are likely more widespread throughout the Murray formation that originally reported based on orbital data alone. Their detection from orbit is hampered by dust and mixing with dark sand.
5. Variations in depth of ferric absorptions crosscuts stratigraphy; secondary processes are responsible for the strong hematite spectral signature in VRR.
6. Using Curiosity's exploration at VRR, we have demonstrated that with some knowledge of surface properties from in situ observations, CRISM can be extrapolated to regions beyond a narrow traverse with much more confidence. In return, CRISM data successfully highlight outcrops with scientifically important compositions for rovers to study in situ.
7. Combined, these experiences demonstrate the enriched nature of exploration with CRISM and rover data sets at multiple spatial scales. The present results obtained at Gale Crater hold significant promise for future exploration strategies relying on a combination of orbital and in situ (hyper)spectral observations.

## Supporting information

Supporting Information S1

Table S1

Table S2

## Data Availability

All derived data products used to reproduce the results from this paper are available for downloading from the Zenodo open data repository (Fraeman, 2020).
