# Supplementary material for: Synergistic Ground and Orbital Observations of Iron Oxides on Mt. Sharp and Vera Rubin Ridge
Source: J Geophys Res Planets. 2020 Sep 23;125(9):e2019JE006294. doi: 10.1029/2019JE006294 (PMC7539960; doi:10.1029/2019JE006294)
Supplement: Supplementary file 1 — Supporting Information S1 [file JGRE-125-e2019JE006294-s001.docx]

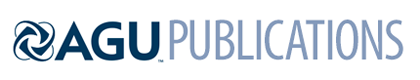


*Journal of Geophysical Research: Planets*

Supporting Information for

**Synergistic ground and orbital observations of iron oxides on Mt. Sharp and Vera Rubin ridge**

A.A. Fraeman^1^, J.R. Johnson^2^, R.E. Arvidson^3^, M.S. Rice^4^, S.R. Jacob^5^, V.Z. Sun^1^, D.F. Wellington^5^, B.H. Horgan^6^, V.K. Fox^7^, R.V. Morris^8^, M.R. Salvatore^9^, P. Pinet^10^, J.F. Bell III^5^, R.C. Wiens^11^, A.R. Vasavada^1^

^1^Jet Propulsion Laboratory, California Institute of Technology, Pasadena, CA, USA

^2^The Johns Hopkins University Applied Physics Laboratory, Laurel, Maryland, USA

^3^Department of Earth and Planetary Sciences, Washington University, St. Louis, Missouri, USA

^4^Geology Department, Physics and Astronomy Department, Western Washington University, Bellingham, Washington, USA

^5^School of Earth and Space Exploration, Arizona State University, Tempe, AZ, USA

^6^Department of Earth, Atmospheric, and Planetary Sciences, Purdue University, West Lafayette, Indiana, USA

^7^Division of Geological and Planetary Sciences, California Institute of Technology, Pasadena, California, USA

^8^NASA Johnson Space Center, Houston, Texas, USA

^9^Northern Arizona University, Flagstaff, AZ, USA

^10^Institut de Recherche en Astrophysique et Planétologie, Université de Toulouse, CNRS, UPS, CNES, Toulouse, France

^11^Los Alamos National Laboratory, Los Alamos, NM, USA

**Additional Supporting Information (Files uploaded separately)**

Captions for Tables S1 to S2

**Introduction**

These files list the Mastcam multispectral and ChemCam passive observations that were used to create figures 6, 7, and 10 in the main manuscript. All data can be accessed from the online Planetary Data System Geosciences Node.

Table S1.

Soil, sequence IDs, filters, target names, local true solar time, and rover elevation for all Mastcam multispectral images used to create figure 7.

Table S2.

File name, target name, LIBS shot number, sol, and rover elevation for ChemCam passive spectral data used to create figure 6 and 10.
