# Supplementary material for: Synergistic Ground and Orbital Observations of Iron Oxides on Mt. Sharp and Vera Rubin Ridge
Source: J Geophys Res Planets. 2020 Sep 23;125(9):e2019JE006294. doi: 10.1029/2019JE006294 (PMC7539960; doi:10.1029/2019JE006294)
Supplement: Supplementary file 2 — Table S1 [file JGRE-125-e2019JE006294-s002.pdf]

Sol	Seq ID	Filters	Target Name	LTST	Rover Elevation
758	mcam03257	L0-6, R0-6	Confidence Hills	12:07:59	-4460.6216
762	mcam03273	L0-6, R0-6	Confidence Hills 2x1	13:20:12	-4460.6216
771	mcam03310	L0-6, R0-6	Comb Ridge	11:34:40	-4460.6216
773	mcam03347	L0-6, R0-6	Confidence Hills Dump	11:13:31	-4460.6216
782	mcam03413	L0-6, R0-6	Confidence Hills Dump 2	11:42:04	-4458.9824
807	mcam03538	L0-6, R0-6	Pelona Ricardo 1x2	11:44:23	-4460.9048
812	mcam03564	L0-6, R0-6	Mojave	12:51:49	-4459.4614
812	mcam03565	L0-6, R0-6	Rosamund	12:54:26	-4459.4614
816	mcam03594	L0-6, R0-6	Topanga	11:49:06	-4458.562
816	mcam03595	L0-6, R0-6	Afton Canyon	11:51:38	-4458.562
816	mcam03596	L0-6, R0-6	Punchbowl	11:54:27	-4458.562
822	mcam03618	L0-6, R0-6	Mescal	11:22:57	-4456.875
826	mcam03629	L0-6, R0-6	Puente	11:35:50	-4456.875
832	mcam03653	L0-6, R0-6	Pickhandle	11:11:28	-4455.2041
832	mcam03654	L0-6, R0-6	Goldstone	11:14:09	-4455.2041
837	mcam03675	L0-6, R0-6	Cowhole Mountain	11:59:59	-4453.7212
845	mcam03762	L0-6, R0-6	Santa Ana	12:09:00	-4452.6309
855	mcam03777	L0-6, R0-6	Tecoya	11:55:11	-4452.6309
868	mcam03812	L0-6, R0-6	Mini Drill Tailings	12:38:01	-4459.7119
883	mcam03851	L0-6, R0-6	Mojave2 Drill Tailings	12:06:50	-4459.7119
895	mcam03933	L0-6, R0-6	Mojave2 Postsieve Dump	11:56:45	-4459.7119
909	mcam03977	L0-6, R0-6	Telegraph Peak Tailings	11:36:39	-4453.5083
929	mcam04084	L0-6, R0-6	Sanpete	12:15:14	-4451.0293
929	mcam04085	L0-6, R0-6	Hoskinnini Ouray	12:17:59	-4451.0293
935	mcam04103	L0-6, R0-6	Aneth	11:29:00	-4451.0293
939	mcam04120	L0-6, R0-6	Hyrum	12:07:12	-4451.0293
939	mcam04121	L0-6, R0-6	Telegraph Peak Dump	12:10:00	-4451.0293
946	mcam04158	L0-6, R0-6	Amboy Jacumba	12:28:11	-4451.042
949	mcam04170	L0-6, R0-6	Anza Borrego	11:30:10	-4451.042
955	mcam04222	L0-6, R0-6	Telegraph Peak Dump	11:18:32	-4452.3652
976	mcam04311	L0-6, R0-6	Albert	10:55:03	-4456.2212
990	mcam04372	L0-6, R0-6	Pinedale	10:48:43	-4454.9189
994	mcam04399	L0-6, R0-6	Red Sleep 1x2 Rall	11:45:06	-4446.9775
999	mcam04430	L0-6, R0-6	Seely	11:05:38	-4446.7397
1001	mcam04465	L0-6, R0-6	Wallace	10:53:11	-4446.7397
1030	mcam04498	L0-6, R0-6	Lowary	11:32:00	-4446.7397
1032	mcam04512	L0-6, R0-6	Lowary 2	12:09:16	-4446.939
1033	mcam04517	L0-6, R0-6	Thunderbolt	11:24:51	-4446.939
1034	mcam04526	L0-6, R0-6	Seeley	11:34:11	-4446.939
1041	mcam04560	R0-6	Elk	11:51:16	-4447.5078
1041	mcam04561	R0-6	Lamoose	11:53:14	-4447.5078
1041	mcam04562	L0-6, R0-6	Mosquito Frog	11:55:41	-4447.5078
1062	mcam04672	L0-6, R0-6	Buckskin Drill Tailings	13:00:54	-4446.8306
1066	mcam04689	L0-6, R0-6	Buckskin Presieve Dump	12:23:47	-4446.8306
1090	mcam04784	L0-6, R0-6	Buckskin Dump Pile	11:21:29	-4437.7095

1160	mcam05245	L0-6, R0-6	Bogenfels	12:14:39	-4432.2305
1167	mcam05284	L0-6, R0-6	Garub	11:56:24	-4432.3184
1183	mcam05361	L0-6, R0-6	Narabeb	12:07:54	-4424.5732
1204	mcam05546	L0-6, R0-6	Greenhorn Dump Pile	13:20:26	-4419.91
1219	mcam05592	L0-6, R0-6	Solitaire	12:32:05	-4424.15
1234	mcam05705	L0-6, R0-6	Sanitatis	12:55:37	-4423.76
1246	mcam05813	L0-6, R0-6	Kudis	12:06:25	-4423.43
1252	mcam05846	L0-6, R0-6	Fiskus	12:50:52	-4426.54
1253	mcam05852	L0-6, R0-6	Kuiseb, Dumps R3x1	13:18:14	-4426.54
1260	mcam05889	L0-6, R0-6	Gorob DRT	12:31:56	-4427.63
1267	mcam05932	L0-6, R0-6	Stockdale DRT	11:52:54	-4432.08
1273	mcam05953	L0-6, R0-6	Murray Stimson Contact	12:32:10	-4432.62
1274	mcam05965	L0-6, R0-6	Schwarzrand DRT	12:40:02	-4432.62
1276	mcam05989	L0-6, R0-6	Mirabib Lall 2x1 Rall	13:22:36	-4427.36
1355	mcam06577	L0-6, R0-6	Kunjas	12:43:15	-4434.87
1355	mcam06578	L0-6, R0-6	Navachab	12:46:16	-4434.87
1356	mcam06595	L0-6, R0-6	Inamagando	11:58:47	-4434.87
1359	mcam06626	L0-6, R0-6	Okoruso Dump Oudam	12:44:25	-4435.59
1363	mcam06671	L0-6, R0-6	Oudam Drill Hole	12:45:04	-4435.59
1366	mcam06683	L0-6, R0-6	Oudam Presieve Dump	12:27:58	-4435.59
1368	mcam06691	L0-6, R0-6	Aubures	13:07:27	-4435.59
1373	mcam06720	L0-6, R0-6	Uis	13:10:56	-4430.25
1382	mcam06774	L0-6, R0-6	Koes	11:51:07	-4428.65
1405	mcam06879	L0-6	Galo	13:16:54	-4417.31
1405	mcam06880	L0-6	Guri	13:19:03	-4417.31
1417	mcam06946	L0-6, R0-6	Chibia DRT	12:39:09	-4410.83
1421	mcam07009	L0-6, R0-6	Marimba Drill Tailings	13:23:04	-4410.38
1421	mcam07011	L0-6, R0-6	Oudam Postsieve Dump	13:30:13	-4410.38
1425	mcam07034	L0-6, R0-6	Marimba2 Drill Tailings	11:37:33	-4410.38
1427	mcam07056	L0-6, R0-6	Marimba Presieve Dump	12:51:29	-4410.38
1438	mcam07111	L0-6, R0-6	Conda DRT	11:30:16	-4398.71
1445	mcam07152	L0-6, R0-6	Ganda DRT	12:40:58	-4393.77
1458	mcam07231	L0-6, R0-6	Marimba Post Sieve	12:12:16	-4379.46
1459	mcam07236	L0-6, R0-6	Quela DRT	12:11:30	-4379.46
1460	mcam07283	L0-6, R0-6	Cuimba	11:47:25	-4379.46
1465	mcam07323	L0-6, R0-6	Quela Drill Tailings	11:59:33	-4379.46
1467	mcam07337	L0-6, R0-6	Quela Presieve Dump	11:36:09	-4379.46
1475	mcam07395	L0-6, R0-6	Jwaneng	12:12:53	-4371.23
1475	mcam07396	L0-6, R0-6	Kopong Munhango	12:16:54	-4371.23
1478	mcam07422	L0-6, R0-6	Catumbela Caungula	12:22:12	-4370.07
1485	mcam07463	L0-6, R0-6	Serowe	12:09:15	-4364.32
1492	mcam07509	L0-6, R0-6	Sebina DRT + Quela	12:01:58	-4360.75
1493	mcam07516	L0-6, R0-6	Cahama	12:05:50	-4360.75
1496	mcam07566	L0-6, R0-6	Sebina Tailings	11:40:34	-4360.75
1498	mcam07582	L0-6, R0-6	Sebina Presieve Dump	12:12:46	-4360.75
1505	mcam07641	L0-6, R0-6	Thrumcap	12:04:40	-4351.81
1505	mcam07642	L0-6, R0-6	Egg Rock	12:06:52	-4351.81

1508	mcam07661	L0-6, R0-6	Thunder Hole	11:31:08	-4349.51
1512	mcam07688	L0-6, R0-6	Penobscot DRT	11:34:20	-4349.78
1520	mcam07741	R046	Hematite Ridge	11:56:23	-4337.92
1524	mcam07770	L0-6, R0-6	SuttonIsland Manset 2x1	11:55:47	-4336.36
1532	mcam07809	L0-6, R0-6	Precipice DRT	12:02:21	-4335.8
1534	mcam07824	L0-6, R0-6	Sebina Post Sieve Dump	11:26:57	-4335.8
1552	mcam07972	L0-6, R0-6	Western Head	11:49:03	-4335.8
1566	mcam07987	L0-6, R0-6	Old Soaker	12:31:57	-4335.75
1566	mcam07988	L0-6, R0-6	Schooner Head	12:34:51	-4335.75
1571	mcam07999	L0-6, R0-6	Greening Island	11:34:24	-4335.75
1587	mcam08091	L0-6, R0-6	Belle Lake DRT	11:44:25	-4317.52
1592	mcam08115	L0-6, R0-6	Hematite Ridge	11:56:42	-4311.29
1596	mcam08130	L0-6, R0-6	Parker Bog	12:02:53	-4310.43
1608	mcam08214	L0-6, R0-6	Quoddy Quimby	11:38:08	-4313.46
1608	mcam08215	L0-6, R0-6	Jemtland	11:41:59	-4313.46
1610	mcam08231	L0-6, R0-6	Perry	11:17:57	-4313.44
1610	mcam08232	L0-6, R0-6	Spurwink	11:22:48	-4313.44
1624	mcam08347	L0-6, R0-6	Dunn Brook	11:48:30	-4306.63
1634	mcam08440	L0-6, R0-6	North Haven	12:07:16	-4304.13
1657	mcam08597	L0-6	Gts Fivemile	11:03:07	-4299.95
1666	mcam08643	L0-6, R0-6	Moosehead Lake	11:44:37	-4298.57
1682	mcam08745	L0-6, R0-6	Duck Brook Bridge DRT	11:43:07	-4286.24
1688	mcam08786	L0-6, R0-6	Eddie Brook	12:46:37	-4280.45
1693	mcam08822	L0-6, R0-6	Bear Island	12:03:36	-4279.4
1696	mcam08845	L0-6, R0-6	Mason Point DRT	10:58:49	-4279.19
1703	mcam08884	L0-6, R0-6	Broad Cove	11:20:06	-4270.38
1711	mcam08923	L0-6, R0-6	White Ledge	12:06:12	-4267.57
1714	mcam08948	L0-6, R0-6	Heron Island	13:03:22	-4265.51
1725	mcam09004	L0-6, R0-6	Haynes Point	12:51:30	-4253.95
1727	mcam09015	L0-6, R0-6	Freeman Ridge	11:59:06	-4251.66
1728	mcam09026	L0-6, R0-6	Fernald Point	11:53:47	-4248.72
1729	mcam09034	L0-6, R0-6	Mark Island	12:08:40	-4247.8
1734	mcam09057	L0-6, R0-6	Northern Neck	12:07:54	-4246.16
1737	mcam09080	L0-6, R0-6	Winter Harbor DRT	12:25:15	-4246.41
1745	mcam09118	L0-6, R0-6	Jobbies Mingo	11:53:11	-4243.45
1745	mcam09120	L0-6, R0-6	Ld VRR	12:00:00	-4243.45
1790	mcam09243	L0-6, R0-6	Zephyr Ledges 2x1	11:46:36	-4225.13
1797	mcam09276	L0-6, R0-6	Bois Bubert Stereo	13:22:03	-4217.47
1805	mcam09310	R0-6	Toothacher Island	12:57:53	-4206.88
1807	mcam09325	L0-6, R0-6	Robinson Rock Gunning	12:38:56	-4206.88
1812	mcam09351	L0-6, R0-6	Kemps Folly Stereo	12:49:53	-4200.1
1814	mcam09364	L0356	Weymouth Point	12:16:47	-4198.86
1819	mcam09395	L0-6, R0-6	Christmas Cove Stereo	12:22:38	-4196.55
1819	mcam09396	R0-6	Jaquish Ledge	12:24:15	-4196.55
1821	mcam09401	L0-6, R0-6	Mustards Island	12:31:31	-4195.37
1822	mcam09411	L0-6, R0-6	Passadumkeag Stereo	12:06:29	-4195.37
1827	mcam09449	L0-6, R0-6	Sherwood Tableland	12:04:44	-4191.61

1837	mcam09630	L0-6, R0-6	Ecca Lucknow DRT 2x1	12:08:45	-4182.32
1843	mcam09640	L0-6, R0-6	Iron Mask Stereo	12:32:34	-4180.47
1850	mcam09674	L0-6, R0-6	Zeederberg Stereo	12:07:02	-4176.74
1850	mcam09675	L0-6, R0-6	Blinkberg Stereo	12:09:33	-4176.74
1852	mcam09683	L0356	VRR Region 7	12:14:11	-4174.11
1864	mcam09742	R0-6	Sibasa Drt	11:54:24	-4174.11
1866	mcam09746	L0-6, R0-6	VRR Region 6c	11:38:45	-4174.66
1871	mcam09783	L0-6, R0-6	Platberg DRT Stereo	13:06:49	-4170.47
1872	mcam09789	L0-6, R0-6	Frisco Stereo	12:01:06	-4168.45
1876	mcam09813	L0-6, R0-6	Fort Brown DRT	12:04:02	-4168.34
1876	mcam09814	L0-6, R0-6	Middleton	12:05:35	-4168.34
1877	mcam09822	L0-6, R0-6	Table Mountain 2x1	12:13:30	-4168.34
1879	mcam09830	L0-6, R0-6	Natal Stereo	11:55:32	-4166.6
1885	mcam09853	L0-6, R0-6	Hexriver Stereo	11:46:09	-4166.6
1893	mcam09909	L0-6, R0-6	Drakensberg DRT Stereo	12:36:08	-4157.98
1893	mcam09911	L0-6, R0-6	Kamden 1x2	12:44:51	-4157.98
1899	mcam09933	L0-6, R0-6	Farr 2x1	12:02:10	-4157.04
1899	mcam09934	L0-6, R0-6	Muck	12:05:41	-4157.04
1905	mcam09994	L0-6, R0-6	Oban Laphroaig	12:58:29	-4154.57
1906	mcam10003	L0-6, R0-6	Holyrood Haddo House	12:25:26	-4152.91
1936	mcam10107	L0-6, R0-6	Unst Ross of Mull 2x1	12:51:08	-4149.29
1939	mcam10132	L0-6, R0-6	Bowmore Stereo L2x1 R3x1	12:24:44	-4149.29
1950	mcam10202	L0-6, R0-6	Balmedie Stereo 2x1	12:53:56	-4146.66
1959	mcam10243	L0-6, R0-6	Cocksburnpath_hara 2x1	12:02:41	-4146.38
1964	mcam10267	L0-6, R0-6	Newmachar DRT	12:11:35	-4146.8
1964	mcam10269	L0-6, R0-6	St Kilda	12:19:06	-4146.8
1964	mcam10270	L0-6, R0-6	Benbecula	12:21:05	-4146.8
1967	mcam10287	L0-6, R0-6	Loch Ba	12:42:28	-4146.8
1967	mcam10288	L0-6, R0-6	Lake Orcadie DRT	12:46:12	-4146.8
1970	mcam10306	L0-6, R0-6	Ogunquit Dump R2x1	12:07:10	-4146.8
1978	mcam10342	L0-6, R0-6	Lake Orcadie Tailings	11:23:48	-4146.8
1980	mcam10366	L0-6, R0-6	Lake Orcadie 2 DRT	12:41:01	-4146.8
1984	mcam10386	L0-6, R0-6	Lake Orcadie2 Drill Hole	11:58:28	-4146.8
1986	mcam10410	L0-6, R0-6	Braemar	12:08:07	-4147.32
1996	mcam10460	L0-6, R0-6	Durness Stereo	12:37:20	-4154.2
1998	mcam10471	L0-6	Red Hills 4x1	12:22:59	-4155.42
2000	mcam10487	L0356	Region 12 3x1	12:01:16	-4159.72
2002	mcam10505	L0-6, R0-6	VRR Region 12	13:05:48	-4159.72
2002	mcam10507	L0-6, R0-6	Sgurr of Eig & Brora	13:14:52	-4159.72
2004	mcam10518	L0-6, R0-6	Mousa Stereo	11:41:27	-4159.74
2005	mcam10524	L0-6, R0-6	Port Ellen Stereo	12:11:05	-4164.62
2006	mcam10558	L0356	Region 13 5x1	11:21:25	-4164.62
2007	mcam10560	L0-6, R0-6	Stranraer DRT	11:17:47	-4164.62
2007	mcam10561	L0-6, R0-6	Burghead Stereo	11:21:52	-4164.62
2007	mcam10562	L0-6, R0-6	Walls Peninsula Stereo	11:24:26	-4164.62
2009	mcam10581	L0-6, R0-6	Lanark DRT	11:20:27	-4160.97
2009	mcam10583	L0-6, R0-6	Stac Fada 2x1/3x3	11:26:47	-4160.97

2013	mcam10610	L0-6	Galloway 4x1	11:29:28	-4155.39
2014	mcam10624	L0-6, R0-6	Lingarabay DRT	12:50:14	-4155.39
2016	mcam10636	L0-6, R0-6	Askival Lall 1x3 Rall	11:48:09	-4152.72
2029	mcam10709	L0-6, R0-6	Taconite Stereo	11:46:22	-4165.89
2034	mcam10737	L0-6, R0-6	Logan	12:40:55	-4168.18
2036	mcam10747	L0-6, R0-6	Britt Stereo	12:11:25	-4175.97
2036	mcam10749	L0-6, R0-6	Aurora Stereo	12:18:04	-4175.97
2041	mcam10786	L0-6, R0-6	Barto Lake & Homer Lake	12:32:19	-4178.79
2043	mcam10810	L0-6, R0-6	Pigeon River & Bald Eagle Lake 2x1	11:31:18	-4179.22
2046	mcam10829	L0-6, R0-6	Mud Lake Stereo	13:01:35	-4180.64
2050	mcam10863	L0-6, R0-6	Giants Range and Bilbert	12:18:26	-4187.41
2050	mcam10865	L0-6, R0-6	Floodwood DRT Stereo	12:25:51	-4187.41
2056	mcam10897	L0-6, R0-6	Duluth DRT stereo	12:21:29	-4191.28
2059	mcam10916	L0-6, R0-6	Duluth Drill Stereo	11:32:18	-4191.28
2081	mcam11061	L0-6, R0-6	Ambridge Stereo	13:12:41	-4191.28
2081	mcam11063	L0-6, R0-6	Duluth Dump Pile Stereo	13:20:14	-4191.28
2091	mcam11157	L0-6, R0-6	Caribou lake	12:21:43	-4192.42
2101	mcam11212	L0-6, R0-6	Dumbarton_rock	11:10:11	-4164.83
2110	mcam11271	L0-6, R0-6	Voyageurs Stereo	12:58:33	-4164.35
2113	mcam11291	L0-6, R0-6	Voyageurs Drill Hole	12:09:53	-4164.35
2120	mcam11351	L0-6, R0-6	Squrrofeigg Appin Brora	12:26:12	-4157.64
2122	mcam11363	L0-6, R0-6	Ailsa Craig Stereo	12:15:54	-4158.73
2123	mcam11371	L0-6, R0-6	Ailsa Craig Stereo	11:37:47	-4158.73
2123	mcam11373	L0356	Peterhead	11:43:06	-4158.73
2123	mcam11374	L0356	Taconite Crater Ejecta	11:44:25	-4158.73
2124	mcam11377	L0-6, R0-6	Lamlash Bay	12:15:24	-4158.73
2132	mcam11431	L0-6, R0-6	Dobbs Linn Stereo	12:24:20	-4167.55
2135	mcam11450	L0-6, R0-6	Stoer DRT	12:08:44	-4169.04
2138	mcam11468	L0-6, R0-6	Stoer Drill Tailings	11:53:20	-4169.04
2147	mcam11535	L0-6, R0-6	Pentland_Hills	12:06:47	-4169.04
2147	mcam11536	L0-6, R0-6	Strontian Stereo	12:09:49	-4169.04
2153	mcam11593	L0-6, R0-6	Stoer Portion	11:42:39	-4169.04
2160	mcam11633	L0-6, R0-6	Rosie	12:35:46	-4156.63
2160	mcam11634	L0-6, R0-6	Rhinns of Galloway	12:38:18	-4156.63
2165	mcam11658	L0-6, R0-6	Great Todday	12:02:49	-4150.12
2169	mcam11680	L0-6, R0-6	Inverness DRT	12:13:49	-4149.87
2169	mcam11681	L0-6, R0-6	Stoneyburn	12:16:35	-4149.87
2171	mcam11693	L0-6, R0-6	Inverness Tailings	12:04:20	-4149.87
2217	mcam11747	L0-6, R0-6	Inverness	12:39:51	-4150.11
2222	mcam11774	L0356	Lake Orcadie Area 2x1	11:36:47	-4146.67
2225	mcam11791	L0-6, R0-6	Highfield Drill Tailing	11:26:28	-4146.95
2229	mcam11824	L0356	Slate Islands	11:34:42	-4146.95
2229	mcam11825	L0356	Loch Ba 3x1	11:36:06	-4146.95
2231	mcam11838	L0-6, R0-6	Slate Islands	11:59:51	-4146.95
2231	mcam11839	L0-6, R0-6	Little Todday	12:02:15	-4146.95
2235	mcam11884	L0356	Falls of Clyde	11:08:54	-4146.95
2246	mcam12018	L0-6, R0-6	Highfield Dump	12:54:52	-4146.95

2247	mcam12027	L0356	Eshaness Coast 2x1	12:17:40	-4146.95
2255	mcam12067	L0-6, R0-6	Woodhill	12:03:55	-4144.95
2255	mcam12069	L0-6, R0-6	Newburgh	12:11:17	-4144.95
2257	mcam12084	L0-6, R0-6	Rock Hall R2x1	10:46:44	-4143.6
2259	mcam12088	L0-6, R0-6	Gometra	11:22:22	-4143.8
2262	mcam12094	L0-6, R0-6	Rock Hall Tailings	11:56:07	-4143.8
2289	mcam12199	L0-6, R0-6	Rock Hall Dump Pile	12:02:22	-4143.8
2296	mcam12237	L0-6, R0-6	Bothwell DRT	11:31:43	-4143.8
2299	mcam12266	L0-6, R0-6	Fossil Grove	11:50:14	-4141.05
2300	mcam12276	L0-6, R0-6	Linlithgow	12:17:55	-4139.85
2302	mcam12294	L0-6, R0-6	Loch_Ness DRT	11:31:20	-4140.06
2302	mcam12295	L0-6, R0-6	Loch_Skeen	11:33:25	-4140.06
2304	mcam12306	L0-6, R0-6	St_Fergus	11:43:21	-4141.97
2313	mcam12349	L0-6, R0-6	Alba Dun Ara	12:03:32	-4145.4
2316	mcam12363	L0-6, R0-6	Dauntless_to_Emerald	11:39:14	-4147.44
2319	mcam12388	L0-6, R0-6	Clyde_Gannet	12:04:59	-4148.29
2319	mcam12390	L0-6, R0-6	Curlew	12:12:42	-4148.29
2320	mcam12405	L0-6, R0-6	Curlew (redo)	12:07:36	-4148.29
2338	mcam12420	L0-6, R0-6	Cuttyhill	11:13:22	-4150.33
2338	mcam12421	L0-6, R0-6	Leslie	11:17:06	-4150.33
2347	mcam12448	L0-6, R0-6	Arbuthnott Fife	12:13:03	-4150.62
2349	mcam12464	L0-6, R0-6	Caledonia	12:09:31	-4150.62
2351	mcam12472	L0-6, R0-6	Muir of Ord	11:36:30	-4150.45
