# Supplementary material for: Synergistic Ground and Orbital Observations of Iron Oxides on Mt. Sharp and Vera Rubin Ridge
Source: J Geophys Res Planets. 2020 Sep 23;125(9):e2019JE006294. doi: 10.1029/2019JE006294 (PMC7539960; doi:10.1029/2019JE006294)
Supplement: Supplementary file 3 — Table S2 [file JGRE-125-e2019JE006294-s003.pdf]

| File Name | Target Name | Shot # | Sol | Elevation |
|---|---|---|---|---|
| CL9_464777775PSV_F0421020CCAM01758P1 | Mammoth | 1 | 758 | -4460.62 |
| CL9_464777850PSV_F0421020CCAM01758P1 | Mammoth | 2 | 758 | -4460.62 |
| CL9_464777916PSV_F0421020CCAM01758P1 | Mammoth | 3 | 758 | -4460.62 |
| CL9_464778452PSV_F0421020CCAM01758P1 | Mammoth | 7 | 758 | -4460.62 |
| CL9_464779132PSV_F0421020CCAM02758P1 | Morrison | 3 | 758 | -4460.62 |
| CL9_465587409PSV_F0421020CCAM02767P1 | The_Maze | 1 | 767 | -4460.62 |
| CL9_465587474PSV_F0421020CCAM02767P1 | The_Maze | 2 | 767 | -4460.62 |
| CL9_465587541PSV_F0421020CCAM02767P1 | The_Maze | 3 | 767 | -4460.62 |
| CL9_465587641PSV_F0421020CCAM02767P1 | The_Maze | 4 | 767 | -4460.62 |
| CL9_465587707PSV_F0421020CCAM02767P1 | The_Maze | 5 | 767 | -4460.62 |
| CL9_465587774PSV_F0421020CCAM02767P1 | The_Maze | 6 | 767 | -4460.62 |
| CL9_465587874PSV_F0421020CCAM02767P1 | The_Maze | 7 | 767 | -4460.62 |
| CL9_465587940PSV_F0421020CCAM02767P1 | The_Maze | 8 | 767 | -4460.62 |
| CL9_465588006PSV_F0421020CCAM02767P1 | The_Maze | 9 | 767 | -4460.62 |
| CL9_465939794PSV_F0421020CCAM03771P1 | White_Cliffs | 1 | 771 | -4460.62 |
| CL9_465940060PSV_F0421020CCAM03771P1 | White_Cliffs | 5 | 771 | -4460.62 |
| CL9_465940409PSV_F0421020CCAM04771P1 | Crowley | 1 | 771 | -4460.62 |
| CL9_465940474PSV_F0421020CCAM04771P1 | Crowley | 2 | 771 | -4460.62 |
| CL9_465940539PSV_F0421020CCAM04771P1 | Crowley | 3 | 771 | -4460.62 |
| CL9_465940677PSV_F0421020CCAM04771P1 | Crowley | 4 | 771 | -4460.62 |
| CL9_465940743PSV_F0421020CCAM04771P1 | Crowley | 5 | 771 | -4460.62 |
| CL9_465940808PSV_F0421020CCAM04771P1 | Crowley | 6 | 771 | -4460.62 |
| CL9_465940946PSV_F0421020CCAM04771P1 | Crowley | 7 | 771 | -4460.62 |
| CL9_465941011PSV_F0421020CCAM04771P1 | Crowley | 8 | 771 | -4460.62 |
| CL9_465941077PSV_F0421020CCAM04771P1 | Crowley | 9 | 771 | -4460.62 |
| CL9_465941142PSV_F0421020CCAM04771P1 | Crowley | 10 | 771 | -4460.62 |
| CL9_465941564PSV_F0421020CCAM05771P1 | Fairyland_Point | 2 | 771 | -4460.62 |
| CL9_465941628PSV_F0421020CCAM05771P1 | Fairyland_Point | 3 | 771 | -4460.62 |
| CL9_465941692PSV_F0421020CCAM05771P1 | Fairyland_Point | 4 | 771 | -4460.62 |
| CL9_465941756PSV_F0421020CCAM05771P1 | Fairyland_Point | 5 | 771 | -4460.62 |
| CL9_466110820PSV_F0421020CCAM03773P1 | Cockscomb | 1 | 773 | -4460.62 |
| CL9_466110977PSV_F0421020CCAM03773P1 | Cockscomb | 2 | 773 | -4460.62 |
| CL0_466375899PSV_F0421020CCAM01775P1 | Maturango_Passive | 1 | 776 | -4460.62 |
| CL0_466375962PSV_F0421020CCAM01775P1 | Maturango_Passive | 2 | 776 | -4460.62 |
| CL0_466376016PSV_F0421020CCAM01775P1 | Maturango_Passive | 3 | 776 | -4460.62 |
| CL0_466376069PSV_F0421020CCAM01775P1 | Maturango_Passive | 4 | 776 | -4460.62 |
| CL0_466376122PSV_F0421020CCAM01775P1 | Maturango_Passive | 5 | 776 | -4460.62 |
| CL9_466644364PSV_F0430000CCAM03779P1 | Hanaupah | 1 | 779 | -4460.29 |
| CL9_466644439PSV_F0430000CCAM03779P1 | Hanaupah | 2 | 779 | -4460.29 |
| CL9_466644505PSV_F0430000CCAM03779P1 | Hanaupah | 3 | 779 | -4460.29 |
| CL9_466644678PSV_F0430000CCAM03779P1 | Hanaupah | 4 | 779 | -4460.29 |
| CL9_466644744PSV_F0430000CCAM03779P1 | Hanaupah | 5 | 779 | -4460.29 |
| CL9_466644810PSV_F0430000CCAM03779P1 | Hanaupah | 6 | 779 | -4460.29 |
| CL9_466644986PSV_F0430000CCAM03779P1 | Hanaupah | 7 | 779 | -4460.29 |
| CL9_466645056PSV_F0430000CCAM03779P1 | Hanaupah | 8 | 779 | -4460.29 |
| CL9_466645122PSV_F0430000CCAM03779P1 | Hanaupah | 9 | 779 | -4460.29 |
| CL9_466821313PSV_F0430216CCAM02781P1 | Delta | 1 | 781 | -4458.98 |
| CL9_466821388PSV_F0430216CCAM02781P1 | Delta | 2 | 781 | -4458.98 |
| CL9_466821453PSV_F0430216CCAM02781P1 | Delta | 3 | 781 | -4458.98 |
| CL9_466821518PSV_F0430216CCAM02781P1 | Delta | 4 | 781 | -4458.98 |
| CL9_466821739PSV_F0430216CCAM02781P1 | Delta | 5 | 781 | -4458.98 |
| CL9_466821814PSV_F0430216CCAM02781P1 | Delta | 6 | 781 | -4458.98 |
| CL9_466821879PSV_F0430216CCAM02781P1 | Delta | 7 | 781 | -4458.98 |
| CL9_466822011PSV_F0430216CCAM02781P1 | Delta | 9 | 781 | -4458.98 |
| CL9_466822077PSV_F0430216CCAM02781P1 | Delta | 10 | 781 | -4458.98 |
| CL9_466822497PSV_F0430216CCAM03781P1 | San_Rafael_Swell | 1 | 781 | -4458.98 |
| CL9_466822572PSV_F0430216CCAM03781P1 | San_Rafael_Swell | 2 | 781 | -4458.98 |
| CL9_466822642PSV_F0430216CCAM03781P1 | San_Rafael_Swell | 3 | 781 | -4458.98 |

| | | | | |
|---|---|---|---|---|
| CL9_466822708PSV_F0430216CCAM03781P1 | San_Rafael_Swell | 4 | 781 | -4458.98 |
| CL9_466822967PSV_F0430216CCAM03781P1 | San_Rafael_Swell | 6 | 781 | -4458.98 |
| CL9_466823033PSV_F0430216CCAM03781P1 | San_Rafael_Swell | 7 | 781 | -4458.98 |
| CL9_466823099PSV_F0430216CCAM03781P1 | San_Rafael_Swell | 8 | 781 | -4458.98 |
| CL9_466823165PSV_F0430216CCAM03781P1 | San_Rafael_Swell | 9 | 781 | -4458.98 |
| CL9_466823231PSV_F0430216CCAM03781P1 | San_Rafael_Swell | 10 | 781 | -4458.98 |
| CL9_466823578PSV_F0430216CCAM04781P1 | Castle_Valley | 1 | 781 | -4458.98 |
| CL9_466823647PSV_F0430216CCAM04781P1 | Castle_Valley | 2 | 781 | -4458.98 |
| CL9_466823904PSV_F0430216CCAM04781P1 | Castle_Valley | 4 | 781 | -4458.98 |
| CL9_466823970PSV_F0430216CCAM04781P1 | Castle_Valley | 5 | 781 | -4458.98 |
| CL9_466824036PSV_F0430216CCAM04781P1 | Castle_Valley | 6 | 781 | -4458.98 |
| CL9_466824227PSV_F0430216CCAM04781P1 | Castle_Valley | 7 | 781 | -4458.98 |
| CL9_466824293PSV_F0430216CCAM04781P1 | Castle_Valley | 8 | 781 | -4458.98 |
| CL9_466824358PSV_F0430216CCAM04781P1 | Castle_Valley | 9 | 781 | -4458.98 |
| CL9_466824425PSV_F0430216CCAM04781P1 | Castle_Valley | 10 | 781 | -4458.98 |
| CL9_467178367PSV_F0440000CCAM02785P1 | Ibex_Pass | 8 | 785 | -4458.98 |
| CL9_467178433PSV_F0440000CCAM02785P1 | Ibex_Pass | 9 | 785 | -4458.98 |
| CL9_467178499PSV_F0440000CCAM02785P1 | Ibex_Pass | 10 | 785 | -4458.98 |
| CL9_467178850PSV_F0440000CCAM03785P1 | Hayden_Peak | 1 | 785 | -4458.98 |
| CL9_467178918PSV_F0440000CCAM03785P1 | Hayden_Peak | 2 | 785 | -4458.98 |
| CL9_467178984PSV_F0440000CCAM03785P1 | Hayden_Peak | 3 | 785 | -4458.98 |
| CL9_467179157PSV_F0440000CCAM03785P1 | Hayden_Peak | 4 | 785 | -4458.98 |
| CL9_467179223PSV_F0440000CCAM03785P1 | Hayden_Peak | 5 | 785 | -4458.98 |
| CL9_467179289PSV_F0440000CCAM03785P1 | Hayden_Peak | 6 | 785 | -4458.98 |
| CL9_467179462PSV_F0440000CCAM03785P1 | Hayden_Peak | 7 | 785 | -4458.98 |
| CL9_467179532PSV_F0440000CCAM03785P1 | Hayden_Peak | 8 | 785 | -4458.98 |
| CL9_467179598PSV_F0440000CCAM03785P1 | Hayden_Peak | 9 | 785 | -4458.98 |
| CL9_467179960PSV_F0440000CCAM04785P1 | Saddle_Peak | 1 | 785 | -4458.98 |
| CL9_467180028PSV_F0440000CCAM04785P1 | Saddle_Peak | 2 | 785 | -4458.98 |
| CL9_467180094PSV_F0440000CCAM04785P1 | Saddle_Peak | 3 | 785 | -4458.98 |
| CL9_467180285PSV_F0440000CCAM04785P1 | Saddle_Peak | 4 | 785 | -4458.98 |
| CL9_467352563PSV_F0440036CCAM01787P1 | Goblin_Valley | 1 | 787 | -4458.75 |
| CL9_467353520PSV_F0440036CCAM01787P1 | Goblin_Valley | 10 | 787 | -4458.75 |
| CL9_467354684PSV_F0440036CCAM02787P1 | Deadman_Pass | 8 | 787 | -4458.75 |
| CL9_467355315PSV_F0440036CCAM03787P1 | Funeral_Peak | 1 | 787 | -4458.75 |
| CL9_467355390PSV_F0440036CCAM03787P1 | Funeral_Peak | 2 | 787 | -4458.75 |
| CL9_467355456PSV_F0440036CCAM03787P1 | Funeral_Peak | 3 | 787 | -4458.75 |
| CL9_467355729PSV_F0440036CCAM03787P1 | Funeral_Peak | 5 | 787 | -4458.75 |
| CL9_467355804PSV_F0440036CCAM03787P1 | Funeral_Peak | 6 | 787 | -4458.75 |
| CL9_467355870PSV_F0440036CCAM03787P1 | Funeral_Peak | 7 | 787 | -4458.75 |
| CL9_467355936PSV_F0440036CCAM03787P1 | Funeral_Peak | 8 | 787 | -4458.75 |
| CL9_467356006PSV_F0440036CCAM03787P1 | Funeral_Peak | 9 | 787 | -4458.75 |
| CL9_467356072PSV_F0440036CCAM03787P1 | Funeral_Peak | 10 | 787 | -4458.75 |
| CL9_467530589PSV_F0440190CCAM01789P1 | Skyline | 3 | 789 | -4457.81 |
| CL9_467530948PSV_F0440190CCAM01789P1 | Skyline | 7 | 789 | -4457.81 |
| CL9_467531179PSV_F0440190CCAM01789P1 | Skyline | 9 | 789 | -4457.81 |
| CL9_467531243PSV_F0440190CCAM01789P1 | Skyline | 10 | 789 | -4457.81 |
| CL9_467531763PSV_F0440190CCAM02789P1 | Barstow | 1 | 789 | -4457.81 |
| CL9_467531838PSV_F0440190CCAM02789P1 | Barstow | 2 | 789 | -4457.81 |
| CL9_467531904PSV_F0440190CCAM02789P1 | Barstow | 3 | 789 | -4457.81 |
| CL9_467531970PSV_F0440190CCAM02789P1 | Barstow | 4 | 789 | -4457.81 |
| CL9_467532036PSV_F0440190CCAM02789P1 | Barstow | 5 | 789 | -4457.81 |
| CL9_467532102PSV_F0440190CCAM02789P1 | Barstow | 6 | 789 | -4457.81 |
| CL9_467532168PSV_F0440190CCAM02789P1 | Barstow | 7 | 789 | -4457.81 |
| CL9_467532234PSV_F0440190CCAM02789P1 | Barstow | 8 | 789 | -4457.81 |
| CL9_467532300PSV_F0440190CCAM02789P1 | Barstow | 9 | 789 | -4457.81 |
| CL9_467532366PSV_F0440190CCAM02789P1 | Barstow | 10 | 789 | -4457.81 |
| CL9_467533122PSV_F0440190CCAM03789P1 | San_Gabriel | 6 | 789 | -4457.81 |
| CL9_467533188PSV_F0440190CCAM03789P1 | San_Gabriel | 7 | 789 | -4457.81 |

| | | | | |
|---|---|---|---|---|
| CL9_467533254PSV_F0440190CCAM03789P1 | San_Gabriel | 8 | 789 | -4457.81 |
| CL9_467533320PSV_F0440190CCAM03789P1 | San_Gabriel | 9 | 789 | -4457.81 |
| CL9_467533386PSV_F0440190CCAM03789P1 | San_Gabriel | 10 | 789 | -4457.81 |
| CL9_467797525PSV_F0440256CCAM02792P1 | Agate_Hill | 1 | 792 | -4457.2 |
| CL9_467797594PSV_F0440256CCAM02792P1 | Agate_Hill | 2 | 792 | -4457.2 |
| CL9_467797658PSV_F0440256CCAM02792P1 | Agate_Hill | 3 | 792 | -4457.2 |
| CL9_467797933PSV_F0440256CCAM02792P1 | Agate_Hill | 5 | 792 | -4457.2 |
| CL9_467797997PSV_F0440256CCAM02792P1 | Agate_Hill | 6 | 792 | -4457.2 |
| CL9_467799186PSV_F0440256CCAM03792P1 | Aztec_2 | 1 | 792 | -4457.2 |
| CL9_467799255PSV_F0440256CCAM03792P1 | Aztec_2 | 2 | 792 | -4457.2 |
| CL9_467799319PSV_F0440256CCAM03792P1 | Aztec_2 | 3 | 792 | -4457.2 |
| CL9_467799496PSV_F0440256CCAM03792P1 | Aztec_2 | 4 | 792 | -4457.2 |
| CL9_467799569PSV_F0440256CCAM03792P1 | Aztec_2 | 5 | 792 | -4457.2 |
| CL9_467799633PSV_F0440256CCAM03792P1 | Aztec_2 | 6 | 792 | -4457.2 |
| CL9_467799810PSV_F0440256CCAM03792P1 | Aztec_2 | 7 | 792 | -4457.2 |
| CL9_467799874PSV_F0440256CCAM03792P1 | Aztec_2 | 8 | 792 | -4457.2 |
| CL9_467800412PSV_F0440256CCAM04792P1 | Crowder | 1 | 792 | -4457.2 |
| CL9_467800481PSV_F0440256CCAM04792P1 | Crowder | 2 | 792 | -4457.2 |
| CL9_467800547PSV_F0440256CCAM04792P1 | Crowder | 3 | 792 | -4457.2 |
| CL9_467800744PSV_F0440256CCAM04792P1 | Crowder | 4 | 792 | -4457.2 |
| CL9_467970298PSV_F0440370CCAM01794P1 | Cima_ccam | 1 | 794 | -4455.46 |
| CL9_467970373PSV_F0440370CCAM01794P1 | Cima_ccam | 2 | 794 | -4455.46 |
| CL9_467970439PSV_F0440370CCAM01794P1 | Cima_ccam | 3 | 794 | -4455.46 |
| CL9_467970666PSV_F0440370CCAM01794P1 | Cima_ccam | 4 | 794 | -4455.46 |
| CL9_467970741PSV_F0440370CCAM01794P1 | Cima_ccam | 5 | 794 | -4455.46 |
| CL9_467971656PSV_F0440370CCAM02794P1 | Sespe_ccam | 1 | 794 | -4455.46 |
| CL9_467971724PSV_F0440370CCAM02794P1 | Sespe_ccam | 2 | 794 | -4455.46 |
| CL9_467971788PSV_F0440370CCAM02794P1 | Sespe_ccam | 3 | 794 | -4455.46 |
| CL9_467971852PSV_F0440370CCAM02794P1 | Sespe_ccam | 4 | 794 | -4455.46 |
| CL9_467972077PSV_F0440370CCAM02794P1 | Sespe_ccam | 5 | 794 | -4455.46 |
| CL9_467972145PSV_F0440370CCAM02794P1 | Sespe_ccam | 6 | 794 | -4455.46 |
| CL9_467972273PSV_F0440370CCAM02794P1 | Sespe_ccam | 8 | 794 | -4455.46 |
| CL9_467972337PSV_F0440370CCAM02794P1 | Sespe_ccam | 9 | 794 | -4455.46 |
| CL9_467972401PSV_F0440370CCAM02794P1 | Sespe_ccam | 10 | 794 | -4455.46 |
| CL9_467973129PSV_F0440370CCAM03794P1 | Aguereeberry_Point | 1 | 794 | -4455.46 |
| CL9_467973198PSV_F0440370CCAM03794P1 | Aguereeberry_Point | 2 | 794 | -4455.46 |
| CL9_467973262PSV_F0440370CCAM03794P1 | Aguereeberry_Point | 3 | 794 | -4455.46 |
| CL9_467973326PSV_F0440370CCAM03794P1 | Aguereeberry_Point | 4 | 794 | -4455.46 |
| CL9_467973390PSV_F0440370CCAM03794P1 | Aguereeberry_Point | 5 | 794 | -4455.46 |
| CL9_467973811PSV_F0440370CCAM04794P1 | Soledad_Pass | 1 | 794 | -4455.46 |
| CL9_467973885PSV_F0440370CCAM04794P1 | Soledad_Pass | 2 | 794 | -4455.46 |
| CL9_467974013PSV_F0440370CCAM04794P1 | Soledad_Pass | 4 | 794 | -4455.46 |
| CL9_467974232PSV_F0440370CCAM04794P1 | Soledad_Pass | 5 | 794 | -4455.46 |
| CL9_467974300PSV_F0440370CCAM04794P1 | Soledad_Pass | 6 | 794 | -4455.46 |
| CL9_467974428PSV_F0440370CCAM04794P1 | Soledad_Pass | 8 | 794 | -4455.46 |
| CL9_467974556PSV_F0440370CCAM04794P1 | Soledad_Pass | 10 | 794 | -4455.46 |
| CL9_468153552PSV_F0440568CCAM01796P1 | Orocopia | 10 | 796 | -4453.85 |
| CL9_468154003PSV_F0440568CCAM02796P1 | Wild_Horse_Mesa | 1 | 796 | -4453.85 |
| CL9_468154087PSV_F0440568CCAM02796P1 | Wild_Horse_Mesa | 2 | 796 | -4453.85 |
| CL9_468154161PSV_F0440568CCAM02796P1 | Wild_Horse_Mesa | 3 | 796 | -4453.85 |
| CL9_468154386PSV_F0440568CCAM02796P1 | Wild_Horse_Mesa | 4 | 796 | -4453.85 |
| CL9_468154468PSV_F0440568CCAM02796P1 | Wild_Horse_Mesa | 5 | 796 | -4453.85 |
| CL9_468154541PSV_F0440568CCAM02796P1 | Wild_Horse_Mesa | 6 | 796 | -4453.85 |
| CL9_468154846PSV_F0440568CCAM02796P1 | Wild_Horse_Mesa | 8 | 796 | -4453.85 |
| CL9_468154919PSV_F0440568CCAM02796P1 | Wild_Horse_Mesa | 9 | 796 | -4453.85 |
| CL9_468154992PSV_F0440568CCAM02796P1 | Wild_Horse_Mesa | 10 | 796 | -4453.85 |
| CL9_468155687PSV_F0440568CCAM03796P1 | Baldwin | 1 | 796 | -4453.85 |
| CL9_468155762PSV_F0440568CCAM03796P1 | Baldwin | 2 | 796 | -4453.85 |
| CL9_468155835PSV_F0440568CCAM03796P1 | Baldwin | 3 | 796 | -4453.85 |

| | | | | |
|---|---|---|---|---|
| CL9_468155908PSV_F0440568CCAM03796P1 | Baldwin | 4 | 796 | -4453.85 |
| CL9_468156786PSV_F0440568CCAM04796P1 | Vasquez | 1 | 796 | -4453.85 |
| CL9_468156935PSV_F0440568CCAM04796P1 | Vasquez | 3 | 796 | -4453.85 |
| CL9_468157199PSV_F0440568CCAM04796P1 | Vasquez | 5 | 796 | -4453.85 |
| CL9_468157273PSV_F0440568CCAM04796P1 | Vasquez | 6 | 796 | -4453.85 |
| CL9_468157345PSV_F0440568CCAM04796P1 | Vasquez | 7 | 796 | -4453.85 |
| CL9_468157418PSV_F0440568CCAM04796P1 | Vasquez | 8 | 796 | -4453.85 |
| CL9_468157489PSV_F0440568CCAM04796P1 | Vasquez | 9 | 796 | -4453.85 |
| CL9_468419378PSV_F0440920CCAM01799P1 | Hackberry_Spring | 1 | 799 | -4460.67 |
| CL9_468419446PSV_F0440920CCAM01799P1 | Hackberry_Spring | 2 | 799 | -4460.67 |
| CL9_468419510PSV_F0440920CCAM01799P1 | Hackberry_Spring | 3 | 799 | -4460.67 |
| CL9_468419575PSV_F0440920CCAM01799P1 | Hackberry_Spring | 4 | 799 | -4460.67 |
| CL9_468419638PSV_F0440920CCAM01799P1 | Hackberry_Spring | 5 | 799 | -4460.67 |
| CL0_469927354PSV_F0441546CCAM01816P1 | Punchbowl | 1 | 816 | -4458.56 |
| CL0_469927406PSV_F0441546CCAM01816P1 | Punchbowl | 2 | 816 | -4458.56 |
| CL0_469927459PSV_F0441546CCAM01816P1 | Punchbowl | 3 | 816 | -4458.56 |
| CL0_469927512PSV_F0441546CCAM01816P1 | Punchbowl | 4 | 816 | -4458.56 |
| CL0_469927565PSV_F0441546CCAM01816P1 | Punchbowl | 5 | 816 | -4458.56 |
| CL0_469927890PSV_F0441546CCAM02816P1 | AftonCayon | 1 | 816 | -4458.56 |
| CL0_469927940PSV_F0441546CCAM02816P1 | AftonCayon | 2 | 816 | -4458.56 |
| CL0_469927991PSV_F0441546CCAM02816P1 | AftonCayon | 3 | 816 | -4458.56 |
| CL0_469928042PSV_F0441546CCAM02816P1 | AftonCayon | 4 | 816 | -4458.56 |
| CL0_469928093PSV_F0441546CCAM02816P1 | AftonCayon | 5 | 816 | -4458.56 |
| CL0_469928432PSV_F0441546CCAM03816P1 | Topanga | 1 | 816 | -4458.56 |
| CL0_469928485PSV_F0441546CCAM03816P1 | Topanga | 2 | 816 | -4458.56 |
| CL0_469928538PSV_F0441546CCAM03816P1 | Topanga | 3 | 816 | -4458.56 |
| CL0_469928591PSV_F0441546CCAM03816P1 | Topanga | 4 | 816 | -4458.56 |
| CL0_469928644PSV_F0441546CCAM03816P1 | Topanga | 5 | 816 | -4458.56 |
| CL0_470548285PSV_F0441828CCAM01823P1 | MescalDRT | 1 | 823 | -4456.88 |
| CL0_470548335PSV_F0441828CCAM01823P1 | MescalDRT | 2 | 823 | -4456.88 |
| CL0_470548386PSV_F0441828CCAM01823P1 | MescalDRT | 3 | 823 | -4456.88 |
| CL0_470548437PSV_F0441828CCAM01823P1 | MescalDRT | 4 | 823 | -4456.88 |
| CL0_470548488PSV_F0441828CCAM01823P1 | MescalDRT | 5 | 823 | -4456.88 |
| CL0_470814253PSV_F0441828CCAM01826P1 | PuenteDRT | 1 | 826 | -4456.88 |
| CL0_470814305PSV_F0441828CCAM01826P1 | PuenteDRT | 2 | 826 | -4456.88 |
| CL0_470814358PSV_F0441828CCAM01826P1 | PuenteDRT | 3 | 826 | -4456.88 |
| CL0_470814411PSV_F0441828CCAM01826P1 | PuenteDRT | 4 | 826 | -4456.88 |
| CL0_470814464PSV_F0441828CCAM01826P1 | PuenteDRT | 5 | 826 | -4456.88 |
| CL9_471350408PSV_F0442062CCAM04830P1 | PickhandlerDRT | 1 | 830 | -4455.2 |
| CL9_471350448PSV_F0442062CCAM04830P1 | PickhandlerDRT | 2 | 830 | -4455.2 |
| CL9_471350488PSV_F0442062CCAM04830P1 | PickhandlerDRT | 3 | 830 | -4455.2 |
| CL9_471350528PSV_F0442062CCAM04830P1 | PickhandlerDRT | 4 | 830 | -4455.2 |
| CL9_471350568PSV_F0442062CCAM04830P1 | PickhandlerDRT | 5 | 830 | -4455.2 |
| CL9_471350608PSV_F0442062CCAM04830P1 | PickhandlerDRT | 6 | 830 | -4455.2 |
| CL9_471350648PSV_F0442062CCAM04830P1 | PickhandlerDRT | 7 | 830 | -4455.2 |
| CL9_471350688PSV_F0442062CCAM04830P1 | PickhandlerDRT | 8 | 830 | -4455.2 |
| CL9_471350728PSV_F0442062CCAM04830P1 | PickhandlerDRT | 9 | 830 | -4455.2 |
| CL9_471351357PSV_F0442062CCAM06830P1 | GolderGulch | 1 | 830 | -4455.2 |
| CL9_471351398PSV_F0442062CCAM06830P1 | GolderGulch | 2 | 830 | -4455.2 |
| CL9_471351439PSV_F0442062CCAM06830P1 | GolderGulch | 3 | 830 | -4455.2 |
| CL9_471351480PSV_F0442062CCAM06830P1 | GolderGulch | 4 | 830 | -4455.2 |
| CL9_471351521PSV_F0442062CCAM06830P1 | GolderGulch | 5 | 830 | -4455.2 |
| CL9_471351562PSV_F0442062CCAM06830P1 | GolderGulch | 6 | 830 | -4455.2 |
| CL9_471351603PSV_F0442062CCAM06830P1 | GolderGulch | 7 | 830 | -4455.2 |
| CL9_471351644PSV_F0442062CCAM06830P1 | GolderGulch | 8 | 830 | -4455.2 |
| CL9_471351685PSV_F0442062CCAM06830P1 | GolderGulch | 9 | 830 | -4455.2 |
| CL0_471613357PSV_F0442062CCAM01835P1 | GoldstoneDRT_passive | 1 | 835 | -4455.2 |
| CL0_471613408PSV_F0442062CCAM01835P1 | GoldstoneDRT_passive | 2 | 835 | -4455.2 |
| CL0_471613459PSV_F0442062CCAM01835P1 | GoldstoneDRT_passive | 3 | 835 | -4455.2 |

| | | | | |
|---|---|---|---|---|
| CL0_471613509PSV_F0442062CCAM01835P1 | GoldstoneDRT_passive | 4 | 835 | -4455.2 |
| CL0_471613559PSV_F0442062CCAM01835P1 | GoldstoneDRT_passive | 5 | 835 | -4455.2 |
| CL9_473215759PSV_F0442414CCAM01853P1 | SantaAna_DRT | 1 | 853 | -4452.63 |
| CL9_473215819PSV_F0442414CCAM01853P1 | SantaAna_DRT | 2 | 853 | -4452.63 |
| CL9_473215918PSV_F0442414CCAM01853P1 | SantaAna_DRT | 3 | 853 | -4452.63 |
| CL9_473215978PSV_F0442414CCAM01853P1 | SantaAna_DRT | 4 | 853 | -4452.63 |
| CL9_473216038PSV_F0442414CCAM01853P1 | SantaAna_DRT | 5 | 853 | -4452.63 |
| CL9_473216098PSV_F0442414CCAM01853P1 | SantaAna_DRT | 6 | 853 | -4452.63 |
| CL9_473216158PSV_F0442414CCAM01853P1 | SantaAna_DRT | 7 | 853 | -4452.63 |
| CL9_473216218PSV_F0442414CCAM01853P1 | SantaAna_DRT | 8 | 853 | -4452.63 |
| CL9_473216278PSV_F0442414CCAM01853P1 | SantaAna_DRT | 9 | 853 | -4452.63 |
| CL9_485462970PSV_F0480876CCAM01991P1 | Petty | 1 | 991 | -4453.56 |
| CL9_485463046PSV_F0480876CCAM01991P1 | Petty | 2 | 991 | -4453.56 |
| CL9_485552080PSV_F0481146CCAM01992P1 | Elk | 1 | 992 | -4447.94 |
| CL9_485552156PSV_F0481146CCAM01992P1 | Elk | 2 | 992 | -4447.94 |
| CL9_485552222PSV_F0481146CCAM01992P1 | Elk | 3 | 992 | -4447.94 |
| CL9_485552288PSV_F0481146CCAM01992P1 | Elk | 4 | 992 | -4447.94 |
| CL9_485552493PSV_F0481146CCAM01992P1 | Elk | 5 | 992 | -4447.94 |
| CL9_485552819PSV_F0481146CCAM02992P1 | Bull | 1 | 992 | -4447.94 |
| CL9_485552894PSV_F0481146CCAM02992P1 | Bull | 2 | 992 | -4447.94 |
| CL9_485552960PSV_F0481146CCAM02992P1 | Bull | 3 | 992 | -4447.94 |
| CL9_485553026PSV_F0481146CCAM02992P1 | Bull | 4 | 992 | -4447.94 |
| CL9_485553222PSV_F0481146CCAM02992P1 | Bull | 5 | 992 | -4447.94 |
| CL9_485732372PSV_F0481194CCAM03993P1 | Pinehaven | 2 | 994 | -4446.98 |
| CL9_485732734PSV_F0481194CCAM03993P1 | Pinehaven | 5 | 994 | -4446.98 |
| CL9_485732803PSV_F0481194CCAM03993P1 | Pinehaven | 6 | 994 | -4446.98 |
| CL9_485732867PSV_F0481194CCAM03993P1 | Pinehaven | 7 | 994 | -4446.98 |
| CL9_485733101PSV_F0481194CCAM03993P1 | Pinehaven | 8 | 994 | -4446.98 |
| CL9_485733169PSV_F0481194CCAM03993P1 | Pinehaven | 9 | 994 | -4446.98 |
| CL9_485996178PSV_F0481530CCAM01997P1 | Mission | 1 | 997 | -4447.1 |
| CL9_485996253PSV_F0481530CCAM01997P1 | Mission | 2 | 997 | -4447.1 |
| CL9_485996319PSV_F0481530CCAM01997P1 | Mission | 3 | 997 | -4447.1 |
| CL9_485996546PSV_F0481530CCAM01997P1 | Mission | 4 | 997 | -4447.1 |
| CL9_485996614PSV_F0481530CCAM01997P1 | Mission | 5 | 997 | -4447.1 |
| CL9_485996680PSV_F0481530CCAM01997P1 | Mission | 6 | 997 | -4447.1 |
| CL9_485996908PSV_F0481530CCAM01997P1 | Mission | 7 | 997 | -4447.1 |
| CL9_485996976PSV_F0481530CCAM01997P1 | Mission | 8 | 997 | -4447.1 |
| CL9_485997180PSV_F0481530CCAM01997P1 | Mission | 9 | 997 | -4447.1 |
| CL9_485997603PSV_F0481530CCAM02997P1 | Missoula | 1 | 997 | -4447.1 |
| CL9_485997974PSV_F0481530CCAM02997P1 | Missoula | 4 | 997 | -4447.1 |
| CL9_485998042PSV_F0481530CCAM02997P1 | Missoula | 5 | 997 | -4447.1 |
| CL9_485998408PSV_F0481530CCAM02997P1 | Missoula | 8 | 997 | -4447.1 |
| CL9_485998604PSV_F0481530CCAM02997P1 | Missoula | 9 | 997 | -4447.1 |
| CL9_486087050PSV_F0481570CCAM01998P1 | Ronan_drt_2 | 1 | 998 | -4446.74 |
| CL9_486087125PSV_F0481570CCAM01998P1 | Ronan_drt_2 | 2 | 998 | -4446.74 |
| CL9_486087191PSV_F0481570CCAM01998P1 | Ronan_drt_2 | 3 | 998 | -4446.74 |
| CL9_486087420PSV_F0481570CCAM01998P1 | Ronan_drt_2 | 4 | 998 | -4446.74 |
| CL9_486087488PSV_F0481570CCAM01998P1 | Ronan_drt_2 | 5 | 998 | -4446.74 |
| CL9_486087554PSV_F0481570CCAM01998P1 | Ronan_drt_2 | 6 | 998 | -4446.74 |
| CL9_486087783PSV_F0481570CCAM01998P1 | Ronan_drt_2 | 7 | 998 | -4446.74 |
| CL9_486087851PSV_F0481570CCAM01998P1 | Ronan_drt_2 | 8 | 998 | -4446.74 |
| CL9_486088046PSV_F0481570CCAM01998P1 | Ronan_drt_2 | 9 | 998 | -4446.74 |
| CL9_486088365PSV_F0481570CCAM02998P1 | Big_Arm | 1 | 998 | -4446.74 |
| CL9_486088441PSV_F0481570CCAM02998P1 | Big_Arm | 2 | 998 | -4446.74 |
| CL9_486088507PSV_F0481570CCAM02998P1 | Big_Arm | 3 | 998 | -4446.74 |
| CL9_486088735PSV_F0481570CCAM02998P1 | Big_Arm | 4 | 998 | -4446.74 |
| CL9_486088804PSV_F0481570CCAM02998P1 | Big_Arm | 5 | 998 | -4446.74 |
| CL9_486088870PSV_F0481570CCAM02998P1 | Big_Arm | 6 | 998 | -4446.74 |
| CL9_486089098PSV_F0481570CCAM02998P1 | Big_Arm | 7 | 998 | -4446.74 |

| | | | | |
|---|---|---|---|---|
| CL9_486089167PSV_F0481570CCAM02998P1 | Big_Arm | 8 | 998 | -4446.74 |
| CL9_486089363PSV_F0481570CCAM02998P1 | Big_Arm | 9 | 998 | -4446.74 |
| CL9_486263266PSV_F0481570CCAM02000P1 | Newland | 1 | 1000 | -4446.74 |
| CL9_486263341PSV_F0481570CCAM02000P1 | Newland | 2 | 1000 | -4446.74 |
| CL9_486263408PSV_F0481570CCAM02000P1 | Newland | 3 | 1000 | -4446.74 |
| CL9_486263639PSV_F0481570CCAM02000P1 | Newland | 4 | 1000 | -4446.74 |
| CL9_486263707PSV_F0481570CCAM02000P1 | Newland | 5 | 1000 | -4446.74 |
| CL9_486263773PSV_F0481570CCAM02000P1 | Newland | 6 | 1000 | -4446.74 |
| CL9_486263980PSV_F0481570CCAM02000P1 | Newland | 7 | 1000 | -4446.74 |
| CL9_486264046PSV_F0481570CCAM02000P1 | Newland | 8 | 1000 | -4446.74 |
| CL9_486264241PSV_F0481570CCAM02000P1 | Newland | 9 | 1000 | -4446.74 |
| CL9_486351151PSV_F0481570CCAM03000P1 | Big_Arm_2 | 1 | 1001 | -4446.74 |
| CL9_486351227PSV_F0481570CCAM03000P1 | Big_Arm_2 | 2 | 1001 | -4446.74 |
| CL9_486351293PSV_F0481570CCAM03000P1 | Big_Arm_2 | 3 | 1001 | -4446.74 |
| CL9_486351520PSV_F0481570CCAM03000P1 | Big_Arm_2 | 4 | 1001 | -4446.74 |
| CL9_486351588PSV_F0481570CCAM03000P1 | Big_Arm_2 | 5 | 1001 | -4446.74 |
| CL9_486351654PSV_F0481570CCAM03000P1 | Big_Arm_2 | 6 | 1001 | -4446.74 |
| CL9_486351882PSV_F0481570CCAM03000P1 | Big_Arm_2 | 7 | 1001 | -4446.74 |
| CL9_486351950PSV_F0481570CCAM03000P1 | Big_Arm_2 | 8 | 1001 | -4446.74 |
| CL9_486352146PSV_F0481570CCAM03000P1 | Big_Arm_2 | 9 | 1001 | -4446.74 |
| CL9_488664275PSV_F0481570CCAM01027P1 | Piegan | 1 | 1027 | -4446.74 |
| CL9_488664351PSV_F0481570CCAM01027P1 | Piegan | 2 | 1027 | -4446.74 |
| CL9_488664417PSV_F0481570CCAM01027P1 | Piegan | 3 | 1027 | -4446.74 |
| CL9_488664661PSV_F0481570CCAM01027P1 | Piegan | 4 | 1027 | -4446.74 |
| CL9_488664737PSV_F0481570CCAM01027P1 | Piegan | 5 | 1027 | -4446.74 |
| CL9_488664803PSV_F0481570CCAM01027P1 | Piegan | 6 | 1027 | -4446.74 |
| CL9_488665047PSV_F0481570CCAM01027P1 | Piegan | 7 | 1027 | -4446.74 |
| CL9_488665123PSV_F0481570CCAM01027P1 | Piegan | 8 | 1027 | -4446.74 |
| CL9_488665393PSV_F0481570CCAM01027P1 | Piegan | 10 | 1027 | -4446.74 |
| CL9_488665731PSV_F0481570CCAM02027P1 | Wallace_ccam | 1 | 1027 | -4446.74 |
| CL9_488665807PSV_F0481570CCAM02027P1 | Wallace_ccam | 2 | 1027 | -4446.74 |
| CL9_488665873PSV_F0481570CCAM02027P1 | Wallace_ccam | 3 | 1027 | -4446.74 |
| CL9_488666100PSV_F0481570CCAM02027P1 | Wallace_ccam | 4 | 1027 | -4446.74 |
| CL9_488666169PSV_F0481570CCAM02027P1 | Wallace_ccam | 5 | 1027 | -4446.74 |
| CL9_488666235PSV_F0481570CCAM02027P1 | Wallace_ccam | 6 | 1027 | -4446.74 |
| CL9_488666468PSV_F0481570CCAM02027P1 | Wallace_ccam | 7 | 1027 | -4446.74 |
| CL9_488666537PSV_F0481570CCAM02027P1 | Wallace_ccam | 8 | 1027 | -4446.74 |
| CL9_488666734PSV_F0481570CCAM02027P1 | Wallace_ccam | 9 | 1027 | -4446.74 |
| CL9_488925953PSV_F0481570CCAM01030P1 | Lemhi | 2 | 1030 | -4446.74 |
| CL9_488926019PSV_F0481570CCAM01030P1 | Lemhi | 3 | 1030 | -4446.74 |
| CL9_488926249PSV_F0481570CCAM01030P1 | Lemhi | 4 | 1030 | -4446.74 |
| CL9_488926317PSV_F0481570CCAM01030P1 | Lemhi | 5 | 1030 | -4446.74 |
| CL9_488926383PSV_F0481570CCAM01030P1 | Lemhi | 6 | 1030 | -4446.74 |
| CL9_488926613PSV_F0481570CCAM01030P1 | Lemhi | 7 | 1030 | -4446.74 |
| CL9_488926681PSV_F0481570CCAM01030P1 | Lemhi | 8 | 1030 | -4446.74 |
| CL9_488926889PSV_F0481570CCAM01030P1 | Lemhi | 9 | 1030 | -4446.74 |
| CL9_488927266PSV_F0481570CCAM02030P1 | Lowary | 1 | 1030 | -4446.74 |
| CL9_488927342PSV_F0481570CCAM02030P1 | Lowary | 2 | 1030 | -4446.74 |
| CL9_488927408PSV_F0481570CCAM02030P1 | Lowary | 3 | 1030 | -4446.74 |
| CL9_488927643PSV_F0481570CCAM02030P1 | Lowary | 4 | 1030 | -4446.74 |
| CL9_488927719PSV_F0481570CCAM02030P1 | Lowary | 5 | 1030 | -4446.74 |
| CL9_488927785PSV_F0481570CCAM02030P1 | Lowary | 6 | 1030 | -4446.74 |
| CL9_488928021PSV_F0481570CCAM02030P1 | Lowary | 7 | 1030 | -4446.74 |
| CL9_488928097PSV_F0481570CCAM02030P1 | Lowary | 8 | 1030 | -4446.74 |
| CL9_488928302PSV_F0481570CCAM02030P1 | Lowary | 9 | 1030 | -4446.74 |
| CL9_489028481PSV_F0481600CCAM01031P1 | Selow | 1 | 1031 | -4446.94 |
| CL9_489028557PSV_F0481600CCAM01031P1 | Selow | 2 | 1031 | -4446.94 |
| CL9_489030832PSV_F0481600CCAM04031P1 | Seeley | 1 | 1031 | -4446.94 |
| CL9_489030907PSV_F0481600CCAM04031P1 | Seeley | 2 | 1031 | -4446.94 |

| | | | | |
|---|---|---|---|---|
| CL9_489030973PSV_F0481600CCAM04031P1 | Seeley | 3 | 1031 | -4446.94 |
| CL9_489031193PSV_F0481600CCAM04031P1 | Seeley | 4 | 1031 | -4446.94 |
| CL9_489031259PSV_F0481600CCAM04031P1 | Seeley | 5 | 1031 | -4446.94 |
| CL9_489031325PSV_F0481600CCAM04031P1 | Seeley | 6 | 1031 | -4446.94 |
| CL9_489031616PSV_F0481600CCAM04031P1 | Seeley | 8 | 1031 | -4446.94 |
| CL9_489122370PSV_F0481600CCAM01032P1 | Coombs | 1 | 1032 | -4446.94 |
| CL9_489122446PSV_F0481600CCAM01032P1 | Coombs | 2 | 1032 | -4446.94 |
| CL9_489122787PSV_F0481600CCAM01032P1 | Coombs | 5 | 1032 | -4446.94 |
| CL9_489123221PSV_F0481600CCAM02032P1 | Regis | 1 | 1032 | -4446.94 |
| CL9_489123297PSV_F0481600CCAM02032P1 | Regis | 2 | 1032 | -4446.94 |
| CL9_489123363PSV_F0481600CCAM02032P1 | Regis | 3 | 1032 | -4446.94 |
| CL9_489123429PSV_F0481600CCAM02032P1 | Regis | 4 | 1032 | -4446.94 |
| CL9_489123627PSV_F0481600CCAM02032P1 | Regis | 5 | 1032 | -4446.94 |
| CL9_489123969PSV_F0481600CCAM03032P1 | Spotted_Bear | 1 | 1032 | -4446.94 |
| CL9_489124038PSV_F0481600CCAM03032P1 | Spotted_Bear | 2 | 1032 | -4446.94 |
| CL9_489124102PSV_F0481600CCAM03032P1 | Spotted_Bear | 3 | 1032 | -4446.94 |
| CL9_489124166PSV_F0481600CCAM03032P1 | Spotted_Bear | 4 | 1032 | -4446.94 |
| CL9_489124325PSV_F0481600CCAM03032P1 | Spotted_Bear | 5 | 1032 | -4446.94 |
| CL9_489547474PSV_F0481906CCAM02037P1 | Polson | 1 | 1037 | -4447.04 |
| CL9_489547550PSV_F0481906CCAM02037P1 | Polson | 2 | 1037 | -4447.04 |
| CL9_489547616PSV_F0481906CCAM02037P1 | Polson | 3 | 1037 | -4447.04 |
| CL9_489547681PSV_F0481906CCAM02037P1 | Polson | 4 | 1037 | -4447.04 |
| CL9_489547877PSV_F0481906CCAM02037P1 | Polson | 5 | 1037 | -4447.04 |
| CL9_489548195PSV_F0481906CCAM03037P1 | Bonner | 1 | 1037 | -4447.04 |
| CL9_489548270PSV_F0481906CCAM03037P1 | Bonner | 2 | 1037 | -4447.04 |
| CL9_489548336PSV_F0481906CCAM03037P1 | Bonner | 3 | 1037 | -4447.04 |
| CL9_489548402PSV_F0481906CCAM03037P1 | Bonner | 4 | 1037 | -4447.04 |
| CL9_489548597PSV_F0481906CCAM03037P1 | Bonner | 5 | 1037 | -4447.04 |
| CL9_489725153PSV_F0481964CCAM01039P1 | Pistol | 1 | 1039 | -4447.51 |
| CL9_489725221PSV_F0481964CCAM01039P1 | Pistol | 2 | 1039 | -4447.51 |
| CL9_489725427PSV_F0481964CCAM01039P1 | Pistol | 3 | 1039 | -4447.51 |
| CL9_489725495PSV_F0481964CCAM01039P1 | Pistol | 4 | 1039 | -4447.51 |
| CL9_489725559PSV_F0481964CCAM01039P1 | Pistol | 5 | 1039 | -4447.51 |
| CL9_489727216PSV_F0481964CCAM03039P1 | Shepard | 1 | 1039 | -4447.51 |
| CL9_489727284PSV_F0481964CCAM03039P1 | Shepard | 2 | 1039 | -4447.51 |
| CL9_489727348PSV_F0481964CCAM03039P1 | Shepard | 3 | 1039 | -4447.51 |
| CL9_489727588PSV_F0481964CCAM03039P1 | Shepard | 4 | 1039 | -4447.51 |
| CL9_489727657PSV_F0481964CCAM03039P1 | Shepard | 5 | 1039 | -4447.51 |
| CL9_489727721PSV_F0481964CCAM03039P1 | Shepard | 6 | 1039 | -4447.51 |
| CL9_489727961PSV_F0481964CCAM03039P1 | Shepard | 7 | 1039 | -4447.51 |
| CL9_489728030PSV_F0481964CCAM03039P1 | Shepard | 8 | 1039 | -4447.51 |
| CL9_489728232PSV_F0481964CCAM03039P1 | Shepard | 9 | 1039 | -4447.51 |
| CL9_489728548PSV_F0481964CCAM04039P1 | Dublin_Gulch | 1 | 1039 | -4447.51 |
| CL9_489728622PSV_F0481964CCAM04039P1 | Dublin_Gulch | 2 | 1039 | -4447.51 |
| CL9_489728686PSV_F0481964CCAM04039P1 | Dublin_Gulch | 3 | 1039 | -4447.51 |
| CL9_489728921PSV_F0481964CCAM04039P1 | Dublin_Gulch | 4 | 1039 | -4447.51 |
| CL9_489728989PSV_F0481964CCAM04039P1 | Dublin_Gulch | 5 | 1039 | -4447.51 |
| CL9_489729053PSV_F0481964CCAM04039P1 | Dublin_Gulch | 6 | 1039 | -4447.51 |
| CL9_489903521PSV_F0481970CCAM03041P1 | Frog | 1 | 1041 | -4447.51 |
| CL9_489903589PSV_F0481970CCAM03041P1 | Frog | 2 | 1041 | -4447.51 |
| CL9_489903653PSV_F0481970CCAM03041P1 | Frog | 3 | 1041 | -4447.51 |
| CL9_489903893PSV_F0481970CCAM03041P1 | Frog | 4 | 1041 | -4447.51 |
| CL9_489903958PSV_F0481970CCAM03041P1 | Frog | 5 | 1041 | -4447.51 |
| CL9_489904022PSV_F0481970CCAM03041P1 | Frog | 6 | 1041 | -4447.51 |
| CL9_489904262PSV_F0481970CCAM03041P1 | Frog | 7 | 1041 | -4447.51 |
| CL9_489904331PSV_F0481970CCAM03041P1 | Frog | 8 | 1041 | -4447.51 |
| CL9_489904542PSV_F0481970CCAM03041P1 | Frog | 9 | 1041 | -4447.51 |
| CL9_490168288PSV_F0482122CCAM01044P1 | Edith | 1 | 1044 | -4447.97 |
| CL9_490168363PSV_F0482122CCAM01044P1 | Edith | 2 | 1044 | -4447.97 |

| | | | | |
|---|---|---|---|---|
| CL9_490168429PSV_F0482122CCAM01044P1 | Edith | 3 | 1044 | -4447.97 |
| CL9_490168495PSV_F0482122CCAM01044P1 | Edith | 4 | 1044 | -4447.97 |
| CL9_490168691PSV_F0482122CCAM01044P1 | Edith | 5 | 1044 | -4447.97 |
| CL9_490169099PSV_F0482122CCAM02044P1 | Sheffer | 1 | 1044 | -4447.97 |
| CL9_490169174PSV_F0482122CCAM02044P1 | Sheffer | 2 | 1044 | -4447.97 |
| CL9_490169240PSV_F0482122CCAM02044P1 | Sheffer | 3 | 1044 | -4447.97 |
| CL9_490169306PSV_F0482122CCAM02044P1 | Sheffer | 4 | 1044 | -4447.97 |
| CL9_490169501PSV_F0482122CCAM02044P1 | Sheffer | 5 | 1044 | -4447.97 |
| CL9_490169845PSV_F0482122CCAM03044P1 | Finley | 1 | 1044 | -4447.97 |
| CL9_490169913PSV_F0482122CCAM03044P1 | Finley | 2 | 1044 | -4447.97 |
| CL9_490169977PSV_F0482122CCAM03044P1 | Finley | 3 | 1044 | -4447.97 |
| CL9_490170041PSV_F0482122CCAM03044P1 | Finley | 4 | 1044 | -4447.97 |
| CL9_490170234PSV_F0482122CCAM03044P1 | Finley | 5 | 1044 | -4447.97 |
| CL9_490345150PSV_F0482200CCAM01046P1 | Mustang | 1 | 1046 | -4447.94 |
| CL9_490345218PSV_F0482200CCAM01046P1 | Mustang | 2 | 1046 | -4447.94 |
| CL9_490345602PSV_F0482200CCAM01046P1 | Mustang | 5 | 1046 | -4447.94 |
| CL9_490345914PSV_F0482200CCAM01046P1 | Mustang | 7 | 1046 | -4447.94 |
| CL9_490345983PSV_F0482200CCAM01046P1 | Mustang | 8 | 1046 | -4447.94 |
| CL9_490346254PSV_F0482200CCAM01046P1 | Mustang | 10 | 1046 | -4447.94 |
| CL9_490527987PSV_F0482224CCAM01048P1 | Pinto | 1 | 1048 | -4448.02 |
| CL9_490528055PSV_F0482224CCAM01048P1 | Pinto | 2 | 1048 | -4448.02 |
| CL9_490528119PSV_F0482224CCAM01048P1 | Pinto | 3 | 1048 | -4448.02 |
| CL9_490791972PSV_F0482422CCAM03051P1 | Mullan | 2 | 1051 | -4447.01 |
| CL9_490792038PSV_F0482422CCAM03051P1 | Mullan | 3 | 1051 | -4447.01 |
| CL9_490792104PSV_F0482422CCAM03051P1 | Mullan | 4 | 1051 | -4447.01 |
| CL9_490792299PSV_F0482422CCAM03051P1 | Mullan | 5 | 1051 | -4447.01 |
| CL9_490966957PSV_F0482470CCAM01053P1 | Sorrel_Springs | 1 | 1053 | -4446.78 |
| CL9_490967032PSV_F0482470CCAM01053P1 | Sorrel_Springs | 2 | 1053 | -4446.78 |
| CL9_490967097PSV_F0482470CCAM01053P1 | Sorrel_Springs | 3 | 1053 | -4446.78 |
| CL9_490967337PSV_F0482470CCAM01053P1 | Sorrel_Springs | 4 | 1053 | -4446.78 |
| CL9_490967409PSV_F0482470CCAM01053P1 | Sorrel_Springs | 5 | 1053 | -4446.78 |
| CL9_490967475PSV_F0482470CCAM01053P1 | Sorrel_Springs | 6 | 1053 | -4446.78 |
| CL9_490967716PSV_F0482470CCAM01053P1 | Sorrel_Springs | 7 | 1053 | -4446.78 |
| CL9_490967791PSV_F0482470CCAM01053P1 | Sorrel_Springs | 8 | 1053 | -4446.78 |
| CL9_490967995PSV_F0482470CCAM01053P1 | Sorrel_Springs | 9 | 1053 | -4446.78 |
| CL9_490968352PSV_F0482470CCAM02053P1 | Buckskin | 1 | 1053 | -4446.78 |
| CL9_490968420PSV_F0482470CCAM02053P1 | Buckskin | 2 | 1053 | -4446.78 |
| CL9_490968484PSV_F0482470CCAM02053P1 | Buckskin | 3 | 1053 | -4446.78 |
| CL9_490968547PSV_F0482470CCAM02053P1 | Buckskin | 4 | 1053 | -4446.78 |
| CL9_490968749PSV_F0482470CCAM02053P1 | Buckskin | 5 | 1053 | -4446.78 |
| CL9_491145038PSV_F0482518CCAM01055P1 | Marent | 1 | 1055 | -4446.69 |
| CL9_491145106PSV_F0482518CCAM01055P1 | Marent | 2 | 1055 | -4446.69 |
| CL9_491145170PSV_F0482518CCAM01055P1 | Marent | 3 | 1055 | -4446.69 |
| CL9_491145234PSV_F0482518CCAM01055P1 | Marent | 4 | 1055 | -4446.69 |
| CL9_491145436PSV_F0482518CCAM01055P1 | Marent | 5 | 1055 | -4446.69 |
| CL9_491145781PSV_F0482518CCAM02055P1 | Pilcher | 1 | 1055 | -4446.69 |
| CL9_491145849PSV_F0482518CCAM02055P1 | Pilcher | 2 | 1055 | -4446.69 |
| CL9_491145913PSV_F0482518CCAM02055P1 | Pilcher | 3 | 1055 | -4446.69 |
| CL9_491146154PSV_F0482518CCAM02055P1 | Pilcher | 4 | 1055 | -4446.69 |
| CL9_491146222PSV_F0482518CCAM02055P1 | Pilcher | 5 | 1055 | -4446.69 |
| CL9_491146286PSV_F0482518CCAM02055P1 | Pilcher | 6 | 1055 | -4446.69 |
| CL9_491146529PSV_F0482518CCAM02055P1 | Pilcher | 7 | 1055 | -4446.69 |
| CL9_491146597PSV_F0482518CCAM02055P1 | Pilcher | 8 | 1055 | -4446.69 |
| CL9_491146799PSV_F0482518CCAM02055P1 | Pilcher | 9 | 1055 | -4446.69 |
| CL9_491147142PSV_F0482518CCAM03055P1 | Beckwith | 1 | 1055 | -4446.69 |
| CL9_491147210PSV_F0482518CCAM03055P1 | Beckwith | 2 | 1055 | -4446.69 |
| CL9_491147273PSV_F0482518CCAM03055P1 | Beckwith | 3 | 1055 | -4446.69 |
| CL9_491147515PSV_F0482518CCAM03055P1 | Beckwith | 4 | 1055 | -4446.69 |
| CL9_491147583PSV_F0482518CCAM03055P1 | Beckwith | 5 | 1055 | -4446.69 |

| | | | | |
|---|---|---|---|---|
| CL9_491147647PSV_F0482518CCAM03055P1 | Beckwith | 6 | 1055 | -4446.69 |
| CL9_491147889PSV_F0482518CCAM03055P1 | Beckwith | 7 | 1055 | -4446.69 |
| CL9_491147957PSV_F0482518CCAM03055P1 | Beckwith | 8 | 1055 | -4446.69 |
| CL9_491148159PSV_F0482518CCAM03055P1 | Beckwith | 9 | 1055 | -4446.69 |
| CL9_491148523PSV_F0482518CCAM04055P1 | Twinkle | 1 | 1055 | -4446.69 |
| CL9_491148597PSV_F0482518CCAM04055P1 | Twinkle | 2 | 1055 | -4446.69 |
| CL9_491148661PSV_F0482518CCAM04055P1 | Twinkle | 3 | 1055 | -4446.69 |
| CL9_491148897PSV_F0482518CCAM04055P1 | Twinkle | 4 | 1055 | -4446.69 |
| CL9_491148965PSV_F0482518CCAM04055P1 | Twinkle | 5 | 1055 | -4446.69 |
| CL9_491149029PSV_F0482518CCAM04055P1 | Twinkle | 6 | 1055 | -4446.69 |
| CL9_491149270PSV_F0482518CCAM04055P1 | Twinkle | 7 | 1055 | -4446.69 |
| CL9_491149338PSV_F0482518CCAM04055P1 | Twinkle | 8 | 1055 | -4446.69 |
| CL9_491149540PSV_F0482518CCAM04055P1 | Twinkle | 9 | 1055 | -4446.69 |
| CL9_491322436PSV_F0482542CCAM01057P1 | Blind_Gulch | 1 | 1057 | -4446.83 |
| CL9_491322511PSV_F0482542CCAM01057P1 | Blind_Gulch | 2 | 1057 | -4446.83 |
| CL9_491322577PSV_F0482542CCAM01057P1 | Blind_Gulch | 3 | 1057 | -4446.83 |
| CL9_491322783PSV_F0482542CCAM01057P1 | Blind_Gulch | 4 | 1057 | -4446.83 |
| CL9_491322848PSV_F0482542CCAM01057P1 | Blind_Gulch | 5 | 1057 | -4446.83 |
| CL9_491322914PSV_F0482542CCAM01057P1 | Blind_Gulch | 6 | 1057 | -4446.83 |
| CL9_491323120PSV_F0482542CCAM01057P1 | Blind_Gulch | 7 | 1057 | -4446.83 |
| CL9_491323186PSV_F0482542CCAM01057P1 | Blind_Gulch | 8 | 1057 | -4446.83 |
| CL9_491323358PSV_F0482542CCAM01057P1 | Blind_Gulch | 9 | 1057 | -4446.83 |
| CL9_491323725PSV_F0482542CCAM02057P1 | Sorrel_Springs_2 | 1 | 1057 | -4446.83 |
| CL9_491323800PSV_F0482542CCAM02057P1 | Sorrel_Springs_2 | 2 | 1057 | -4446.83 |
| CL9_491323866PSV_F0482542CCAM02057P1 | Sorrel_Springs_2 | 3 | 1057 | -4446.83 |
| CL9_491323932PSV_F0482542CCAM02057P1 | Sorrel_Springs_2 | 4 | 1057 | -4446.83 |
| CL9_491324104PSV_F0482542CCAM02057P1 | Sorrel_Springs_2 | 5 | 1057 | -4446.83 |
| CL9_491324450PSV_F0482542CCAM03057P1 | Buffalo_Basin | 1 | 1057 | -4446.83 |
| CL9_491324525PSV_F0482542CCAM03057P1 | Buffalo_Basin | 2 | 1057 | -4446.83 |
| CL9_491324591PSV_F0482542CCAM03057P1 | Buffalo_Basin | 3 | 1057 | -4446.83 |
| CL9_491324861PSV_F0482542CCAM03057P1 | Buffalo_Basin | 5 | 1057 | -4446.83 |
| CL9_491776926PSV_F0482542CCAM04061P1 | Diamond_Point | 1 | 1062 | -4446.83 |
| CL9_491777001PSV_F0482542CCAM04061P1 | Diamond_Point | 2 | 1062 | -4446.83 |
| CL9_491777067PSV_F0482542CCAM04061P1 | Diamond_Point | 3 | 1062 | -4446.83 |
| CL9_491777315PSV_F0482542CCAM04061P1 | Diamond_Point | 4 | 1062 | -4446.83 |
| CL9_491777390PSV_F0482542CCAM04061P1 | Diamond_Point | 5 | 1062 | -4446.83 |
| CL9_491777456PSV_F0482542CCAM04061P1 | Diamond_Point | 6 | 1062 | -4446.83 |
| CL9_491777704PSV_F0482542CCAM04061P1 | Diamond_Point | 7 | 1062 | -4446.83 |
| CL9_491777779PSV_F0482542CCAM04061P1 | Diamond_Point | 8 | 1062 | -4446.83 |
| CL9_491777845PSV_F0482542CCAM04061P1 | Diamond_Point | 9 | 1062 | -4446.83 |
| CL9_491778049PSV_F0482542CCAM04061P1 | Diamond_Point | 10 | 1062 | -4446.83 |
| CL9_491945828PSV_F0482542CCAM03064P1 | Mountain_Home | 1 | 1064 | -4446.83 |
| CL9_491945903PSV_F0482542CCAM03064P1 | Mountain_Home | 2 | 1064 | -4446.83 |
| CL9_491945969PSV_F0482542CCAM03064P1 | Mountain_Home | 3 | 1064 | -4446.83 |
| CL9_491946288PSV_F0482542CCAM03064P1 | Mountain_Home | 5 | 1064 | -4446.83 |
| CL9_491946354PSV_F0482542CCAM03064P1 | Mountain_Home | 6 | 1064 | -4446.83 |
| CL9_491946598PSV_F0482542CCAM03064P1 | Mountain_Home | 7 | 1064 | -4446.83 |
| CL9_491946673PSV_F0482542CCAM03064P1 | Mountain_Home | 8 | 1064 | -4446.83 |
| CL9_491946911PSV_F0482542CCAM03064P1 | Mountain_Home | 10 | 1064 | -4446.83 |
| CL9_491947229PSV_F0482542CCAM04064P1 | Martz | 1 | 1064 | -4446.83 |
| CL9_491947304PSV_F0482542CCAM04064P1 | Martz | 2 | 1064 | -4446.83 |
| CL9_491947370PSV_F0482542CCAM04064P1 | Martz | 3 | 1064 | -4446.83 |
| CL9_491947582PSV_F0482542CCAM04064P1 | Martz | 4 | 1064 | -4446.83 |
| CL9_491947658PSV_F0482542CCAM04064P1 | Martz | 5 | 1064 | -4446.83 |
| CL9_491947724PSV_F0482542CCAM04064P1 | Martz | 6 | 1064 | -4446.83 |
| CL9_491947936PSV_F0482542CCAM04064P1 | Martz | 7 | 1064 | -4446.83 |
| CL9_491948011PSV_F0482542CCAM04064P1 | Martz | 8 | 1064 | -4446.83 |
| CL9_491948077PSV_F0482542CCAM04064P1 | Martz | 9 | 1064 | -4446.83 |
| CL9_491948249PSV_F0482542CCAM04064P1 | Martz | 10 | 1064 | -4446.83 |

| | | | | |
|---|---|---|---|---|
| CL9_492123842PSV_F0482542CCAM01066P1 | Moiese | 1 | 1066 | -4446.83 |
| CL9_492123917PSV_F0482542CCAM01066P1 | Moiese | 2 | 1066 | -4446.83 |
| CL9_492123983PSV_F0482542CCAM01066P1 | Moiese | 3 | 1066 | -4446.83 |
| CL9_492124227PSV_F0482542CCAM01066P1 | Moiese | 4 | 1066 | -4446.83 |
| CL9_492124302PSV_F0482542CCAM01066P1 | Moiese | 5 | 1066 | -4446.83 |
| CL9_492124368PSV_F0482542CCAM01066P1 | Moiese | 6 | 1066 | -4446.83 |
| CL9_492124612PSV_F0482542CCAM01066P1 | Moiese | 7 | 1066 | -4446.83 |
| CL9_492124687PSV_F0482542CCAM01066P1 | Moiese | 8 | 1066 | -4446.83 |
| CL9_492124753PSV_F0482542CCAM01066P1 | Moiese | 9 | 1066 | -4446.83 |
| CL9_492124957PSV_F0482542CCAM01066P1 | Moiese | 10 | 1066 | -4446.83 |
| CL9_492211494PSV_F0482794CCAM02067P1 | Dixon | 1 | 1067 | -4447.79 |
| CL9_492211569PSV_F0482794CCAM02067P1 | Dixon | 2 | 1067 | -4447.79 |
| CL9_492211635PSV_F0482794CCAM02067P1 | Dixon | 3 | 1067 | -4447.79 |
| CL9_492211701PSV_F0482794CCAM02067P1 | Dixon | 4 | 1067 | -4447.79 |
| CL9_492211896PSV_F0482794CCAM02067P1 | Dixon | 5 | 1067 | -4447.79 |
| CL9_492212359PSV_F0482794CCAM03067P1 | Doney | 1 | 1067 | -4447.79 |
| CL9_492212434PSV_F0482794CCAM03067P1 | Doney | 2 | 1067 | -4447.79 |
| CL9_492212500PSV_F0482794CCAM03067P1 | Doney | 3 | 1067 | -4447.79 |
| CL9_492212764PSV_F0482794CCAM03067P1 | Doney | 4 | 1067 | -4447.79 |
| CL9_492212906PSV_F0482794CCAM03067P1 | Doney | 6 | 1067 | -4447.79 |
| CL9_492213170PSV_F0482794CCAM03067P1 | Doney | 7 | 1067 | -4447.79 |
| CL9_492213245PSV_F0482794CCAM03067P1 | Doney | 8 | 1067 | -4447.79 |
| CL9_492213310PSV_F0482794CCAM03067P1 | Doney | 9 | 1067 | -4447.79 |
| CL9_492213502PSV_F0482794CCAM03067P1 | Doney | 10 | 1067 | -4447.79 |
| CL0_492564751PSV_F0490000CCAM01071P1 | Coombs | 2 | 1071 | -4447.1 |
| CL0_492564803PSV_F0490000CCAM01071P1 | Coombs | 3 | 1071 | -4447.1 |
| CL0_492564855PSV_F0490000CCAM01071P1 | Coombs | 4 | 1071 | -4447.1 |
| CL0_492564907PSV_F0490000CCAM01071P1 | Coombs | 5 | 1071 | -4447.1 |
| CL9_500208774PSV_F0501928CCAM01157P1 | Augusta | 1 | 1157 | -4433.24 |
| CL9_500208849PSV_F0501928CCAM01157P1 | Augusta | 2 | 1157 | -4433.24 |
| CL9_500208915PSV_F0501928CCAM01157P1 | Augusta | 3 | 1157 | -4433.24 |
| CL9_500208980PSV_F0501928CCAM01157P1 | Augusta | 4 | 1157 | -4433.24 |
| CL9_500209176PSV_F0501928CCAM01157P1 | Augusta | 5 | 1157 | -4433.24 |
| CL9_500470659PSV_F0502438CCAM01160P1 | Roter_Kamm | 1 | 1160 | -4432.23 |
| CL9_500470735PSV_F0502438CCAM01160P1 | Roter_Kamm | 2 | 1160 | -4432.23 |
| CL9_500470801PSV_F0502438CCAM01160P1 | Roter_Kamm | 3 | 1160 | -4432.23 |
| CL9_500471063PSV_F0502438CCAM01160P1 | Roter_Kamm | 5 | 1160 | -4432.23 |
| CL9_500648508PSV_F0502772CCAM02162P1 | Tsumeb | 1 | 1162 | -4433.83 |
| CL9_500648583PSV_F0502772CCAM02162P1 | Tsumeb | 2 | 1162 | -4433.83 |
| CL9_500648648PSV_F0502772CCAM02162P1 | Tsumeb | 3 | 1162 | -4433.83 |
| CL9_500648713PSV_F0502772CCAM02162P1 | Tsumeb | 4 | 1162 | -4433.83 |
| CL9_500648778PSV_F0502772CCAM02162P1 | Tsumeb | 5 | 1162 | -4433.83 |
| CL9_500648843PSV_F0502772CCAM02162P1 | Tsumeb | 6 | 1162 | -4433.83 |
| CL9_500648908PSV_F0502772CCAM02162P1 | Tsumeb | 7 | 1162 | -4433.83 |
| CL9_501005014PSV_F0503076CCAM01166P1 | Zaris | 6 | 1166 | -4432.32 |
| CL9_501005078PSV_F0503076CCAM01166P1 | Zaris | 7 | 1166 | -4432.32 |
| CL9_501005793PSV_F0503076CCAM02166P1 | Swakop | 2 | 1166 | -4432.32 |
| CL9_501005859PSV_F0503076CCAM02166P1 | Swakop | 3 | 1166 | -4432.32 |
| CL9_501006121PSV_F0503076CCAM02166P1 | Swakop | 5 | 1166 | -4432.32 |
| CL9_501181718PSV_F0510000CCAM01168P1 | Etosha | 1 | 1168 | -4429.3 |
| CL9_501182032PSV_F0510000CCAM01168P1 | Etosha | 3 | 1168 | -4429.3 |
| CL9_501182108PSV_F0510000CCAM01168P1 | Etosha | 4 | 1168 | -4429.3 |
| CL9_501282112PSV_F0510268CCAM02169P1 | Dordabis | 1 | 1169 | -4427.44 |
| CL9_501282188PSV_F0510268CCAM02169P1 | Dordabis | 2 | 1169 | -4427.44 |
| CL9_501282505PSV_F0510268CCAM02169P1 | Dordabis | 4 | 1169 | -4427.44 |
| CL9_501282647PSV_F0510268CCAM02169P1 | Dordabis | 6 | 1169 | -4427.44 |
| CL9_501282898PSV_F0510268CCAM02169P1 | Dordabis | 7 | 1169 | -4427.44 |
| CL9_501282974PSV_F0510268CCAM02169P1 | Dordabis | 8 | 1169 | -4427.44 |
| CL9_501283040PSV_F0510268CCAM02169P1 | Dordabis | 9 | 1169 | -4427.44 |

| | | | | |
|---|---|---|---|---|
| CL9_501283245PSV_F0510268CCAM02169P1 | Dordabis | 10 | 1169 | -4427.44 |
| CL9_501283579PSV_F0510268CCAM03169P1 | Kuibis | 1 | 1169 | -4427.44 |
| CL9_501283655PSV_F0510268CCAM03169P1 | Kuibis | 2 | 1169 | -4427.44 |
| CL9_501283721PSV_F0510268CCAM03169P1 | Kuibis | 3 | 1169 | -4427.44 |
| CL9_501283787PSV_F0510268CCAM03169P1 | Kuibis | 4 | 1169 | -4427.44 |
| CL9_501283989PSV_F0510268CCAM03169P1 | Kuibis | 5 | 1169 | -4427.44 |
| CL9_501458660PSV_F0510268CCAM03170P1 | Grootfontein | 3 | 1171 | -4427.44 |
| CL9_501458907PSV_F0510268CCAM03170P1 | Grootfontein | 4 | 1171 | -4427.44 |
| CL9_501981290PSV_F0511102CCAM02177P1 | Hoarusib | 1 | 1177 | -4424.91 |
| CL9_501981358PSV_F0511102CCAM02177P1 | Hoarusib | 2 | 1177 | -4424.91 |
| CL9_501981422PSV_F0511102CCAM02177P1 | Hoarusib | 3 | 1177 | -4424.91 |
| CL9_501981657PSV_F0511102CCAM02177P1 | Hoarusib | 4 | 1177 | -4424.91 |
| CL9_501981725PSV_F0511102CCAM02177P1 | Hoarusib | 5 | 1177 | -4424.91 |
| CL9_501981789PSV_F0511102CCAM02177P1 | Hoarusib | 6 | 1177 | -4424.91 |
| CL9_501982024PSV_F0511102CCAM02177P1 | Hoarusib | 7 | 1177 | -4424.91 |
| CL9_501982092PSV_F0511102CCAM02177P1 | Hoarusib | 8 | 1177 | -4424.91 |
| CL9_501982156PSV_F0511102CCAM02177P1 | Hoarusib | 9 | 1177 | -4424.91 |
| CL9_501982350PSV_F0511102CCAM02177P1 | Hoarusib | 10 | 1177 | -4424.91 |
| CL9_502868268PSV_F0511800CCAM01187P1 | Rehoboth | 2 | 1187 | -4422.86 |
| CL9_502868333PSV_F0511800CCAM01187P1 | Rehoboth | 3 | 1187 | -4422.86 |
| CL9_502868399PSV_F0511800CCAM01187P1 | Rehoboth | 4 | 1187 | -4422.86 |
| CL9_502868603PSV_F0511800CCAM01187P1 | Rehoboth | 5 | 1187 | -4422.86 |
| CL9_502868808PSV_F0511800CCAM01187P1 | Rehoboth | 8 | 1187 | -4422.86 |
| CL9_502868874PSV_F0511800CCAM01187P1 | Rehoboth | 9 | 1187 | -4422.86 |
| CL9_503045627PSV_F0512004CCAM01189P1 | Opononono | 1 | 1189 | -4422.88 |
| CL9_503045702PSV_F0512004CCAM01189P1 | Oponono | 2 | 1189 | -4422.88 |
| CL9_503045767PSV_F0512004CCAM01189P1 | Oponono | 3 | 1189 | -4422.88 |
| CL9_503046004PSV_F0512004CCAM01189P1 | Oponono | 4 | 1189 | -4422.88 |
| CL9_503046079PSV_F0512004CCAM01189P1 | Oponono | 5 | 1189 | -4422.88 |
| CL9_503046477PSV_F0512004CCAM01189P1 | Oponono | 8 | 1189 | -4422.88 |
| CL9_503046543PSV_F0512004CCAM01189P1 | Oponono | 9 | 1189 | -4422.88 |
| CL9_503047232PSV_F0512004CCAM02189P1 | Khakabas | 3 | 1189 | -4422.88 |
| CL9_503047469PSV_F0512004CCAM02189P1 | Khakabas | 4 | 1189 | -4422.88 |
| CL9_503047544PSV_F0512004CCAM02189P1 | Khakabas | 5 | 1189 | -4422.88 |
| CL9_503047610PSV_F0512004CCAM02189P1 | Khakabas | 6 | 1189 | -4422.88 |
| CL9_503047847PSV_F0512004CCAM02189P1 | Khakabas | 7 | 1189 | -4422.88 |
| CL9_503047922PSV_F0512004CCAM02189P1 | Khakabas | 8 | 1189 | -4422.88 |
| CL9_503048184PSV_F0512004CCAM02189P1 | Khakabas | 10 | 1189 | -4422.88 |
| CL9_503238193PSV_F0512004CCAM01191P1 | Messum | 1 | 1191 | -4422.88 |
| CL9_503238262PSV_F0512004CCAM01191P1 | Messum | 2 | 1191 | -4422.88 |
| CL9_503238326PSV_F0512004CCAM01191P1 | Messum | 3 | 1191 | -4422.88 |
| CL9_503238670PSV_F0512004CCAM01191P1 | Messum | 5 | 1191 | -4422.88 |
| CL9_503238735PSV_F0512004CCAM01191P1 | Messum | 6 | 1191 | -4422.88 |
| CL9_503239015PSV_F0512004CCAM01191P1 | Messum | 7 | 1191 | -4422.88 |
| CL9_503239083PSV_F0512004CCAM01191P1 | Messum | 8 | 1191 | -4422.88 |
| CL9_503239147PSV_F0512004CCAM01191P1 | Messum | 9 | 1191 | -4422.88 |
| CL9_503239805PSV_F0512004CCAM02191P1 | Karoo | 1 | 1191 | -4422.88 |
| CL9_503239873PSV_F0512004CCAM02191P1 | Karoo | 2 | 1191 | -4422.88 |
| CL9_503239937PSV_F0512004CCAM02191P1 | Karoo | 3 | 1191 | -4422.88 |
| CL9_503240001PSV_F0512004CCAM02191P1 | Karoo | 4 | 1191 | -4422.88 |
| CL9_503240209PSV_F0512004CCAM02191P1 | Karoo | 5 | 1191 | -4422.88 |
| CL9_503487357PSV_F0512322CCAM01194P1 | Karfenkliff | 1 | 1194 | -4421.85 |
| CL9_503487432PSV_F0512322CCAM01194P1 | Karfenkliff | 2 | 1194 | -4421.85 |
| CL9_503487564PSV_F0512322CCAM01194P1 | Karfenkliff | 4 | 1194 | -4421.85 |
| CL9_503487760PSV_F0512322CCAM01194P1 | Karfenkliff | 5 | 1194 | -4421.85 |
| CL9_503488242PSV_F0512322CCAM02194P1 | Grillental | 3 | 1194 | -4421.85 |
| CL9_503488477PSV_F0512322CCAM02194P1 | Grillental | 4 | 1194 | -4421.85 |
| CL9_503488546PSV_F0512322CCAM02194P1 | Grillental | 5 | 1194 | -4421.85 |
| CL9_503488610PSV_F0512322CCAM02194P1 | Grillental | 6 | 1194 | -4421.85 |

| | | | | |
|---|---|---|---|---|
| CL9_503488845PSV_F0512322CCAM02194P1 | Grillental | 7 | 1194 | -4421.85 |
| CL9_504034226PSV_F0520000CCAM02200P1 | Matchless | 2 | 1200 | -4419.91 |
| CL9_504034292PSV_F0520000CCAM02200P1 | Matchless | 3 | 1200 | -4419.91 |
| CL9_504034541PSV_F0520000CCAM02200P1 | Matchless | 4 | 1200 | -4419.91 |
| CL9_504034617PSV_F0520000CCAM02200P1 | Matchless | 5 | 1200 | -4419.91 |
| CL9_504035625PSV_F0520000CCAM03200P1 | Nama | 1 | 1200 | -4419.91 |
| CL9_504035693PSV_F0520000CCAM03200P1 | Nama | 2 | 1200 | -4419.91 |
| CL9_504035757PSV_F0520000CCAM03200P1 | Nama | 3 | 1200 | -4419.91 |
| CL9_504036014PSV_F0520000CCAM03200P1 | Nama | 4 | 1200 | -4419.91 |
| CL9_504036082PSV_F0520000CCAM03200P1 | Nama | 5 | 1200 | -4419.91 |
| CL9_504297737PSV_F0520000CCAM03202P1 | Aus | 1 | 1203 | -4419.91 |
| CL9_504297805PSV_F0520000CCAM03202P1 | Aus | 2 | 1203 | -4419.91 |
| CL9_504297868PSV_F0520000CCAM03202P1 | Aus | 3 | 1203 | -4419.91 |
| CL9_504297932PSV_F0520000CCAM03202P1 | Aus | 4 | 1203 | -4419.91 |
| CL9_504297996PSV_F0520000CCAM03202P1 | Aus | 5 | 1203 | -4419.91 |
| CL9_504298059PSV_F0520000CCAM03202P1 | Aus | 6 | 1203 | -4419.91 |
| CL9_504298122PSV_F0520000CCAM03202P1 | Aus | 7 | 1203 | -4419.91 |
| CL9_504298185PSV_F0520000CCAM03202P1 | Aus | 8 | 1203 | -4419.91 |
| CL9_504298249PSV_F0520000CCAM03202P1 | Aus | 9 | 1203 | -4419.91 |
| CL9_504298497PSV_F0520000CCAM03202P1 | Aus | 10 | 1203 | -4419.91 |
| CL9_504298565PSV_F0520000CCAM03202P1 | Aus | 11 | 1203 | -4419.91 |
| CL9_504298628PSV_F0520000CCAM03202P1 | Aus | 12 | 1203 | -4419.91 |
| CL9_504298691PSV_F0520000CCAM03202P1 | Aus | 13 | 1203 | -4419.91 |
| CL9_504298754PSV_F0520000CCAM03202P1 | Aus | 14 | 1203 | -4419.91 |
| CL9_504298817PSV_F0520000CCAM03202P1 | Aus | 15 | 1203 | -4419.91 |
| CL9_504298881PSV_F0520000CCAM03202P1 | Aus | 16 | 1203 | -4419.91 |
| CL9_504298944PSV_F0520000CCAM03202P1 | Aus | 17 | 1203 | -4419.91 |
| CL9_504299007PSV_F0520000CCAM03202P1 | Aus | 18 | 1203 | -4419.91 |
| CL9_504299071PSV_F0520000CCAM03202P1 | Aus | 19 | 1203 | -4419.91 |
| CL9_504299274PSV_F0520000CCAM03202P1 | Aus | 20 | 1203 | -4419.91 |
| CL9_505263903PSV_F0520004CCAM01214P1 | Hunkab | 1 | 1214 | -4419.91 |
| CL9_505263971PSV_F0520004CCAM01214P1 | Hunkab | 2 | 1214 | -4419.91 |
| CL9_505264035PSV_F0520004CCAM01214P1 | Hunkab | 3 | 1214 | -4419.91 |
| CL9_505264099PSV_F0520004CCAM01214P1 | Hunkab | 4 | 1214 | -4419.91 |
| CL9_505264294PSV_F0520004CCAM01214P1 | Hunkab | 5 | 1214 | -4419.91 |
| CL9_505885237PSV_F0520936CCAM01221P1 | Probeer | 1 | 1221 | -4424.15 |
| CL9_505885306PSV_F0520936CCAM01221P1 | Probeer | 2 | 1221 | -4424.15 |
| CL9_505885370PSV_F0520936CCAM01221P1 | Probeer | 3 | 1221 | -4424.15 |
| CL9_505885434PSV_F0520936CCAM01221P1 | Probeer | 4 | 1221 | -4424.15 |
| CL9_505885628PSV_F0520936CCAM01221P1 | Probeer | 5 | 1221 | -4424.15 |
| CL9_508119174PSV_F0521370CCAM04245P1 | Kudis | 1 | 1246 | -4423.43 |
| CL9_508119242PSV_F0521370CCAM04245P1 | Kudis | 2 | 1246 | -4423.43 |
| CL9_508119306PSV_F0521370CCAM04245P1 | Kudis | 3 | 1246 | -4423.43 |
| CL9_508119550PSV_F0521370CCAM04245P1 | Kudis | 4 | 1246 | -4423.43 |
| CL9_508119618PSV_F0521370CCAM04245P1 | Kudis | 5 | 1246 | -4423.43 |
| CL9_508119682PSV_F0521370CCAM04245P1 | Kudis | 6 | 1246 | -4423.43 |
| CL9_508119926PSV_F0521370CCAM04245P1 | Kudis | 7 | 1246 | -4423.43 |
| CL9_508119994PSV_F0521370CCAM04245P1 | Kudis | 8 | 1246 | -4423.43 |
| CL9_508120197PSV_F0521370CCAM04245P1 | Kudis | 9 | 1246 | -4423.43 |
| CL9_508120965PSV_F0521370CCAM05245P1 | Purros_ccam | 5 | 1246 | -4423.43 |
| CL9_508458896PSV_F0522262CCAM01250P1 | Kuiseb | 1 | 1250 | -4426.71 |
| CL9_508458965PSV_F0522262CCAM01250P1 | Kuiseb | 2 | 1250 | -4426.71 |
| CL9_508459029PSV_F0522262CCAM01250P1 | Kuiseb | 3 | 1250 | -4426.71 |
| CL9_508459093PSV_F0522262CCAM01250P1 | Kuiseb | 4 | 1250 | -4426.71 |
| CL9_508459287PSV_F0522262CCAM01250P1 | Kuiseb | 5 | 1250 | -4426.71 |
| CL9_508639525PSV_F0522388CCAM03251P1 | Buntfeldschuh | 1 | 1252 | -4426.54 |
| CL9_508639976PSV_F0522388CCAM03251P1 | Buntfeldschuh | 5 | 1252 | -4426.54 |
| CL9_508640041PSV_F0522388CCAM03251P1 | Buntfeldschuh | 6 | 1252 | -4426.54 |
| CL9_508640278PSV_F0522388CCAM03251P1 | Buntfeldschuh | 7 | 1252 | -4426.54 |

| | | | | |
|---|---|---|---|---|
| CL9_508640353PSV_F0522388CCAM03251P1 | Buntfeldschuh | 8 | 1252 | -4426.54 |
| CL9_508640419PSV_F0522388CCAM03251P1 | Buntfeldschuh | 9 | 1252 | -4426.54 |
| CL9_508640615PSV_F0522388CCAM03251P1 | Buntfeldschuh | 10 | 1252 | -4426.54 |
| CL9_508640924PSV_F0522388CCAM04251P1 | Vingerklip | 1 | 1252 | -4426.54 |
| CL9_508641065PSV_F0522388CCAM04251P1 | Vingerklip | 3 | 1252 | -4426.54 |
| CL9_508641302PSV_F0522388CCAM04251P1 | Vingerklip | 4 | 1252 | -4426.54 |
| CL9_508641442PSV_F0522388CCAM04251P1 | Vingerklip | 6 | 1252 | -4426.54 |
| CL9_509257746PSV_F0522678CCAM01259P1 | Gross_Aub | 5 | 1259 | -4427.63 |
| CL9_509258072PSV_F0522678CCAM02259P1 | Groot_Aub | 1 | 1259 | -4427.63 |
| CL9_509258148PSV_F0522678CCAM02259P1 | Groot_Aub | 2 | 1259 | -4427.63 |
| CL9_509258214PSV_F0522678CCAM02259P1 | Groot_Aub | 3 | 1259 | -4427.63 |
| CL9_509258280PSV_F0522678CCAM02259P1 | Groot_Aub | 4 | 1259 | -4427.63 |
| CL9_509258518PSV_F0522678CCAM02259P1 | Groot_Aub | 5 | 1259 | -4427.63 |
| CL9_509258594PSV_F0522678CCAM02259P1 | Groot_Aub | 6 | 1259 | -4427.63 |
| CL9_509258660PSV_F0522678CCAM02259P1 | Groot_Aub | 7 | 1259 | -4427.63 |
| CL9_509258726PSV_F0522678CCAM02259P1 | Groot_Aub | 8 | 1259 | -4427.63 |
| CL9_509258792PSV_F0522678CCAM02259P1 | Groot_Aub | 9 | 1259 | -4427.63 |
| CL9_509258957PSV_F0522678CCAM02259P1 | Groot_Aub | 10 | 1259 | -4427.63 |
| CL9_509259301PSV_F0522678CCAM03259P1 | Gorob_ccam | 1 | 1259 | -4427.63 |
| CL9_509259377PSV_F0522678CCAM03259P1 | Gorob_ccam | 2 | 1259 | -4427.63 |
| CL9_509259443PSV_F0522678CCAM03259P1 | Gorob_ccam | 3 | 1259 | -4427.63 |
| CL9_509259739PSV_F0522678CCAM03259P1 | Gorob_ccam | 5 | 1259 | -4427.63 |
| CL9_509259805PSV_F0522678CCAM03259P1 | Gorob_ccam | 6 | 1259 | -4427.63 |
| CL9_509260032PSV_F0522678CCAM03259P1 | Gorob_ccam | 7 | 1259 | -4427.63 |
| CL9_509260104PSV_F0522678CCAM03259P1 | Gorob_ccam | 8 | 1259 | -4427.63 |
| CL9_509260301PSV_F0522678CCAM03259P1 | Gorob_ccam | 9 | 1259 | -4427.63 |
| CL9_509260613PSV_F0522678CCAM04259P1 | Grosskopf | 1 | 1259 | -4427.63 |
| CL9_509260688PSV_F0522678CCAM04259P1 | Grosskopf | 2 | 1259 | -4427.63 |
| CL9_509260754PSV_F0522678CCAM04259P1 | Grosskopf | 3 | 1259 | -4427.63 |
| CL9_509260820PSV_F0522678CCAM04259P1 | Grosskopf | 4 | 1259 | -4427.63 |
| CL9_509261015PSV_F0522678CCAM04259P1 | Grosskopf | 5 | 1259 | -4427.63 |
| CL9_509524280PSV_F0522772CCAM01262P1 | Gemsboktal | 1 | 1262 | -4428 |
| CL9_509524349PSV_F0522772CCAM01262P1 | Gemsboktal | 2 | 1262 | -4428 |
| CL9_509524413PSV_F0522772CCAM01262P1 | Gemsboktal | 3 | 1262 | -4428 |
| CL9_509524648PSV_F0522772CCAM01262P1 | Gemsboktal | 4 | 1262 | -4428 |
| CL9_509524716PSV_F0522772CCAM01262P1 | Gemsboktal | 5 | 1262 | -4428 |
| CL9_509524780PSV_F0522772CCAM01262P1 | Gemsboktal | 6 | 1262 | -4428 |
| CL9_509525015PSV_F0522772CCAM01262P1 | Gemsboktal | 7 | 1262 | -4428 |
| CL9_509525147PSV_F0522772CCAM01262P1 | Gemsboktal | 9 | 1262 | -4428 |
| CL9_509525649PSV_F0522772CCAM02262P1 | Ghaub | 1 | 1262 | -4428 |
| CL9_509525717PSV_F0522772CCAM02262P1 | Ghaub | 2 | 1262 | -4428 |
| CL9_509525781PSV_F0522772CCAM02262P1 | Ghaub | 3 | 1262 | -4428 |
| CL9_509525845PSV_F0522772CCAM02262P1 | Ghaub | 4 | 1262 | -4428 |
| CL9_509526046PSV_F0522772CCAM02262P1 | Ghaub | 5 | 1262 | -4428 |
| CL9_509704043PSV_F0530000CCAM01264P1 | Awahab | 1 | 1264 | -4430.07 |
| CL9_509704118PSV_F0530000CCAM01264P1 | Awahab | 2 | 1264 | -4430.07 |
| CL9_509704184PSV_F0530000CCAM01264P1 | Awahab | 3 | 1264 | -4430.07 |
| CL9_509704250PSV_F0530000CCAM01264P1 | Awahab | 4 | 1264 | -4430.07 |
| CL9_509704446PSV_F0530000CCAM01264P1 | Awahab | 5 | 1264 | -4430.07 |
| CL9_509704779PSV_F0530000CCAM02264P1 | Awa_gamteb | 1 | 1264 | -4430.07 |
| CL9_509704854PSV_F0530000CCAM02264P1 | Awa_gamteb | 2 | 1264 | -4430.07 |
| CL9_509704920PSV_F0530000CCAM02264P1 | Awa_gamteb | 3 | 1264 | -4430.07 |
| CL9_509704986PSV_F0530000CCAM02264P1 | Awa_gamteb | 4 | 1264 | -4430.07 |
| CL9_509705182PSV_F0530000CCAM02264P1 | Awa_gamteb | 5 | 1264 | -4430.07 |
| CL9_509882221PSV_F0530186CCAM01266P1 | Ugab | 5 | 1266 | -4432.08 |
| CL9_509884073PSV_F0530186CCAM04266P1 | Stockdale | 1 | 1266 | -4432.08 |
| CL9_509884145PSV_F0530186CCAM04266P1 | Stockdale | 2 | 1266 | -4432.08 |
| CL9_509884211PSV_F0530186CCAM04266P1 | Stockdale | 3 | 1266 | -4432.08 |
| CL9_509884441PSV_F0530186CCAM04266P1 | Stockdale | 4 | 1266 | -4432.08 |

| | | | | |
|---|---|---|---|---|
| CL9_509884509PSV_F0530186CCAM04266P1 | Stockdale | 5 | 1266 | -4432.08 |
| CL9_509884575PSV_F0530186CCAM04266P1 | Stockdale | 6 | 1266 | -4432.08 |
| CL9_509884806PSV_F0530186CCAM04266P1 | Stockdale | 7 | 1266 | -4432.08 |
| CL9_509884874PSV_F0530186CCAM04266P1 | Stockdale | 8 | 1266 | -4432.08 |
| CL9_509885069PSV_F0530186CCAM04266P1 | Stockdale | 9 | 1266 | -4432.08 |
| CL9_510145534PSV_F0530372CCAM01269P1 | Swartpunt | 1 | 1269 | -4433.46 |
| CL9_510145602PSV_F0530372CCAM01269P1 | Swartpunt | 2 | 1269 | -4433.46 |
| CL9_510326884PSV_F0530636CCAM01271P1 | Uniab | 1 | 1271 | -4432.62 |
| CL9_510326952PSV_F0530636CCAM01271P1 | Uniab | 2 | 1271 | -4432.62 |
| CL9_510327016PSV_F0530636CCAM01271P1 | Uniab | 3 | 1271 | -4432.62 |
| CL9_510327080PSV_F0530636CCAM01271P1 | Uniab | 4 | 1271 | -4432.62 |
| CL9_510327313PSV_F0530636CCAM01271P1 | Uniab | 5 | 1271 | -4432.62 |
| CL9_510327378PSV_F0530636CCAM01271P1 | Uniab | 6 | 1271 | -4432.62 |
| CL9_510327442PSV_F0530636CCAM01271P1 | Uniab | 7 | 1271 | -4432.62 |
| CL9_510327506PSV_F0530636CCAM01271P1 | Uniab | 8 | 1271 | -4432.62 |
| CL9_510327741PSV_F0530636CCAM01271P1 | Uniab | 9 | 1271 | -4432.62 |
| CL9_510327806PSV_F0530636CCAM01271P1 | Uniab | 10 | 1271 | -4432.62 |
| CL9_510327870PSV_F0530636CCAM01271P1 | Uniab | 11 | 1271 | -4432.62 |
| CL9_510327934PSV_F0530636CCAM01271P1 | Uniab | 12 | 1271 | -4432.62 |
| CL9_510328169PSV_F0530636CCAM01271P1 | Uniab | 13 | 1271 | -4432.62 |
| CL9_510328234PSV_F0530636CCAM01271P1 | Uniab | 14 | 1271 | -4432.62 |
| CL9_510328298PSV_F0530636CCAM01271P1 | Uniab | 15 | 1271 | -4432.62 |
| CL9_510328362PSV_F0530636CCAM01271P1 | Uniab | 16 | 1271 | -4432.62 |
| CL9_510328703PSV_F0530636CCAM02271P1 | Tumas | 1 | 1271 | -4432.62 |
| CL9_510328771PSV_F0530636CCAM02271P1 | Tumas | 2 | 1271 | -4432.62 |
| CL9_510329202PSV_F0530636CCAM02271P1 | Tumas | 6 | 1271 | -4432.62 |
| CL9_510329568PSV_F0530636CCAM02271P1 | Tumas | 9 | 1271 | -4432.62 |
| CL9_510329761PSV_F0530636CCAM02271P1 | Tumas | 10 | 1271 | -4432.62 |
| CL9_510502632PSV_F0530636CCAM01273P1 | Kleinberg | 1 | 1273 | -4432.62 |
| CL9_510502700PSV_F0530636CCAM01273P1 | Kleinberg | 2 | 1273 | -4432.62 |
| CL9_510502764PSV_F0530636CCAM01273P1 | Kleinberg | 3 | 1273 | -4432.62 |
| CL9_510504291PSV_F0530636CCAM02273P1 | Tumas_2 | 10 | 1273 | -4432.62 |
| CL9_510679620PSV_F0531056CCAM02275P1 | Palmhorst | 1 | 1275 | -4427.36 |
| CL9_510679689PSV_F0531056CCAM02275P1 | Palmhorst | 2 | 1275 | -4427.36 |
| CL9_510679753PSV_F0531056CCAM02275P1 | Palmhorst | 3 | 1275 | -4427.36 |
| CL9_510680000PSV_F0531056CCAM02275P1 | Palmhorst | 4 | 1275 | -4427.36 |
| CL9_510680068PSV_F0531056CCAM02275P1 | Palmhorst | 5 | 1275 | -4427.36 |
| CL9_510680132PSV_F0531056CCAM02275P1 | Palmhorst | 6 | 1275 | -4427.36 |
| CL9_510680379PSV_F0531056CCAM02275P1 | Palmhorst | 7 | 1275 | -4427.36 |
| CL9_510680447PSV_F0531056CCAM02275P1 | Palmhorst | 8 | 1275 | -4427.36 |
| CL9_510680511PSV_F0531056CCAM02275P1 | Palmhorst | 9 | 1275 | -4427.36 |
| CL9_510680713PSV_F0531056CCAM02275P1 | Palmhorst | 10 | 1275 | -4427.36 |
| CL9_510681518PSV_F0531056CCAM03275P1 | Palmwag | 5 | 1275 | -4427.36 |
| CL9_510681584PSV_F0531056CCAM03275P1 | Palmwag | 6 | 1275 | -4427.36 |
| CL9_510681832PSV_F0531056CCAM03275P1 | Palmwag | 7 | 1275 | -4427.36 |
| CL9_510681908PSV_F0531056CCAM03275P1 | Palmwag | 8 | 1275 | -4427.36 |
| CL9_510681974PSV_F0531056CCAM03275P1 | Palmwag | 9 | 1275 | -4427.36 |
| CL9_510682179PSV_F0531056CCAM03275P1 | Palmwag | 10 | 1275 | -4427.36 |
| CL9_510682544PSV_F0531056CCAM04275P1 | Mirabib_ccam_DRT_2 | 1 | 1275 | -4427.36 |
| CL9_510682612PSV_F0531056CCAM04275P1 | Mirabib_ccam_DRT_2 | 2 | 1275 | -4427.36 |
| CL9_510682676PSV_F0531056CCAM04275P1 | Mirabib_ccam_DRT_2 | 3 | 1275 | -4427.36 |
| CL9_510682740PSV_F0531056CCAM04275P1 | Mirabib_ccam_DRT_2 | 4 | 1275 | -4427.36 |
| CL9_510682934PSV_F0531056CCAM04275P1 | Mirabib_ccam_DRT_2 | 5 | 1275 | -4427.36 |
| CL9_510765941PSV_F0531056CCAM03276P1 | Aranos | 2 | 1276 | -4427.36 |
| CL9_510766006PSV_F0531056CCAM03276P1 | Aranos | 3 | 1276 | -4427.36 |
| CL9_510766071PSV_F0531056CCAM03276P1 | Aranos | 4 | 1276 | -4427.36 |
| CL9_510766275PSV_F0531056CCAM03276P1 | Aranos | 5 | 1276 | -4427.36 |
| CL9_510855914PSV_F0531182CCAM01277P1 | Vogelfederberg | 2 | 1277 | -4426.57 |
| CL9_510855978PSV_F0531182CCAM01277P1 | Vogelfederberg | 3 | 1277 | -4426.57 |

| | | | | |
|---|---|---|---|---|
| CL9_510856236PSV_F0531182CCAM01277P1 | Vogelfederberg | 5 | 1277 | -4426.57 |
| CL9_510944517PSV_F0531182CCAM01278P1 | Tsarabis | 5 | 1278 | -4426.57 |
| CL9_517775980PSV_F0542202CCAM01355P1 | Auchas | 1 | 1355 | -4434.87 |
| CL9_517776048PSV_F0542202CCAM01355P1 | Auchas | 2 | 1355 | -4434.87 |
| CL9_517776250PSV_F0542202CCAM01355P1 | Auchas | 3 | 1355 | -4434.87 |
| CL9_517776315PSV_F0542202CCAM01355P1 | Auchas | 4 | 1355 | -4434.87 |
| CL9_517776562PSV_F0542202CCAM01355P1 | Auchas | 5 | 1355 | -4434.87 |
| CL9_517776630PSV_F0542202CCAM01355P1 | Auchas | 6 | 1355 | -4434.87 |
| CL9_517776897PSV_F0542202CCAM01355P1 | Auchas | 8 | 1355 | -4434.87 |
| CL9_517776961PSV_F0542202CCAM01355P1 | Auchas | 9 | 1355 | -4434.87 |
| CL9_517777163PSV_F0542202CCAM01355P1 | Auchas | 10 | 1355 | -4434.87 |
| CL9_517777502PSV_F0542202CCAM02355P1 | Kaisosi | 1 | 1355 | -4434.87 |
| CL9_517777570PSV_F0542202CCAM02355P1 | Kaisosi | 2 | 1355 | -4434.87 |
| CL9_517777634PSV_F0542202CCAM02355P1 | Kaisosi | 3 | 1355 | -4434.87 |
| CL9_517777698PSV_F0542202CCAM02355P1 | Kaisosi | 4 | 1355 | -4434.87 |
| CL9_517777900PSV_F0542202CCAM02355P1 | Kaisosi | 5 | 1355 | -4434.87 |
| CL9_517778275PSV_F0542202CCAM03355P1 | Inamagando | 1 | 1355 | -4434.87 |
| CL9_517778343PSV_F0542202CCAM03355P1 | Inamagando | 2 | 1355 | -4434.87 |
| CL9_517778407PSV_F0542202CCAM03355P1 | Inamagando | 3 | 1355 | -4434.87 |
| CL9_517778509PSV_F0542202CCAM03355P1 | Inamagando | 4 | 1355 | -4434.87 |
| CL9_517778711PSV_F0542202CCAM03355P1 | Inamagando | 5 | 1355 | -4434.87 |
| CL9_517778776PSV_F0542202CCAM03355P1 | Inamagando | 6 | 1355 | -4434.87 |
| CL9_517778879PSV_F0542202CCAM03355P1 | Inamagando | 7 | 1355 | -4434.87 |
| CL9_517778943PSV_F0542202CCAM03355P1 | Inamagando | 8 | 1355 | -4434.87 |
| CL9_517779145PSV_F0542202CCAM03355P1 | Inamagando | 9 | 1355 | -4434.87 |
| CL9_517779512PSV_F0542202CCAM04355P1 | Horingbaai_ccam | 1 | 1355 | -4434.87 |
| CL9_517779586PSV_F0542202CCAM04355P1 | Horingbaai_ccam | 2 | 1355 | -4434.87 |
| CL9_517779650PSV_F0542202CCAM04355P1 | Horingbaai_ccam | 3 | 1355 | -4434.87 |
| CL9_517779714PSV_F0542202CCAM04355P1 | Horingbaai_ccam | 4 | 1355 | -4434.87 |
| CL9_517779916PSV_F0542202CCAM04355P1 | Horingbaai_ccam | 5 | 1355 | -4434.87 |
| CL9_518044352PSV_F0542280CCAM01358P1 | Otiiha | 1 | 1358 | -4435.59 |
| CL9_518044428PSV_F0542280CCAM01358P1 | Otiiha | 2 | 1358 | -4435.59 |
| CL9_518044494PSV_F0542280CCAM01358P1 | Otiiha | 3 | 1358 | -4435.59 |
| CL9_518044560PSV_F0542280CCAM01358P1 | Otiiha | 4 | 1358 | -4435.59 |
| CL9_518044764PSV_F0542280CCAM01358P1 | Otiiha | 5 | 1358 | -4435.59 |
| CL9_518045097PSV_F0542280CCAM02358P1 | Otjihase | 1 | 1358 | -4435.59 |
| CL9_518045173PSV_F0542280CCAM02358P1 | Otjihase | 2 | 1358 | -4435.59 |
| CL9_518045239PSV_F0542280CCAM02358P1 | Otjihase | 3 | 1358 | -4435.59 |
| CL9_518045305PSV_F0542280CCAM02358P1 | Otjihase | 4 | 1358 | -4435.59 |
| CL9_518045509PSV_F0542280CCAM02358P1 | Otjihase | 5 | 1358 | -4435.59 |
| CL9_518045845PSV_F0542280CCAM03358P1 | Otjikoto | 1 | 1358 | -4435.59 |
| CL9_518045921PSV_F0542280CCAM03358P1 | Otjikoto | 2 | 1358 | -4435.59 |
| CL9_518045987PSV_F0542280CCAM03358P1 | Otjikoto | 3 | 1358 | -4435.59 |
| CL9_518046053PSV_F0542280CCAM03358P1 | Otjikoto | 4 | 1358 | -4435.59 |
| CL9_518046258PSV_F0542280CCAM03358P1 | Otjikoto | 5 | 1358 | -4435.59 |
| CL9_518046625PSV_F0542280CCAM04358P1 | Otjimbingwe | 1 | 1358 | -4435.59 |
| CL9_518046701PSV_F0542280CCAM04358P1 | Otjimbingwe | 2 | 1358 | -4435.59 |
| CL9_518046767PSV_F0542280CCAM04358P1 | Otjimbingwe | 3 | 1358 | -4435.59 |
| CL9_518046833PSV_F0542280CCAM04358P1 | Otjimbingwe | 4 | 1358 | -4435.59 |
| CL9_518047038PSV_F0542280CCAM04358P1 | Otjimbingwe | 5 | 1358 | -4435.59 |
| CL9_518134642PSV_F0542280CCAM02359P1 | Otjosondu | 1 | 1359 | -4435.59 |
| CL9_518134718PSV_F0542280CCAM02359P1 | Otjosondu | 2 | 1359 | -4435.59 |
| CL9_518134784PSV_F0542280CCAM02359P1 | Otjosondu | 3 | 1359 | -4435.59 |
| CL9_518134850PSV_F0542280CCAM02359P1 | Otjosondu | 4 | 1359 | -4435.59 |
| CL9_518135055PSV_F0542280CCAM02359P1 | Otjosondu | 5 | 1359 | -4435.59 |
| CL9_518227614PSV_F0542280CCAM01360P1 | Charlottenfelder | 1 | 1360 | -4435.59 |
| CL9_518227746PSV_F0542280CCAM01360P1 | Charlottenfelder | 3 | 1360 | -4435.59 |
| CL9_518227810PSV_F0542280CCAM01360P1 | Charlottenfelder | 4 | 1360 | -4435.59 |
| CL9_518228452PSV_F0542280CCAM02360P1 | Chameis_Bay | 1 | 1360 | -4435.59 |

| | | | | |
|---|---|---|---|---|
| CL9_518228527PSV_F0542280CCAM02360P1 | Chameis_Bay | 2 | 1360 | -4435.59 |
| CL9_518228592PSV_F0542280CCAM02360P1 | Chameis_Bay | 3 | 1360 | -4435.59 |
| CL9_518228833PSV_F0542280CCAM02360P1 | Chameis_Bay | 4 | 1360 | -4435.59 |
| CL9_518228908PSV_F0542280CCAM02360P1 | Chameis_Bay | 5 | 1360 | -4435.59 |
| CL9_518228974PSV_F0542280CCAM02360P1 | Chameis_Bay | 6 | 1360 | -4435.59 |
| CL9_518229198PSV_F0542280CCAM02360P1 | Chameis_Bay | 7 | 1360 | -4435.59 |
| CL9_518229273PSV_F0542280CCAM02360P1 | Chameis_Bay | 8 | 1360 | -4435.59 |
| CL9_518229338PSV_F0542280CCAM02360P1 | Chameis_Bay | 9 | 1360 | -4435.59 |
| CL9_518229520PSV_F0542280CCAM02360P1 | Chameis_Bay | 10 | 1360 | -4435.59 |
| CL9_518492086PSV_F0542280CCAM02361P1 | Ongeama | 1 | 1363 | -4435.59 |
| CL9_518492162PSV_F0542280CCAM02361P1 | Ongeama | 2 | 1363 | -4435.59 |
| CL9_518492228PSV_F0542280CCAM02361P1 | Ongeama | 3 | 1363 | -4435.59 |
| CL9_518492478PSV_F0542280CCAM02361P1 | Ongeama | 4 | 1363 | -4435.59 |
| CL9_518492554PSV_F0542280CCAM02361P1 | Ongeama | 5 | 1363 | -4435.59 |
| CL9_518492620PSV_F0542280CCAM02361P1 | Ongeama | 6 | 1363 | -4435.59 |
| CL9_518492870PSV_F0542280CCAM02361P1 | Ongeama | 7 | 1363 | -4435.59 |
| CL9_518492946PSV_F0542280CCAM02361P1 | Ongeama | 8 | 1363 | -4435.59 |
| CL9_518493012PSV_F0542280CCAM02361P1 | Ongeama | 9 | 1363 | -4435.59 |
| CL9_518493217PSV_F0542280CCAM02361P1 | Ongeama | 10 | 1363 | -4435.59 |
| CL9_518493614PSV_F0542280CCAM03361P1 | Onganja | 1 | 1363 | -4435.59 |
| CL9_518493683PSV_F0542280CCAM03361P1 | Onganja | 2 | 1363 | -4435.59 |
| CL9_518493991PSV_F0542280CCAM03361P1 | Onganja | 4 | 1363 | -4435.59 |
| CL9_518494060PSV_F0542280CCAM03361P1 | Onganja | 5 | 1363 | -4435.59 |
| CL9_518494124PSV_F0542280CCAM03361P1 | Onganja | 6 | 1363 | -4435.59 |
| CL9_518494346PSV_F0542280CCAM03361P1 | Onganja | 7 | 1363 | -4435.59 |
| CL9_518494410PSV_F0542280CCAM03361P1 | Onganja | 8 | 1363 | -4435.59 |
| CL9_518494600PSV_F0542280CCAM03361P1 | Onganja | 9 | 1363 | -4435.59 |
| CL9_518753819PSV_F0542280CCAM02366P1 | Omulonga | 3 | 1366 | -4435.59 |
| CL9_518754054PSV_F0542280CCAM02366P1 | Omulonga | 4 | 1366 | -4435.59 |
| CL9_518754130PSV_F0542280CCAM02366P1 | Omulonga | 5 | 1366 | -4435.59 |
| CL9_518754196PSV_F0542280CCAM02366P1 | Omulonga | 6 | 1366 | -4435.59 |
| CL9_518932584PSV_F0542280CCAM02368P1 | Onguati | 1 | 1368 | -4435.59 |
| CL9_518932659PSV_F0542280CCAM02368P1 | Onguati | 2 | 1368 | -4435.59 |
| CL9_518933326PSV_F0542280CCAM02368P1 | Onguati | 6 | 1368 | -4435.59 |
| CL9_518933565PSV_F0542280CCAM02368P1 | Onguati | 7 | 1368 | -4435.59 |
| CL9_518933640PSV_F0542280CCAM02368P1 | Onguati | 8 | 1368 | -4435.59 |
| CL9_518933878PSV_F0542280CCAM02368P1 | Onguati | 9 | 1368 | -4435.59 |
| CL9_518933953PSV_F0542280CCAM02368P1 | Onguati | 10 | 1368 | -4435.59 |
| CL9_518934433PSV_F0542280CCAM03368P1 | Onganja_2 | 3 | 1368 | -4435.59 |
| CL9_518934497PSV_F0542280CCAM03368P1 | Onganja_2 | 4 | 1368 | -4435.59 |
| CL9_518934560PSV_F0542280CCAM03368P1 | Onganja_2 | 5 | 1368 | -4435.59 |
| CL9_519197182PSV_F0542508CCAM01371P1 | Kupferberg | 1 | 1371 | -4433.31 |
| CL9_519197258PSV_F0542508CCAM01371P1 | Kupferberg | 2 | 1371 | -4433.31 |
| CL9_519197573PSV_F0542508CCAM01371P1 | Kupferberg | 4 | 1371 | -4433.31 |
| CL9_519197964PSV_F0542508CCAM01371P1 | Kupferberg | 7 | 1371 | -4433.31 |
| CL9_519198040PSV_F0542508CCAM01371P1 | Kupferberg | 8 | 1371 | -4433.31 |
| CL9_519198106PSV_F0542508CCAM01371P1 | Kupferberg | 9 | 1371 | -4433.31 |
| CL9_519198278PSV_F0542508CCAM01371P1 | Kupferberg | 10 | 1371 | -4433.31 |
| CL9_519198604PSV_F0542508CCAM02371P1 | Kranzberg | 1 | 1371 | -4433.31 |
| CL9_519198680PSV_F0542508CCAM02371P1 | Kranzberg | 2 | 1371 | -4433.31 |
| CL9_519198746PSV_F0542508CCAM02371P1 | Kranzberg | 3 | 1371 | -4433.31 |
| CL9_519198812PSV_F0542508CCAM02371P1 | Kranzberg | 4 | 1371 | -4433.31 |
| CL9_519553416PSV_F0543036CCAM01375P1 | Andara | 1 | 1375 | -4429.46 |
| CL9_519553491PSV_F0543036CCAM01375P1 | Andara | 2 | 1375 | -4429.46 |
| CL9_519553557PSV_F0543036CCAM01375P1 | Andara | 3 | 1375 | -4429.46 |
| CL9_519553785PSV_F0543036CCAM01375P1 | Andara | 4 | 1375 | -4429.46 |
| CL9_519553853PSV_F0543036CCAM01375P1 | Andara | 5 | 1375 | -4429.46 |
| CL9_519553919PSV_F0543036CCAM01375P1 | Andara | 6 | 1375 | -4429.46 |
| CL9_519554147PSV_F0543036CCAM01375P1 | Andara | 7 | 1375 | -4429.46 |

| | | | | |
|---|---|---|---|---|
| CL9_519554215PSV_F0543036CCAM01375P1 | Andara | 8 | 1375 | -4429.46 |
| CL9_519554411PSV_F0543036CCAM01375P1 | Andara | 9 | 1375 | -4429.46 |
| CL9_519554735PSV_F0543036CCAM02375P1 | Okoloti | 1 | 1375 | -4429.46 |
| CL9_519554813PSV_F0543036CCAM02375P1 | Okoloti | 2 | 1375 | -4429.46 |
| CL9_519554879PSV_F0543036CCAM02375P1 | Okoloti | 3 | 1375 | -4429.46 |
| CL9_519554945PSV_F0543036CCAM02375P1 | Okoloti | 4 | 1375 | -4429.46 |
| CL9_519555140PSV_F0543036CCAM02375P1 | Okoloti | 5 | 1375 | -4429.46 |
| CL9_519556203PSV_F0543036CCAM04375P1 | Khorixas | 1 | 1375 | -4429.46 |
| CL9_519556279PSV_F0543036CCAM04375P1 | Khorixas | 2 | 1375 | -4429.46 |
| CL9_519556345PSV_F0543036CCAM04375P1 | Khorixas | 3 | 1375 | -4429.46 |
| CL9_519556561PSV_F0543036CCAM04375P1 | Khorixas | 4 | 1375 | -4429.46 |
| CL9_519556637PSV_F0543036CCAM04375P1 | Khorixas | 5 | 1375 | -4429.46 |
| CL9_519556703PSV_F0543036CCAM04375P1 | Khorixas | 6 | 1375 | -4429.46 |
| CL9_519556918PSV_F0543036CCAM04375P1 | Khorixas | 7 | 1375 | -4429.46 |
| CL9_519556994PSV_F0543036CCAM04375P1 | Khorixas | 8 | 1375 | -4429.46 |
| CL9_519557060PSV_F0543036CCAM04375P1 | Khorixas | 9 | 1375 | -4429.46 |
| CL9_519557212PSV_F0543036CCAM04375P1 | Khorixas | 10 | 1375 | -4429.46 |
| CL9_519819184PSV_F0550000CCAM01378P1 | Tombua | 1 | 1378 | -4429.39 |
| CL9_519819380PSV_F0550000CCAM01378P1 | Tombua | 4 | 1378 | -4429.39 |
| CL9_519819483PSV_F0550000CCAM01378P1 | Tombua | 5 | 1378 | -4429.39 |
| CL9_519819547PSV_F0550000CCAM01378P1 | Tombua | 6 | 1378 | -4429.39 |
| CL9_519820050PSV_F0550000CCAM01378P1 | Tombua | 11 | 1378 | -4429.39 |
| CL9_519820114PSV_F0550000CCAM01378P1 | Tombua | 12 | 1378 | -4429.39 |
| CL9_519820219PSV_F0550000CCAM01378P1 | Tombua | 13 | 1378 | -4429.39 |
| CL9_519820283PSV_F0550000CCAM01378P1 | Tombua | 14 | 1378 | -4429.39 |
| CL9_519820549PSV_F0550000CCAM01378P1 | Tombua | 16 | 1378 | -4429.39 |
| CL9_519998487PSV_F0550310CCAM01380P1 | Koes | 1 | 1380 | -4428.65 |
| CL9_519998555PSV_F0550310CCAM01380P1 | Koes | 2 | 1380 | -4428.65 |
| CL9_519998619PSV_F0550310CCAM01380P1 | Koes | 3 | 1380 | -4428.65 |
| CL9_519998683PSV_F0550310CCAM01380P1 | Koes | 4 | 1380 | -4428.65 |
| CL9_519998747PSV_F0550310CCAM01380P1 | Koes | 5 | 1380 | -4428.65 |
| CL9_519998811PSV_F0550310CCAM01380P1 | Koes | 6 | 1380 | -4428.65 |
| CL9_519998875PSV_F0550310CCAM01380P1 | Koes | 7 | 1380 | -4428.65 |
| CL9_519998939PSV_F0550310CCAM01380P1 | Koes | 8 | 1380 | -4428.65 |
| CL9_519999003PSV_F0550310CCAM01380P1 | Koes | 9 | 1380 | -4428.65 |
| CL9_519999205PSV_F0550310CCAM01380P1 | Koes | 10 | 1380 | -4428.65 |
| CL9_519999577PSV_F0550310CCAM02380P1 | Onawa | 1 | 1380 | -4428.65 |
| CL9_519999645PSV_F0550310CCAM02380P1 | Onawa | 2 | 1380 | -4428.65 |
| CL9_519999709PSV_F0550310CCAM02380P1 | Onawa | 3 | 1380 | -4428.65 |
| CL9_519999773PSV_F0550310CCAM02380P1 | Onawa | 4 | 1380 | -4428.65 |
| CL9_519999975PSV_F0550310CCAM02380P1 | Onawa | 5 | 1380 | -4428.65 |
| CL9_520176190PSV_F0550310CCAM02382P1 | Rundu | 1 | 1382 | -4428.65 |
| CL9_520176266PSV_F0550310CCAM02382P1 | Rundu | 2 | 1382 | -4428.65 |
| CL9_520176332PSV_F0550310CCAM02382P1 | Rundu | 3 | 1382 | -4428.65 |
| CL9_520176399PSV_F0550310CCAM02382P1 | Rundu | 4 | 1382 | -4428.65 |
| CL9_520176647PSV_F0550310CCAM02382P1 | Rundu | 5 | 1382 | -4428.65 |
| CL9_520176723PSV_F0550310CCAM02382P1 | Rundu | 6 | 1382 | -4428.65 |
| CL9_520176789PSV_F0550310CCAM02382P1 | Rundu | 7 | 1382 | -4428.65 |
| CL9_520176855PSV_F0550310CCAM02382P1 | Rundu | 8 | 1382 | -4428.65 |
| CL9_520176921PSV_F0550310CCAM02382P1 | Rundu | 9 | 1382 | -4428.65 |
| CL9_520177125PSV_F0550310CCAM02382P1 | Rundu | 10 | 1382 | -4428.65 |
| CL9_520349808PSV_F0550538CCAM01384P1 | Berseba | 1 | 1384 | -4427.95 |
| CL9_520349877PSV_F0550538CCAM01384P1 | Berseba | 2 | 1384 | -4427.95 |
| CL9_520439221PSV_F0550940CCAM01385P1 | Epembe | 1 | 1385 | -4426.35 |
| CL9_520439289PSV_F0550940CCAM01385P1 | Epembe | 2 | 1385 | -4426.35 |
| CL9_520439353PSV_F0550940CCAM01385P1 | Epembe | 3 | 1385 | -4426.35 |
| CL9_520439417PSV_F0550940CCAM01385P1 | Epembe | 4 | 1385 | -4426.35 |
| CL9_520439619PSV_F0550940CCAM01385P1 | Epembe | 5 | 1385 | -4426.35 |
| CL9_520533317PSV_F0551312CCAM01386P1 | Trekkopje | 1 | 1386 | -4421.8 |

| | | | | |
|---|---|---|---|---|
| CL9_520533392PSV_F0551312CCAM01386P1 | Trekkopje | 2 | 1386 | -4421.8 |
| CL9_520533459PSV_F0551312CCAM01386P1 | Trekkopje | 3 | 1386 | -4421.8 |
| CL9_520533697PSV_F0551312CCAM01386P1 | Trekkopje | 4 | 1386 | -4421.8 |
| CL9_520533769PSV_F0551312CCAM01386P1 | Trekkopje | 5 | 1386 | -4421.8 |
| CL9_520533835PSV_F0551312CCAM01386P1 | Trekkopje | 6 | 1386 | -4421.8 |
| CL9_520534074PSV_F0551312CCAM01386P1 | Trekkopje | 7 | 1386 | -4421.8 |
| CL9_520534146PSV_F0551312CCAM01386P1 | Trekkopje | 8 | 1386 | -4421.8 |
| CL9_520534321PSV_F0551312CCAM01386P1 | Trekkopje | 9 | 1386 | -4421.8 |
| CL9_520616740PSV_F0551336CCAM01387P1 | Noordoewer | 1 | 1387 | -4421.69 |
| CL9_520616815PSV_F0551336CCAM01387P1 | Noordoewer | 2 | 1387 | -4421.69 |
| CL9_520616881PSV_F0551336CCAM01387P1 | Noordoewer | 3 | 1387 | -4421.69 |
| CL9_520617111PSV_F0551336CCAM01387P1 | Noordoewer | 4 | 1387 | -4421.69 |
| CL9_520617475PSV_F0551336CCAM01387P1 | Noordoewer | 7 | 1387 | -4421.69 |
| CL9_520617542PSV_F0551336CCAM01387P1 | Noordoewer | 8 | 1387 | -4421.69 |
| CL9_520617738PSV_F0551336CCAM01387P1 | Noordoewer | 9 | 1387 | -4421.69 |
| CL9_521594927PSV_F0551420CCAM01398P1 | Luanda | 1 | 1398 | -4420.63 |
| CL9_521595003PSV_F0551420CCAM01398P1 | Luanda | 2 | 1398 | -4420.63 |
| CL9_521595135PSV_F0551420CCAM01398P1 | Luanda | 4 | 1398 | -4420.63 |
| CL9_521595340PSV_F0551420CCAM01398P1 | Luanda | 5 | 1398 | -4420.63 |
| CL9_521682662PSV_F0551864CCAM01399P1 | Arandis | 1 | 1399 | -4419.35 |
| CL9_521682803PSV_F0551864CCAM01399P1 | Arandis | 3 | 1399 | -4419.35 |
| CL9_521683193PSV_F0551864CCAM01399P1 | Arandis | 6 | 1399 | -4419.35 |
| CL9_521683442PSV_F0551864CCAM01399P1 | Arandis | 7 | 1399 | -4419.35 |
| CL9_521683517PSV_F0551864CCAM01399P1 | Arandis | 8 | 1399 | -4419.35 |
| CL9_521683583PSV_F0551864CCAM01399P1 | Arandis | 9 | 1399 | -4419.35 |
| CL9_521953961PSV_F0552444CCAM02402P1 | Songo | 1 | 1402 | -4417.31 |
| CL9_521954026PSV_F0552444CCAM02402P1 | Songo | 2 | 1402 | -4417.31 |
| CL9_521954090PSV_F0552444CCAM02402P1 | Songo | 3 | 1402 | -4417.31 |
| CL9_521954325PSV_F0552444CCAM02402P1 | Songo | 5 | 1402 | -4417.31 |
| CL9_521954389PSV_F0552444CCAM02402P1 | Songo | 6 | 1402 | -4417.31 |
| CL9_521954561PSV_F0552444CCAM02402P1 | Songo | 7 | 1402 | -4417.31 |
| CL9_521954625PSV_F0552444CCAM02402P1 | Songo | 8 | 1402 | -4417.31 |
| CL9_521954689PSV_F0552444CCAM02402P1 | Songo | 9 | 1402 | -4417.31 |
| CL9_521954861PSV_F0552444CCAM02402P1 | Songo | 10 | 1402 | -4417.31 |
| CL9_522837438PSV_F0560462CCAM02412P1 | Huambo | 1 | 1412 | -4415.7 |
| CL9_522837506PSV_F0560462CCAM02412P1 | Huambo | 2 | 1412 | -4415.7 |
| CL9_522837570PSV_F0560462CCAM02412P1 | Huambo | 3 | 1412 | -4415.7 |
| CL9_522837634PSV_F0560462CCAM02412P1 | Huambo | 4 | 1412 | -4415.7 |
| CL9_522837836PSV_F0560462CCAM02412P1 | Huambo | 5 | 1412 | -4415.7 |
| CL9_523011821PSV_F0560774CCAM01414P1 | Okahandja | 1 | 1414 | -4412.15 |
| CL9_523011896PSV_F0560774CCAM01414P1 | Okahandja | 2 | 1414 | -4412.15 |
| CL9_523011962PSV_F0560774CCAM01414P1 | Okahandja | 3 | 1414 | -4412.15 |
| CL9_523012285PSV_F0560774CCAM01414P1 | Okahandja | 5 | 1414 | -4412.15 |
| CL9_523012351PSV_F0560774CCAM01414P1 | Okahandja | 6 | 1414 | -4412.15 |
| CL9_523012599PSV_F0560774CCAM01414P1 | Okahandja | 7 | 1414 | -4412.15 |
| CL9_523012674PSV_F0560774CCAM01414P1 | Okahandja | 8 | 1414 | -4412.15 |
| CL9_523012740PSV_F0560774CCAM01414P1 | Okahandja | 9 | 1414 | -4412.15 |
| CL9_523012943PSV_F0560774CCAM01414P1 | Okahandja | 10 | 1414 | -4412.15 |
| CL9_523013289PSV_F0560774CCAM02414P1 | Swakopmund | 1 | 1414 | -4412.15 |
| CL9_523013421PSV_F0560774CCAM02414P1 | Swakopmund | 3 | 1414 | -4412.15 |
| CL9_523013668PSV_F0560774CCAM02414P1 | Swakopmund | 4 | 1414 | -4412.15 |
| CL9_523013737PSV_F0560774CCAM02414P1 | Swakopmund | 5 | 1414 | -4412.15 |
| CL9_523013801PSV_F0560774CCAM02414P1 | Swakopmund | 6 | 1414 | -4412.15 |
| CL9_523014048PSV_F0560774CCAM02414P1 | Swakopmund | 7 | 1414 | -4412.15 |
| CL9_523014117PSV_F0560774CCAM02414P1 | Swakopmund | 8 | 1414 | -4412.15 |
| CL9_523014181PSV_F0560774CCAM02414P1 | Swakopmund | 9 | 1414 | -4412.15 |
| CL9_523014754PSV_F0560774CCAM03414P1 | Walvis_Bay | 1 | 1414 | -4412.15 |
| CL9_523014830PSV_F0560774CCAM03414P1 | Walvis_Bay | 2 | 1414 | -4412.15 |
| CL9_523014896PSV_F0560774CCAM03414P1 | Walvis_Bay | 3 | 1414 | -4412.15 |

| | | | | |
|---|---|---|---|---|
| CL9_523015145PSV_F0560774CCAM03414P1 | Walvis_Bay | 4 | 1414 | -4412.15 |
| CL9_523015221PSV_F0560774CCAM03414P1 | Walvis_Bay | 5 | 1414 | -4412.15 |
| CL9_523015292PSV_F0560774CCAM03414P1 | Walvis_Bay | 6 | 1414 | -4412.15 |
| CL9_523015616PSV_F0560774CCAM03414P1 | Walvis_Bay | 8 | 1414 | -4412.15 |
| CL9_523015682PSV_F0560774CCAM03414P1 | Walvis_Bay | 9 | 1414 | -4412.15 |
| CL9_523192872PSV_F0561122CCAM01416P1 | Chibia | 1 | 1416 | -4410.83 |
| CL9_523192941PSV_F0561122CCAM01416P1 | Chibia | 2 | 1416 | -4410.83 |
| CL9_523193005PSV_F0561122CCAM01416P1 | Chibia | 3 | 1416 | -4410.83 |
| CL9_523193240PSV_F0561122CCAM01416P1 | Chibia | 4 | 1416 | -4410.83 |
| CL9_523193308PSV_F0561122CCAM01416P1 | Chibia | 5 | 1416 | -4410.83 |
| CL9_523193372PSV_F0561122CCAM01416P1 | Chibia | 6 | 1416 | -4410.83 |
| CL9_523193607PSV_F0561122CCAM01416P1 | Chibia | 7 | 1416 | -4410.83 |
| CL9_523193675PSV_F0561122CCAM01416P1 | Chibia | 8 | 1416 | -4410.83 |
| CL9_523193739PSV_F0561122CCAM01416P1 | Chibia | 9 | 1416 | -4410.83 |
| CL9_523193933PSV_F0561122CCAM01416P1 | Chibia | 10 | 1416 | -4410.83 |
| CL9_523194263PSV_F0561122CCAM02416P1 | Dondo | 1 | 1416 | -4410.83 |
| CL9_523194331PSV_F0561122CCAM02416P1 | Dondo | 2 | 1416 | -4410.83 |
| CL9_523194395PSV_F0561122CCAM02416P1 | Dondo | 3 | 1416 | -4410.83 |
| CL9_523194633PSV_F0561122CCAM02416P1 | Dondo | 4 | 1416 | -4410.83 |
| CL9_523194702PSV_F0561122CCAM02416P1 | Dondo | 5 | 1416 | -4410.83 |
| CL9_523194766PSV_F0561122CCAM02416P1 | Dondo | 6 | 1416 | -4410.83 |
| CL9_523195012PSV_F0561122CCAM02416P1 | Dondo | 7 | 1416 | -4410.83 |
| CL9_523195080PSV_F0561122CCAM02416P1 | Dondo | 8 | 1416 | -4410.83 |
| CL9_523195144PSV_F0561122CCAM02416P1 | Dondo | 9 | 1416 | -4410.83 |
| CL9_523195346PSV_F0561122CCAM02416P1 | Dondo | 10 | 1416 | -4410.83 |
| CL9_523369881PSV_F0561236CCAM01418P1 | Marimba | 1 | 1418 | -4410.38 |
| CL9_523369949PSV_F0561236CCAM01418P1 | Marimba | 2 | 1418 | -4410.38 |
| CL9_523370013PSV_F0561236CCAM01418P1 | Marimba | 3 | 1418 | -4410.38 |
| CL9_523370247PSV_F0561236CCAM01418P1 | Marimba | 4 | 1418 | -4410.38 |
| CL9_523370315PSV_F0561236CCAM01418P1 | Marimba | 5 | 1418 | -4410.38 |
| CL9_523370379PSV_F0561236CCAM01418P1 | Marimba | 6 | 1418 | -4410.38 |
| CL9_523370613PSV_F0561236CCAM01418P1 | Marimba | 7 | 1418 | -4410.38 |
| CL9_523370681PSV_F0561236CCAM01418P1 | Marimba | 8 | 1418 | -4410.38 |
| CL9_523370745PSV_F0561236CCAM01418P1 | Marimba | 9 | 1418 | -4410.38 |
| CL9_523370939PSV_F0561236CCAM01418P1 | Marimba | 10 | 1418 | -4410.38 |
| CL9_523457442PSV_F0561236CCAM01419P1 | Namibe | 1 | 1419 | -4410.38 |
| CL9_523457517PSV_F0561236CCAM01419P1 | Namibe | 2 | 1419 | -4410.38 |
| CL9_523457582PSV_F0561236CCAM01419P1 | Namibe | 3 | 1419 | -4410.38 |
| CL9_523457647PSV_F0561236CCAM01419P1 | Namibe | 4 | 1419 | -4410.38 |
| CL9_523457712PSV_F0561236CCAM01419P1 | Namibe | 5 | 1419 | -4410.38 |
| CL9_523457777PSV_F0561236CCAM01419P1 | Namibe | 6 | 1419 | -4410.38 |
| CL9_523457842PSV_F0561236CCAM01419P1 | Namibe | 7 | 1419 | -4410.38 |
| CL9_523457907PSV_F0561236CCAM01419P1 | Namibe | 8 | 1419 | -4410.38 |
| CL9_523457972PSV_F0561236CCAM01419P1 | Namibe | 9 | 1419 | -4410.38 |
| CL9_523458168PSV_F0561236CCAM01419P1 | Namibe | 10 | 1419 | -4410.38 |
| CL9_523641022PSV_F0561236CCAM05421P1 | Cabinda | 1 | 1421 | -4410.38 |
| CL9_523641090PSV_F0561236CCAM05421P1 | Cabinda | 2 | 1421 | -4410.38 |
| CL9_523641154PSV_F0561236CCAM05421P1 | Cabinda | 3 | 1421 | -4410.38 |
| CL9_523641218PSV_F0561236CCAM05421P1 | Cabinda | 4 | 1421 | -4410.38 |
| CL9_523641398PSV_F0561236CCAM05421P1 | Cabinda | 5 | 1421 | -4410.38 |
| CL9_524256956PSV_F0561326CCAM01428P1 | Xangongo | 1 | 1428 | -4409.6 |
| CL9_524257031PSV_F0561326CCAM01428P1 | Xangongo | 2 | 1428 | -4409.6 |
| CL9_524257097PSV_F0561326CCAM01428P1 | Xangongo | 3 | 1428 | -4409.6 |
| CL9_524257163PSV_F0561326CCAM01428P1 | Xangongo | 4 | 1428 | -4409.6 |
| CL9_524355387PSV_F0561632CCAM01429P1 | Matala | 1 | 1429 | -4407.72 |
| CL9_524355453PSV_F0561632CCAM01429P1 | Matala | 2 | 1429 | -4407.72 |
| CL9_524355517PSV_F0561632CCAM01429P1 | Matala | 3 | 1429 | -4407.72 |
| CL9_524355750PSV_F0561632CCAM01429P1 | Matala | 4 | 1429 | -4407.72 |
| CL9_524355818PSV_F0561632CCAM01429P1 | Matala | 5 | 1429 | -4407.72 |

| | | | | |
|---|---|---|---|---|
| CL9_524355881PSV_F0561632CCAM01429P1 | Matala | 6 | 1429 | -4407.72 |
| CL9_524356182PSV_F0561632CCAM01429P1 | Matala | 8 | 1429 | -4407.72 |
| CL9_524356246PSV_F0561632CCAM01429P1 | Matala | 9 | 1429 | -4407.72 |
| CL9_524356418PSV_F0561632CCAM01429P1 | Matala | 10 | 1429 | -4407.72 |
| CL9_524356736PSV_F0561632CCAM02429P1 | Cazombo | 1 | 1429 | -4407.72 |
| CL9_524356803PSV_F0561632CCAM02429P1 | Cazombo | 2 | 1429 | -4407.72 |
| CL9_524357160PSV_F0561632CCAM02429P1 | Cazombo | 5 | 1429 | -4407.72 |
| CL9_524358198PSV_F0561632CCAM03429P1 | Ondjiva | 3 | 1429 | -4407.72 |
| CL9_524358262PSV_F0561632CCAM03429P1 | Ondjiva | 4 | 1429 | -4407.72 |
| CL9_524612360PSV_F0562034CCAM01432P1 | Longa | 1 | 1432 | -4405.41 |
| CL9_524612435PSV_F0562034CCAM01432P1 | Longa | 2 | 1432 | -4405.41 |
| CL9_524612501PSV_F0562034CCAM01432P1 | Longa | 3 | 1432 | -4405.41 |
| CL9_524612567PSV_F0562034CCAM01432P1 | Longa | 4 | 1432 | -4405.41 |
| CL9_524612771PSV_F0562034CCAM01432P1 | Longa | 5 | 1432 | -4405.41 |
| CL9_524701243PSV_F0562428CCAM01433P1 | Klein_Klipneus | 1 | 1433 | -4403.79 |
| CL9_524701312PSV_F0562428CCAM01433P1 | Klein_Klipneus | 2 | 1433 | -4403.79 |
| CL9_524701375PSV_F0562428CCAM01433P1 | Klein_Klipneus | 3 | 1433 | -4403.79 |
| CL9_524701439PSV_F0562428CCAM01433P1 | Klein_Klipneus | 4 | 1433 | -4403.79 |
| CL9_524701641PSV_F0562428CCAM01433P1 | Klein_Klipneus | 5 | 1433 | -4403.79 |
| CL9_524793410PSV_F0570000CCAM01434P1 | Cubal | 1 | 1434 | -4401.33 |
| CL9_524793485PSV_F0570000CCAM01434P1 | Cubal | 2 | 1434 | -4401.33 |
| CL9_524793551PSV_F0570000CCAM01434P1 | Cubal | 3 | 1434 | -4401.33 |
| CL9_524793653PSV_F0570000CCAM01434P1 | Cubal | 4 | 1434 | -4401.33 |
| CL9_524793719PSV_F0570000CCAM01434P1 | Cubal | 5 | 1434 | -4401.33 |
| CL9_524793785PSV_F0570000CCAM01434P1 | Cubal | 6 | 1434 | -4401.33 |
| CL9_524793886PSV_F0570000CCAM01434P1 | Cubal | 7 | 1434 | -4401.33 |
| CL9_524793952PSV_F0570000CCAM01434P1 | Cubal | 8 | 1434 | -4401.33 |
| CL9_524794134PSV_F0570000CCAM01434P1 | Cubal | 9 | 1434 | -4401.33 |
| CL9_524794462PSV_F0570000CCAM02434P1 | Soyo | 1 | 1434 | -4401.33 |
| CL9_524794537PSV_F0570000CCAM02434P1 | Soyo | 2 | 1434 | -4401.33 |
| CL9_524794669PSV_F0570000CCAM02434P1 | Soyo | 4 | 1434 | -4401.33 |
| CL9_524794970PSV_F0570000CCAM02434P1 | Soyo | 6 | 1434 | -4401.33 |
| CL9_524795036PSV_F0570000CCAM02434P1 | Soyo | 7 | 1434 | -4401.33 |
| CL9_524969851PSV_F0570462CCAM01436P1 | Conda_ccam | 1 | 1436 | -4398.71 |
| CL9_524969927PSV_F0570462CCAM01436P1 | Conda_ccam | 2 | 1436 | -4398.71 |
| CL9_524969993PSV_F0570462CCAM01436P1 | Conda_ccam | 3 | 1436 | -4398.71 |
| CL9_524970059PSV_F0570462CCAM01436P1 | Conda_ccam | 4 | 1436 | -4398.71 |
| CL9_524970307PSV_F0570462CCAM01436P1 | Conda_ccam | 5 | 1436 | -4398.71 |
| CL9_524970383PSV_F0570462CCAM01436P1 | Conda_ccam | 6 | 1436 | -4398.71 |
| CL9_524970449PSV_F0570462CCAM01436P1 | Conda_ccam | 7 | 1436 | -4398.71 |
| CL9_524970515PSV_F0570462CCAM01436P1 | Conda_ccam | 8 | 1436 | -4398.71 |
| CL9_524970581PSV_F0570462CCAM01436P1 | Conda_ccam | 9 | 1436 | -4398.71 |
| CL9_524970786PSV_F0570462CCAM01436P1 | Conda_ccam | 10 | 1436 | -4398.71 |
| CL9_524971201PSV_F0570462CCAM02436P1 | Savungo | 2 | 1436 | -4398.71 |
| CL9_524971267PSV_F0570462CCAM02436P1 | Savungo | 3 | 1436 | -4398.71 |
| CL9_524971504PSV_F0570462CCAM02436P1 | Savungo | 4 | 1436 | -4398.71 |
| CL9_524971862PSV_F0570462CCAM02436P1 | Savungo | 7 | 1436 | -4398.71 |
| CL9_524971938PSV_F0570462CCAM02436P1 | Savungo | 8 | 1436 | -4398.71 |
| CL9_524972004PSV_F0570462CCAM02436P1 | Savungo | 9 | 1436 | -4398.71 |
| CL9_525232637PSV_F0570774CCAM01439P1 | Quibala | 1 | 1439 | -4397.06 |
| CL9_525232713PSV_F0570774CCAM01439P1 | Quibala | 2 | 1439 | -4397.06 |
| CL9_525232779PSV_F0570774CCAM01439P1 | Quibala | 3 | 1439 | -4397.06 |
| CL9_525232845PSV_F0570774CCAM01439P1 | Quibala | 4 | 1439 | -4397.06 |
| CL9_525233017PSV_F0570774CCAM01439P1 | Quibala | 5 | 1439 | -4397.06 |
| CL9_525233392PSV_F0570774CCAM02439P1 | Quibaxe | 1 | 1439 | -4397.06 |
| CL9_525233468PSV_F0570774CCAM02439P1 | Quibaxe | 2 | 1439 | -4397.06 |
| CL9_525233600PSV_F0570774CCAM02439P1 | Quibaxe | 4 | 1439 | -4397.06 |
| CL9_525411481PSV_F0571020CCAM01441P1 | Viana | 1 | 1441 | -4393.77 |
| CL9_525412231PSV_F0571020CCAM02441P1 | Ukuma | 1 | 1441 | -4393.77 |

| | | | | |
|---|---|---|---|---|
| CL9_525412299PSV_F0571020CCAM02441P1 | Ukuma | 2 | 1441 | -4393.77 |
| CL9_525412363PSV_F0571020CCAM02441P1 | Ukuma | 3 | 1441 | -4393.77 |
| CL9_525412427PSV_F0571020CCAM02441P1 | Ukuma | 4 | 1441 | -4393.77 |
| CL9_525412491PSV_F0571020CCAM02441P1 | Ukuma | 5 | 1441 | -4393.77 |
| CL9_525412555PSV_F0571020CCAM02441P1 | Ukuma | 6 | 1441 | -4393.77 |
| CL9_525412619PSV_F0571020CCAM02441P1 | Ukuma | 7 | 1441 | -4393.77 |
| CL9_525412683PSV_F0571020CCAM02441P1 | Ukuma | 8 | 1441 | -4393.77 |
| CL9_525412747PSV_F0571020CCAM02441P1 | Ukuma | 9 | 1441 | -4393.77 |
| CL9_525412948PSV_F0571020CCAM02441P1 | Ukuma | 10 | 1441 | -4393.77 |
| CL9_525592189PSV_F0571020CCAM01443P1 | Viana_2 | 2 | 1443 | -4393.77 |
| CL9_525592255PSV_F0571020CCAM01443P1 | Viana_2 | 3 | 1443 | -4393.77 |
| CL9_525592504PSV_F0571020CCAM01443P1 | Viana_2 | 4 | 1443 | -4393.77 |
| CL9_525676822PSV_F0571020CCAM02443P1 | Ganda_ccam | 1 | 1444 | -4393.77 |
| CL9_525676891PSV_F0571020CCAM02443P1 | Ganda_ccam | 2 | 1444 | -4393.77 |
| CL9_525676955PSV_F0571020CCAM02443P1 | Ganda_ccam | 3 | 1444 | -4393.77 |
| CL9_525768434PSV_F0571020CCAM05443P1 | Calonda | 1 | 1445 | -4393.77 |
| CL9_525768509PSV_F0571020CCAM05443P1 | Calonda | 2 | 1445 | -4393.77 |
| CL9_525768575PSV_F0571020CCAM05443P1 | Calonda | 3 | 1445 | -4393.77 |
| CL9_525768832PSV_F0571020CCAM05443P1 | Calonda | 4 | 1445 | -4393.77 |
| CL9_525768907PSV_F0571020CCAM05443P1 | Calonda | 5 | 1445 | -4393.77 |
| CL9_525768973PSV_F0571020CCAM05443P1 | Calonda | 6 | 1445 | -4393.77 |
| CL9_525769229PSV_F0571020CCAM05443P1 | Calonda | 7 | 1445 | -4393.77 |
| CL9_525769304PSV_F0571020CCAM05443P1 | Calonda | 8 | 1445 | -4393.77 |
| CL9_526034097PSV_F0571392CCAM01448P1 | Luxilo | 1 | 1448 | -4388.82 |
| CL9_526034165PSV_F0571392CCAM01448P1 | Luxilo | 2 | 1448 | -4388.82 |
| CL9_526034229PSV_F0571392CCAM01448P1 | Luxilo | 3 | 1448 | -4388.82 |
| CL9_526034476PSV_F0571392CCAM01448P1 | Luxilo | 4 | 1448 | -4388.82 |
| CL9_526034544PSV_F0571392CCAM01448P1 | Luxilo | 5 | 1448 | -4388.82 |
| CL9_526034778PSV_F0571392CCAM01448P1 | Luxilo | 7 | 1448 | -4388.82 |
| CL9_526034842PSV_F0571392CCAM01448P1 | Luxilo | 8 | 1448 | -4388.82 |
| CL9_526034906PSV_F0571392CCAM01448P1 | Luxilo | 9 | 1448 | -4388.82 |
| CL9_526573112PSV_F0572296CCAM02454P1 | Donkerbos | 1 | 1454 | -4378.84 |
| CL9_526573181PSV_F0572296CCAM02454P1 | Donkerbos | 2 | 1454 | -4378.84 |
| CL9_526573245PSV_F0572296CCAM02454P1 | Donkerbos | 3 | 1454 | -4378.84 |
| CL9_526573309PSV_F0572296CCAM02454P1 | Donkerbos | 4 | 1454 | -4378.84 |
| CL9_526654995PSV_F0572582CCAM01455P1 | Humpata | 1 | 1455 | -4380.08 |
| CL9_526655070PSV_F0572582CCAM01455P1 | Humpata | 2 | 1455 | -4380.08 |
| CL9_526655135PSV_F0572582CCAM01455P1 | Humpata | 3 | 1455 | -4380.08 |
| CL9_526655384PSV_F0572582CCAM01455P1 | Humpata | 4 | 1455 | -4380.08 |
| CL9_526655525PSV_F0572582CCAM01455P1 | Humpata | 6 | 1455 | -4380.08 |
| CL9_526655774PSV_F0572582CCAM01455P1 | Humpata | 7 | 1455 | -4380.08 |
| CL9_526655849PSV_F0572582CCAM01455P1 | Humpata | 8 | 1455 | -4380.08 |
| CL9_526655915PSV_F0572582CCAM01455P1 | Humpata | 9 | 1455 | -4380.08 |
| CL9_526656119PSV_F0572582CCAM01455P1 | Humpata | 10 | 1455 | -4380.08 |
| CL9_526753575PSV_F0572798CCAM01456P1 | Quela_DRT_ccam | 1 | 1456 | -4379.46 |
| CL9_526753650PSV_F0572798CCAM01456P1 | Quela_DRT_ccam | 2 | 1456 | -4379.46 |
| CL9_526753716PSV_F0572798CCAM01456P1 | Quela_DRT_ccam | 3 | 1456 | -4379.46 |
| CL9_526753931PSV_F0572798CCAM01456P1 | Quela_DRT_ccam | 4 | 1456 | -4379.46 |
| CL9_526754006PSV_F0572798CCAM01456P1 | Quela_DRT_ccam | 5 | 1456 | -4379.46 |
| CL9_526754072PSV_F0572798CCAM01456P1 | Quela_DRT_ccam | 6 | 1456 | -4379.46 |
| CL9_526754287PSV_F0572798CCAM01456P1 | Quela_DRT_ccam | 7 | 1456 | -4379.46 |
| CL9_526754362PSV_F0572798CCAM01456P1 | Quela_DRT_ccam | 8 | 1456 | -4379.46 |
| CL9_526754602PSV_F0572798CCAM01456P1 | Quela_DRT_ccam | 10 | 1456 | -4379.46 |
| CL9_527013064PSV_F0572798CCAM03459P1 | Eenhana | 1 | 1459 | -4379.46 |
| CL9_527013132PSV_F0572798CCAM03459P1 | Eenhana | 2 | 1459 | -4379.46 |
| CL9_527013312PSV_F0572798CCAM03459P1 | Eenhana | 3 | 1459 | -4379.46 |
| CL9_527013385PSV_F0572798CCAM03459P1 | Eenhana | 4 | 1459 | -4379.46 |
| CL9_527013565PSV_F0572798CCAM03459P1 | Eenhana | 5 | 1459 | -4379.46 |
| CL9_527634143PSV_F0572798CCAM03466P1 | Okakarara | 1 | 1466 | -4379.46 |

| | | | | |
|---|---|---|---|---|
| CL9_527634218PSV_F0572798CCAM03466P1 | Okakarara | 2 | 1466 | -4379.46 |
| CL9_527634284PSV_F0572798CCAM03466P1 | Okakarara | 3 | 1466 | -4379.46 |
| CL9_527634510PSV_F0572798CCAM03466P1 | Okakarara | 4 | 1466 | -4379.46 |
| CL9_527634585PSV_F0572798CCAM03466P1 | Okakarara | 5 | 1466 | -4379.46 |
| CL9_527634876PSV_F0572798CCAM03466P1 | Okakarara | 7 | 1466 | -4379.46 |
| CL9_527634951PSV_F0572798CCAM03466P1 | Okakarara | 8 | 1466 | -4379.46 |
| CL9_527635017PSV_F0572798CCAM03466P1 | Okakarara | 9 | 1466 | -4379.46 |
| CL9_527635198PSV_F0572798CCAM03466P1 | Okakarara | 10 | 1466 | -4379.46 |
| CL9_527635511PSV_F0572798CCAM04466P1 | Camaxilo | 1 | 1466 | -4379.46 |
| CL9_527635652PSV_F0572798CCAM04466P1 | Camaxilo | 3 | 1466 | -4379.46 |
| CL9_527635878PSV_F0572798CCAM04466P1 | Camaxilo | 4 | 1466 | -4379.46 |
| CL9_527635953PSV_F0572798CCAM04466P1 | Camaxilo | 5 | 1466 | -4379.46 |
| CL9_527636019PSV_F0572798CCAM04466P1 | Camaxilo | 6 | 1466 | -4379.46 |
| CL9_527636250PSV_F0572798CCAM04466P1 | Camaxilo | 7 | 1466 | -4379.46 |
| CL9_527636325PSV_F0572798CCAM04466P1 | Camaxilo | 8 | 1466 | -4379.46 |
| CL9_527636390PSV_F0572798CCAM04466P1 | Camaxilo | 9 | 1466 | -4379.46 |
| CL9_527636576PSV_F0572798CCAM04466P1 | Camaxilo | 10 | 1466 | -4379.46 |
| CL9_527723678PSV_F0572798CCAM04467P1 | Sumbe | 1 | 1467 | -4379.46 |
| CL9_527723820PSV_F0572798CCAM04467P1 | Sumbe | 3 | 1467 | -4379.46 |
| CL9_527724047PSV_F0572798CCAM04467P1 | Sumbe | 4 | 1467 | -4379.46 |
| CL9_527724189PSV_F0572798CCAM04467P1 | Sumbe | 6 | 1467 | -4379.46 |
| CL9_527724416PSV_F0572798CCAM04467P1 | Sumbe | 7 | 1467 | -4379.46 |
| CL9_527724492PSV_F0572798CCAM04467P1 | Sumbe | 8 | 1467 | -4379.46 |
| CL9_527724558PSV_F0572798CCAM04467P1 | Sumbe | 9 | 1467 | -4379.46 |
| CL9_527724740PSV_F0572798CCAM04467P1 | Sumbe | 10 | 1467 | -4379.46 |
| CL9_527897099PSV_F0580000CCAM01469P1 | Cacolo | 1 | 1469 | -4375.83 |
| CL9_527897167PSV_F0580000CCAM01469P1 | Cacolo | 2 | 1469 | -4375.83 |
| CL9_527897231PSV_F0580000CCAM01469P1 | Cacolo | 3 | 1469 | -4375.83 |
| CL9_527897294PSV_F0580000CCAM01469P1 | Cacolo | 4 | 1469 | -4375.83 |
| CL9_527897358PSV_F0580000CCAM01469P1 | Cacolo | 5 | 1469 | -4375.83 |
| CL9_527897422PSV_F0580000CCAM01469P1 | Cacolo | 6 | 1469 | -4375.83 |
| CL9_527897485PSV_F0580000CCAM01469P1 | Cacolo | 7 | 1469 | -4375.83 |
| CL9_527897549PSV_F0580000CCAM01469P1 | Cacolo | 8 | 1469 | -4375.83 |
| CL9_527897613PSV_F0580000CCAM01469P1 | Cacolo | 9 | 1469 | -4375.83 |
| CL9_527897815PSV_F0580000CCAM01469P1 | Cacolo | 10 | 1469 | -4375.83 |
| CL9_527988894PSV_F0580264CCAM01470P1 | Chiagne | 2 | 1470 | -4374.38 |
| CL9_527988960PSV_F0580264CCAM01470P1 | Chiagne | 3 | 1470 | -4374.38 |
| CL9_527989283PSV_F0580264CCAM01470P1 | Chiagne | 5 | 1470 | -4374.38 |
| CL9_527989349PSV_F0580264CCAM01470P1 | Chiagne | 6 | 1470 | -4374.38 |
| CL9_527989597PSV_F0580264CCAM01470P1 | Chiagne | 7 | 1470 | -4374.38 |
| CL9_527989672PSV_F0580264CCAM01470P1 | Chiagne | 8 | 1470 | -4374.38 |
| CL9_527989738PSV_F0580264CCAM01470P1 | Chiagne | 9 | 1470 | -4374.38 |
| CL9_527990323PSV_F0580264CCAM02470P1 | Chibemba | 1 | 1470 | -4374.38 |
| CL9_527990464PSV_F0580264CCAM02470P1 | Chibemba | 3 | 1470 | -4374.38 |
| CL9_527990712PSV_F0580264CCAM02470P1 | Chibemba | 4 | 1470 | -4374.38 |
| CL9_527990787PSV_F0580264CCAM02470P1 | Chibemba | 5 | 1470 | -4374.38 |
| CL9_527990853PSV_F0580264CCAM02470P1 | Chibemba | 6 | 1470 | -4374.38 |
| CL9_527991079PSV_F0580264CCAM02470P1 | Chibemba | 7 | 1470 | -4374.38 |
| CL9_527991154PSV_F0580264CCAM02470P1 | Chibemba | 8 | 1470 | -4374.38 |
| CL9_527991220PSV_F0580264CCAM02470P1 | Chibemba | 9 | 1470 | -4374.38 |
| CL9_527991385PSV_F0580264CCAM02470P1 | Chibemba | 10 | 1470 | -4374.38 |
| CL9_527991703PSV_F0580264CCAM03470P1 | Chibanda | 1 | 1470 | -4374.38 |
| CL9_527991778PSV_F0580264CCAM03470P1 | Chibanda | 2 | 1470 | -4374.38 |
| CL9_527991844PSV_F0580264CCAM03470P1 | Chibanda | 3 | 1470 | -4374.38 |
| CL9_527992437PSV_F0580264CCAM03470P1 | Chibanda | 7 | 1470 | -4374.38 |
| CL9_527992512PSV_F0580264CCAM03470P1 | Chibanda | 8 | 1470 | -4374.38 |
| CL9_527992578PSV_F0580264CCAM03470P1 | Chibanda | 9 | 1470 | -4374.38 |
| CL9_527992760PSV_F0580264CCAM03470P1 | Chibanda | 10 | 1470 | -4374.38 |
| CL9_528253707PSV_F0580642CCAM01473P1 | Mulondo | 9 | 1473 | -4372.68 |

| | | | | |
|---|---|---|---|---|
| CL9_528254235PSV_F0580642CCAM02473P1 | Caboledo | 1 | 1473 | -4372.68 |
| CL9_528254310PSV_F0580642CCAM02473P1 | Caboledo | 2 | 1473 | -4372.68 |
| CL9_528254376PSV_F0580642CCAM02473P1 | Caboledo | 3 | 1473 | -4372.68 |
| CL9_528254762PSV_F0580642CCAM02473P1 | Caboledo | 6 | 1473 | -4372.68 |
| CL9_528254828PSV_F0580642CCAM02473P1 | Caboledo | 7 | 1473 | -4372.68 |
| CL9_528254894PSV_F0580642CCAM02473P1 | Caboledo | 8 | 1473 | -4372.68 |
| CL9_528254960PSV_F0580642CCAM02473P1 | Caboledo | 9 | 1473 | -4372.68 |
| CL9_528255156PSV_F0580642CCAM02473P1 | Caboledo | 10 | 1473 | -4372.68 |
| CL9_528345047PSV_F0580774CCAM01474P1 | Kopong_CCAM | 1 | 1474 | -4371.23 |
| CL9_528345115PSV_F0580774CCAM01474P1 | Kopong_CCAM | 2 | 1474 | -4371.23 |
| CL9_528345179PSV_F0580774CCAM01474P1 | Kopong_CCAM | 3 | 1474 | -4371.23 |
| CL9_528345413PSV_F0580774CCAM01474P1 | Kopong_CCAM | 4 | 1474 | -4371.23 |
| CL9_528345481PSV_F0580774CCAM01474P1 | Kopong_CCAM | 5 | 1474 | -4371.23 |
| CL9_528345545PSV_F0580774CCAM01474P1 | Kopong_CCAM | 6 | 1474 | -4371.23 |
| CL9_528345779PSV_F0580774CCAM01474P1 | Kopong_CCAM | 7 | 1474 | -4371.23 |
| CL9_528345847PSV_F0580774CCAM01474P1 | Kopong_CCAM | 8 | 1474 | -4371.23 |
| CL9_528345911PSV_F0580774CCAM01474P1 | Kopong_CCAM | 9 | 1474 | -4371.23 |
| CL9_528346104PSV_F0580774CCAM01474P1 | Kopong_CCAM | 10 | 1474 | -4371.23 |
| CL9_528430001PSV_F0580774CCAM01475P1 | Munhango | 1 | 1475 | -4371.23 |
| CL9_528430070PSV_F0580774CCAM01475P1 | Munhango | 2 | 1475 | -4371.23 |
| CL9_528430767PSV_F0580774CCAM02475P1 | Jwaneng_CCAM | 1 | 1475 | -4371.23 |
| CL9_528430835PSV_F0580774CCAM02475P1 | Jwaneng_CCAM | 2 | 1475 | -4371.23 |
| CL9_528431133PSV_F0580774CCAM02475P1 | Jwaneng_CCAM | 4 | 1475 | -4371.23 |
| CL9_528431202PSV_F0580774CCAM02475P1 | Jwaneng_CCAM | 5 | 1475 | -4371.23 |
| CL9_528431266PSV_F0580774CCAM02475P1 | Jwaneng_CCAM | 6 | 1475 | -4371.23 |
| CL9_528431500PSV_F0580774CCAM02475P1 | Jwaneng_CCAM | 7 | 1475 | -4371.23 |
| CL9_528431568PSV_F0580774CCAM02475P1 | Jwaneng_CCAM | 8 | 1475 | -4371.23 |
| CL9_528431633PSV_F0580774CCAM02475P1 | Jwaneng_CCAM | 9 | 1475 | -4371.23 |
| CL9_528431827PSV_F0580774CCAM02475P1 | Jwaneng_CCAM | 10 | 1475 | -4371.23 |
| CL9_528605060PSV_F0580912CCAM01477P1 | Chadibe_ccam | 4 | 1477 | -4370.07 |
| CL9_528605136PSV_F0580912CCAM01477P1 | Chadibe_ccam | 5 | 1477 | -4370.07 |
| CL9_528605202PSV_F0580912CCAM01477P1 | Chadibe_ccam | 6 | 1477 | -4370.07 |
| CL9_528605453PSV_F0580912CCAM01477P1 | Chadibe_ccam | 7 | 1477 | -4370.07 |
| CL9_528605529PSV_F0580912CCAM01477P1 | Chadibe_ccam | 8 | 1477 | -4370.07 |
| CL9_528605595PSV_F0580912CCAM01477P1 | Chadibe_ccam | 9 | 1477 | -4370.07 |
| CL9_528605774PSV_F0580912CCAM01477P1 | Chadibe_ccam | 10 | 1477 | -4370.07 |
| CL9_528606105PSV_F0580912CCAM02477P1 | Bobonong | 1 | 1477 | -4370.07 |
| CL9_528606173PSV_F0580912CCAM02477P1 | Bobonong | 2 | 1477 | -4370.07 |
| CL9_528606237PSV_F0580912CCAM02477P1 | Bobonong | 3 | 1477 | -4370.07 |
| CL9_528606473PSV_F0580912CCAM02477P1 | Bobonong | 4 | 1477 | -4370.07 |
| CL9_528606541PSV_F0580912CCAM02477P1 | Bobonong | 5 | 1477 | -4370.07 |
| CL9_528606605PSV_F0580912CCAM02477P1 | Bobonong | 6 | 1477 | -4370.07 |
| CL9_528606841PSV_F0580912CCAM02477P1 | Bobonong | 7 | 1477 | -4370.07 |
| CL9_528606909PSV_F0580912CCAM02477P1 | Bobonong | 8 | 1477 | -4370.07 |
| CL9_528606973PSV_F0580912CCAM02477P1 | Bobonong | 9 | 1477 | -4370.07 |
| CL9_528607147PSV_F0580912CCAM02477P1 | Bobonong | 10 | 1477 | -4370.07 |
| CL9_528607479PSV_F0580912CCAM03477P1 | Dukwi | 1 | 1477 | -4370.07 |
| CL9_528607547PSV_F0580912CCAM03477P1 | Dukwi | 2 | 1477 | -4370.07 |
| CL9_528607611PSV_F0580912CCAM03477P1 | Dukwi | 3 | 1477 | -4370.07 |
| CL9_528607846PSV_F0580912CCAM03477P1 | Dukwi | 4 | 1477 | -4370.07 |
| CL9_528607914PSV_F0580912CCAM03477P1 | Dukwi | 5 | 1477 | -4370.07 |
| CL9_528607978PSV_F0580912CCAM03477P1 | Dukwi | 6 | 1477 | -4370.07 |
| CL9_528608213PSV_F0580912CCAM03477P1 | Dukwi | 7 | 1477 | -4370.07 |
| CL9_528608282PSV_F0580912CCAM03477P1 | Dukwi | 8 | 1477 | -4370.07 |
| CL9_528608345PSV_F0580912CCAM03477P1 | Dukwi | 9 | 1477 | -4370.07 |
| CL9_528608539PSV_F0580912CCAM03477P1 | Dukwi | 10 | 1477 | -4370.07 |
| CL9_528697041PSV_F0580912CCAM04477P1 | Catumbela_CCAM | 1 | 1478 | -4370.07 |
| CL9_528697110PSV_F0580912CCAM04477P1 | Catumbela_CCAM | 2 | 1478 | -4370.07 |
| CL9_528697174PSV_F0580912CCAM04477P1 | Catumbela_CCAM | 3 | 1478 | -4370.07 |

| | | | | |
|---|---|---|---|---|
| CL9_528697238PSV_F0580912CCAM04477P1 | Catumbela_CCAM | 4 | 1478 | -4370.07 |
| CL9_528697474PSV_F0580912CCAM04477P1 | Catumbela_CCAM | 5 | 1478 | -4370.07 |
| CL9_528697542PSV_F0580912CCAM04477P1 | Catumbela_CCAM | 6 | 1478 | -4370.07 |
| CL9_528697606PSV_F0580912CCAM04477P1 | Catumbela_CCAM | 7 | 1478 | -4370.07 |
| CL9_528697670PSV_F0580912CCAM04477P1 | Catumbela_CCAM | 8 | 1478 | -4370.07 |
| CL9_528697734PSV_F0580912CCAM04477P1 | Catumbela_CCAM | 9 | 1478 | -4370.07 |
| CL9_528698261PSV_F0580912CCAM05477P1 | Francistown | 1 | 1478 | -4370.07 |
| CL9_528698393PSV_F0580912CCAM05477P1 | Francistown | 3 | 1478 | -4370.07 |
| CL9_528698457PSV_F0580912CCAM05477P1 | Francistown | 4 | 1478 | -4370.07 |
| CL9_528698651PSV_F0580912CCAM05477P1 | Francistown | 5 | 1478 | -4370.07 |
| CL9_528879674PSV_F0581002CCAM02480P1 | Caconda | 1 | 1480 | -4368.81 |
| CL9_528879742PSV_F0581002CCAM02480P1 | Caconda | 2 | 1480 | -4368.81 |
| CL9_528879806PSV_F0581002CCAM02480P1 | Caconda | 3 | 1480 | -4368.81 |
| CL9_528879870PSV_F0581002CCAM02480P1 | Caconda | 4 | 1480 | -4368.81 |
| CL9_528879935PSV_F0581002CCAM02480P1 | Caconda | 5 | 1480 | -4368.81 |
| CL9_528879998PSV_F0581002CCAM02480P1 | Caconda | 6 | 1480 | -4368.81 |
| CL9_528880062PSV_F0581002CCAM02480P1 | Caconda | 7 | 1480 | -4368.81 |
| CL9_528880126PSV_F0581002CCAM02480P1 | Caconda | 8 | 1480 | -4368.81 |
| CL9_528880190PSV_F0581002CCAM02480P1 | Caconda | 9 | 1480 | -4368.81 |
| CL9_528880371PSV_F0581002CCAM02480P1 | Caconda | 10 | 1480 | -4368.81 |
| CL9_529055144PSV_F0581248CCAM01482P1 | Coutada | 2 | 1482 | -4367.02 |
| CL9_529055210PSV_F0581248CCAM01482P1 | Coutada | 3 | 1482 | -4367.02 |
| CL9_529055463PSV_F0581248CCAM01482P1 | Coutada | 4 | 1482 | -4367.02 |
| CL9_529055538PSV_F0581248CCAM01482P1 | Coutada | 5 | 1482 | -4367.02 |
| CL9_529055852PSV_F0581248CCAM01482P1 | Coutada | 7 | 1482 | -4367.02 |
| CL9_529055927PSV_F0581248CCAM01482P1 | Coutada | 8 | 1482 | -4367.02 |
| CL9_529055993PSV_F0581248CCAM01482P1 | Coutada | 9 | 1482 | -4367.02 |
| CL9_529056197PSV_F0581248CCAM01482P1 | Coutada | 10 | 1482 | -4367.02 |
| CL9_529056559PSV_F0581248CCAM02482P1 | Cuangar | 1 | 1482 | -4367.02 |
| CL9_529056634PSV_F0581248CCAM02482P1 | Cuangar | 2 | 1482 | -4367.02 |
| CL9_529056700PSV_F0581248CCAM02482P1 | Cuangar | 3 | 1482 | -4367.02 |
| CL9_529056766PSV_F0581248CCAM02482P1 | Cuangar | 4 | 1482 | -4367.02 |
| CL9_529056951PSV_F0581248CCAM02482P1 | Cuangar | 5 | 1482 | -4367.02 |
| CL9_529318461PSV_F0581572CCAM01484P1 | Serowe_CCAM | 1 | 1485 | -4364.32 |
| CL9_529318536PSV_F0581572CCAM01484P1 | Serowe_CCAM | 2 | 1485 | -4364.32 |
| CL9_529318602PSV_F0581572CCAM01484P1 | Serowe_CCAM | 3 | 1485 | -4364.32 |
| CL9_529318668PSV_F0581572CCAM01484P1 | Serowe_CCAM | 4 | 1485 | -4364.32 |
| CL9_529318872PSV_F0581572CCAM01484P1 | Serowe_CCAM | 5 | 1485 | -4364.32 |
| CL9_529319234PSV_F0581572CCAM02484P1 | Tobane_CCAM | 1 | 1485 | -4364.32 |
| CL9_529319303PSV_F0581572CCAM02484P1 | Tobane_CCAM | 2 | 1485 | -4364.32 |
| CL9_529319367PSV_F0581572CCAM02484P1 | Tobane_CCAM | 3 | 1485 | -4364.32 |
| CL9_529319431PSV_F0581572CCAM02484P1 | Tobane_CCAM | 4 | 1485 | -4364.32 |
| CL9_529319633PSV_F0581572CCAM02484P1 | Tobane_CCAM | 5 | 1485 | -4364.32 |
| CL9_529495402PSV_F0581836CCAM03487P1 | Shoshong | 1 | 1487 | -4361.29 |
| CL9_529495478PSV_F0581836CCAM03487P1 | Shoshong | 2 | 1487 | -4361.29 |
| CL9_529495941PSV_F0581836CCAM03487P1 | Shoshong | 6 | 1487 | -4361.29 |
| CL9_529496913PSV_F0581836CCAM04487P1 | Molepolole | 2 | 1487 | -4361.29 |
| CL9_529496977PSV_F0581836CCAM04487P1 | Molepolole | 3 | 1487 | -4361.29 |
| CL9_529497041PSV_F0581836CCAM04487P1 | Molepolole | 4 | 1487 | -4361.29 |
| CL9_529497243PSV_F0581836CCAM04487P1 | Molepolole | 5 | 1487 | -4361.29 |
| CL9_529672268PSV_F0581986CCAM01489P1 | Sangwali | 1 | 1489 | -4360.54 |
| CL9_529672343PSV_F0581986CCAM01489P1 | Sangwali | 2 | 1489 | -4360.54 |
| CL9_529672410PSV_F0581986CCAM01489P1 | Sangwali | 3 | 1489 | -4360.54 |
| CL9_529672657PSV_F0581986CCAM01489P1 | Sangwali | 4 | 1489 | -4360.54 |
| CL9_529672732PSV_F0581986CCAM01489P1 | Sangwali | 5 | 1489 | -4360.54 |
| CL9_529672797PSV_F0581986CCAM01489P1 | Sangwali | 6 | 1489 | -4360.54 |
| CL9_529673045PSV_F0581986CCAM01489P1 | Sangwali | 7 | 1489 | -4360.54 |
| CL9_529673120PSV_F0581986CCAM01489P1 | Sangwali | 8 | 1489 | -4360.54 |
| CL9_529673186PSV_F0581986CCAM01489P1 | Sangwali | 9 | 1489 | -4360.54 |

| | | | | |
|---|---|---|---|---|
| CL9_529673390PSV_F0581986CCAM01489P1 | Sangwali | 10 | 1489 | -4360.54 |
| CL9_529673774PSV_F0581986CCAM02489P1 | Orapa | 1 | 1489 | -4360.54 |
| CL9_529673849PSV_F0581986CCAM02489P1 | Orapa | 2 | 1489 | -4360.54 |
| CL9_529673915PSV_F0581986CCAM02489P1 | Orapa | 3 | 1489 | -4360.54 |
| CL9_529673980PSV_F0581986CCAM02489P1 | Orapa | 4 | 1489 | -4360.54 |
| CL9_529674230PSV_F0581986CCAM02489P1 | Orapa | 5 | 1489 | -4360.54 |
| CL9_529674305PSV_F0581986CCAM02489P1 | Orapa | 6 | 1489 | -4360.54 |
| CL9_529674370PSV_F0581986CCAM02489P1 | Orapa | 7 | 1489 | -4360.54 |
| CL9_529674435PSV_F0581986CCAM02489P1 | Orapa | 8 | 1489 | -4360.54 |
| CL9_529674500PSV_F0581986CCAM02489P1 | Orapa | 9 | 1489 | -4360.54 |
| CL9_529674704PSV_F0581986CCAM02489P1 | Orapa | 10 | 1489 | -4360.54 |
| CL9_529675055PSV_F0581986CCAM03489P1 | Katima_Mulilo | 1 | 1489 | -4360.54 |
| CL9_529675129PSV_F0581986CCAM03489P1 | Katima_Mulilo | 2 | 1489 | -4360.54 |
| CL9_529675193PSV_F0581986CCAM03489P1 | Katima_Mulilo | 3 | 1489 | -4360.54 |
| CL9_529675440PSV_F0581986CCAM03489P1 | Katima_Mulilo | 4 | 1489 | -4360.54 |
| CL9_529675572PSV_F0581986CCAM03489P1 | Katima_Mulilo | 6 | 1489 | -4360.54 |
| CL9_529675819PSV_F0581986CCAM03489P1 | Katima_Mulilo | 7 | 1489 | -4360.54 |
| CL9_529675887PSV_F0581986CCAM03489P1 | Katima_Mulilo | 8 | 1489 | -4360.54 |
| CL9_529675951PSV_F0581986CCAM03489P1 | Katima_Mulilo | 9 | 1489 | -4360.54 |
| CL9_529944445PSV_F0582046CCAM02491P1 | Sebina_ccam | 1 | 1492 | -4360.75 |
| CL9_529944520PSV_F0582046CCAM02491P1 | Sebina_ccam | 2 | 1492 | -4360.75 |
| CL9_529944585PSV_F0582046CCAM02491P1 | Sebina_ccam | 3 | 1492 | -4360.75 |
| CL9_529944802PSV_F0582046CCAM02491P1 | Sebina_ccam | 4 | 1492 | -4360.75 |
| CL9_529944868PSV_F0582046CCAM02491P1 | Sebina_ccam | 5 | 1492 | -4360.75 |
| CL9_529944933PSV_F0582046CCAM02491P1 | Sebina_ccam | 6 | 1492 | -4360.75 |
| CL9_529945133PSV_F0582046CCAM02491P1 | Sebina_ccam | 7 | 1492 | -4360.75 |
| CL9_529945199PSV_F0582046CCAM02491P1 | Sebina_ccam | 8 | 1492 | -4360.75 |
| CL9_529945385PSV_F0582046CCAM02491P1 | Sebina_ccam | 9 | 1492 | -4360.75 |
| CL9_529946427PSV_F0582046CCAM04491P1 | Mussende | 1 | 1492 | -4360.75 |
| CL9_529946502PSV_F0582046CCAM04491P1 | Mussende | 2 | 1492 | -4360.75 |
| CL9_529946567PSV_F0582046CCAM04491P1 | Mussende | 3 | 1492 | -4360.75 |
| CL9_529946632PSV_F0582046CCAM04491P1 | Mussende | 4 | 1492 | -4360.75 |
| CL9_529946806PSV_F0582046CCAM04491P1 | Mussende | 5 | 1492 | -4360.75 |
| CL9_530028821PSV_F0582046CCAM02493P1 | Mavinga | 1 | 1493 | -4360.75 |
| CL9_530028897PSV_F0582046CCAM02493P1 | Mavinga | 2 | 1493 | -4360.75 |
| CL9_530028963PSV_F0582046CCAM02493P1 | Mavinga | 3 | 1493 | -4360.75 |
| CL9_530029201PSV_F0582046CCAM02493P1 | Mavinga | 4 | 1493 | -4360.75 |
| CL9_530029277PSV_F0582046CCAM02493P1 | Mavinga | 5 | 1493 | -4360.75 |
| CL9_530029343PSV_F0582046CCAM02493P1 | Mavinga | 6 | 1493 | -4360.75 |
| CL9_530029657PSV_F0582046CCAM02493P1 | Mavinga | 8 | 1493 | -4360.75 |
| CL9_530029887PSV_F0582046CCAM02493P1 | Mavinga | 10 | 1493 | -4360.75 |
| CL9_530386186PSV_F0582046CCAM01497P1 | Okambonde | 1 | 1497 | -4360.75 |
| CL9_530386262PSV_F0582046CCAM01497P1 | Okambonde | 2 | 1497 | -4360.75 |
| CL9_530386328PSV_F0582046CCAM01497P1 | Okambonde | 3 | 1497 | -4360.75 |
| CL9_530386394PSV_F0582046CCAM01497P1 | Okambonde | 4 | 1497 | -4360.75 |
| CL9_530386603PSV_F0582046CCAM01497P1 | Okambonde | 5 | 1497 | -4360.75 |
| CL9_530387358PSV_F0582046CCAM02497P1 | Nokaneng | 5 | 1497 | -4360.75 |
| CL9_530484920PSV_F0582046CCAM07497P1 | Coemba | 1 | 1498 | -4360.75 |
| CL9_530484993PSV_F0582046CCAM07497P1 | Coemba | 2 | 1498 | -4360.75 |
| CL9_530485059PSV_F0582046CCAM07497P1 | Coemba | 3 | 1498 | -4360.75 |
| CL9_530485274PSV_F0582046CCAM07497P1 | Coemba | 4 | 1498 | -4360.75 |
| CL9_530485350PSV_F0582046CCAM07497P1 | Coemba | 5 | 1498 | -4360.75 |
| CL9_530485416PSV_F0582046CCAM07497P1 | Coemba | 6 | 1498 | -4360.75 |
| CL9_530485631PSV_F0582046CCAM07497P1 | Coemba | 7 | 1498 | -4360.75 |
| CL9_530485707PSV_F0582046CCAM07497P1 | Coemba | 8 | 1498 | -4360.75 |
| CL9_530485773PSV_F0582046CCAM07497P1 | Coemba | 9 | 1498 | -4360.75 |
| CL9_530485947PSV_F0582046CCAM07497P1 | Coemba | 10 | 1498 | -4360.75 |
| CL9_530486283PSV_F0582046CCAM08497P1 | Luma_Cassao | 1 | 1498 | -4360.75 |
| CL9_530486358PSV_F0582046CCAM08497P1 | Luma_Cassao | 2 | 1498 | -4360.75 |

| | | | | |
|---|---|---|---|---|
| CL9_530486424PSV_F0582046CCAM08497P1 | Luma_Cassao | 3 | 1498 | -4360.75 |
| CL9_530486490PSV_F0582046CCAM08497P1 | Luma_Cassao | 4 | 1498 | -4360.75 |
| CL9_530486663PSV_F0582046CCAM08497P1 | Luma_Cassao | 5 | 1498 | -4360.75 |
| CL9_530560998PSV_F0582046CCAM09497P1 | Nokaneng_2 | 1 | 1499 | -4360.75 |
| CL9_530561073PSV_F0582046CCAM09497P1 | Nokaneng_2 | 2 | 1499 | -4360.75 |
| CL9_530561139PSV_F0582046CCAM09497P1 | Nokaneng_2 | 3 | 1499 | -4360.75 |
| CL9_530561401PSV_F0582046CCAM09497P1 | Nokaneng_2 | 5 | 1499 | -4360.75 |
| CL9_530650554PSV_F0582136CCAM01500P1 | Ranaka | 1 | 1500 | -4359.84 |
| CL9_530650623PSV_F0582136CCAM01500P1 | Ranaka | 2 | 1500 | -4359.84 |
| CL9_530650687PSV_F0582136CCAM01500P1 | Ranaka | 3 | 1500 | -4359.84 |
| CL9_530651010PSV_F0582136CCAM01500P1 | Ranaka | 5 | 1500 | -4359.84 |
| CL9_530651074PSV_F0582136CCAM01500P1 | Ranaka | 6 | 1500 | -4359.84 |
| CL9_530651397PSV_F0582136CCAM01500P1 | Ranaka | 8 | 1500 | -4359.84 |
| CL9_530651461PSV_F0582136CCAM01500P1 | Ranaka | 9 | 1500 | -4359.84 |
| CL9_530651631PSV_F0582136CCAM01500P1 | Ranaka | 10 | 1500 | -4359.84 |
| CL9_530651964PSV_F0582136CCAM02500P1 | Seleka | 1 | 1500 | -4359.84 |
| CL9_530652039PSV_F0582136CCAM02500P1 | Seleka | 2 | 1500 | -4359.84 |
| CL9_530652104PSV_F0582136CCAM02500P1 | Seleka | 3 | 1500 | -4359.84 |
| CL9_530652342PSV_F0582136CCAM02500P1 | Seleka | 4 | 1500 | -4359.84 |
| CL9_530652414PSV_F0582136CCAM02500P1 | Seleka | 5 | 1500 | -4359.84 |
| CL9_530652480PSV_F0582136CCAM02500P1 | Seleka | 6 | 1500 | -4359.84 |
| CL9_530652720PSV_F0582136CCAM02500P1 | Seleka | 7 | 1500 | -4359.84 |
| CL9_530652792PSV_F0582136CCAM02500P1 | Seleka | 8 | 1500 | -4359.84 |
| CL9_530652996PSV_F0582136CCAM02500P1 | Seleka | 9 | 1500 | -4359.84 |
| CL9_530737404PSV_F0582394CCAM01501P1 | The_Bubbles | 1 | 1501 | -4358.73 |
| CL9_530737545PSV_F0582394CCAM01501P1 | The_Bubbles | 3 | 1501 | -4358.73 |
| CL9_530737866PSV_F0582394CCAM01501P1 | The_Bubbles | 5 | 1501 | -4358.73 |
| CL9_530737932PSV_F0582394CCAM01501P1 | The_Bubbles | 6 | 1501 | -4358.73 |
| CL9_530738169PSV_F0582394CCAM01501P1 | The_Bubbles | 7 | 1501 | -4358.73 |
| CL9_530738245PSV_F0582394CCAM01501P1 | The_Bubbles | 8 | 1501 | -4358.73 |
| CL9_530738312PSV_F0582394CCAM01501P1 | The_Bubbles | 9 | 1501 | -4358.73 |
| CL9_530738508PSV_F0582394CCAM01501P1 | The_Bubbles | 10 | 1501 | -4358.73 |
| CL9_530738859PSV_F0582394CCAM02501P1 | The_Bowl | 1 | 1501 | -4358.73 |
| CL9_530738934PSV_F0582394CCAM02501P1 | The_Bowl | 2 | 1501 | -4358.73 |
| CL9_530739000PSV_F0582394CCAM02501P1 | The_Bowl | 3 | 1501 | -4358.73 |
| CL9_530739066PSV_F0582394CCAM02501P1 | The_Bowl | 4 | 1501 | -4358.73 |
| CL9_530739261PSV_F0582394CCAM02501P1 | The_Bowl | 5 | 1501 | -4358.73 |
| CL9_530825450PSV_F0582760CCAM01502P1 | St_Sauveur | 1 | 1502 | -4354.93 |
| CL9_530825970PSV_F0582760CCAM01502P1 | St_Sauveur | 6 | 1502 | -4354.93 |
| CL9_530826544PSV_F0582760CCAM01502P1 | St_Sauveur | 10 | 1502 | -4354.93 |
| CL9_530826887PSV_F0582760CCAM02502P1 | The_Tarn | 1 | 1502 | -4354.93 |
| CL9_530826964PSV_F0582760CCAM02502P1 | The_Tarn | 2 | 1502 | -4354.93 |
| CL9_530827029PSV_F0582760CCAM02502P1 | The_Tarn | 3 | 1502 | -4354.93 |
| CL9_530827095PSV_F0582760CCAM02502P1 | The_Tarn | 4 | 1502 | -4354.93 |
| CL9_530827353PSV_F0582760CCAM02502P1 | The_Tarn | 5 | 1502 | -4354.93 |
| CL9_530827428PSV_F0582760CCAM02502P1 | The_Tarn | 6 | 1502 | -4354.93 |
| CL9_530827494PSV_F0582760CCAM02502P1 | The_Tarn | 7 | 1502 | -4354.93 |
| CL9_530827561PSV_F0582760CCAM02502P1 | The_Tarn | 8 | 1502 | -4354.93 |
| CL9_530827627PSV_F0582760CCAM02502P1 | The_Tarn | 9 | 1502 | -4354.93 |
| CL9_530827822PSV_F0582760CCAM02502P1 | The_Tarn | 10 | 1502 | -4354.93 |
| CL9_530914713PSV_F0582946CCAM01503P1 | Somesville | 1 | 1503 | -4353.81 |
| CL9_530914789PSV_F0582946CCAM01503P1 | Somesville | 2 | 1503 | -4353.81 |
| CL9_530915103PSV_F0582946CCAM01503P1 | Somesville | 4 | 1503 | -4353.81 |
| CL9_530915179PSV_F0582946CCAM01503P1 | Somesville | 5 | 1503 | -4353.81 |
| CL9_530915245PSV_F0582946CCAM01503P1 | Somesville | 6 | 1503 | -4353.81 |
| CL9_530915485PSV_F0582946CCAM01503P1 | Somesville | 7 | 1503 | -4353.81 |
| CL9_530915561PSV_F0582946CCAM01503P1 | Somesville | 8 | 1503 | -4353.81 |
| CL9_530915823PSV_F0582946CCAM01503P1 | Somesville | 10 | 1503 | -4353.81 |
| CL9_531019416PSV_F0590000CCAM01504P1 | Cedar_Swamp | 10 | 1504 | -4351.81 |

| | | | | |
|---|---|---|---|---|
| CL9_531020611PSV_F0590000CCAM02504P1 | Cadillac_Mountain | 10 | 1504 | -4351.81 |
| CL9_531100725PSV_F0590000CCAM06504P1 | Upper_Hadlock | 1 | 1505 | -4351.81 |
| CL9_531100793PSV_F0590000CCAM06504P1 | Upper_Hadlock | 2 | 1505 | -4351.81 |
| CL9_531101507PSV_F0590000CCAM06504P1 | Upper_Hadlock | 8 | 1505 | -4351.81 |
| CL9_531101571PSV_F0590000CCAM06504P1 | Upper_Hadlock | 9 | 1505 | -4351.81 |
| CL9_531101751PSV_F0590000CCAM06504P1 | Upper_Hadlock | 10 | 1505 | -4351.81 |
| CL9_531272441PSV_F0590372CCAM01507P1 | Witch_Hole_Pond | 1 | 1507 | -4348.61 |
| CL9_531272516PSV_F0590372CCAM01507P1 | Witch_Hole_Pond | 2 | 1507 | -4348.61 |
| CL9_531272582PSV_F0590372CCAM01507P1 | Witch_Hole_Pond | 3 | 1507 | -4348.61 |
| CL9_531272830PSV_F0590372CCAM01507P1 | Witch_Hole_Pond | 4 | 1507 | -4348.61 |
| CL9_531272905PSV_F0590372CCAM01507P1 | Witch_Hole_Pond | 5 | 1507 | -4348.61 |
| CL9_531272971PSV_F0590372CCAM01507P1 | Witch_Hole_Pond | 6 | 1507 | -4348.61 |
| CL9_531273219PSV_F0590372CCAM01507P1 | Witch_Hole_Pond | 7 | 1507 | -4348.61 |
| CL9_531273294PSV_F0590372CCAM01507P1 | Witch_Hole_Pond | 8 | 1507 | -4348.61 |
| CL9_531273360PSV_F0590372CCAM01507P1 | Witch_Hole_Pond | 9 | 1507 | -4348.61 |
| CL9_531273564PSV_F0590372CCAM01507P1 | Witch_Hole_Pond | 10 | 1507 | -4348.61 |
| CL9_531361711PSV_F0590612CCAM01508P1 | Ingraham_Point | 1 | 1508 | -4349.51 |
| CL9_531361780PSV_F0590612CCAM01508P1 | Ingraham_Point | 2 | 1508 | -4349.51 |
| CL9_531361844PSV_F0590612CCAM01508P1 | Ingraham_Point | 3 | 1508 | -4349.51 |
| CL9_531361908PSV_F0590612CCAM01508P1 | Ingraham_Point | 4 | 1508 | -4349.51 |
| CL9_531362102PSV_F0590612CCAM01508P1 | Ingraham_Point | 5 | 1508 | -4349.51 |
| CL9_531454092PSV_F0590936CCAM04509P1 | Rum_Island | 1 | 1509 | -4350.97 |
| CL9_531454157PSV_F0590936CCAM04509P1 | Rum_Island | 2 | 1509 | -4350.97 |
| CL9_531454221PSV_F0590936CCAM04509P1 | Rum_Island | 3 | 1509 | -4350.97 |
| CL9_531454445PSV_F0590936CCAM04509P1 | Rum_Island | 4 | 1509 | -4350.97 |
| CL9_531454513PSV_F0590936CCAM04509P1 | Rum_Island | 5 | 1509 | -4350.97 |
| CL9_531625541PSV_F0591260CCAM01511P1 | Pemetic | 1 | 1511 | -4349.78 |
| CL9_531625609PSV_F0591260CCAM01511P1 | Pemetic | 2 | 1511 | -4349.78 |
| CL9_531625673PSV_F0591260CCAM01511P1 | Pemetic | 3 | 1511 | -4349.78 |
| CL9_531625737PSV_F0591260CCAM01511P1 | Pemetic | 4 | 1511 | -4349.78 |
| CL9_531625907PSV_F0591260CCAM01511P1 | Pemetic | 5 | 1511 | -4349.78 |
| CL9_531626275PSV_F0591260CCAM02511P1 | Jordan_Pond | 1 | 1511 | -4349.78 |
| CL9_531626343PSV_F0591260CCAM02511P1 | Jordan_Pond | 2 | 1511 | -4349.78 |
| CL9_531626407PSV_F0591260CCAM02511P1 | Jordan_Pond | 3 | 1511 | -4349.78 |
| CL9_531626471PSV_F0591260CCAM02511P1 | Jordan_Pond | 4 | 1511 | -4349.78 |
| CL9_531626673PSV_F0591260CCAM02511P1 | Jordan_Pond | 5 | 1511 | -4349.78 |
| CL9_531627007PSV_F0591260CCAM03511P1 | Penobscot_ccam | 1 | 1511 | -4349.78 |
| CL9_531627075PSV_F0591260CCAM03511P1 | Penobscot_ccam | 2 | 1511 | -4349.78 |
| CL9_531627139PSV_F0591260CCAM03511P1 | Penobscot_ccam | 3 | 1511 | -4349.78 |
| CL9_531627385PSV_F0591260CCAM03511P1 | Penobscot_ccam | 4 | 1511 | -4349.78 |
| CL9_531627454PSV_F0591260CCAM03511P1 | Penobscot_ccam | 5 | 1511 | -4349.78 |
| CL9_531627518PSV_F0591260CCAM03511P1 | Penobscot_ccam | 6 | 1511 | -4349.78 |
| CL9_531627764PSV_F0591260CCAM03511P1 | Penobscot_ccam | 7 | 1511 | -4349.78 |
| CL9_531627832PSV_F0591260CCAM03511P1 | Penobscot_ccam | 8 | 1511 | -4349.78 |
| CL9_531627896PSV_F0591260CCAM03511P1 | Penobscot_ccam | 9 | 1511 | -4349.78 |
| CL9_531628098PSV_F0591260CCAM03511P1 | Penobscot_ccam | 10 | 1511 | -4349.78 |
| CL9_531897203PSV_F0591596CCAM01514P1 | Asticou | 1 | 1514 | -4346.46 |
| CL9_531897278PSV_F0591596CCAM01514P1 | Asticou | 2 | 1514 | -4346.46 |
| CL9_531897344PSV_F0591596CCAM01514P1 | Asticou | 3 | 1514 | -4346.46 |
| CL9_531897410PSV_F0591596CCAM01514P1 | Asticou | 4 | 1514 | -4346.46 |
| CL9_531897619PSV_F0591596CCAM01514P1 | Asticou | 5 | 1514 | -4346.46 |
| CL9_531897989PSV_F0591596CCAM02514P1 | Bass_Harbor_Head | 1 | 1514 | -4346.46 |
| CL9_531898057PSV_F0591596CCAM02514P1 | Bass_Harbor_Head | 2 | 1514 | -4346.46 |
| CL9_531898121PSV_F0591596CCAM02514P1 | Bass_Harbor_Head | 3 | 1514 | -4346.46 |
| CL9_531898185PSV_F0591596CCAM02514P1 | Bass_Harbor_Head | 4 | 1514 | -4346.46 |
| CL9_531898431PSV_F0591596CCAM02514P1 | Bass_Harbor_Head | 5 | 1514 | -4346.46 |
| CL9_531898499PSV_F0591596CCAM02514P1 | Bass_Harbor_Head | 6 | 1514 | -4346.46 |
| CL9_531898563PSV_F0591596CCAM02514P1 | Bass_Harbor_Head | 7 | 1514 | -4346.46 |
| CL9_531898627PSV_F0591596CCAM02514P1 | Bass_Harbor_Head | 8 | 1514 | -4346.46 |

| | | | | |
|---|---|---|---|---|
| CL9_531898691PSV_F0591596CCAM02514P1 | Bass_Harbor_Head | 9 | 1514 | -4346.46 |
| CL9_531898937PSV_F0591596CCAM02514P1 | Bass_Harbor_Head | 10 | 1514 | -4346.46 |
| CL9_531899005PSV_F0591596CCAM02514P1 | Bass_Harbor_Head | 11 | 1514 | -4346.46 |
| CL9_531899069PSV_F0591596CCAM02514P1 | Bass_Harbor_Head | 12 | 1514 | -4346.46 |
| CL9_531899133PSV_F0591596CCAM02514P1 | Bass_Harbor_Head | 13 | 1514 | -4346.46 |
| CL9_531899197PSV_F0591596CCAM02514P1 | Bass_Harbor_Head | 14 | 1514 | -4346.46 |
| CL9_531899421PSV_F0591596CCAM02514P1 | Bass_Harbor_Head | 15 | 1514 | -4346.46 |
| CL9_531899489PSV_F0591596CCAM02514P1 | Bass_Harbor_Head | 16 | 1514 | -4346.46 |
| CL9_531899553PSV_F0591596CCAM02514P1 | Bass_Harbor_Head | 17 | 1514 | -4346.46 |
| CL9_531899617PSV_F0591596CCAM02514P1 | Bass_Harbor_Head | 18 | 1514 | -4346.46 |
| CL9_532069513PSV_F0591998CCAM01516P1 | Ship_Island | 1 | 1516 | -4343.37 |
| CL9_532069588PSV_F0591998CCAM01516P1 | Ship_Island | 2 | 1516 | -4343.37 |
| CL9_532069654PSV_F0591998CCAM01516P1 | Ship_Island | 3 | 1516 | -4343.37 |
| CL9_532069886PSV_F0591998CCAM01516P1 | Ship_Island | 6 | 1516 | -4343.37 |
| CL9_532069952PSV_F0591998CCAM01516P1 | Ship_Island | 7 | 1516 | -4343.37 |
| CL9_532070264PSV_F0591998CCAM01516P1 | Ship_Island | 9 | 1516 | -4343.37 |
| CL9_532070561PSV_F0591998CCAM01516P1 | Ship_Island | 11 | 1516 | -4343.37 |
| CL9_532070629PSV_F0591998CCAM01516P1 | Ship_Island | 12 | 1516 | -4343.37 |
| CL9_532070695PSV_F0591998CCAM01516P1 | Ship_Island | 13 | 1516 | -4343.37 |
| CL9_532070761PSV_F0591998CCAM01516P1 | Ship_Island | 14 | 1516 | -4343.37 |
| CL9_532070827PSV_F0591998CCAM01516P1 | Ship_Island | 15 | 1516 | -4343.37 |
| CL9_532070928PSV_F0591998CCAM01516P1 | Ship_Island | 16 | 1516 | -4343.37 |
| CL9_532070994PSV_F0591998CCAM01516P1 | Ship_Island | 17 | 1516 | -4343.37 |
| CL9_532071231PSV_F0591998CCAM01516P1 | Ship_Island | 18 | 1516 | -4343.37 |
| CL9_532071306PSV_F0591998CCAM01516P1 | Ship_Island | 19 | 1516 | -4343.37 |
| CL9_532071372PSV_F0591998CCAM01516P1 | Ship_Island | 20 | 1516 | -4343.37 |
| CL9_532071692PSV_F0591998CCAM01516P1 | Ship_Island | 22 | 1516 | -4343.37 |
| CL9_532071758PSV_F0591998CCAM01516P1 | Ship_Island | 23 | 1516 | -4343.37 |
| CL9_532071824PSV_F0591998CCAM01516P1 | Ship_Island | 24 | 1516 | -4343.37 |
| CL9_532072032PSV_F0591998CCAM01516P1 | Ship_Island | 25 | 1516 | -4343.37 |
| CL9_532072370PSV_F0591998CCAM02516P1 | Stave_Island | 1 | 1516 | -4343.37 |
| CL9_532072446PSV_F0591998CCAM02516P1 | Stave_Island | 2 | 1516 | -4343.37 |
| CL9_532072512PSV_F0591998CCAM02516P1 | Stave_Island | 3 | 1516 | -4343.37 |
| CL9_532072579PSV_F0591998CCAM02516P1 | Stave_Island | 4 | 1516 | -4343.37 |
| CL9_532072774PSV_F0591998CCAM02516P1 | Stave_Island | 5 | 1516 | -4343.37 |
| CL9_532246528PSV_F0592242CCAM01518P1 | Folly_Island_ccam | 1 | 1518 | -4341.62 |
| CL9_532246597PSV_F0592242CCAM01518P1 | Folly_Island_ccam | 2 | 1518 | -4341.62 |
| CL9_532246661PSV_F0592242CCAM01518P1 | Folly_Island_ccam | 3 | 1518 | -4341.62 |
| CL9_532246726PSV_F0592242CCAM01518P1 | Folly_Island_ccam | 4 | 1518 | -4341.62 |
| CL9_532246967PSV_F0592242CCAM01518P1 | Folly_Island_ccam | 5 | 1518 | -4341.62 |
| CL9_532247036PSV_F0592242CCAM01518P1 | Folly_Island_ccam | 6 | 1518 | -4341.62 |
| CL9_532247099PSV_F0592242CCAM01518P1 | Folly_Island_ccam | 7 | 1518 | -4341.62 |
| CL9_532247163PSV_F0592242CCAM01518P1 | Folly_Island_ccam | 8 | 1518 | -4341.62 |
| CL9_532247227PSV_F0592242CCAM01518P1 | Folly_Island_ccam | 9 | 1518 | -4341.62 |
| CL9_532247421PSV_F0592242CCAM01518P1 | Folly_Island_ccam | 10 | 1518 | -4341.62 |
| CL9_532251051PSV_F0592242CCAM04518P1 | Old_Whale_Ledge | 5 | 1518 | -4341.62 |
| CL9_532251119PSV_F0592242CCAM04518P1 | Old_Whale_Ledge | 6 | 1518 | -4341.62 |
| CL9_532251182PSV_F0592242CCAM04518P1 | Old_Whale_Ledge | 7 | 1518 | -4341.62 |
| CL9_532251245PSV_F0592242CCAM04518P1 | Old_Whale_Ledge | 8 | 1518 | -4341.62 |
| CL9_532251615PSV_F0592242CCAM04518P1 | Old_Whale_Ledge | 10 | 1518 | -4341.62 |
| CL9_532517400PSV_F0592578CCAM01521P1 | Daggett | 1 | 1521 | -4337.92 |
| CL9_532517476PSV_F0592578CCAM01521P1 | Daggett | 2 | 1521 | -4337.92 |
| CL9_532517542PSV_F0592578CCAM01521P1 | Daggett | 3 | 1521 | -4337.92 |
| CL9_532517608PSV_F0592578CCAM01521P1 | Daggett | 4 | 1521 | -4337.92 |
| CL9_532517804PSV_F0592578CCAM01521P1 | Daggett | 5 | 1521 | -4337.92 |
| CL9_532518578PSV_F0592578CCAM02521P1 | Madrid | 4 | 1521 | -4337.92 |
| CL9_532518654PSV_F0592578CCAM02521P1 | Madrid | 5 | 1521 | -4337.92 |
| CL9_532518720PSV_F0592578CCAM02521P1 | Madrid | 6 | 1521 | -4337.92 |
| CL9_532698242PSV_F0592668CCAM01523P1 | Islesford | 1 | 1523 | -4336.36 |

| | | | | |
|---|---|---|---|---|
| CL9_532698310PSV_F0592668CCAM01523P1 | Islesford | 2 | 1523 | -4336.36 |
| CL9_532698374PSV_F0592668CCAM01523P1 | Islesford | 3 | 1523 | -4336.36 |
| CL9_532698438PSV_F0592668CCAM01523P1 | Islesford | 4 | 1523 | -4336.36 |
| CL9_532698632PSV_F0592668CCAM01523P1 | Islesford | 5 | 1523 | -4336.36 |
| CL9_532698941PSV_F0592668CCAM02523P1 | Sutton_Island_ccam | 1 | 1523 | -4336.36 |
| CL9_532699009PSV_F0592668CCAM02523P1 | Sutton_Island_ccam | 2 | 1523 | -4336.36 |
| CL9_532699307PSV_F0592668CCAM02523P1 | Sutton_Island_ccam | 4 | 1523 | -4336.36 |
| CL9_532699375PSV_F0592668CCAM02523P1 | Sutton_Island_ccam | 5 | 1523 | -4336.36 |
| CL9_532699439PSV_F0592668CCAM02523P1 | Sutton_Island_ccam | 6 | 1523 | -4336.36 |
| CL9_532699674PSV_F0592668CCAM02523P1 | Sutton_Island_ccam | 7 | 1523 | -4336.36 |
| CL9_532699742PSV_F0592668CCAM02523P1 | Sutton_Island_ccam | 8 | 1523 | -4336.36 |
| CL9_532699806PSV_F0592668CCAM02523P1 | Sutton_Island_ccam | 9 | 1523 | -4336.36 |
| CL9_532699988PSV_F0592668CCAM02523P1 | Sutton_Island_ccam | 10 | 1523 | -4336.36 |
| CL9_532700303PSV_F0592668CCAM03523P1 | Duck_Harbor | 1 | 1523 | -4336.36 |
| CL9_532700378PSV_F0592668CCAM03523P1 | Duck_Harbor | 2 | 1523 | -4336.36 |
| CL9_532700444PSV_F0592668CCAM03523P1 | Duck_Harbor | 3 | 1523 | -4336.36 |
| CL9_532700821PSV_F0592668CCAM03523P1 | Duck_Harbor | 6 | 1523 | -4336.36 |
| CL9_532701057PSV_F0592668CCAM03523P1 | Duck_Harbor | 7 | 1523 | -4336.36 |
| CL9_532701132PSV_F0592668CCAM03523P1 | Duck_Harbor | 8 | 1523 | -4336.36 |
| CL9_532701198PSV_F0592668CCAM03523P1 | Duck_Harbor | 9 | 1523 | -4336.36 |
| CL9_532701372PSV_F0592668CCAM03523P1 | Duck_Harbor | 10 | 1523 | -4336.36 |
| CL9_532873445PSV_F0592668CCAM01525P1 | Ironbound_Island | 1 | 1525 | -4336.36 |
| CL9_532873520PSV_F0592668CCAM01525P1 | Ironbound_Island | 2 | 1525 | -4336.36 |
| CL9_532873590PSV_F0592668CCAM01525P1 | Ironbound_Island | 3 | 1525 | -4336.36 |
| CL9_532873819PSV_F0592668CCAM01525P1 | Ironbound_Island | 4 | 1525 | -4336.36 |
| CL9_532873887PSV_F0592668CCAM01525P1 | Ironbound_Island | 5 | 1525 | -4336.36 |
| CL9_532873953PSV_F0592668CCAM01525P1 | Ironbound_Island | 6 | 1525 | -4336.36 |
| CL9_532874149PSV_F0592668CCAM01525P1 | Ironbound_Island | 7 | 1525 | -4336.36 |
| CL9_532874215PSV_F0592668CCAM01525P1 | Ironbound_Island | 8 | 1525 | -4336.36 |
| CL9_532874378PSV_F0592668CCAM01525P1 | Ironbound_Island | 9 | 1525 | -4336.36 |
| CL9_533228035PSV_F0592830CCAM02528P1 | Thomas_Bay | 6 | 1529 | -4335.8 |
| CL9_533228300PSV_F0592830CCAM02528P1 | Thomas_Bay | 7 | 1529 | -4335.8 |
| CL9_533228377PSV_F0592830CCAM02528P1 | Thomas_Bay | 8 | 1529 | -4335.8 |
| CL9_533228443PSV_F0592830CCAM02528P1 | Thomas_Bay | 9 | 1529 | -4335.8 |
| CL9_533228662PSV_F0592830CCAM02528P1 | Thomas_Bay | 10 | 1529 | -4335.8 |
| CL9_533228992PSV_F0592830CCAM03528P1 | The_Anvil | 1 | 1529 | -4335.8 |
| CL9_533229305PSV_F0592830CCAM03528P1 | The_Anvil | 3 | 1529 | -4335.8 |
| CL9_533229447PSV_F0592830CCAM03528P1 | The_Anvil | 5 | 1529 | -4335.8 |
| CL9_533229693PSV_F0592830CCAM03528P1 | The_Anvil | 6 | 1529 | -4335.8 |
| CL9_533229769PSV_F0592830CCAM03528P1 | The_Anvil | 7 | 1529 | -4335.8 |
| CL9_533229943PSV_F0592830CCAM03528P1 | The_Anvil | 8 | 1529 | -4335.8 |
| CL9_533231120PSV_F0592830CCAM04528P1 | The_Ovens | 5 | 1529 | -4335.8 |
| CL9_533231186PSV_F0592830CCAM04528P1 | The_Ovens | 6 | 1529 | -4335.8 |
| CL9_533231422PSV_F0592830CCAM04528P1 | The_Ovens | 7 | 1529 | -4335.8 |
| CL9_533231498PSV_F0592830CCAM04528P1 | The_Ovens | 8 | 1529 | -4335.8 |
| CL9_533231564PSV_F0592830CCAM04528P1 | The_Ovens | 9 | 1529 | -4335.8 |
| CL9_533231747PSV_F0592830CCAM04528P1 | The_Ovens | 10 | 1529 | -4335.8 |
| CL9_533402940PSV_F0592830CCAM01531P1 | Precipice_DRT_ccam | 1 | 1531 | -4335.8 |
| CL9_533403015PSV_F0592830CCAM01531P1 | Precipice_DRT_ccam | 2 | 1531 | -4335.8 |
| CL9_533403081PSV_F0592830CCAM01531P1 | Precipice_DRT_ccam | 3 | 1531 | -4335.8 |
| CL9_533403323PSV_F0592830CCAM01531P1 | Precipice_DRT_ccam | 4 | 1531 | -4335.8 |
| CL9_533403395PSV_F0592830CCAM01531P1 | Precipice_DRT_ccam | 5 | 1531 | -4335.8 |
| CL9_533403461PSV_F0592830CCAM01531P1 | Precipice_DRT_ccam | 6 | 1531 | -4335.8 |
| CL9_533403703PSV_F0592830CCAM01531P1 | Precipice_DRT_ccam | 7 | 1531 | -4335.8 |
| CL9_533403775PSV_F0592830CCAM01531P1 | Precipice_DRT_ccam | 8 | 1531 | -4335.8 |
| CL9_533403979PSV_F0592830CCAM01531P1 | Precipice_DRT_ccam | 9 | 1531 | -4335.8 |
| CL9_533404297PSV_F0592830CCAM02531P1 | Frenchman_Bay | 1 | 1531 | -4335.8 |
| CL9_533404373PSV_F0592830CCAM02531P1 | Frenchman_Bay | 2 | 1531 | -4335.8 |
| CL9_533404439PSV_F0592830CCAM02531P1 | Frenchman_Bay | 3 | 1531 | -4335.8 |

| | | | | |
|---|---|---|---|---|
| CL9_533404696PSV_F0592830CCAM02531P1 | Frenchman_Bay | 4 | 1531 | -4335.8 |
| CL9_533404772PSV_F0592830CCAM02531P1 | Frenchman_Bay | 5 | 1531 | -4335.8 |
| CL9_533405091PSV_F0592830CCAM02531P1 | Frenchman_Bay | 7 | 1531 | -4335.8 |
| CL9_533405167PSV_F0592830CCAM02531P1 | Frenchman_Bay | 8 | 1531 | -4335.8 |
| CL9_533405233PSV_F0592830CCAM02531P1 | Frenchman_Bay | 9 | 1531 | -4335.8 |
| CL9_533405430PSV_F0592830CCAM02531P1 | Frenchman_Bay | 10 | 1531 | -4335.8 |
| CL9_533405891PSV_F0592830CCAM03531P1 | Hunters_Beach | 1 | 1531 | -4335.8 |
| CL9_533405959PSV_F0592830CCAM03531P1 | Hunters_Beach | 2 | 1531 | -4335.8 |
| CL9_533406023PSV_F0592830CCAM03531P1 | Hunters_Beach | 3 | 1531 | -4335.8 |
| CL9_533406284PSV_F0592830CCAM03531P1 | Hunters_Beach | 4 | 1531 | -4335.8 |
| CL9_533406352PSV_F0592830CCAM03531P1 | Hunters_Beach | 5 | 1531 | -4335.8 |
| CL9_533406425PSV_F0592830CCAM03531P1 | Hunters_Beach | 6 | 1531 | -4335.8 |
| CL9_533406682PSV_F0592830CCAM03531P1 | Hunters_Beach | 7 | 1531 | -4335.8 |
| CL9_533406814PSV_F0592830CCAM03531P1 | Hunters_Beach | 9 | 1531 | -4335.8 |
| CL9_533407022PSV_F0592830CCAM03531P1 | Hunters_Beach | 10 | 1531 | -4335.8 |
| CL9_533756206PSV_F0592830CCAM01535P1 | West_Tremont | 1 | 1535 | -4335.8 |
| CL9_533756274PSV_F0592830CCAM01535P1 | West_Tremont | 2 | 1535 | -4335.8 |
| CL9_533756338PSV_F0592830CCAM01535P1 | West_Tremont | 3 | 1535 | -4335.8 |
| CL9_533756584PSV_F0592830CCAM01535P1 | West_Tremont | 4 | 1535 | -4335.8 |
| CL9_533756652PSV_F0592830CCAM01535P1 | West_Tremont | 5 | 1535 | -4335.8 |
| CL9_533756716PSV_F0592830CCAM01535P1 | West_Tremont | 6 | 1535 | -4335.8 |
| CL9_533756962PSV_F0592830CCAM01535P1 | West_Tremont | 7 | 1535 | -4335.8 |
| CL9_533757031PSV_F0592830CCAM01535P1 | West_Tremont | 8 | 1535 | -4335.8 |
| CL9_533757094PSV_F0592830CCAM01535P1 | West_Tremont | 9 | 1535 | -4335.8 |
| CL9_533757296PSV_F0592830CCAM01535P1 | West_Tremont | 10 | 1535 | -4335.8 |
| CL9_533757634PSV_F0592830CCAM02535P1 | Eastern_Head | 1 | 1535 | -4335.8 |
| CL9_533757702PSV_F0592830CCAM02535P1 | Eastern_Head | 2 | 1535 | -4335.8 |
| CL9_533757766PSV_F0592830CCAM02535P1 | Eastern_Head | 3 | 1535 | -4335.8 |
| CL9_533757830PSV_F0592830CCAM02535P1 | Eastern_Head | 4 | 1535 | -4335.8 |
| CL9_533758032PSV_F0592830CCAM02535P1 | Eastern_Head | 5 | 1535 | -4335.8 |
| CL9_533931017PSV_F0592830CCAM01537P1 | Birch_Point | 1 | 1537 | -4335.8 |
| CL9_533931160PSV_F0592830CCAM01537P1 | Birch_Point | 3 | 1537 | -4335.8 |
| CL9_533931407PSV_F0592830CCAM01537P1 | Birch_Point | 4 | 1537 | -4335.8 |
| CL9_533931484PSV_F0592830CCAM01537P1 | Birch_Point | 5 | 1537 | -4335.8 |
| CL9_533931549PSV_F0592830CCAM01537P1 | Birch_Point | 6 | 1537 | -4335.8 |
| CL9_533931875PSV_F0592830CCAM01537P1 | Birch_Point | 8 | 1537 | -4335.8 |
| CL9_533934300PSV_F0592830CCAM03537P1 | Compass_Harbor | 4 | 1537 | -4335.8 |
| CL9_533934368PSV_F0592830CCAM03537P1 | Compass_Harbor | 5 | 1537 | -4335.8 |
| CL9_533934432PSV_F0592830CCAM03537P1 | Compass_Harbor | 6 | 1537 | -4335.8 |
| CL9_533934688PSV_F0592830CCAM03537P1 | Compass_Harbor | 7 | 1537 | -4335.8 |
| CL9_533934820PSV_F0592830CCAM03537P1 | Compass_Harbor | 9 | 1537 | -4335.8 |
| CL9_534289198PSV_F0592830CCAM01541P1 | Hunters_Beach_2 | 3 | 1541 | -4335.8 |
| CL9_534289388PSV_F0592830CCAM01541P1 | Hunters_Beach_2 | 4 | 1541 | -4335.8 |
| CL9_534291720PSV_F0592830CCAM03541P1 | Gorham_Mountain_Top | 1 | 1541 | -4335.8 |
| CL9_534291788PSV_F0592830CCAM03541P1 | Gorham_Mountain_Top | 2 | 1541 | -4335.8 |
| CL9_534291852PSV_F0592830CCAM03541P1 | Gorham_Mountain_Top | 3 | 1541 | -4335.8 |
| CL9_534291916PSV_F0592830CCAM03541P1 | Gorham_Mountain_Top | 4 | 1541 | -4335.8 |
| CL9_534292122PSV_F0592830CCAM03541P1 | Gorham_Mountain_Top | 5 | 1541 | -4335.8 |
| CL9_534380831PSV_F0592830CCAM01542P1 | Hunters_Beach_3 | 7 | 1542 | -4335.8 |
| CL9_534380897PSV_F0592830CCAM01542P1 | Hunters_Beach_3 | 8 | 1542 | -4335.8 |
| CL9_534381075PSV_F0592830CCAM01542P1 | Hunters_Beach_3 | 9 | 1542 | -4335.8 |
| CL9_534381542PSV_F0592830CCAM02542P1 | Hunters_Beach_3 | 10 | 1542 | -4335.8 |
| CL9_534382454PSV_F0592830CCAM03542P1 | Youngs_Mountain | 3 | 1542 | -4335.8 |
| CL9_534382520PSV_F0592830CCAM03542P1 | Youngs_Mountain | 4 | 1542 | -4335.8 |
| CL9_534382683PSV_F0592830CCAM03542P1 | Youngs_Mountain | 5 | 1542 | -4335.8 |
| CL9_534465929PSV_F0592830CCAM01543P1 | Bracy_Cove | 2 | 1543 | -4335.8 |
| CL9_534466230PSV_F0592830CCAM01543P1 | Bracy_Cove | 4 | 1543 | -4335.8 |
| CL9_534466701PSV_F0592830CCAM01543P1 | Bracy_Cove | 8 | 1543 | -4335.8 |
| CL9_534466766PSV_F0592830CCAM01543P1 | Bracy_Cove | 9 | 1543 | -4335.8 |

| | | | | |
|---|---|---|---|---|
| CL9_534467407PSV_F0592830CCAM02543P1 | The_Anvil_2 | 1 | 1543 | -4335.8 |
| CL9_534467475PSV_F0592830CCAM02543P1 | The_Anvil_2 | 2 | 1543 | -4335.8 |
| CL9_534467636PSV_F0592830CCAM02543P1 | The_Anvil_2 | 3 | 1543 | -4335.8 |
| CL9_534467699PSV_F0592830CCAM02543P1 | The_Anvil_2 | 4 | 1543 | -4335.8 |
| CL9_534467762PSV_F0592830CCAM02543P1 | The_Anvil_2 | 5 | 1543 | -4335.8 |
| CL9_534467964PSV_F0592830CCAM02543P1 | The_Anvil_2 | 6 | 1543 | -4335.8 |
| CL9_534468027PSV_F0592830CCAM02543P1 | The_Anvil_2 | 7 | 1543 | -4335.8 |
| CL9_534468090PSV_F0592830CCAM02543P1 | The_Anvil_2 | 8 | 1543 | -4335.8 |
| CL9_534468153PSV_F0592830CCAM02543P1 | The_Anvil_2 | 9 | 1543 | -4335.8 |
| CL9_534468314PSV_F0592830CCAM02543P1 | The_Anvil_2 | 10 | 1543 | -4335.8 |
| CL9_534559524PSV_F0592830CCAM01544P1 | Aunt_Betty_Pond | 1 | 1544 | -4335.8 |
| CL9_534559600PSV_F0592830CCAM01544P1 | Aunt_Betty_Pond | 2 | 1544 | -4335.8 |
| CL9_534559667PSV_F0592830CCAM01544P1 | Aunt_Betty_Pond | 3 | 1544 | -4335.8 |
| CL9_534559904PSV_F0592830CCAM01544P1 | Aunt_Betty_Pond | 4 | 1544 | -4335.8 |
| CL9_534559980PSV_F0592830CCAM01544P1 | Aunt_Betty_Pond | 5 | 1544 | -4335.8 |
| CL9_534560046PSV_F0592830CCAM01544P1 | Aunt_Betty_Pond | 6 | 1544 | -4335.8 |
| CL9_534560284PSV_F0592830CCAM01544P1 | Aunt_Betty_Pond | 7 | 1544 | -4335.8 |
| CL9_534560360PSV_F0592830CCAM01544P1 | Aunt_Betty_Pond | 8 | 1544 | -4335.8 |
| CL9_534560533PSV_F0592830CCAM01544P1 | Aunt_Betty_Pond | 9 | 1544 | -4335.8 |
| CL9_534561010PSV_F0592830CCAM02544P1 | Kebo_Mountain | 1 | 1544 | -4335.8 |
| CL9_534561085PSV_F0592830CCAM02544P1 | Kebo_Mountain | 2 | 1544 | -4335.8 |
| CL9_534561150PSV_F0592830CCAM02544P1 | Kebo_Mountain | 3 | 1544 | -4335.8 |
| CL9_534561407PSV_F0592830CCAM02544P1 | Kebo_Mountain | 4 | 1544 | -4335.8 |
| CL9_534561772PSV_F0592830CCAM02544P1 | Kebo_Mountain | 7 | 1544 | -4335.8 |
| CL9_534561847PSV_F0592830CCAM02544P1 | Kebo_Mountain | 8 | 1544 | -4335.8 |
| CL9_534652662PSV_F0592830CCAM01545P1 | The_Anvil_3 | 1 | 1545 | -4335.8 |
| CL9_534652725PSV_F0592830CCAM01545P1 | The_Anvil_3 | 2 | 1545 | -4335.8 |
| CL9_534652788PSV_F0592830CCAM01545P1 | The_Anvil_3 | 3 | 1545 | -4335.8 |
| CL9_534652851PSV_F0592830CCAM01545P1 | The_Anvil_3 | 4 | 1545 | -4335.8 |
| CL9_534653098PSV_F0592830CCAM01545P1 | The_Anvil_3 | 5 | 1545 | -4335.8 |
| CL9_534653167PSV_F0592830CCAM01545P1 | The_Anvil_3 | 6 | 1545 | -4335.8 |
| CL9_534653230PSV_F0592830CCAM01545P1 | The_Anvil_3 | 7 | 1545 | -4335.8 |
| CL9_534653436PSV_F0592830CCAM01545P1 | The_Anvil_3 | 8 | 1545 | -4335.8 |
| CL9_534654659PSV_F0592830CCAM02545P1 | Blue_Hill | 10 | 1545 | -4335.8 |
| CL9_534917011PSV_F0592830CCAM02548P1 | Blue_Hill_2 | 5 | 1548 | -4335.8 |
| CL9_534917077PSV_F0592830CCAM02548P1 | Blue_Hill_2 | 6 | 1548 | -4335.8 |
| CL9_534917335PSV_F0592830CCAM02548P1 | Blue_Hill_2 | 7 | 1548 | -4335.8 |
| CL9_534917410PSV_F0592830CCAM02548P1 | Blue_Hill_2 | 8 | 1548 | -4335.8 |
| CL9_534917684PSV_F0592830CCAM02548P1 | Blue_Hill_2 | 10 | 1548 | -4335.8 |
| CL9_535089603PSV_F0592830CCAM02550P1 | Canon_Brook | 5 | 1550 | -4335.8 |
| CL9_535089668PSV_F0592830CCAM02550P1 | Canon_Brook | 6 | 1550 | -4335.8 |
| CL9_535089731PSV_F0592830CCAM02550P1 | Canon_Brook | 7 | 1550 | -4335.8 |
| CL9_535090051PSV_F0592830CCAM02550P1 | Canon_Brook | 10 | 1550 | -4335.8 |
| CL9_535265853PSV_F0592830CCAM01552P1 | Hall_Quarry | 1 | 1552 | -4335.8 |
| CL9_535265928PSV_F0592830CCAM01552P1 | Hall_Quarry | 2 | 1552 | -4335.8 |
| CL9_535265993PSV_F0592830CCAM01552P1 | Hall_Quarry | 3 | 1552 | -4335.8 |
| CL9_535266223PSV_F0592830CCAM01552P1 | Hall_Quarry | 4 | 1552 | -4335.8 |
| CL9_535266291PSV_F0592830CCAM01552P1 | Hall_Quarry | 5 | 1552 | -4335.8 |
| CL9_535266357PSV_F0592830CCAM01552P1 | Hall_Quarry | 6 | 1552 | -4335.8 |
| CL9_535266587PSV_F0592830CCAM01552P1 | Hall_Quarry | 7 | 1552 | -4335.8 |
| CL9_535266654PSV_F0592830CCAM01552P1 | Hall_Quarry | 8 | 1552 | -4335.8 |
| CL9_535266850PSV_F0592830CCAM01552P1 | Hall_Quarry | 9 | 1552 | -4335.8 |
| CL9_535267200PSV_F0592830CCAM02552P1 | Long_Porcupine | 1 | 1552 | -4335.8 |
| CL9_535267275PSV_F0592830CCAM02552P1 | Long_Porcupine | 2 | 1552 | -4335.8 |
| CL9_535267340PSV_F0592830CCAM02552P1 | Long_Porcupine | 3 | 1552 | -4335.8 |
| CL9_535267995PSV_F0592830CCAM02552P1 | Long_Porcupine | 10 | 1552 | -4335.8 |
| CL9_535268060PSV_F0592830CCAM02552P1 | Long_Porcupine | 11 | 1552 | -4335.8 |
| CL9_535268125PSV_F0592830CCAM02552P1 | Long_Porcupine | 12 | 1552 | -4335.8 |
| CL9_535268374PSV_F0592830CCAM02552P1 | Long_Porcupine | 13 | 1552 | -4335.8 |

| | | | | |
|---|---|---|---|---|
| CL9_535268449PSV_F0592830CCAM02552P1 | Long_Porcupine | 14 | 1552 | -4335.8 |
| CL9_535268514PSV_F0592830CCAM02552P1 | Long_Porcupine | 15 | 1552 | -4335.8 |
| CL9_535268644PSV_F0592830CCAM02552P1 | Long_Porcupine | 17 | 1552 | -4335.8 |
| CL9_535268775PSV_F0592830CCAM02552P1 | Long_Porcupine | 19 | 1552 | -4335.8 |
| CL9_535536482PSV_F0593004CCAM01555P1 | Somes_Sound | 1 | 1555 | -4335.75 |
| CL9_535536557PSV_F0593004CCAM01555P1 | Somes_Sound | 2 | 1555 | -4335.75 |
| CL9_535536622PSV_F0593004CCAM01555P1 | Somes_Sound | 3 | 1555 | -4335.75 |
| CL9_535536817PSV_F0593004CCAM01555P1 | Somes_Sound | 4 | 1555 | -4335.75 |
| CL9_535537210PSV_F0593004CCAM01555P1 | Somes_Sound | 8 | 1555 | -4335.75 |
| CL9_535537278PSV_F0593004CCAM01555P1 | Somes_Sound | 9 | 1555 | -4335.75 |
| CL9_535537473PSV_F0593004CCAM01555P1 | Somes_Sound | 10 | 1555 | -4335.75 |
| CL9_535537855PSV_F0593004CCAM02555P1 | Schoodic_Peninsula | 2 | 1555 | -4335.75 |
| CL9_535538112PSV_F0593004CCAM02555P1 | Schoodic_Peninsula | 4 | 1555 | -4335.75 |
| CL9_535538177PSV_F0593004CCAM02555P1 | Schoodic_Peninsula | 5 | 1555 | -4335.75 |
| CL9_535538241PSV_F0593004CCAM02555P1 | Schoodic_Peninsula | 6 | 1555 | -4335.75 |
| CL9_535538435PSV_F0593004CCAM02555P1 | Schoodic_Peninsula | 7 | 1555 | -4335.75 |
| CL9_535538500PSV_F0593004CCAM02555P1 | Schoodic_Peninsula | 8 | 1555 | -4335.75 |
| CL9_535538563PSV_F0593004CCAM02555P1 | Schoodic_Peninsula | 9 | 1555 | -4335.75 |
| CL9_535538757PSV_F0593004CCAM02555P1 | Schoodic_Peninsula | 10 | 1555 | -4335.75 |
| CL9_535539849PSV_F0593004CCAM03555P1 | South_Bubble | 5 | 1555 | -4335.75 |
| CL9_535539917PSV_F0593004CCAM03555P1 | South_Bubble | 6 | 1555 | -4335.75 |
| CL9_535539981PSV_F0593004CCAM03555P1 | South_Bubble | 7 | 1555 | -4335.75 |
| CL9_535540045PSV_F0593004CCAM03555P1 | South_Bubble | 8 | 1555 | -4335.75 |
| CL9_535540293PSV_F0593004CCAM03555P1 | South_Bubble | 10 | 1555 | -4335.75 |
| CL9_535540608PSV_F0593004CCAM04555P1 | Schooner_Head | 1 | 1555 | -4335.75 |
| CL9_535540673PSV_F0593004CCAM04555P1 | Schooner_Head | 2 | 1555 | -4335.75 |
| CL9_535540737PSV_F0593004CCAM04555P1 | Schooner_Head | 3 | 1555 | -4335.75 |
| CL9_535540950PSV_F0593004CCAM04555P1 | Schooner_Head | 4 | 1555 | -4335.75 |
| CL9_535541018PSV_F0593004CCAM04555P1 | Schooner_Head | 5 | 1555 | -4335.75 |
| CL9_535541082PSV_F0593004CCAM04555P1 | Schooner_Head | 6 | 1555 | -4335.75 |
| CL9_535541295PSV_F0593004CCAM04555P1 | Schooner_Head | 7 | 1555 | -4335.75 |
| CL9_535541363PSV_F0593004CCAM04555P1 | Schooner_Head | 8 | 1555 | -4335.75 |
| CL9_536517354PSV_F0593016CCAM01566P1 | Moore_Harbor | 1 | 1566 | -4335.75 |
| CL9_536518127PSV_F0593016CCAM01566P1 | Moore_Harbor | 7 | 1566 | -4335.75 |
| CL9_536518195PSV_F0593016CCAM01566P1 | Moore_Harbor | 8 | 1566 | -4335.75 |
| CL9_536518259PSV_F0593016CCAM01566P1 | Moore_Harbor | 9 | 1566 | -4335.75 |
| CL9_536518467PSV_F0593016CCAM01566P1 | Moore_Harbor | 10 | 1566 | -4335.75 |
| CL9_536519301PSV_F0593016CCAM02566P1 | Northeast_Harbor | 5 | 1566 | -4335.75 |
| CL9_536519377PSV_F0593016CCAM02566P1 | Northeast_Harbor | 6 | 1566 | -4335.75 |
| CL9_536519443PSV_F0593016CCAM02566P1 | Northeast_Harbor | 7 | 1566 | -4335.75 |
| CL9_536519630PSV_F0593016CCAM02566P1 | Northeast_Harbor | 8 | 1566 | -4335.75 |
| CL9_536519696PSV_F0593016CCAM02566P1 | Northeast_Harbor | 9 | 1566 | -4335.75 |
| CL9_536519883PSV_F0593016CCAM02566P1 | Northeast_Harbor | 10 | 1566 | -4335.75 |
| CL9_536690255PSV_F0593016CCAM04566P1 | Deep_Cove | 3 | 1568 | -4335.75 |
| CL9_536690864PSV_F0593016CCAM04566P1 | Deep_Cove | 7 | 1568 | -4335.75 |
| CL9_536691190PSV_F0593016CCAM04566P1 | Deep_Cove | 10 | 1568 | -4335.75 |
| CL9_536691674PSV_F0593016CCAM05566P1 | Dix_Point | 2 | 1568 | -4335.75 |
| CL9_536691738PSV_F0593016CCAM05566P1 | Dix_Point | 3 | 1568 | -4335.75 |
| CL9_536692015PSV_F0593016CCAM05566P1 | Dix_Point | 4 | 1568 | -4335.75 |
| CL9_536692083PSV_F0593016CCAM05566P1 | Dix_Point | 5 | 1568 | -4335.75 |
| CL9_536692147PSV_F0593016CCAM05566P1 | Dix_Point | 6 | 1568 | -4335.75 |
| CL9_536692211PSV_F0593016CCAM05566P1 | Dix_Point | 7 | 1568 | -4335.75 |
| CL9_536692474PSV_F0593016CCAM05566P1 | Dix_Point | 8 | 1568 | -4335.75 |
| CL9_536692543PSV_F0593016CCAM05566P1 | Dix_Point | 9 | 1568 | -4335.75 |
| CL9_536692765PSV_F0593016CCAM05566P1 | Dix_Point | 10 | 1568 | -4335.75 |
| CL9_536780277PSV_F0593016CCAM01569P1 | Beech_Mountain_ccam | 1 | 1569 | -4335.75 |
| CL9_536780345PSV_F0593016CCAM01569P1 | Beech_Mountain_ccam | 2 | 1569 | -4335.75 |
| CL9_536780409PSV_F0593016CCAM01569P1 | Beech_Mountain_ccam | 3 | 1569 | -4335.75 |
| CL9_536780473PSV_F0593016CCAM01569P1 | Beech_Mountain_ccam | 4 | 1569 | -4335.75 |

| | | | | |
|---|---|---|---|---|
| CL9_536780537PSV_F0593016CCAM01569P1 | Beech_Mountain_ccam | 5 | 1569 | -4335.75 |
| CL9_536780601PSV_F0593016CCAM01569P1 | Beech_Mountain_ccam | 6 | 1569 | -4335.75 |
| CL9_536780665PSV_F0593016CCAM01569P1 | Beech_Mountain_ccam | 7 | 1569 | -4335.75 |
| CL9_536780729PSV_F0593016CCAM01569P1 | Beech_Mountain_ccam | 8 | 1569 | -4335.75 |
| CL9_536780793PSV_F0593016CCAM01569P1 | Beech_Mountain_ccam | 9 | 1569 | -4335.75 |
| CL9_536780987PSV_F0593016CCAM01569P1 | Beech_Mountain_ccam | 10 | 1569 | -4335.75 |
| CL9_536871111PSV_F0593016CCAM03570P1 | Deep_Cove_2 | 3 | 1570 | -4335.75 |
| CL9_536871174PSV_F0593016CCAM03570P1 | Deep_Cove_2 | 4 | 1570 | -4335.75 |
| CL9_536871439PSV_F0593016CCAM03570P1 | Deep_Cove_2 | 8 | 1570 | -4335.75 |
| CL9_536871697PSV_F0593016CCAM03570P1 | Deep_Cove_2 | 10 | 1570 | -4335.75 |
| CL9_536953152PSV_F0593016CCAM01571P1 | Gilley_Field_ccam | 3 | 1571 | -4335.75 |
| CL9_536953341PSV_F0593016CCAM01571P1 | Gilley_Field_ccam | 6 | 1571 | -4335.75 |
| CL9_536953404PSV_F0593016CCAM01571P1 | Gilley_Field_ccam | 7 | 1571 | -4335.75 |
| CL9_536953724PSV_F0593016CCAM01571P1 | Gilley_Field_ccam | 10 | 1571 | -4335.75 |
| CL9_537145479PSV_F0600000CCAM02572P1 | Birch_Harbor_Mountain | 1 | 1573 | -4335.04 |
| CL9_537145554PSV_F0600000CCAM02572P1 | Birch_Harbor_Mountain | 2 | 1573 | -4335.04 |
| CL9_537146000PSV_F0600000CCAM02572P1 | Birch_Harbor_Mountain | 7 | 1573 | -4335.04 |
| CL9_537146065PSV_F0600000CCAM02572P1 | Birch_Harbor_Mountain | 8 | 1573 | -4335.04 |
| CL9_537146130PSV_F0600000CCAM02572P1 | Birch_Harbor_Mountain | 9 | 1573 | -4335.04 |
| CL9_537217525PSV_F0600000CCAM03572P1 | Isle_Au_Haut_ccam | 1 | 1574 | -4335.04 |
| CL9_537217601PSV_F0600000CCAM03572P1 | Isle_Au_Haut_ccam | 2 | 1574 | -4335.04 |
| CL9_537217667PSV_F0600000CCAM03572P1 | Isle_Au_Haut_ccam | 3 | 1574 | -4335.04 |
| CL9_537217767PSV_F0600000CCAM03572P1 | Isle_Au_Haut_ccam | 4 | 1574 | -4335.04 |
| CL9_537217833PSV_F0600000CCAM03572P1 | Isle_Au_Haut_ccam | 5 | 1574 | -4335.04 |
| CL9_537217899PSV_F0600000CCAM03572P1 | Isle_Au_Haut_ccam | 6 | 1574 | -4335.04 |
| CL9_537217999PSV_F0600000CCAM03572P1 | Isle_Au_Haut_ccam | 7 | 1574 | -4335.04 |
| CL9_537218065PSV_F0600000CCAM03572P1 | Isle_Au_Haut_ccam | 8 | 1574 | -4335.04 |
| CL9_537218228PSV_F0600000CCAM03572P1 | Isle_Au_Haut_ccam | 9 | 1574 | -4335.04 |
| CL9_537400057PSV_F0600180CCAM01576P1 | Dorr_Mountain_CCAM | 1 | 1576 | -4333.45 |
| CL9_537400132PSV_F0600180CCAM01576P1 | Dorr_Mountain_CCAM | 2 | 1576 | -4333.45 |
| CL9_537400753PSV_F0600180CCAM02576P1 | Parkman_Mountain | 1 | 1576 | -4333.45 |
| CL9_537400822PSV_F0600180CCAM02576P1 | Parkman_Mountain | 2 | 1576 | -4333.45 |
| CL9_537400886PSV_F0600180CCAM02576P1 | Parkman_Mountain | 3 | 1576 | -4333.45 |
| CL9_537400950PSV_F0600180CCAM02576P1 | Parkman_Mountain | 4 | 1576 | -4333.45 |
| CL9_537489608PSV_F0600396CCAM02577P1 | Day_Mountain | 1 | 1577 | -4331.33 |
| CL9_537489683PSV_F0600396CCAM02577P1 | Day_Mountain | 2 | 1577 | -4331.33 |
| CL9_537489749PSV_F0600396CCAM02577P1 | Day_Mountain | 3 | 1577 | -4331.33 |
| CL9_537489815PSV_F0600396CCAM02577P1 | Day_Mountain | 4 | 1577 | -4331.33 |
| CL9_537490018PSV_F0600396CCAM02577P1 | Day_Mountain | 5 | 1577 | -4331.33 |
| CL9_537576027PSV_F0600684CCAM01578P1 | Megunticook | 2 | 1578 | -4327.39 |
| CL9_537576292PSV_F0600684CCAM01578P1 | Megunticook | 4 | 1578 | -4327.39 |
| CL9_537753650PSV_F0600888CCAM03579P1 | Oak Bay | 1 | 1580 | -4325.12 |
| CL9_537753725PSV_F0600888CCAM03579P1 | Oak Bay | 2 | 1580 | -4325.12 |
| CL9_537753791PSV_F0600888CCAM03579P1 | Oak Bay | 3 | 1580 | -4325.12 |
| CL9_537753857PSV_F0600888CCAM03579P1 | Oak Bay | 4 | 1580 | -4325.12 |
| CL9_537754065PSV_F0600888CCAM03579P1 | Oak Bay | 5 | 1580 | -4325.12 |
| CL9_537768000PSV_F0600888CCAM07579P1 | Traveler | 1 | 1580 | -4325.12 |
| CL9_537768075PSV_F0600888CCAM07579P1 | Traveler | 2 | 1580 | -4325.12 |
| CL9_537768141PSV_F0600888CCAM07579P1 | Traveler | 3 | 1580 | -4325.12 |
| CL9_537768207PSV_F0600888CCAM07579P1 | Traveler | 4 | 1580 | -4325.12 |
| CL9_537768394PSV_F0600888CCAM07579P1 | Traveler | 5 | 1580 | -4325.12 |
| CL9_537769046PSV_F0600888CCAM08579P1 | Mars_Hill | 1 | 1580 | -4325.12 |
| CL9_537769121PSV_F0600888CCAM08579P1 | Mars_Hill | 2 | 1580 | -4325.12 |
| CL9_537769187PSV_F0600888CCAM08579P1 | Mars_Hill | 3 | 1580 | -4325.12 |
| CL9_537769404PSV_F0600888CCAM08579P1 | Mars_Hill | 4 | 1580 | -4325.12 |
| CL9_537769470PSV_F0600888CCAM08579P1 | Mars_Hill | 5 | 1580 | -4325.12 |
| CL9_537769536PSV_F0600888CCAM08579P1 | Mars_Hill | 6 | 1580 | -4325.12 |
| CL9_537769753PSV_F0600888CCAM08579P1 | Mars_Hill | 7 | 1580 | -4325.12 |
| CL9_537770002PSV_F0600888CCAM08579P1 | Mars_Hill | 9 | 1580 | -4325.12 |

| | | | | |
|---|---|---|---|---|
| CL9_538021031PSV_F0601266CCAM02583P1 | Benner_Hill_2 | 5 | 1583 | -4321.49 |
| CL9_538109786PSV_F0601422CCAM01584P1 | Frost_Pond | 1 | 1584 | -4321.16 |
| CL9_538109855PSV_F0601422CCAM01584P1 | Frost_Pond | 2 | 1584 | -4321.16 |
| CL9_538109919PSV_F0601422CCAM01584P1 | Frost_Pond | 3 | 1584 | -4321.16 |
| CL9_538109983PSV_F0601422CCAM01584P1 | Frost_Pond | 4 | 1584 | -4321.16 |
| CL9_538110185PSV_F0601422CCAM01584P1 | Frost_Pond | 5 | 1584 | -4321.16 |
| CL9_539969749PSV_F0610000CCAM01605P1 | Carys_Mills_ccam | 1 | 1605 | -4311.51 |
| CL9_539969824PSV_F0610000CCAM01605P1 | Carys_Mills_ccam | 2 | 1605 | -4311.51 |
| CL9_539969889PSV_F0610000CCAM01605P1 | Carys_Mills_ccam | 3 | 1605 | -4311.51 |
| CL9_539969954PSV_F0610000CCAM01605P1 | Carys_Mills_ccam | 4 | 1605 | -4311.51 |
| CL9_539970150PSV_F0610000CCAM01605P1 | Carys_Mills_ccam | 7 | 1605 | -4311.51 |
| CL9_539970215PSV_F0610000CCAM01605P1 | Carys_Mills_ccam | 8 | 1605 | -4311.51 |
| CL9_539970281PSV_F0610000CCAM01605P1 | Carys_Mills_ccam | 9 | 1605 | -4311.51 |
| CL9_539970476PSV_F0610000CCAM01605P1 | Carys_Mills_ccam | 10 | 1605 | -4311.51 |
| CL9_540067164PSV_F0610156CCAM02606P1 | Quoddy | 1 | 1606 | -4313.46 |
| CL9_540067232PSV_F0610156CCAM02606P1 | Quoddy | 2 | 1606 | -4313.46 |
| CL9_540067295PSV_F0610156CCAM02606P1 | Quoddy | 3 | 1606 | -4313.46 |
| CL9_540067358PSV_F0610156CCAM02606P1 | Quoddy | 4 | 1606 | -4313.46 |
| CL9_540067421PSV_F0610156CCAM02606P1 | Quoddy | 5 | 1606 | -4313.46 |
| CL9_540067484PSV_F0610156CCAM02606P1 | Quoddy | 6 | 1606 | -4313.46 |
| CL9_540067547PSV_F0610156CCAM02606P1 | Quoddy | 7 | 1606 | -4313.46 |
| CL9_540067610PSV_F0610156CCAM02606P1 | Quoddy | 8 | 1606 | -4313.46 |
| CL9_540067867PSV_F0610156CCAM02606P1 | Quoddy | 10 | 1606 | -4313.46 |
| CL9_540238402PSV_F0610156CCAM04606P1 | Cushing | 1 | 1609 | -4313.46 |
| CL9_540238470PSV_F0610156CCAM04606P1 | Cushing | 2 | 1609 | -4313.46 |
| CL9_540238534PSV_F0610156CCAM04606P1 | Cushing | 3 | 1609 | -4313.46 |
| CL9_540238769PSV_F0610156CCAM04606P1 | Cushing | 4 | 1609 | -4313.46 |
| CL9_540238837PSV_F0610156CCAM04606P1 | Cushing | 5 | 1609 | -4313.46 |
| CL9_540238901PSV_F0610156CCAM04606P1 | Cushing | 6 | 1609 | -4313.46 |
| CL9_540239136PSV_F0610156CCAM04606P1 | Cushing | 7 | 1609 | -4313.46 |
| CL9_540239204PSV_F0610156CCAM04606P1 | Cushing | 8 | 1609 | -4313.46 |
| CL9_540239268PSV_F0610156CCAM04606P1 | Cushing | 9 | 1609 | -4313.46 |
| CL9_540239462PSV_F0610156CCAM04606P1 | Cushing | 10 | 1609 | -4313.46 |
| CL9_540239775PSV_F0610156CCAM05606P1 | Bucksport | 1 | 1609 | -4313.46 |
| CL9_540239843PSV_F0610156CCAM05606P1 | Bucksport | 2 | 1609 | -4313.46 |
| CL9_540240141PSV_F0610156CCAM05606P1 | Bucksport | 4 | 1609 | -4313.46 |
| CL9_540240206PSV_F0610156CCAM05606P1 | Bucksport | 5 | 1609 | -4313.46 |
| CL9_540240270PSV_F0610156CCAM05606P1 | Bucksport | 6 | 1609 | -4313.46 |
| CL9_540240537PSV_F0610156CCAM05606P1 | Bucksport | 8 | 1609 | -4313.46 |
| CL9_540331308PSV_F0610252CCAM02609P1 | Spurwink_ccam | 1 | 1609 | -4313.44 |
| CL9_540331383PSV_F0610252CCAM02609P1 | Spurwink_ccam | 2 | 1609 | -4313.44 |
| CL9_540331449PSV_F0610252CCAM02609P1 | Spurwink_ccam | 3 | 1609 | -4313.44 |
| CL9_540331548PSV_F0610252CCAM02609P1 | Spurwink_ccam | 4 | 1609 | -4313.44 |
| CL9_540331614PSV_F0610252CCAM02609P1 | Spurwink_ccam | 5 | 1609 | -4313.44 |
| CL9_540331818PSV_F0610252CCAM02609P1 | Spurwink_ccam | 6 | 1609 | -4313.44 |
| CL9_540331924PSV_F0610252CCAM02609P1 | Spurwink_ccam | 7 | 1609 | -4313.44 |
| CL9_540331990PSV_F0610252CCAM02609P1 | Spurwink_ccam | 8 | 1609 | -4313.44 |
| CL9_540332194PSV_F0610252CCAM02609P1 | Spurwink_ccam | 9 | 1609 | -4313.44 |
| CL9_540416419PSV_F0610252CCAM01610P1 | Fogelin | 1 | 1610 | -4313.44 |
| CL9_540416494PSV_F0610252CCAM01610P1 | Fogelin | 2 | 1610 | -4313.44 |
| CL9_540416560PSV_F0610252CCAM01610P1 | Fogelin | 3 | 1610 | -4313.44 |
| CL9_540416626PSV_F0610252CCAM01610P1 | Fogelin | 4 | 1610 | -4313.44 |
| CL9_540416863PSV_F0610252CCAM01610P1 | Fogelin | 5 | 1610 | -4313.44 |
| CL9_540416938PSV_F0610252CCAM01610P1 | Fogelin | 6 | 1610 | -4313.44 |
| CL9_540417008PSV_F0610252CCAM01610P1 | Fogelin | 7 | 1610 | -4313.44 |
| CL9_540417074PSV_F0610252CCAM01610P1 | Fogelin | 8 | 1610 | -4313.44 |
| CL9_540502182PSV_F0610456CCAM01611P1 | Patch_Mountain | 1 | 1611 | -4312.11 |
| CL9_540502250PSV_F0610456CCAM01611P1 | Patch_Mountain | 2 | 1611 | -4312.11 |
| CL9_540502314PSV_F0610456CCAM01611P1 | Patch_Mountain | 3 | 1611 | -4312.11 |

| | | | | |
|---|---|---|---|---|
| CL9_540502378PSV_F0610456CCAM01611P1 | Patch_Mountain | 4 | 1611 | -4312.11 |
| CL9_540502441PSV_F0610456CCAM01611P1 | Patch_Mountain | 5 | 1611 | -4312.11 |
| CL9_540502504PSV_F0610456CCAM01611P1 | Patch_Mountain | 6 | 1611 | -4312.11 |
| CL9_540502665PSV_F0610456CCAM01611P1 | Patch_Mountain | 7 | 1611 | -4312.11 |
| CL9_540592151PSV_F0610648CCAM01612P1 | Frenchville | 1 | 1612 | -4309.91 |
| CL9_540592226PSV_F0610648CCAM01612P1 | Frenchville | 2 | 1612 | -4309.91 |
| CL9_540592292PSV_F0610648CCAM01612P1 | Frenchville | 3 | 1612 | -4309.91 |
| CL9_540592358PSV_F0610648CCAM01612P1 | Frenchville | 4 | 1612 | -4309.91 |
| CL9_540592554PSV_F0610648CCAM01612P1 | Frenchville | 5 | 1612 | -4309.91 |
| CL9_540771930PSV_F0610924CCAM01614P1 | Columbia_Falls | 1 | 1614 | -4306.86 |
| CL9_540771998PSV_F0610924CCAM01614P1 | Columbia_Falls | 2 | 1614 | -4306.86 |
| CL9_540772062PSV_F0610924CCAM01614P1 | Columbia_Falls | 3 | 1614 | -4306.86 |
| CL9_540772126PSV_F0610924CCAM01614P1 | Columbia_Falls | 4 | 1614 | -4306.86 |
| CL9_540772362PSV_F0610924CCAM01614P1 | Columbia_Falls | 5 | 1614 | -4306.86 |
| CL9_540772430PSV_F0610924CCAM01614P1 | Columbia_Falls | 6 | 1614 | -4306.86 |
| CL9_540772494PSV_F0610924CCAM01614P1 | Columbia_Falls | 7 | 1614 | -4306.86 |
| CL9_540772558PSV_F0610924CCAM01614P1 | Columbia_Falls | 8 | 1614 | -4306.86 |
| CL9_540772622PSV_F0610924CCAM01614P1 | Columbia_Falls | 9 | 1614 | -4306.86 |
| CL9_540772878PSV_F0610924CCAM01614P1 | Columbia_Falls | 10 | 1614 | -4306.86 |
| CL9_540773139PSV_F0610924CCAM01614P1 | Columbia_Falls | 14 | 1614 | -4306.86 |
| CL9_540773696PSV_F0610924CCAM02614P1 | Spider_Lake | 1 | 1614 | -4306.86 |
| CL9_540773765PSV_F0610924CCAM02614P1 | Spider_Lake | 2 | 1614 | -4306.86 |
| CL9_540773829PSV_F0610924CCAM02614P1 | Spider_Lake | 3 | 1614 | -4306.86 |
| CL9_540774032PSV_F0610924CCAM02614P1 | Spider_Lake | 4 | 1614 | -4306.86 |
| CL9_540774096PSV_F0610924CCAM02614P1 | Spider_Lake | 5 | 1614 | -4306.86 |
| CL9_540774160PSV_F0610924CCAM02614P1 | Spider_Lake | 6 | 1614 | -4306.86 |
| CL9_540774363PSV_F0610924CCAM02614P1 | Spider_Lake | 7 | 1614 | -4306.86 |
| CL9_540774427PSV_F0610924CCAM02614P1 | Spider_Lake | 8 | 1614 | -4306.86 |
| CL9_540775285PSV_F0610924CCAM03614P1 | Loon_Stream | 4 | 1614 | -4306.86 |
| CL9_540775349PSV_F0610924CCAM03614P1 | Loon_Stream | 5 | 1614 | -4306.86 |
| CL9_540775422PSV_F0610924CCAM03614P1 | Loon_Stream | 6 | 1614 | -4306.86 |
| CL9_540775632PSV_F0610924CCAM03614P1 | Loon_Stream | 7 | 1614 | -4306.86 |
| CL9_540775696PSV_F0610924CCAM03614P1 | Loon_Stream | 8 | 1614 | -4306.86 |
| CL9_540775866PSV_F0610924CCAM03614P1 | Loon_Stream | 9 | 1614 | -4306.86 |
| CL9_541310800PSV_F0611140CCAM01620P1 | Chase_Brook | 2 | 1620 | -4306.63 |
| CL9_541310866PSV_F0611140CCAM01620P1 | Chase_Brook | 3 | 1620 | -4306.63 |
| CL9_541311103PSV_F0611140CCAM01620P1 | Chase_Brook | 4 | 1620 | -4306.63 |
| CL9_541311179PSV_F0611140CCAM01620P1 | Chase_Brook | 5 | 1620 | -4306.63 |
| CL9_541311624PSV_F0611140CCAM01620P1 | Chase_Brook | 9 | 1620 | -4306.63 |
| CL9_541311821PSV_F0611140CCAM01620P1 | Chase_Brook | 10 | 1620 | -4306.63 |
| CL9_541312154PSV_F0611140CCAM02620P1 | Aziscohos | 1 | 1620 | -4306.63 |
| CL9_541312222PSV_F0611140CCAM02620P1 | Aziscohos | 2 | 1620 | -4306.63 |
| CL9_541313244PSV_F0611140CCAM03620P1 | Chandler_Ridge | 1 | 1620 | -4306.63 |
| CL9_541313319PSV_F0611140CCAM03620P1 | Chandler_Ridge | 2 | 1620 | -4306.63 |
| CL9_541313385PSV_F0611140CCAM03620P1 | Chandler_Ridge | 3 | 1620 | -4306.63 |
| CL9_541313611PSV_F0611140CCAM03620P1 | Chandler_Ridge | 4 | 1620 | -4306.63 |
| CL9_541313686PSV_F0611140CCAM03620P1 | Chandler_Ridge | 5 | 1620 | -4306.63 |
| CL9_541313752PSV_F0611140CCAM03620P1 | Chandler_Ridge | 6 | 1620 | -4306.63 |
| CL9_541313966PSV_F0611140CCAM03620P1 | Chandler_Ridge | 7 | 1620 | -4306.63 |
| CL9_541314041PSV_F0611140CCAM03620P1 | Chandler_Ridge | 8 | 1620 | -4306.63 |
| CL9_541314107PSV_F0611140CCAM03620P1 | Chandler_Ridge | 9 | 1620 | -4306.63 |
| CL9_541314281PSV_F0611140CCAM03620P1 | Chandler_Ridge | 10 | 1620 | -4306.63 |
| CL9_541575493PSV_F0611140CCAM01623P1 | Dunn_Brook | 1 | 1623 | -4306.63 |
| CL9_541575568PSV_F0611140CCAM01623P1 | Dunn_Brook | 2 | 1623 | -4306.63 |
| CL9_541575634PSV_F0611140CCAM01623P1 | Dunn_Brook | 3 | 1623 | -4306.63 |
| CL9_541575700PSV_F0611140CCAM01623P1 | Dunn_Brook | 4 | 1623 | -4306.63 |
| CL9_541575937PSV_F0611140CCAM01623P1 | Dunn_Brook | 5 | 1623 | -4306.63 |
| CL9_541576012PSV_F0611140CCAM01623P1 | Dunn_Brook | 6 | 1623 | -4306.63 |
| CL9_541576077PSV_F0611140CCAM01623P1 | Dunn_Brook | 7 | 1623 | -4306.63 |

| | | | | |
|---|---|---|---|---|
| CL9_541576143PSV_F0611140CCAM01623P1 | Dunn_Brook | 8 | 1623 | -4306.63 |
| CL9_541576209PSV_F0611140CCAM01623P1 | Dunn_Brook | 9 | 1623 | -4306.63 |
| CL9_541576405PSV_F0611140CCAM01623P1 | Dunn_Brook | 10 | 1623 | -4306.63 |
| CL9_541836890PSV_F0611140CCAM03625P1 | Allagash | 1 | 1626 | -4306.63 |
| CL9_541836965PSV_F0611140CCAM03625P1 | Allagash | 2 | 1626 | -4306.63 |
| CL9_541837031PSV_F0611140CCAM03625P1 | Allagash | 3 | 1626 | -4306.63 |
| CL9_541837260PSV_F0611140CCAM03625P1 | Allagash | 4 | 1626 | -4306.63 |
| CL9_541837328PSV_F0611140CCAM03625P1 | Allagash | 5 | 1626 | -4306.63 |
| CL9_541837394PSV_F0611140CCAM03625P1 | Allagash | 6 | 1626 | -4306.63 |
| CL9_541837624PSV_F0611140CCAM03625P1 | Allagash | 7 | 1626 | -4306.63 |
| CL9_541837692PSV_F0611140CCAM03625P1 | Allagash | 8 | 1626 | -4306.63 |
| CL9_541838345PSV_F0611140CCAM04625P1 | Hersey | 3 | 1626 | -4306.63 |
| CL9_541838411PSV_F0611140CCAM04625P1 | Hersey | 4 | 1626 | -4306.63 |
| CL9_541838648PSV_F0611140CCAM04625P1 | Hersey | 5 | 1626 | -4306.63 |
| CL9_541838724PSV_F0611140CCAM04625P1 | Hersey | 6 | 1626 | -4306.63 |
| CL9_541838856PSV_F0611140CCAM04625P1 | Hersey | 8 | 1626 | -4306.63 |
| CL9_541838922PSV_F0611140CCAM04625P1 | Hersey | 9 | 1626 | -4306.63 |
| CL9_541839119PSV_F0611140CCAM04625P1 | Hersey | 10 | 1626 | -4306.63 |
| CL9_541930332PSV_F0611140CCAM02627P1 | Rangeley | 1 | 1627 | -4306.63 |
| CL9_541930400PSV_F0611140CCAM02627P1 | Rangeley | 2 | 1627 | -4306.63 |
| CL9_541930464PSV_F0611140CCAM02627P1 | Rangeley | 3 | 1627 | -4306.63 |
| CL9_541930528PSV_F0611140CCAM02627P1 | Rangeley | 4 | 1627 | -4306.63 |
| CL9_541930784PSV_F0611140CCAM02627P1 | Rangeley | 5 | 1627 | -4306.63 |
| CL9_541930852PSV_F0611140CCAM02627P1 | Rangeley | 6 | 1627 | -4306.63 |
| CL9_541930916PSV_F0611140CCAM02627P1 | Rangeley | 7 | 1627 | -4306.63 |
| CL9_541930980PSV_F0611140CCAM02627P1 | Rangeley | 8 | 1627 | -4306.63 |
| CL9_541931044PSV_F0611140CCAM02627P1 | Rangeley | 9 | 1627 | -4306.63 |
| CL9_541931238PSV_F0611140CCAM02627P1 | Rangeley | 10 | 1627 | -4306.63 |
| CL9_542027822PSV_F0611140CCAM03627P1 | Thorofare | 1 | 1628 | -4306.63 |
| CL9_542027887PSV_F0611140CCAM03627P1 | Thorofare | 2 | 1628 | -4306.63 |
| CL9_542027951PSV_F0611140CCAM03627P1 | Thorofare | 3 | 1628 | -4306.63 |
| CL9_542028015PSV_F0611140CCAM03627P1 | Thorofare | 4 | 1628 | -4306.63 |
| CL9_542028228PSV_F0611140CCAM03627P1 | Thorofare | 5 | 1628 | -4306.63 |
| CL9_542028296PSV_F0611140CCAM03627P1 | Thorofare | 6 | 1628 | -4306.63 |
| CL9_542028360PSV_F0611140CCAM03627P1 | Thorofare | 7 | 1628 | -4306.63 |
| CL9_542028424PSV_F0611140CCAM03627P1 | Thorofare | 8 | 1628 | -4306.63 |
| CL9_542028660PSV_F0611140CCAM03627P1 | Thorofare | 10 | 1628 | -4306.63 |
| CL9_542197787PSV_F0611332CCAM01630P1 | Temple_Stream | 3 | 1630 | -4305.76 |
| CL9_542198033PSV_F0611332CCAM01630P1 | Temple_Stream | 4 | 1630 | -4305.76 |
| CL9_542198101PSV_F0611332CCAM01630P1 | Temple_Stream | 5 | 1630 | -4305.76 |
| CL9_542198165PSV_F0611332CCAM01630P1 | Temple_Stream | 6 | 1630 | -4305.76 |
| CL9_542198400PSV_F0611332CCAM01630P1 | Temple_Stream | 7 | 1630 | -4305.76 |
| CL9_542198532PSV_F0611332CCAM01630P1 | Temple_Stream | 9 | 1630 | -4305.76 |
| CL9_542198738PSV_F0611332CCAM01630P1 | Temple_Stream | 10 | 1630 | -4305.76 |
| CL9_542200049PSV_F0611332CCAM03630P1 | Vassalboro | 1 | 1630 | -4305.76 |
| CL9_542200117PSV_F0611332CCAM03630P1 | Vassalboro | 2 | 1630 | -4305.76 |
| CL9_542200181PSV_F0611332CCAM03630P1 | Vassalboro | 3 | 1630 | -4305.76 |
| CL9_542200412PSV_F0611332CCAM03630P1 | Vassalboro | 4 | 1630 | -4305.76 |
| CL9_542200477PSV_F0611332CCAM03630P1 | Vassalboro | 5 | 1630 | -4305.76 |
| CL9_542200541PSV_F0611332CCAM03630P1 | Vassalboro | 6 | 1630 | -4305.76 |
| CL9_542200774PSV_F0611332CCAM03630P1 | Vassalboro | 7 | 1630 | -4305.76 |
| CL9_542200839PSV_F0611332CCAM03630P1 | Vassalboro | 8 | 1630 | -4305.76 |
| CL9_542201033PSV_F0611332CCAM03630P1 | Vassalboro | 9 | 1630 | -4305.76 |
| CL9_542382925PSV_F0611650CCAM02632P1 | Nine_Lake | 1 | 1632 | -4304.56 |
| CL9_542382990PSV_F0611650CCAM02632P1 | Nine_Lake | 2 | 1632 | -4304.56 |
| CL9_542383054PSV_F0611650CCAM02632P1 | Nine_Lake | 3 | 1632 | -4304.56 |
| CL9_542383267PSV_F0611650CCAM02632P1 | Nine_Lake | 4 | 1632 | -4304.56 |
| CL9_542383330PSV_F0611650CCAM02632P1 | Nine_Lake | 5 | 1632 | -4304.56 |
| CL9_542383394PSV_F0611650CCAM02632P1 | Nine_Lake | 6 | 1632 | -4304.56 |

| | | | | |
|---|---|---|---|---|
| CL9_542383607PSV_F0611650CCAM02632P1 | Nine_Lake | 7 | 1632 | -4304.56 |
| CL9_542383671PSV_F0611650CCAM02632P1 | Nine_Lake | 8 | 1632 | -4304.56 |
| CL9_542383843PSV_F0611650CCAM02632P1 | Nine_Lake | 9 | 1632 | -4304.56 |
| CL9_542549001PSV_F0611908CCAM01634P1 | Hardwood_Mountain | 2 | 1634 | -4304.13 |
| CL9_542549853PSV_F0611908CCAM01634P1 | Hardwood_Mountain | 9 | 1634 | -4304.13 |
| CL9_542550062PSV_F0611908CCAM01634P1 | Hardwood_Mountain | 10 | 1634 | -4304.13 |
| CL9_542550423PSV_F0611908CCAM02634P1 | Hurricane_Mountain | 1 | 1634 | -4304.13 |
| CL9_542550491PSV_F0611908CCAM02634P1 | Hurricane_Mountain | 2 | 1634 | -4304.13 |
| CL9_542550555PSV_F0611908CCAM02634P1 | Hurricane_Mountain | 3 | 1634 | -4304.13 |
| CL9_542550620PSV_F0611908CCAM02634P1 | Hurricane_Mountain | 4 | 1634 | -4304.13 |
| CL9_542550813PSV_F0611908CCAM02634P1 | Hurricane_Mountain | 5 | 1634 | -4304.13 |
| CL9_542725215PSV_F0612148CCAM02636P1 | Buck_Cove_Mountain | 1 | 1636 | -4304.09 |
| CL9_542725290PSV_F0612148CCAM02636P1 | Buck_Cove_Mountain | 2 | 1636 | -4304.09 |
| CL9_542725356PSV_F0612148CCAM02636P1 | Buck_Cove_Mountain | 3 | 1636 | -4304.09 |
| CL9_542725422PSV_F0612148CCAM02636P1 | Buck_Cove_Mountain | 4 | 1636 | -4304.09 |
| CL9_542725586PSV_F0612148CCAM02636P1 | Buck_Cove_Mountain | 5 | 1636 | -4304.09 |
| CL9_542725985PSV_F0612148CCAM03636P1 | Smyrna_Mills | 2 | 1636 | -4304.09 |
| CL9_542726051PSV_F0612148CCAM03636P1 | Smyrna_Mills | 3 | 1636 | -4304.09 |
| CL9_542726255PSV_F0612148CCAM03636P1 | Smyrna_Mills | 4 | 1636 | -4304.09 |
| CL9_543087777PSV_F0612472CCAM03640P1 | Big_Moose_Mountain | 1 | 1640 | -4302.33 |
| CL9_543087845PSV_F0612472CCAM03640P1 | Big_Moose_Mountain | 2 | 1640 | -4302.33 |
| CL9_543087909PSV_F0612472CCAM03640P1 | Big_Moose_Mountain | 3 | 1640 | -4302.33 |
| CL9_543088212PSV_F0612472CCAM03640P1 | Big_Moose_Mountain | 5 | 1640 | -4302.33 |
| CL9_543088275PSV_F0612472CCAM03640P1 | Big_Moose_Mountain | 6 | 1640 | -4302.33 |
| CL9_543088510PSV_F0612472CCAM03640P1 | Big_Moose_Mountain | 7 | 1640 | -4302.33 |
| CL9_543088578PSV_F0612472CCAM03640P1 | Big_Moose_Mountain | 8 | 1640 | -4302.33 |
| CL9_543088641PSV_F0612472CCAM03640P1 | Big_Moose_Mountain | 9 | 1640 | -4302.33 |
| CL9_543088845PSV_F0612472CCAM03640P1 | Big_Moose_Mountain | 10 | 1640 | -4302.33 |
| CL9_543176815PSV_F0612472CCAM06640P1 | Frye_Island | 1 | 1641 | -4302.33 |
| CL9_543176883PSV_F0612472CCAM06640P1 | Frye_Island | 2 | 1641 | -4302.33 |
| CL9_543176946PSV_F0612472CCAM06640P1 | Frye_Island | 3 | 1641 | -4302.33 |
| CL9_543177010PSV_F0612472CCAM06640P1 | Frye_Island | 4 | 1641 | -4302.33 |
| CL9_543177074PSV_F0612472CCAM06640P1 | Frye_Island | 5 | 1641 | -4302.33 |
| CL9_543177137PSV_F0612472CCAM06640P1 | Frye_Island | 6 | 1641 | -4302.33 |
| CL9_543177201PSV_F0612472CCAM06640P1 | Frye_Island | 7 | 1641 | -4302.33 |
| CL9_543177264PSV_F0612472CCAM06640P1 | Frye_Island | 8 | 1641 | -4302.33 |
| CL9_543177327PSV_F0612472CCAM06640P1 | Frye_Island | 9 | 1641 | -4302.33 |
| CL9_543177521PSV_F0612472CCAM06640P1 | Frye_Island | 10 | 1641 | -4302.33 |
| CL9_543433645PSV_F0613076CCAM01644P1 | The_Hop_ccam | 1 | 1644 | -4300.34 |
| CL9_543433720PSV_F0613076CCAM01644P1 | The_Hop_ccam | 2 | 1644 | -4300.34 |
| CL9_543433786PSV_F0613076CCAM01644P1 | The_Hop_ccam | 3 | 1644 | -4300.34 |
| CL9_543434088PSV_F0613076CCAM01644P1 | The_Hop_ccam | 5 | 1644 | -4300.34 |
| CL9_543434163PSV_F0613076CCAM01644P1 | The_Hop_ccam | 6 | 1644 | -4300.34 |
| CL9_543434229PSV_F0613076CCAM01644P1 | The_Hop_ccam | 7 | 1644 | -4300.34 |
| CL9_543434295PSV_F0613076CCAM01644P1 | The_Hop_ccam | 8 | 1644 | -4300.34 |
| CL9_543434361PSV_F0613076CCAM01644P1 | The_Hop_ccam | 9 | 1644 | -4300.34 |
| CL9_543434557PSV_F0613076CCAM01644P1 | The_Hop_ccam | 10 | 1644 | -4300.34 |
| CL9_543522109PSV_F0613076CCAM01645P1 | Snows_Point | 1 | 1645 | -4300.34 |
| CL9_543522185PSV_F0613076CCAM01645P1 | Snows_Point | 2 | 1645 | -4300.34 |
| CL9_543522251PSV_F0613076CCAM01645P1 | Snows_Point | 3 | 1645 | -4300.34 |
| CL9_543522487PSV_F0613076CCAM01645P1 | Snows_Point | 4 | 1645 | -4300.34 |
| CL9_543522563PSV_F0613076CCAM01645P1 | Snows_Point | 5 | 1645 | -4300.34 |
| CL9_543522629PSV_F0613076CCAM01645P1 | Snows_Point | 6 | 1645 | -4300.34 |
| CL9_543522833PSV_F0613076CCAM01645P1 | Snows_Point | 7 | 1645 | -4300.34 |
| CL9_543522909PSV_F0613076CCAM01645P1 | Snows_Point | 8 | 1645 | -4300.34 |
| CL9_543522975PSV_F0613076CCAM01645P1 | Snows_Point | 9 | 1645 | -4300.34 |
| CL9_543523525PSV_F0613076CCAM02645P1 | Clam_Ledge | 1 | 1645 | -4300.34 |
| CL9_543523600PSV_F0613076CCAM02645P1 | Clam_Ledge | 2 | 1645 | -4300.34 |
| CL9_543523666PSV_F0613076CCAM02645P1 | Clam_Ledge | 3 | 1645 | -4300.34 |

| | | | | |
|---|---|---|---|---|
| CL9_543523863PSV_F0613076CCAM02645P1 | Clam_Ledge | 4 | 1645 | -4300.34 |
| CL9_543523929PSV_F0613076CCAM02645P1 | Clam_Ledge | 5 | 1645 | -4300.34 |
| CL9_543523995PSV_F0613076CCAM02645P1 | Clam_Ledge | 6 | 1645 | -4300.34 |
| CL9_543524193PSV_F0613076CCAM02645P1 | Clam_Ledge | 7 | 1645 | -4300.34 |
| CL9_543524259PSV_F0613076CCAM02645P1 | Clam_Ledge | 8 | 1645 | -4300.34 |
| CL9_543524422PSV_F0613076CCAM02645P1 | Clam_Ledge | 9 | 1645 | -4300.34 |
| CL9_543610609PSV_F0613226CCAM01646P1 | Bald_Rock_Ledge | 1 | 1646 | -4300 |
| CL9_543610750PSV_F0613226CCAM01646P1 | Bald_Rock_Ledge | 3 | 1646 | -4300 |
| CL9_543610986PSV_F0613226CCAM01646P1 | Bald_Rock_Ledge | 4 | 1646 | -4300 |
| CL9_543611061PSV_F0613226CCAM01646P1 | Bald_Rock_Ledge | 5 | 1646 | -4300 |
| CL9_543611364PSV_F0613226CCAM01646P1 | Bald_Rock_Ledge | 7 | 1646 | -4300 |
| CL9_543611439PSV_F0613226CCAM01646P1 | Bald_Rock_Ledge | 8 | 1646 | -4300 |
| CL9_543611669PSV_F0613226CCAM01646P1 | Bald_Rock_Ledge | 10 | 1646 | -4300 |
| CL9_543611992PSV_F0613226CCAM02646P1 | Porcupine_Dry_Ledge | 1 | 1646 | -4300 |
| CL9_543612060PSV_F0613226CCAM02646P1 | Porcupine_Dry_Ledge | 2 | 1646 | -4300 |
| CL9_543612124PSV_F0613226CCAM02646P1 | Porcupine_Dry_Ledge | 3 | 1646 | -4300 |
| CL9_543612390PSV_F0613226CCAM02646P1 | Porcupine_Dry_Ledge | 5 | 1646 | -4300 |
| CL9_545385104PSV_F0620690CCAM01666P1 | Ingalls_Island | 1 | 1666 | -4298.57 |
| CL9_545385180PSV_F0620690CCAM01666P1 | Ingalls_Island | 2 | 1666 | -4298.57 |
| CL9_545385246PSV_F0620690CCAM01666P1 | Ingalls_Island | 3 | 1666 | -4298.57 |
| CL9_545385625PSV_F0620690CCAM01666P1 | Ingalls_Island | 6 | 1666 | -4298.57 |
| CL9_545386200PSV_F0620690CCAM01666P1 | Ingalls_Island | 10 | 1666 | -4298.57 |
| CL9_545386546PSV_F0620690CCAM02666P1 | Yellow_Island | 1 | 1666 | -4298.57 |
| CL9_545387577PSV_F0620690CCAM03666P1 | Bunker_Cove | 1 | 1666 | -4298.57 |
| CL9_545387903PSV_F0620690CCAM03666P1 | Bunker_Cove | 4 | 1666 | -4298.57 |
| CL9_545388097PSV_F0620690CCAM03666P1 | Bunker_Cove | 5 | 1666 | -4298.57 |
| CL9_545388162PSV_F0620690CCAM03666P1 | Bunker_Cove | 6 | 1666 | -4298.57 |
| CL9_545388355PSV_F0620690CCAM03666P1 | Bunker_Cove | 7 | 1666 | -4298.57 |
| CL9_545388687PSV_F0620690CCAM03666P1 | Bunker_Cove | 10 | 1666 | -4298.57 |
| CL9_545388999PSV_F0620690CCAM04666P1 | Cromwell_Cove | 1 | 1666 | -4298.57 |
| CL9_545389067PSV_F0620690CCAM04666P1 | Cromwell_Cove | 2 | 1666 | -4298.57 |
| CL9_545389302PSV_F0620690CCAM04666P1 | Cromwell_Cove | 3 | 1666 | -4298.57 |
| CL9_545389370PSV_F0620690CCAM04666P1 | Cromwell_Cove | 4 | 1666 | -4298.57 |
| CL9_545389564PSV_F0620690CCAM04666P1 | Cromwell_Cove | 5 | 1666 | -4298.57 |
| CL9_545389629PSV_F0620690CCAM04666P1 | Cromwell_Cove | 6 | 1666 | -4298.57 |
| CL9_545389863PSV_F0620690CCAM04666P1 | Cromwell_Cove | 7 | 1666 | -4298.57 |
| CL9_545562373PSV_F0620786CCAM01668P1 | Sheldrake_Island_ccam | 1 | 1668 | -4298.05 |
| CL9_545562449PSV_F0620786CCAM01668P1 | Sheldrake_Island_ccam | 2 | 1668 | -4298.05 |
| CL9_545562515PSV_F0620786CCAM01668P1 | Sheldrake_Island_ccam | 3 | 1668 | -4298.05 |
| CL9_545562580PSV_F0620786CCAM01668P1 | Sheldrake_Island_ccam | 4 | 1668 | -4298.05 |
| CL9_545562743PSV_F0620786CCAM01668P1 | Sheldrake_Island_ccam | 5 | 1668 | -4298.05 |
| CL9_545563085PSV_F0620786CCAM02668P1 | crabtree_neck | 1 | 1668 | -4298.05 |
| CL9_545563160PSV_F0620786CCAM02668P1 | crabtree_neck | 2 | 1668 | -4298.05 |
| CL9_545563226PSV_F0620786CCAM02668P1 | crabtree_neck | 3 | 1668 | -4298.05 |
| CL9_545563915PSV_F0620786CCAM02668P1 | crabtree_neck | 8 | 1668 | -4298.05 |
| CL9_545563981PSV_F0620786CCAM02668P1 | crabtree_neck | 9 | 1668 | -4298.05 |
| CL9_545564177PSV_F0620786CCAM02668P1 | crabtree_neck | 10 | 1668 | -4298.05 |
| CL9_545564796PSV_F0620786CCAM03668P1 | Waukeah_neck | 1 | 1668 | -4298.05 |
| CL9_545564864PSV_F0620786CCAM03668P1 | Waukeah_neck | 2 | 1668 | -4298.05 |
| CL9_545564928PSV_F0620786CCAM03668P1 | Waukeah_neck | 3 | 1668 | -4298.05 |
| CL9_545564991PSV_F0620786CCAM03668P1 | Waukeah_neck | 4 | 1668 | -4298.05 |
| CL9_545565226PSV_F0620786CCAM03668P1 | Waukeah_neck | 5 | 1668 | -4298.05 |
| CL9_545565294PSV_F0620786CCAM03668P1 | Waukeah_neck | 6 | 1668 | -4298.05 |
| CL9_545565421PSV_F0620786CCAM03668P1 | Waukeah_neck | 8 | 1668 | -4298.05 |
| CL9_545565485PSV_F0620786CCAM03668P1 | Waukeah_neck | 9 | 1668 | -4298.05 |
| CL9_545565963PSV_F0620786CCAM04668P1 | Morancy_stream_ccam | 1 | 1668 | -4298.05 |
| CL9_545566028PSV_F0620786CCAM04668P1 | Morancy_stream_ccam | 2 | 1668 | -4298.05 |
| CL9_545566156PSV_F0620786CCAM04668P1 | Morancy_stream_ccam | 4 | 1668 | -4298.05 |
| CL9_545566220PSV_F0620786CCAM04668P1 | Morancy_stream_ccam | 5 | 1668 | -4298.05 |

| | | | | |
|---|---|---|---|---|
| CL9_545566822PSV_F0620786CCAM05668P1 | Ogden_Point | 1 | 1668 | -4298.05 |
| CL9_545566890PSV_F0620786CCAM05668P1 | Ogden_Point | 2 | 1668 | -4298.05 |
| CL9_545566954PSV_F0620786CCAM05668P1 | Ogden_Point | 3 | 1668 | -4298.05 |
| CL9_545567018PSV_F0620786CCAM05668P1 | Ogden_Point | 4 | 1668 | -4298.05 |
| CL9_545567212PSV_F0620786CCAM05668P1 | Ogden_Point | 5 | 1668 | -4298.05 |
| CL9_545842471PSV_F0621080CCAM01671P1 | calf_island | 3 | 1671 | -4296.42 |
| CL9_545842547PSV_F0621080CCAM01671P1 | calf_island | 4 | 1671 | -4296.42 |
| CL9_545843086PSV_F0621080CCAM02671P1 | deer_island_ccam | 1 | 1671 | -4296.42 |
| CL9_545843162PSV_F0621080CCAM02671P1 | deer_island_ccam | 2 | 1671 | -4296.42 |
| CL9_545843358PSV_F0621080CCAM02671P1 | deer_island_ccam | 3 | 1671 | -4296.42 |
| CL9_545843426PSV_F0621080CCAM02671P1 | deer_island_ccam | 4 | 1671 | -4296.42 |
| CL9_546006565PSV_F0621314CCAM01673P1 | sorrento_harbor | 1 | 1673 | -4295.08 |
| CL9_546006634PSV_F0621314CCAM01673P1 | sorrento_harbor | 2 | 1673 | -4295.08 |
| CL9_546006698PSV_F0621314CCAM01673P1 | sorrento_harbor | 3 | 1673 | -4295.08 |
| CL9_546006933PSV_F0621314CCAM01673P1 | sorrento_harbor | 4 | 1673 | -4295.08 |
| CL9_546008230PSV_F0621314CCAM02673P1 | bean_point | 4 | 1673 | -4295.08 |
| CL9_546008437PSV_F0621314CCAM02673P1 | bean_point | 5 | 1673 | -4295.08 |
| CL9_546008789PSV_F0621314CCAM03673P1 | chimney_peak | 1 | 1673 | -4295.08 |
| CL9_546008857PSV_F0621314CCAM03673P1 | chimney_peak | 2 | 1673 | -4295.08 |
| CL9_546008921PSV_F0621314CCAM03673P1 | chimney_peak | 3 | 1673 | -4295.08 |
| CL9_546009229PSV_F0621314CCAM03673P1 | chimney_peak | 5 | 1673 | -4295.08 |
| CL9_546009293PSV_F0621314CCAM03673P1 | chimney_peak | 6 | 1673 | -4295.08 |
| CL9_546009527PSV_F0621314CCAM03673P1 | chimney_peak | 7 | 1673 | -4295.08 |
| CL9_546009596PSV_F0621314CCAM03673P1 | chimney_peak | 8 | 1673 | -4295.08 |
| CL9_546009660PSV_F0621314CCAM03673P1 | chimney_peak | 9 | 1673 | -4295.08 |
| CL9_546010177PSV_F0621314CCAM04673P1 | cape_levi | 1 | 1673 | -4295.08 |
| CL9_546010245PSV_F0621314CCAM04673P1 | cape_levi | 2 | 1673 | -4295.08 |
| CL9_546010309PSV_F0621314CCAM04673P1 | cape_levi | 3 | 1673 | -4295.08 |
| CL9_546010373PSV_F0621314CCAM04673P1 | cape_levi | 4 | 1673 | -4295.08 |
| CL9_546010566PSV_F0621314CCAM04673P1 | cape_levi | 5 | 1673 | -4295.08 |
| CL9_546103346PSV_F0621386CCAM01674P1 | South_Brother | 7 | 1674 | -4294.63 |
| CL9_546103411PSV_F0621386CCAM01674P1 | South_Brother | 8 | 1674 | -4294.63 |
| CL9_546103606PSV_F0621386CCAM01674P1 | South_Brother | 9 | 1674 | -4294.63 |
| CL9_546103989PSV_F0621386CCAM02674P1 | Lookout_Point | 1 | 1674 | -4294.63 |
| CL9_546104057PSV_F0621386CCAM02674P1 | Lookout_Point | 2 | 1674 | -4294.63 |
| CL9_546104121PSV_F0621386CCAM02674P1 | Lookout_Point | 3 | 1674 | -4294.63 |
| CL9_546104362PSV_F0621386CCAM02674P1 | Lookout_Point | 4 | 1674 | -4294.63 |
| CL9_546104431PSV_F0621386CCAM02674P1 | Lookout_Point | 5 | 1674 | -4294.63 |
| CL9_546104495PSV_F0621386CCAM02674P1 | Lookout_Point | 6 | 1674 | -4294.63 |
| CL9_546104737PSV_F0621386CCAM02674P1 | Lookout_Point | 7 | 1674 | -4294.63 |
| CL9_546104806PSV_F0621386CCAM02674P1 | Lookout_Point | 8 | 1674 | -4294.63 |
| CL9_546105013PSV_F0621386CCAM02674P1 | Lookout_Point | 9 | 1674 | -4294.63 |
| CL9_546105341PSV_F0621386CCAM03674P1 | Back_Cove | 1 | 1674 | -4294.63 |
| CL9_546105406PSV_F0621386CCAM03674P1 | Back_Cove | 2 | 1674 | -4294.63 |
| CL9_546105469PSV_F0621386CCAM03674P1 | Back_Cove | 3 | 1674 | -4294.63 |
| CL9_546105532PSV_F0621386CCAM03674P1 | Back_Cove | 4 | 1674 | -4294.63 |
| CL9_546105605PSV_F0621386CCAM03674P1 | Back_Cove | 5 | 1674 | -4294.63 |
| CL9_546105668PSV_F0621386CCAM03674P1 | Back_Cove | 6 | 1674 | -4294.63 |
| CL9_546105731PSV_F0621386CCAM03674P1 | Back_Cove | 7 | 1674 | -4294.63 |
| CL9_546105795PSV_F0621386CCAM03674P1 | Back_Cove | 8 | 1674 | -4294.63 |
| CL9_546105858PSV_F0621386CCAM03674P1 | Back_Cove | 9 | 1674 | -4294.63 |
| CL9_546106052PSV_F0621386CCAM03674P1 | Back_Cove | 10 | 1674 | -4294.63 |
| CL9_546366910PSV_F0621530CCAM01677P1 | Casco_Bay_ccam | 1 | 1677 | -4293.77 |
| CL9_546366985PSV_F0621530CCAM01677P1 | Casco_Bay_ccam | 2 | 1677 | -4293.77 |
| CL9_546367051PSV_F0621530CCAM01677P1 | Casco_Bay_ccam | 3 | 1677 | -4293.77 |
| CL9_546367117PSV_F0621530CCAM01677P1 | Casco_Bay_ccam | 4 | 1677 | -4293.77 |
| CL9_546367313PSV_F0621530CCAM01677P1 | Casco_Bay_ccam | 5 | 1677 | -4293.77 |
| CL9_546450571PSV_F0621776CCAM01678P1 | Hancock_Point | 1 | 1678 | -4291.64 |
| CL9_546450639PSV_F0621776CCAM01678P1 | Hancock_Point | 2 | 1678 | -4291.64 |

| | | | | |
|---|---|---|---|---|
| CL9_546450703PSV_F0621776CCAM01678P1 | Hancock_Point | 3 | 1678 | -4291.64 |
| CL9_546450961PSV_F0621776CCAM01678P1 | Hancock_Point | 5 | 1678 | -4291.64 |
| CL9_546451026PSV_F0621776CCAM01678P1 | Hancock_Point | 6 | 1678 | -4291.64 |
| CL9_546451090PSV_F0621776CCAM01678P1 | Hancock_Point | 7 | 1678 | -4291.64 |
| CL9_546451153PSV_F0621776CCAM01678P1 | Hancock_Point | 8 | 1678 | -4291.64 |
| CL9_546451217PSV_F0621776CCAM01678P1 | Hancock_Point | 9 | 1678 | -4291.64 |
| CL9_546451411PSV_F0621776CCAM01678P1 | Hancock_Point | 10 | 1678 | -4291.64 |
| CL9_546451764PSV_F0621776CCAM02678P1 | Crocker_Mountain | 1 | 1678 | -4291.64 |
| CL9_546451832PSV_F0621776CCAM02678P1 | Crocker_Mountain | 2 | 1678 | -4291.64 |
| CL9_546451895PSV_F0621776CCAM02678P1 | Crocker_Mountain | 3 | 1678 | -4291.64 |
| CL9_546451958PSV_F0621776CCAM02678P1 | Crocker_Mountain | 4 | 1678 | -4291.64 |
| CL9_546452152PSV_F0621776CCAM02678P1 | Crocker_Mountain | 5 | 1678 | -4291.64 |
| CL9_546452217PSV_F0621776CCAM02678P1 | Crocker_Mountain | 6 | 1678 | -4291.64 |
| CL9_546452280PSV_F0621776CCAM02678P1 | Crocker_Mountain | 7 | 1678 | -4291.64 |
| CL9_546452343PSV_F0621776CCAM02678P1 | Crocker_Mountain | 8 | 1678 | -4291.64 |
| CL9_546452406PSV_F0621776CCAM02678P1 | Crocker_Mountain | 9 | 1678 | -4291.64 |
| CL9_546452600PSV_F0621776CCAM02678P1 | Crocker_Mountain | 10 | 1678 | -4291.64 |
| CL9_546544976PSV_F0622026CCAM01679P1 | Maple_Spring_ccam | 1 | 1679 | -4289.65 |
| CL9_546545044PSV_F0622026CCAM01679P1 | Maple_Spring_ccam | 2 | 1679 | -4289.65 |
| CL9_546545108PSV_F0622026CCAM01679P1 | Maple_Spring_ccam | 3 | 1679 | -4289.65 |
| CL9_546545172PSV_F0622026CCAM01679P1 | Maple_Spring_ccam | 4 | 1679 | -4289.65 |
| CL9_546545374PSV_F0622026CCAM01679P1 | Maple_Spring_ccam | 5 | 1679 | -4289.65 |
| CL9_546627482PSV_F0622248CCAM01680P1 | Trenton_Bridge | 1 | 1680 | -4287.69 |
| CL9_546627550PSV_F0622248CCAM01680P1 | Trenton_Bridge | 2 | 1680 | -4287.69 |
| CL9_546627614PSV_F0622248CCAM01680P1 | Trenton_Bridge | 3 | 1680 | -4287.69 |
| CL9_546627677PSV_F0622248CCAM01680P1 | Trenton_Bridge | 4 | 1680 | -4287.69 |
| CL9_546627871PSV_F0622248CCAM01680P1 | Trenton_Bridge | 5 | 1680 | -4287.69 |
| CL9_546628944PSV_F0622248CCAM03680P1 | Beach_Cliff | 1 | 1680 | -4287.69 |
| CL9_546629012PSV_F0622248CCAM03680P1 | Beach_Cliff | 2 | 1680 | -4287.69 |
| CL9_546629076PSV_F0622248CCAM03680P1 | Beach_Cliff | 3 | 1680 | -4287.69 |
| CL9_546629140PSV_F0622248CCAM03680P1 | Beach_Cliff | 4 | 1680 | -4287.69 |
| CL9_546629346PSV_F0622248CCAM03680P1 | Beach_Cliff | 5 | 1680 | -4287.69 |
| CL9_546629668PSV_F0622248CCAM04680P1 | Browns_Brook | 1 | 1680 | -4287.69 |
| CL9_546629744PSV_F0622248CCAM04680P1 | Browns_Brook | 2 | 1680 | -4287.69 |
| CL9_546629875PSV_F0622248CCAM04680P1 | Browns_Brook | 4 | 1680 | -4287.69 |
| CL9_546630071PSV_F0622248CCAM04680P1 | Browns_Brook | 5 | 1680 | -4287.69 |
| CL9_546716444PSV_F0622452CCAM01681P1 | Duck_Brook_Bridge_ccam | 1 | 1681 | -4286.24 |
| CL9_546716519PSV_F0622452CCAM01681P1 | Duck_Brook_Bridge_ccam | 2 | 1681 | -4286.24 |
| CL9_546716585PSV_F0622452CCAM01681P1 | Duck_Brook_Bridge_ccam | 3 | 1681 | -4286.24 |
| CL9_546716682PSV_F0622452CCAM01681P1 | Duck_Brook_Bridge_ccam | 4 | 1681 | -4286.24 |
| CL9_546716748PSV_F0622452CCAM01681P1 | Duck_Brook_Bridge_ccam | 5 | 1681 | -4286.24 |
| CL9_546716814PSV_F0622452CCAM01681P1 | Duck_Brook_Bridge_ccam | 6 | 1681 | -4286.24 |
| CL9_546716912PSV_F0622452CCAM01681P1 | Duck_Brook_Bridge_ccam | 7 | 1681 | -4286.24 |
| CL9_546716978PSV_F0622452CCAM01681P1 | Duck_Brook_Bridge_ccam | 8 | 1681 | -4286.24 |
| CL9_546717142PSV_F0622452CCAM01681P1 | Duck_Brook_Bridge_ccam | 9 | 1681 | -4286.24 |
| CL9_546717476PSV_F0622452CCAM02681P1 | Waterfall_Bridge | 1 | 1681 | -4286.24 |
| CL9_546717552PSV_F0622452CCAM02681P1 | Waterfall_Bridge | 2 | 1681 | -4286.24 |
| CL9_546717618PSV_F0622452CCAM02681P1 | Waterfall_Bridge | 3 | 1681 | -4286.24 |
| CL9_546717874PSV_F0622452CCAM02681P1 | Waterfall_Bridge | 4 | 1681 | -4286.24 |
| CL9_546717950PSV_F0622452CCAM02681P1 | Waterfall_Bridge | 5 | 1681 | -4286.24 |
| CL9_546718016PSV_F0622452CCAM02681P1 | Waterfall_Bridge | 6 | 1681 | -4286.24 |
| CL9_546718272PSV_F0622452CCAM02681P1 | Waterfall_Bridge | 7 | 1681 | -4286.24 |
| CL9_546718348PSV_F0622452CCAM02681P1 | Waterfall_Bridge | 8 | 1681 | -4286.24 |
| CL9_546718414PSV_F0622452CCAM02681P1 | Waterfall_Bridge | 9 | 1681 | -4286.24 |
| CL9_546718674PSV_F0622452CCAM02681P1 | Waterfall_Bridge | 10 | 1681 | -4286.24 |
| CL9_546718750PSV_F0622452CCAM02681P1 | Waterfall_Bridge | 11 | 1681 | -4286.24 |
| CL9_546718816PSV_F0622452CCAM02681P1 | Waterfall_Bridge | 12 | 1681 | -4286.24 |
| CL9_546719068PSV_F0622452CCAM02681P1 | Waterfall_Bridge | 13 | 1681 | -4286.24 |
| CL9_546719144PSV_F0622452CCAM02681P1 | Waterfall_Bridge | 14 | 1681 | -4286.24 |

| | | | | |
|---|---|---|---|---|
| CL9_546719210PSV_F0622452CCAM02681P1 | Waterfall_Bridge | 15 | 1681 | -4286.24 |
| CL9_546719469PSV_F0622452CCAM02681P1 | Waterfall_Bridge | 16 | 1681 | -4286.24 |
| CL9_546719545PSV_F0622452CCAM02681P1 | Waterfall_Bridge | 17 | 1681 | -4286.24 |
| CL9_546719611PSV_F0622452CCAM02681P1 | Waterfall_Bridge | 18 | 1681 | -4286.24 |
| CL9_546719790PSV_F0622452CCAM02681P1 | Waterfall_Bridge | 19 | 1681 | -4286.24 |
| CL9_546808729PSV_F0622452CCAM03681P1 | Cobblestone_Bridge | 1 | 1681 | -4286.24 |
| CL9_546808804PSV_F0622452CCAM03681P1 | Cobblestone_Bridge | 2 | 1681 | -4286.24 |
| CL9_546808870PSV_F0622452CCAM03681P1 | Cobblestone_Bridge | 3 | 1681 | -4286.24 |
| CL9_546808968PSV_F0622452CCAM03681P1 | Cobblestone_Bridge | 4 | 1681 | -4286.24 |
| CL9_546809034PSV_F0622452CCAM03681P1 | Cobblestone_Bridge | 5 | 1681 | -4286.24 |
| CL9_546809100PSV_F0622452CCAM03681P1 | Cobblestone_Bridge | 6 | 1681 | -4286.24 |
| CL9_546809328PSV_F0622452CCAM03681P1 | Cobblestone_Bridge | 7 | 1681 | -4286.24 |
| CL9_546809396PSV_F0622452CCAM03681P1 | Cobblestone_Bridge | 8 | 1681 | -4286.24 |
| CL9_546809592PSV_F0622452CCAM03681P1 | Cobblestone_Bridge | 9 | 1681 | -4286.24 |
| CL9_546809904PSV_F0622452CCAM04681P1 | Amphitheater_Bridge | 1 | 1681 | -4286.24 |
| CL9_546809979PSV_F0622452CCAM04681P1 | Amphitheater_Bridge | 2 | 1681 | -4286.24 |
| CL9_546810045PSV_F0622452CCAM04681P1 | Amphitheater_Bridge | 3 | 1681 | -4286.24 |
| CL9_546810111PSV_F0622452CCAM04681P1 | Amphitheater_Bridge | 4 | 1681 | -4286.24 |
| CL9_546810306PSV_F0622452CCAM04681P1 | Amphitheater_Bridge | 5 | 1681 | -4286.24 |
| CL9_546983162PSV_F0622726CCAM01684P1 | Cow_Ledge | 1 | 1684 | -4284.58 |
| CL9_546983230PSV_F0622726CCAM01684P1 | Cow_Ledge | 2 | 1684 | -4284.58 |
| CL9_546983293PSV_F0622726CCAM01684P1 | Cow_Ledge | 3 | 1684 | -4284.58 |
| CL9_546983357PSV_F0622726CCAM01684P1 | Cow_Ledge | 4 | 1684 | -4284.58 |
| CL9_546983421PSV_F0622726CCAM01684P1 | Cow_Ledge | 5 | 1684 | -4284.58 |
| CL9_546983485PSV_F0622726CCAM01684P1 | Cow_Ledge | 6 | 1684 | -4284.58 |
| CL9_546983549PSV_F0622726CCAM01684P1 | Cow_Ledge | 7 | 1684 | -4284.58 |
| CL9_546983613PSV_F0622726CCAM01684P1 | Cow_Ledge | 8 | 1684 | -4284.58 |
| CL9_546983677PSV_F0622726CCAM01684P1 | Cow_Ledge | 9 | 1684 | -4284.58 |
| CL9_546983879PSV_F0622726CCAM01684P1 | Cow_Ledge | 10 | 1684 | -4284.58 |
| CL9_546984208PSV_F0622726CCAM02684P1 | Carter_Cove | 1 | 1684 | -4284.58 |
| CL9_546984273PSV_F0622726CCAM02684P1 | Carter_Cove | 2 | 1684 | -4284.58 |
| CL9_546984337PSV_F0622726CCAM02684P1 | Carter_Cove | 3 | 1684 | -4284.58 |
| CL9_546984401PSV_F0622726CCAM02684P1 | Carter_Cove | 4 | 1684 | -4284.58 |
| CL9_546984465PSV_F0622726CCAM02684P1 | Carter_Cove | 5 | 1684 | -4284.58 |
| CL9_546984529PSV_F0622726CCAM02684P1 | Carter_Cove | 6 | 1684 | -4284.58 |
| CL9_546984593PSV_F0622726CCAM02684P1 | Carter_Cove | 7 | 1684 | -4284.58 |
| CL9_546984657PSV_F0622726CCAM02684P1 | Carter_Cove | 8 | 1684 | -4284.58 |
| CL9_546984720PSV_F0622726CCAM02684P1 | Carter_Cove | 9 | 1684 | -4284.58 |
| CL9_546984914PSV_F0622726CCAM02684P1 | Carter_Cove | 10 | 1684 | -4284.58 |
| CL9_547165477PSV_F0623188CCAM01686P1 | Newport_Ledge_ccam | 1 | 1686 | -4280.85 |
| CL9_547165545PSV_F0623188CCAM01686P1 | Newport_Ledge_ccam | 2 | 1686 | -4280.85 |
| CL9_547165609PSV_F0623188CCAM01686P1 | Newport_Ledge_ccam | 3 | 1686 | -4280.85 |
| CL9_547165673PSV_F0623188CCAM01686P1 | Newport_Ledge_ccam | 4 | 1686 | -4280.85 |
| CL9_547165867PSV_F0623188CCAM01686P1 | Newport_Ledge_ccam | 5 | 1686 | -4280.85 |
| CL9_547436005PSV_F0623350CCAM03688P1 | Denning_Brook | 1 | 1689 | -4280.45 |
| CL9_547436081PSV_F0623350CCAM03688P1 | Denning_Brook | 2 | 1689 | -4280.45 |
| CL9_547436147PSV_F0623350CCAM03688P1 | Denning_Brook | 3 | 1689 | -4280.45 |
| CL9_547436384PSV_F0623350CCAM03688P1 | Denning_Brook | 4 | 1689 | -4280.45 |
| CL9_547436460PSV_F0623350CCAM03688P1 | Denning_Brook | 5 | 1689 | -4280.45 |
| CL9_547436526PSV_F0623350CCAM03688P1 | Denning_Brook | 6 | 1689 | -4280.45 |
| CL9_547436762PSV_F0623350CCAM03688P1 | Denning_Brook | 7 | 1689 | -4280.45 |
| CL9_547436837PSV_F0623350CCAM03688P1 | Denning_Brook | 8 | 1689 | -4280.45 |
| CL9_547436903PSV_F0623350CCAM03688P1 | Denning_Brook | 9 | 1689 | -4280.45 |
| CL9_547437067PSV_F0623350CCAM03688P1 | Denning_Brook | 10 | 1689 | -4280.45 |
| CL9_547608576PSV_F0630000CCAM01691P1 | Green_Nubble | 1 | 1691 | -4279.75 |
| CL9_547608651PSV_F0630000CCAM01691P1 | Green_Nubble | 2 | 1691 | -4279.75 |
| CL9_547608717PSV_F0630000CCAM01691P1 | Green_Nubble | 3 | 1691 | -4279.75 |
| CL9_547608974PSV_F0630000CCAM01691P1 | Green_Nubble | 4 | 1691 | -4279.75 |
| CL9_547609050PSV_F0630000CCAM01691P1 | Green_Nubble | 5 | 1691 | -4279.75 |

| | | | | |
|---|---|---|---|---|
| CL9_547609494PSV_F0630000CCAM01691P1 | Green_Nubble | 9 | 1691 | -4279.75 |
| CL9_547609690PSV_F0630000CCAM01691P1 | Green_Nubble | 10 | 1691 | -4279.75 |
| CL9_547697664PSV_F0630100CCAM01692P1 | The_Cleft | 1 | 1692 | -4279.4 |
| CL9_547697732PSV_F0630100CCAM01692P1 | The_Cleft | 2 | 1692 | -4279.4 |
| CL9_547697796PSV_F0630100CCAM01692P1 | The_Cleft | 3 | 1692 | -4279.4 |
| CL9_547697860PSV_F0630100CCAM01692P1 | The_Cleft | 4 | 1692 | -4279.4 |
| CL9_547698055PSV_F0630100CCAM01692P1 | The_Cleft | 5 | 1692 | -4279.4 |
| CL9_547698397PSV_F0630100CCAM02692P1 | Weaver_Rock | 1 | 1692 | -4279.4 |
| CL9_547698465PSV_F0630100CCAM02692P1 | Weaver_Rock | 2 | 1692 | -4279.4 |
| CL9_547698529PSV_F0630100CCAM02692P1 | Weaver_Rock | 3 | 1692 | -4279.4 |
| CL9_547698593PSV_F0630100CCAM02692P1 | Weaver_Rock | 4 | 1692 | -4279.4 |
| CL9_547698657PSV_F0630100CCAM02692P1 | Weaver_Rock | 5 | 1692 | -4279.4 |
| CL9_547698892PSV_F0630100CCAM02692P1 | Weaver_Rock | 6 | 1692 | -4279.4 |
| CL9_547698960PSV_F0630100CCAM02692P1 | Weaver_Rock | 7 | 1692 | -4279.4 |
| CL9_547699024PSV_F0630100CCAM02692P1 | Weaver_Rock | 8 | 1692 | -4279.4 |
| CL9_547699088PSV_F0630100CCAM02692P1 | Weaver_Rock | 9 | 1692 | -4279.4 |
| CL9_547699216PSV_F0630100CCAM02692P1 | Weaver_Rock | 11 | 1692 | -4279.4 |
| CL9_547699539PSV_F0630100CCAM02692P1 | Weaver_Rock | 13 | 1692 | -4279.4 |
| CL9_547699603PSV_F0630100CCAM02692P1 | Weaver_Rock | 14 | 1692 | -4279.4 |
| CL9_547699667PSV_F0630100CCAM02692P1 | Weaver_Rock | 15 | 1692 | -4279.4 |
| CL9_547699731PSV_F0630100CCAM02692P1 | Weaver_Rock | 16 | 1692 | -4279.4 |
| CL9_547700041PSV_F0630100CCAM02692P1 | Weaver_Rock | 18 | 1692 | -4279.4 |
| CL9_547700105PSV_F0630100CCAM02692P1 | Weaver_Rock | 19 | 1692 | -4279.4 |
| CL9_547700299PSV_F0630100CCAM02692P1 | Weaver_Rock | 20 | 1692 | -4279.4 |
| CL9_547700619PSV_F0630100CCAM03692P1 | The_Maypole | 1 | 1692 | -4279.4 |
| CL9_547700693PSV_F0630100CCAM03692P1 | The_Maypole | 2 | 1692 | -4279.4 |
| CL9_547700757PSV_F0630100CCAM03692P1 | The_Maypole | 3 | 1692 | -4279.4 |
| CL9_547700821PSV_F0630100CCAM03692P1 | The_Maypole | 4 | 1692 | -4279.4 |
| CL9_547701015PSV_F0630100CCAM03692P1 | The_Maypole | 5 | 1692 | -4279.4 |
| CL9_547786125PSV_F0630100CCAM03693P1 | Bear_Island_1 | 1 | 1693 | -4279.4 |
| CL9_547786194PSV_F0630100CCAM03693P1 | Bear_Island_1 | 2 | 1693 | -4279.4 |
| CL9_547786258PSV_F0630100CCAM03693P1 | Bear_Island_1 | 3 | 1693 | -4279.4 |
| CL9_547786514PSV_F0630100CCAM03693P1 | Bear_Island_1 | 4 | 1693 | -4279.4 |
| CL9_547786646PSV_F0630100CCAM03693P1 | Bear_Island_1 | 6 | 1693 | -4279.4 |
| CL9_547786901PSV_F0630100CCAM03693P1 | Bear_Island_1 | 7 | 1693 | -4279.4 |
| CL9_547786969PSV_F0630100CCAM03693P1 | Bear_Island_1 | 8 | 1693 | -4279.4 |
| CL9_547787033PSV_F0630100CCAM03693P1 | Bear_Island_1 | 9 | 1693 | -4279.4 |
| CL9_547787227PSV_F0630100CCAM03693P1 | Bear_Island_1 | 10 | 1693 | -4279.4 |
| CL9_547787540PSV_F0630100CCAM04693P1 | Bear_Island_2 | 1 | 1693 | -4279.4 |
| CL9_547787669PSV_F0630100CCAM04693P1 | Bear_Island_2 | 3 | 1693 | -4279.4 |
| CL9_547787733PSV_F0630100CCAM04693P1 | Bear_Island_2 | 4 | 1693 | -4279.4 |
| CL9_547787927PSV_F0630100CCAM04693P1 | Bear_Island_2 | 5 | 1693 | -4279.4 |
| CL9_547960448PSV_F0630346CCAM01695P1 | Mason_Point_ccam | 1 | 1695 | -4279.19 |
| CL9_547960523PSV_F0630346CCAM01695P1 | Mason_Point_ccam | 2 | 1695 | -4279.19 |
| CL9_547960589PSV_F0630346CCAM01695P1 | Mason_Point_ccam | 3 | 1695 | -4279.19 |
| CL9_547960690PSV_F0630346CCAM01695P1 | Mason_Point_ccam | 4 | 1695 | -4279.19 |
| CL9_547960756PSV_F0630346CCAM01695P1 | Mason_Point_ccam | 5 | 1695 | -4279.19 |
| CL9_547960822PSV_F0630346CCAM01695P1 | Mason_Point_ccam | 6 | 1695 | -4279.19 |
| CL9_547960924PSV_F0630346CCAM01695P1 | Mason_Point_ccam | 7 | 1695 | -4279.19 |
| CL9_547960990PSV_F0630346CCAM01695P1 | Mason_Point_ccam | 8 | 1695 | -4279.19 |
| CL9_547961186PSV_F0630346CCAM01695P1 | Mason_Point_ccam | 9 | 1695 | -4279.19 |
| CL9_547961575PSV_F0630346CCAM02695P1 | Mitchell_Hill | 2 | 1695 | -4279.19 |
| CL9_547961640PSV_F0630346CCAM02695P1 | Mitchell_Hill | 3 | 1695 | -4279.19 |
| CL9_547961877PSV_F0630346CCAM02695P1 | Mitchell_Hill | 4 | 1695 | -4279.19 |
| CL9_547962017PSV_F0630346CCAM02695P1 | Mitchell_Hill | 6 | 1695 | -4279.19 |
| CL9_547962253PSV_F0630346CCAM02695P1 | Mitchell_Hill | 7 | 1695 | -4279.19 |
| CL9_547962557PSV_F0630346CCAM02695P1 | Mitchell_Hill | 10 | 1695 | -4279.19 |
| CL9_547962910PSV_F0630346CCAM03695P1 | Mount_Gilboa | 1 | 1695 | -4279.19 |
| CL9_547962985PSV_F0630346CCAM03695P1 | Mount_Gilboa | 2 | 1695 | -4279.19 |

| | | | | |
|---|---|---|---|---|
| CL9_547963050PSV_F0630346CCAM03695P1 | Mount_Gilboa | 3 | 1695 | -4279.19 |
| CL9_547963115PSV_F0630346CCAM03695P1 | Mount_Gilboa | 4 | 1695 | -4279.19 |
| CL9_547963352PSV_F0630346CCAM03695P1 | Mount_Gilboa | 5 | 1695 | -4279.19 |
| CL9_547963427PSV_F0630346CCAM03695P1 | Mount_Gilboa | 6 | 1695 | -4279.19 |
| CL9_547963493PSV_F0630346CCAM03695P1 | Mount_Gilboa | 7 | 1695 | -4279.19 |
| CL9_547963558PSV_F0630346CCAM03695P1 | Mount_Gilboa | 8 | 1695 | -4279.19 |
| CL9_547963624PSV_F0630346CCAM03695P1 | Mount_Gilboa | 9 | 1695 | -4279.19 |
| CL9_547963820PSV_F0630346CCAM03695P1 | Mount_Gilboa | 10 | 1695 | -4279.19 |
| CL9_548239073PSV_F0630766CCAM01698P1 | Spurling_Rock | 1 | 1698 | -4275.58 |
| CL9_548239148PSV_F0630766CCAM01698P1 | Spurling_Rock | 2 | 1698 | -4275.58 |
| CL9_548239214PSV_F0630766CCAM01698P1 | Spurling_Rock | 3 | 1698 | -4275.58 |
| CL9_548239280PSV_F0630766CCAM01698P1 | Spurling_Rock | 4 | 1698 | -4275.58 |
| CL9_548239476PSV_F0630766CCAM01698P1 | Spurling_Rock | 5 | 1698 | -4275.58 |
| CL9_548240969PSV_F0630766CCAM03698P1 | Knight_Nubble | 1 | 1698 | -4275.58 |
| CL9_548241045PSV_F0630766CCAM03698P1 | Knight_Nubble | 2 | 1698 | -4275.58 |
| CL9_548241111PSV_F0630766CCAM03698P1 | Knight_Nubble | 3 | 1698 | -4275.58 |
| CL9_548241177PSV_F0630766CCAM03698P1 | Knight_Nubble | 4 | 1698 | -4275.58 |
| CL9_548241373PSV_F0630766CCAM03698P1 | Knight_Nubble | 5 | 1698 | -4275.58 |
| CL9_548581419PSV_F0631420CCAM02702P1 | Redfield_Hill | 1 | 1702 | -4270.38 |
| CL9_548581487PSV_F0631420CCAM02702P1 | Redfield_Hill | 2 | 1702 | -4270.38 |
| CL9_548581551PSV_F0631420CCAM02702P1 | Redfield_Hill | 3 | 1702 | -4270.38 |
| CL9_548581615PSV_F0631420CCAM02702P1 | Redfield_Hill | 4 | 1702 | -4270.38 |
| CL9_548581679PSV_F0631420CCAM02702P1 | Redfield_Hill | 5 | 1702 | -4270.38 |
| CL9_548581742PSV_F0631420CCAM02702P1 | Redfield_Hill | 6 | 1702 | -4270.38 |
| CL9_548581806PSV_F0631420CCAM02702P1 | Redfield_Hill | 7 | 1702 | -4270.38 |
| CL9_548581870PSV_F0631420CCAM02702P1 | Redfield_Hill | 8 | 1702 | -4270.38 |
| CL9_548581934PSV_F0631420CCAM02702P1 | Redfield_Hill | 9 | 1702 | -4270.38 |
| CL9_548582095PSV_F0631420CCAM02702P1 | Redfield_Hill | 10 | 1702 | -4270.38 |
| CL9_548582378PSV_F0631420CCAM03702P1 | Pulpit_Ledge_ccam | 1 | 1702 | -4270.38 |
| CL9_548582444PSV_F0631420CCAM03702P1 | Pulpit_Ledge_ccam | 2 | 1702 | -4270.38 |
| CL9_548582510PSV_F0631420CCAM03702P1 | Pulpit_Ledge_ccam | 3 | 1702 | -4270.38 |
| CL9_548582714PSV_F0631420CCAM03702P1 | Pulpit_Ledge_ccam | 4 | 1702 | -4270.38 |
| CL9_548582789PSV_F0631420CCAM03702P1 | Pulpit_Ledge_ccam | 5 | 1702 | -4270.38 |
| CL9_548582855PSV_F0631420CCAM03702P1 | Pulpit_Ledge_ccam | 6 | 1702 | -4270.38 |
| CL9_548583058PSV_F0631420CCAM03702P1 | Pulpit_Ledge_ccam | 7 | 1702 | -4270.38 |
| CL9_548583130PSV_F0631420CCAM03702P1 | Pulpit_Ledge_ccam | 8 | 1702 | -4270.38 |
| CL9_548583196PSV_F0631420CCAM03702P1 | Pulpit_Ledge_ccam | 9 | 1702 | -4270.38 |
| CL9_548583360PSV_F0631420CCAM03702P1 | Pulpit_Ledge_ccam | 10 | 1702 | -4270.38 |
| CL9_548671488PSV_F0631420CCAM04702P1 | Fern_Spring | 1 | 1703 | -4270.38 |
| CL9_548671563PSV_F0631420CCAM04702P1 | Fern_Spring | 2 | 1703 | -4270.38 |
| CL9_548671629PSV_F0631420CCAM04702P1 | Fern_Spring | 3 | 1703 | -4270.38 |
| CL9_548671866PSV_F0631420CCAM04702P1 | Fern_Spring | 4 | 1703 | -4270.38 |
| CL9_548672007PSV_F0631420CCAM04702P1 | Fern_Spring | 6 | 1703 | -4270.38 |
| CL9_548672242PSV_F0631420CCAM04702P1 | Fern_Spring | 7 | 1703 | -4270.38 |
| CL9_548672317PSV_F0631420CCAM04702P1 | Fern_Spring | 8 | 1703 | -4270.38 |
| CL9_548672383PSV_F0631420CCAM04702P1 | Fern_Spring | 9 | 1703 | -4270.38 |
| CL9_548672579PSV_F0631420CCAM04702P1 | Fern_Spring | 10 | 1703 | -4270.38 |
| CL9_548848507PSV_F0631450CCAM01705P1 | Turtle_Island | 1 | 1705 | -4269.91 |
| CL9_548848576PSV_F0631450CCAM01705P1 | Turtle_Island | 2 | 1705 | -4269.91 |
| CL9_548848640PSV_F0631450CCAM01705P1 | Turtle_Island | 3 | 1705 | -4269.91 |
| CL9_548848874PSV_F0631450CCAM01705P1 | Turtle_Island | 4 | 1705 | -4269.91 |
| CL9_548848943PSV_F0631450CCAM01705P1 | Turtle_Island | 5 | 1705 | -4269.91 |
| CL9_548849007PSV_F0631450CCAM01705P1 | Turtle_Island | 6 | 1705 | -4269.91 |
| CL9_548849241PSV_F0631450CCAM01705P1 | Turtle_Island | 7 | 1705 | -4269.91 |
| CL9_548849310PSV_F0631450CCAM01705P1 | Turtle_Island | 8 | 1705 | -4269.91 |
| CL9_548849374PSV_F0631450CCAM01705P1 | Turtle_Island | 9 | 1705 | -4269.91 |
| CL9_548849705PSV_F0631450CCAM02705P1 | Stony_Brook | 1 | 1705 | -4269.91 |
| CL9_548849781PSV_F0631450CCAM02705P1 | Stony_Brook | 2 | 1705 | -4269.91 |
| CL9_548849847PSV_F0631450CCAM02705P1 | Stony_Brook | 3 | 1705 | -4269.91 |

| | | | | |
|---|---|---|---|---|
| CL9_548850098PSV_F0631450CCAM02705P1 | Stony_Brook | 4 | 1705 | -4269.91 |
| CL9_548850174PSV_F0631450CCAM02705P1 | Stony_Brook | 5 | 1705 | -4269.91 |
| CL9_548850385PSV_F0631450CCAM02705P1 | Stony_Brook | 6 | 1705 | -4269.91 |
| CL9_548850461PSV_F0631450CCAM02705P1 | Stony_Brook | 7 | 1705 | -4269.91 |
| CL9_548850713PSV_F0631450CCAM02705P1 | Stony_Brook | 8 | 1705 | -4269.91 |
| CL9_548850789PSV_F0631450CCAM02705P1 | Stony_Brook | 9 | 1705 | -4269.91 |
| CL9_548850967PSV_F0631450CCAM02705P1 | Stony_Brook | 10 | 1705 | -4269.91 |
| CL9_549027020PSV_F0631636CCAM01707P1 | White_Cap_Mountain | 10 | 1707 | -4268.98 |
| CL9_549302673PSV_F0631840CCAM01709P1 | White_Ledge_ccam | 1 | 1710 | -4267.57 |
| CL9_549302742PSV_F0631840CCAM01709P1 | White_Ledge_ccam | 2 | 1710 | -4267.57 |
| CL9_549302806PSV_F0631840CCAM01709P1 | White_Ledge_ccam | 3 | 1710 | -4267.57 |
| CL9_549303040PSV_F0631840CCAM01709P1 | White_Ledge_ccam | 4 | 1710 | -4267.57 |
| CL9_549303108PSV_F0631840CCAM01709P1 | White_Ledge_ccam | 5 | 1710 | -4267.57 |
| CL9_549303172PSV_F0631840CCAM01709P1 | White_Ledge_ccam | 6 | 1710 | -4267.57 |
| CL9_549303406PSV_F0631840CCAM01709P1 | White_Ledge_ccam | 7 | 1710 | -4267.57 |
| CL9_549303474PSV_F0631840CCAM01709P1 | White_Ledge_ccam | 8 | 1710 | -4267.57 |
| CL9_549303538PSV_F0631840CCAM01709P1 | White_Ledge_ccam | 9 | 1710 | -4267.57 |
| CL9_549303732PSV_F0631840CCAM01709P1 | White_Ledge_ccam | 10 | 1710 | -4267.57 |
| CL9_549304160PSV_F0631840CCAM02709P1 | Shooting_Ledge | 2 | 1710 | -4267.57 |
| CL9_549304549PSV_F0631840CCAM02709P1 | Shooting_Ledge | 5 | 1710 | -4267.57 |
| CL9_549385223PSV_F0631840CCAM04709P1 | Middle_Ledge | 1 | 1711 | -4267.57 |
| CL9_549385291PSV_F0631840CCAM04709P1 | Middle_Ledge | 2 | 1711 | -4267.57 |
| CL9_549385355PSV_F0631840CCAM04709P1 | Middle_Ledge | 3 | 1711 | -4267.57 |
| CL9_549385419PSV_F0631840CCAM04709P1 | Middle_Ledge | 4 | 1711 | -4267.57 |
| CL9_549385613PSV_F0631840CCAM04709P1 | Middle_Ledge | 5 | 1711 | -4267.57 |
| CL9_549472419PSV_F0632008CCAM01712P1 | Ned_Island | 1 | 1712 | -4266.58 |
| CL9_549472867PSV_F0632008CCAM01712P1 | Ned_Island | 8 | 1712 | -4266.58 |
| CL9_549472930PSV_F0632008CCAM01712P1 | Ned_Island | 9 | 1712 | -4266.58 |
| CL9_549473485PSV_F0632008CCAM02712P1 | Ravens_Nest | 1 | 1712 | -4266.58 |
| CL9_549473560PSV_F0632008CCAM02712P1 | Ravens_Nest | 2 | 1712 | -4266.58 |
| CL9_549473626PSV_F0632008CCAM02712P1 | Ravens_Nest | 3 | 1712 | -4266.58 |
| CL9_549473862PSV_F0632008CCAM02712P1 | Ravens_Nest | 4 | 1712 | -4266.58 |
| CL9_549473937PSV_F0632008CCAM02712P1 | Ravens_Nest | 5 | 1712 | -4266.58 |
| CL9_549474003PSV_F0632008CCAM02712P1 | Ravens_Nest | 6 | 1712 | -4266.58 |
| CL9_549474239PSV_F0632008CCAM02712P1 | Ravens_Nest | 7 | 1712 | -4266.58 |
| CL9_549474314PSV_F0632008CCAM02712P1 | Ravens_Nest | 8 | 1712 | -4266.58 |
| CL9_549474380PSV_F0632008CCAM02712P1 | Ravens_Nest | 9 | 1712 | -4266.58 |
| CL9_549474575PSV_F0632008CCAM02712P1 | Ravens_Nest | 10 | 1712 | -4266.58 |
| CL9_549649876PSV_F0632086CCAM01714P1 | Heron_Island | 1 | 1714 | -4265.51 |
| CL9_549649944PSV_F0632086CCAM01714P1 | Heron_Island | 2 | 1714 | -4265.51 |
| CL9_549650008PSV_F0632086CCAM01714P1 | Heron_Island | 3 | 1714 | -4265.51 |
| CL9_549650072PSV_F0632086CCAM01714P1 | Heron_Island | 4 | 1714 | -4265.51 |
| CL9_549650318PSV_F0632086CCAM01714P1 | Heron_Island | 5 | 1714 | -4265.51 |
| CL9_549650386PSV_F0632086CCAM01714P1 | Heron_Island | 6 | 1714 | -4265.51 |
| CL9_549650450PSV_F0632086CCAM01714P1 | Heron_Island | 7 | 1714 | -4265.51 |
| CL9_549650514PSV_F0632086CCAM01714P1 | Heron_Island | 8 | 1714 | -4265.51 |
| CL9_549650578PSV_F0632086CCAM01714P1 | Heron_Island | 9 | 1714 | -4265.51 |
| CL9_549650781PSV_F0632086CCAM01714P1 | Heron_Island | 10 | 1714 | -4265.51 |
| CL9_549651111PSV_F0632086CCAM02714P1 | McNeil_Point | 1 | 1714 | -4265.51 |
| CL9_549651179PSV_F0632086CCAM02714P1 | McNeil_Point | 2 | 1714 | -4265.51 |
| CL9_549651305PSV_F0632086CCAM02714P1 | McNeil_Point | 4 | 1714 | -4265.51 |
| CL9_549651539PSV_F0632086CCAM02714P1 | McNeil_Point | 5 | 1714 | -4265.51 |
| CL9_549651607PSV_F0632086CCAM02714P1 | McNeil_Point | 6 | 1714 | -4265.51 |
| CL9_549651734PSV_F0632086CCAM02714P1 | McNeil_Point | 8 | 1714 | -4265.51 |
| CL9_549651798PSV_F0632086CCAM02714P1 | McNeil_Point | 9 | 1714 | -4265.51 |
| CL9_549651992PSV_F0632086CCAM02714P1 | McNeil_Point | 10 | 1714 | -4265.51 |
| CL9_549745845PSV_F0632086CCAM01715P1 | Old_Mill_Brook_ccam | 1 | 1715 | -4265.51 |
| CL9_549745909PSV_F0632086CCAM01715P1 | Old_Mill_Brook_ccam | 2 | 1715 | -4265.51 |
| CL9_549746142PSV_F0632086CCAM01715P1 | Old_Mill_Brook_ccam | 3 | 1715 | -4265.51 |

| | | | | |
|---|---|---|---|---|
| CL9_549746207PSV_F0632086CCAM01715P1 | Old_Mill_Brook_ccam | 4 | 1715 | -4265.51 |
| CL9_549746271PSV_F0632086CCAM01715P1 | Old_Mill_Brook_ccam | 5 | 1715 | -4265.51 |
| CL9_549746504PSV_F0632086CCAM01715P1 | Old_Mill_Brook_ccam | 6 | 1715 | -4265.51 |
| CL9_549746569PSV_F0632086CCAM01715P1 | Old_Mill_Brook_ccam | 7 | 1715 | -4265.51 |
| CL9_549746763PSV_F0632086CCAM01715P1 | Old_Mill_Brook_ccam | 8 | 1715 | -4265.51 |
| CL9_549747081PSV_F0632086CCAM02715P1 | Timber_Point_ccam | 1 | 1715 | -4265.51 |
| CL9_549747149PSV_F0632086CCAM02715P1 | Timber_Point_ccam | 2 | 1715 | -4265.51 |
| CL9_549747213PSV_F0632086CCAM02715P1 | Timber_Point_ccam | 3 | 1715 | -4265.51 |
| CL9_549747446PSV_F0632086CCAM02715P1 | Timber_Point_ccam | 4 | 1715 | -4265.51 |
| CL9_549747511PSV_F0632086CCAM02715P1 | Timber_Point_ccam | 5 | 1715 | -4265.51 |
| CL9_549747575PSV_F0632086CCAM02715P1 | Timber_Point_ccam | 6 | 1715 | -4265.51 |
| CL9_549747808PSV_F0632086CCAM02715P1 | Timber_Point_ccam | 7 | 1715 | -4265.51 |
| CL9_549747873PSV_F0632086CCAM02715P1 | Timber_Point_ccam | 8 | 1715 | -4265.51 |
| CL9_549748067PSV_F0632086CCAM02715P1 | Timber_Point_ccam | 9 | 1715 | -4265.51 |
| CL9_549833831PSV_F0632086CCAM03715P1 | Spectacle_Island | 1 | 1716 | -4265.51 |
| CL9_549833899PSV_F0632086CCAM03715P1 | Spectacle_Island | 2 | 1716 | -4265.51 |
| CL9_549833963PSV_F0632086CCAM03715P1 | Spectacle_Island | 3 | 1716 | -4265.51 |
| CL9_549834027PSV_F0632086CCAM03715P1 | Spectacle_Island | 4 | 1716 | -4265.51 |
| CL9_549834261PSV_F0632086CCAM03715P1 | Spectacle_Island | 5 | 1716 | -4265.51 |
| CL9_549834329PSV_F0632086CCAM03715P1 | Spectacle_Island | 6 | 1716 | -4265.51 |
| CL9_549834393PSV_F0632086CCAM03715P1 | Spectacle_Island | 7 | 1716 | -4265.51 |
| CL9_549834457PSV_F0632086CCAM03715P1 | Spectacle_Island | 8 | 1716 | -4265.51 |
| CL9_549834521PSV_F0632086CCAM03715P1 | Spectacle_Island | 9 | 1716 | -4265.51 |
| CL9_549834714PSV_F0632086CCAM03715P1 | Spectacle_Island | 10 | 1716 | -4265.51 |
| CL9_549835077PSV_F0632086CCAM04715P1 | Goose_Eye_Mountain_ccam | 1 | 1716 | -4265.51 |
| CL9_549835145PSV_F0632086CCAM04715P1 | Goose_Eye_Mountain_ccam | 2 | 1716 | -4265.51 |
| CL9_549835218PSV_F0632086CCAM04715P1 | Goose_Eye_Mountain_ccam | 3 | 1716 | -4265.51 |
| CL9_549835453PSV_F0632086CCAM04715P1 | Goose_Eye_Mountain_ccam | 4 | 1716 | -4265.51 |
| CL9_549835521PSV_F0632086CCAM04715P1 | Goose_Eye_Mountain_ccam | 5 | 1716 | -4265.51 |
| CL9_549835585PSV_F0632086CCAM04715P1 | Goose_Eye_Mountain_ccam | 6 | 1716 | -4265.51 |
| CL9_549835820PSV_F0632086CCAM04715P1 | Goose_Eye_Mountain_ccam | 7 | 1716 | -4265.51 |
| CL9_549835888PSV_F0632086CCAM04715P1 | Goose_Eye_Mountain_ccam | 8 | 1716 | -4265.51 |
| CL9_549835952PSV_F0632086CCAM04715P1 | Goose_Eye_Mountain_ccam | 9 | 1716 | -4265.51 |
| CL9_549836145PSV_F0632086CCAM04715P1 | Goose_Eye_Mountain_ccam | 10 | 1716 | -4265.51 |
| CL9_550001601PSV_F0632372CCAM01718P1 | Eastern_Point_Harbor | 1 | 1718 | -4262.49 |
| CL9_550001742PSV_F0632372CCAM01718P1 | Eastern_Point_Harbor | 3 | 1718 | -4262.49 |
| CL9_550001808PSV_F0632372CCAM01718P1 | Eastern_Point_Harbor | 4 | 1718 | -4262.49 |
| CL9_550002324PSV_F0632372CCAM02718P1 | East_Pond | 1 | 1718 | -4262.49 |
| CL9_550002457PSV_F0632372CCAM02718P1 | East_Pond | 3 | 1718 | -4262.49 |
| CL9_550002521PSV_F0632372CCAM02718P1 | East_Pond | 4 | 1718 | -4262.49 |
| CL9_550002756PSV_F0632372CCAM02718P1 | East_Pond | 5 | 1718 | -4262.49 |
| CL9_550002825PSV_F0632372CCAM02718P1 | East_Pond | 6 | 1718 | -4262.49 |
| CL9_550002889PSV_F0632372CCAM02718P1 | East_Pond | 7 | 1718 | -4262.49 |
| CL9_550002953PSV_F0632372CCAM02718P1 | East_Pond | 8 | 1718 | -4262.49 |
| CL9_550003634PSV_F0632372CCAM03718P1 | East_Point | 2 | 1718 | -4262.49 |
| CL9_550095343PSV_F0632582CCAM01719P1 | Wonsqueak_Harbor | 1 | 1719 | -4260.24 |
| CL9_550095411PSV_F0632582CCAM01719P1 | Wonsqueak_Harbor | 2 | 1719 | -4260.24 |
| CL9_550095475PSV_F0632582CCAM01719P1 | Wonsqueak_Harbor | 3 | 1719 | -4260.24 |
| CL9_550095538PSV_F0632582CCAM01719P1 | Wonsqueak_Harbor | 4 | 1719 | -4260.24 |
| CL9_550095732PSV_F0632582CCAM01719P1 | Wonsqueak_Harbor | 5 | 1719 | -4260.24 |
| CL9_550179638PSV_F0632672CCAM01720P1 | Marlboro_Beach | 1 | 1720 | -4260.23 |
| CL9_550179714PSV_F0632672CCAM01720P1 | Marlboro_Beach | 2 | 1720 | -4260.23 |
| CL9_550179780PSV_F0632672CCAM01720P1 | Marlboro_Beach | 3 | 1720 | -4260.23 |
| CL9_550180017PSV_F0632672CCAM01720P1 | Marlboro_Beach | 4 | 1720 | -4260.23 |
| CL9_550180093PSV_F0632672CCAM01720P1 | Marlboro_Beach | 5 | 1720 | -4260.23 |
| CL9_550180159PSV_F0632672CCAM01720P1 | Marlboro_Beach | 6 | 1720 | -4260.23 |
| CL9_550180396PSV_F0632672CCAM01720P1 | Marlboro_Beach | 7 | 1720 | -4260.23 |
| CL9_550180472PSV_F0632672CCAM01720P1 | Marlboro_Beach | 8 | 1720 | -4260.23 |
| CL9_550180538PSV_F0632672CCAM01720P1 | Marlboro_Beach | 9 | 1720 | -4260.23 |

| | | | | |
|---|---|---|---|---|
| CL9_550180702PSV_F0632672CCAM01720P1 | Marlboro_Beach | 10 | 1720 | -4260.23 |
| CL9_550181922PSV_F0632672CCAM02720P1 | Lamoine_Beach | 8 | 1720 | -4260.23 |
| CL9_550181988PSV_F0632672CCAM02720P1 | Lamoine_Beach | 9 | 1720 | -4260.23 |
| CL9_550182183PSV_F0632672CCAM02720P1 | Lamoine_Beach | 10 | 1720 | -4260.23 |
| CL9_550275403PSV_F0632978CCAM01721P1 | Kief_Pond | 1 | 1721 | -4257.31 |
| CL9_550275479PSV_F0632978CCAM01721P1 | Kief_Pond | 2 | 1721 | -4257.31 |
| CL9_550275544PSV_F0632978CCAM01721P1 | Kief_Pond | 3 | 1721 | -4257.31 |
| CL9_550275610PSV_F0632978CCAM01721P1 | Kief_Pond | 4 | 1721 | -4257.31 |
| CL9_550275806PSV_F0632978CCAM01721P1 | Kief_Pond | 5 | 1721 | -4257.31 |
| CL9_550533168PSV_F0633092CCAM01722P1 | Old_Point | 1 | 1724 | -4255.55 |
| CL9_550533236PSV_F0633092CCAM01722P1 | Old_Point | 2 | 1724 | -4255.55 |
| CL9_550533301PSV_F0633092CCAM01722P1 | Old_Point | 3 | 1724 | -4255.55 |
| CL9_550533534PSV_F0633092CCAM01722P1 | Old_Point | 4 | 1724 | -4255.55 |
| CL9_550533666PSV_F0633092CCAM01722P1 | Old_Point | 6 | 1724 | -4255.55 |
| CL9_550533794PSV_F0633092CCAM01722P1 | Old_Point | 8 | 1724 | -4255.55 |
| CL9_550533858PSV_F0633092CCAM01722P1 | Old_Point | 9 | 1724 | -4255.55 |
| CL9_550534052PSV_F0633092CCAM01722P1 | Old_Point | 10 | 1724 | -4255.55 |
| CL9_550629055PSV_F0633326CCAM01725P1 | Hayne_Point_ccam | 1 | 1725 | -4253.95 |
| CL9_550629130PSV_F0633326CCAM01725P1 | Hayne_Point_ccam | 2 | 1725 | -4253.95 |
| CL9_550629196PSV_F0633326CCAM01725P1 | Hayne_Point_ccam | 3 | 1725 | -4253.95 |
| CL9_550629444PSV_F0633326CCAM01725P1 | Hayne_Point_ccam | 4 | 1725 | -4253.95 |
| CL9_550629519PSV_F0633326CCAM01725P1 | Hayne_Point_ccam | 5 | 1725 | -4253.95 |
| CL9_550629585PSV_F0633326CCAM01725P1 | Hayne_Point_ccam | 6 | 1725 | -4253.95 |
| CL9_550629833PSV_F0633326CCAM01725P1 | Hayne_Point_ccam | 7 | 1725 | -4253.95 |
| CL9_550629908PSV_F0633326CCAM01725P1 | Hayne_Point_ccam | 8 | 1725 | -4253.95 |
| CL9_550629974PSV_F0633326CCAM01725P1 | Hayne_Point_ccam | 9 | 1725 | -4253.95 |
| CL9_550630178PSV_F0633326CCAM01725P1 | Hayne_Point_ccam | 10 | 1725 | -4253.95 |
| CL9_550716157PSV_F0633326CCAM01726P1 | Mount_Abraham | 1 | 1726 | -4253.95 |
| CL9_550716232PSV_F0633326CCAM01726P1 | Mount_Abraham | 2 | 1726 | -4253.95 |
| CL9_550716298PSV_F0633326CCAM01726P1 | Mount_Abraham | 3 | 1726 | -4253.95 |
| CL9_550716687PSV_F0633326CCAM01726P1 | Mount_Abraham | 6 | 1726 | -4253.95 |
| CL9_550717606PSV_F0633326CCAM02726P1 | Mount_Redington | 1 | 1726 | -4253.95 |
| CL9_550717681PSV_F0633326CCAM02726P1 | Mount_Redington | 2 | 1726 | -4253.95 |
| CL9_550717747PSV_F0633326CCAM02726P1 | Mount_Redington | 3 | 1726 | -4253.95 |
| CL9_550981491PSV_F0640420CCAM01729P1 | Mark_Island | 1 | 1729 | -4247.8 |
| CL9_550981566PSV_F0640420CCAM01729P1 | Mark_Island | 2 | 1729 | -4247.8 |
| CL9_550981632PSV_F0640420CCAM01729P1 | Mark_Island | 3 | 1729 | -4247.8 |
| CL9_550981869PSV_F0640420CCAM01729P1 | Mark_Island | 4 | 1729 | -4247.8 |
| CL9_550981944PSV_F0640420CCAM01729P1 | Mark_Island | 5 | 1729 | -4247.8 |
| CL9_550982010PSV_F0640420CCAM01729P1 | Mark_Island | 6 | 1729 | -4247.8 |
| CL9_550982388PSV_F0640420CCAM01729P1 | Mark_Island | 9 | 1729 | -4247.8 |
| CL9_550982584PSV_F0640420CCAM01729P1 | Mark_Island | 10 | 1729 | -4247.8 |
| CL9_550982928PSV_F0640420CCAM02729P1 | Frazer_Creek_ccam | 1 | 1729 | -4247.8 |
| CL9_550983003PSV_F0640420CCAM02729P1 | Frazer_Creek_ccam | 2 | 1729 | -4247.8 |
| CL9_550983068PSV_F0640420CCAM02729P1 | Frazer_Creek_ccam | 3 | 1729 | -4247.8 |
| CL9_550983134PSV_F0640420CCAM02729P1 | Frazer_Creek_ccam | 4 | 1729 | -4247.8 |
| CL9_550983330PSV_F0640420CCAM02729P1 | Frazer_Creek_ccam | 5 | 1729 | -4247.8 |
| CL9_551250025PSV_F0640678CCAM01732P1 | Pierce_Head_ccam | 1 | 1732 | -4247.29 |
| CL9_551250100PSV_F0640678CCAM01732P1 | Pierce_Head_ccam | 2 | 1732 | -4247.29 |
| CL9_551250166PSV_F0640678CCAM01732P1 | Pierce_Head_ccam | 3 | 1732 | -4247.29 |
| CL9_551250266PSV_F0640678CCAM01732P1 | Pierce_Head_ccam | 4 | 1732 | -4247.29 |
| CL9_551250429PSV_F0640678CCAM01732P1 | Pierce_Head_ccam | 5 | 1732 | -4247.29 |
| CL9_551250495PSV_F0640678CCAM01732P1 | Pierce_Head_ccam | 6 | 1732 | -4247.29 |
| CL9_551250595PSV_F0640678CCAM01732P1 | Pierce_Head_ccam | 7 | 1732 | -4247.29 |
| CL9_551250661PSV_F0640678CCAM01732P1 | Pierce_Head_ccam | 8 | 1732 | -4247.29 |
| CL9_551250825PSV_F0640678CCAM01732P1 | Pierce_Head_ccam | 9 | 1732 | -4247.29 |
| CL9_551251204PSV_F0640678CCAM02732P1 | Mosely_Point | 1 | 1732 | -4247.29 |
| CL9_551251279PSV_F0640678CCAM02732P1 | Mosely_Point | 2 | 1732 | -4247.29 |
| CL9_551251345PSV_F0640678CCAM02732P1 | Mosely_Point | 3 | 1732 | -4247.29 |

| | | | | |
|---|---|---|---|---|
| CL9_551251411PSV_F0640678CCAM02732P1 | Mosely_Point | 4 | 1732 | -4247.29 |
| CL9_551251607PSV_F0640678CCAM02732P1 | Mosely_Point | 5 | 1732 | -4247.29 |
| CL9_551251935PSV_F0640678CCAM03732P1 | Leland_Point | 1 | 1732 | -4247.29 |
| CL9_551252003PSV_F0640678CCAM03732P1 | Leland_Point | 2 | 1732 | -4247.29 |
| CL9_551252067PSV_F0640678CCAM03732P1 | Leland_Point | 3 | 1732 | -4247.29 |
| CL9_551252131PSV_F0640678CCAM03732P1 | Leland_Point | 4 | 1732 | -4247.29 |
| CL9_551252195PSV_F0640678CCAM03732P1 | Leland_Point | 5 | 1732 | -4247.29 |
| CL9_551252430PSV_F0640678CCAM03732P1 | Leland_Point | 6 | 1732 | -4247.29 |
| CL9_551252498PSV_F0640678CCAM03732P1 | Leland_Point | 7 | 1732 | -4247.29 |
| CL9_551252562PSV_F0640678CCAM03732P1 | Leland_Point | 8 | 1732 | -4247.29 |
| CL9_551252626PSV_F0640678CCAM03732P1 | Leland_Point | 9 | 1732 | -4247.29 |
| CL9_551252820PSV_F0640678CCAM03732P1 | Leland_Point | 10 | 1732 | -4247.29 |
| CL9_551428896PSV_F0640846CCAM01734P1 | Pecks_Point | 1 | 1734 | -4246.16 |
| CL9_551428971PSV_F0640846CCAM01734P1 | Pecks_Point | 2 | 1734 | -4246.16 |
| CL9_551429037PSV_F0640846CCAM01734P1 | Pecks_Point | 3 | 1734 | -4246.16 |
| CL9_551429275PSV_F0640846CCAM01734P1 | Pecks_Point | 4 | 1734 | -4246.16 |
| CL9_551429350PSV_F0640846CCAM01734P1 | Pecks_Point | 5 | 1734 | -4246.16 |
| CL9_551429416PSV_F0640846CCAM01734P1 | Pecks_Point | 6 | 1734 | -4246.16 |
| CL9_551429653PSV_F0640846CCAM01734P1 | Pecks_Point | 7 | 1734 | -4246.16 |
| CL9_551429728PSV_F0640846CCAM01734P1 | Pecks_Point | 8 | 1734 | -4246.16 |
| CL9_551429794PSV_F0640846CCAM01734P1 | Pecks_Point | 9 | 1734 | -4246.16 |
| CL9_551429990PSV_F0640846CCAM01734P1 | Pecks_Point | 10 | 1734 | -4246.16 |
| CL9_551603744PSV_F0640996CCAM01736P1 | Winter_Harbor_ccam | 1 | 1736 | -4246.41 |
| CL9_551603819PSV_F0640996CCAM01736P1 | Winter_Harbor_ccam | 2 | 1736 | -4246.41 |
| CL9_551603885PSV_F0640996CCAM01736P1 | Winter_Harbor_ccam | 3 | 1736 | -4246.41 |
| CL9_551603983PSV_F0640996CCAM01736P1 | Winter_Harbor_ccam | 4 | 1736 | -4246.41 |
| CL9_551604187PSV_F0640996CCAM01736P1 | Winter_Harbor_ccam | 5 | 1736 | -4246.41 |
| CL9_551604259PSV_F0640996CCAM01736P1 | Winter_Harbor_ccam | 6 | 1736 | -4246.41 |
| CL9_551604358PSV_F0640996CCAM01736P1 | Winter_Harbor_ccam | 7 | 1736 | -4246.41 |
| CL9_551604424PSV_F0640996CCAM01736P1 | Winter_Harbor_ccam | 8 | 1736 | -4246.41 |
| CL9_551604628PSV_F0640996CCAM01736P1 | Winter_Harbor_ccam | 9 | 1736 | -4246.41 |
| CL9_551604969PSV_F0640996CCAM02736P1 | Beaver_Dam_Pond | 1 | 1736 | -4246.41 |
| CL9_551605037PSV_F0640996CCAM02736P1 | Beaver_Dam_Pond | 2 | 1736 | -4246.41 |
| CL9_551605101PSV_F0640996CCAM02736P1 | Beaver_Dam_Pond | 3 | 1736 | -4246.41 |
| CL9_551605269PSV_F0640996CCAM02736P1 | Beaver_Dam_Pond | 5 | 1736 | -4246.41 |
| CL9_551605563PSV_F0640996CCAM02736P1 | Beaver_Dam_Pond | 7 | 1736 | -4246.41 |
| CL9_551605628PSV_F0640996CCAM02736P1 | Beaver_Dam_Pond | 8 | 1736 | -4246.41 |
| CL9_551605822PSV_F0640996CCAM02736P1 | Beaver_Dam_Pond | 9 | 1736 | -4246.41 |
| CL9_551606189PSV_F0640996CCAM03736P1 | Kitteredge_Brook | 1 | 1736 | -4246.41 |
| CL9_551606397PSV_F0640996CCAM03736P1 | Kitteredge_Brook | 4 | 1736 | -4246.41 |
| CL9_551607137PSV_F0640996CCAM03736P1 | Kitteredge_Brook | 10 | 1736 | -4246.41 |
| CL9_551693434PSV_F0640996CCAM04736P1 | Blunts_Point | 1 | 1737 | -4246.41 |
| CL9_551693502PSV_F0640996CCAM04736P1 | Blunts_Point | 2 | 1737 | -4246.41 |
| CL9_551871632PSV_F0641194CCAM01739P1 | Hamilton_Pond | 1 | 1739 | -4245.12 |
| CL9_551871700PSV_F0641194CCAM01739P1 | Hamilton_Pond | 2 | 1739 | -4245.12 |
| CL9_551871764PSV_F0641194CCAM01739P1 | Hamilton_Pond | 3 | 1739 | -4245.12 |
| CL9_551871828PSV_F0641194CCAM01739P1 | Hamilton_Pond | 4 | 1739 | -4245.12 |
| CL9_551871892PSV_F0641194CCAM01739P1 | Hamilton_Pond | 5 | 1739 | -4245.12 |
| CL9_551871956PSV_F0641194CCAM01739P1 | Hamilton_Pond | 6 | 1739 | -4245.12 |
| CL9_551872148PSV_F0641194CCAM01739P1 | Hamilton_Pond | 9 | 1739 | -4245.12 |
| CL9_551872354PSV_F0641194CCAM01739P1 | Hamilton_Pond | 10 | 1739 | -4245.12 |
| CL9_551873770PSV_F0641194CCAM03739P1 | Rice_Point | 1 | 1739 | -4245.12 |
| CL9_551873845PSV_F0641194CCAM03739P1 | Rice_Point | 2 | 1739 | -4245.12 |
| CL9_551873910PSV_F0641194CCAM03739P1 | Rice_Point | 3 | 1739 | -4245.12 |
| CL9_551873975PSV_F0641194CCAM03739P1 | Rice_Point | 4 | 1739 | -4245.12 |
| CL9_551874040PSV_F0641194CCAM03739P1 | Rice_Point | 5 | 1739 | -4245.12 |
| CL9_551874105PSV_F0641194CCAM03739P1 | Rice_Point | 6 | 1739 | -4245.12 |
| CL9_551874171PSV_F0641194CCAM03739P1 | Rice_Point | 7 | 1739 | -4245.12 |
| CL9_551874236PSV_F0641194CCAM03739P1 | Rice_Point | 8 | 1739 | -4245.12 |

| | | | | |
|---|---|---|---|---|
| CL9_551874301PSV_F0641194CCAM03739P1 | Rice_Point | 9 | 1739 | -4245.12 |
| CL9_551874500PSV_F0641194CCAM03739P1 | Rice_Point | 10 | 1739 | -4245.12 |
| CL9_552400284PSV_F0641626CCAM01744P1 | Mingo_ccam | 1 | 1745 | -4243.45 |
| CL9_552400352PSV_F0641626CCAM01744P1 | Mingo_ccam | 2 | 1745 | -4243.45 |
| CL9_552400416PSV_F0641626CCAM01744P1 | Mingo_ccam | 3 | 1745 | -4243.45 |
| CL9_552400480PSV_F0641626CCAM01744P1 | Mingo_ccam | 4 | 1745 | -4243.45 |
| CL9_552400544PSV_F0641626CCAM01744P1 | Mingo_ccam | 5 | 1745 | -4243.45 |
| CL9_552400608PSV_F0641626CCAM01744P1 | Mingo_ccam | 6 | 1745 | -4243.45 |
| CL9_552400672PSV_F0641626CCAM01744P1 | Mingo_ccam | 7 | 1745 | -4243.45 |
| CL9_552400735PSV_F0641626CCAM01744P1 | Mingo_ccam | 8 | 1745 | -4243.45 |
| CL9_552400799PSV_F0641626CCAM01744P1 | Mingo_ccam | 9 | 1745 | -4243.45 |
| CL9_552400960PSV_F0641626CCAM01744P1 | Mingo_ccam | 10 | 1745 | -4243.45 |
| CL9_552401269PSV_F0641626CCAM02744P1 | Jobbies_ccam | 1 | 1745 | -4243.45 |
| CL9_552401344PSV_F0641626CCAM02744P1 | Jobbies_ccam | 2 | 1745 | -4243.45 |
| CL9_552401410PSV_F0641626CCAM02744P1 | Jobbies_ccam | 3 | 1745 | -4243.45 |
| CL9_552401647PSV_F0641626CCAM02744P1 | Jobbies_ccam | 4 | 1745 | -4243.45 |
| CL9_552401722PSV_F0641626CCAM02744P1 | Jobbies_ccam | 5 | 1745 | -4243.45 |
| CL9_552401788PSV_F0641626CCAM02744P1 | Jobbies_ccam | 6 | 1745 | -4243.45 |
| CL9_552402025PSV_F0641626CCAM02744P1 | Jobbies_ccam | 7 | 1745 | -4243.45 |
| CL9_552402101PSV_F0641626CCAM02744P1 | Jobbies_ccam | 8 | 1745 | -4243.45 |
| CL9_552402167PSV_F0641626CCAM02744P1 | Jobbies_ccam | 9 | 1745 | -4243.45 |
| CL9_552402330PSV_F0641626CCAM02744P1 | Jobbies_ccam | 10 | 1745 | -4243.45 |
| CL9_552487428PSV_F0641626CCAM05744P1 | Blubber | 1 | 1746 | -4243.45 |
| CL9_552487496PSV_F0641626CCAM05744P1 | Blubber | 2 | 1746 | -4243.45 |
| CL9_552487560PSV_F0641626CCAM05744P1 | Blubber | 3 | 1746 | -4243.45 |
| CL9_552487624PSV_F0641626CCAM05744P1 | Blubber | 4 | 1746 | -4243.45 |
| CL9_552487688PSV_F0641626CCAM05744P1 | Blubber | 5 | 1746 | -4243.45 |
| CL9_552487814PSV_F0641626CCAM05744P1 | Blubber | 7 | 1746 | -4243.45 |
| CL9_552487878PSV_F0641626CCAM05744P1 | Blubber | 8 | 1746 | -4243.45 |
| CL9_552487942PSV_F0641626CCAM05744P1 | Blubber | 9 | 1746 | -4243.45 |
| CL9_552488070PSV_F0641626CCAM05744P1 | Blubber | 11 | 1746 | -4243.45 |
| CL9_552488134PSV_F0641626CCAM05744P1 | Blubber | 12 | 1746 | -4243.45 |
| CL9_552488198PSV_F0641626CCAM05744P1 | Blubber | 13 | 1746 | -4243.45 |
| CL9_552582819PSV_F0641890CCAM02747P1 | Whortleberry | 1 | 1747 | -4240.71 |
| CL9_552582894PSV_F0641890CCAM02747P1 | Whortleberry | 2 | 1747 | -4240.71 |
| CL9_552583569PSV_F0641890CCAM03747P1 | Sugar_Plum | 1 | 1747 | -4240.71 |
| CL9_552583644PSV_F0641890CCAM03747P1 | Sugar_Plum | 2 | 1747 | -4240.71 |
| CL9_552583710PSV_F0641890CCAM03747P1 | Sugar_Plum | 3 | 1747 | -4240.71 |
| CL9_552583776PSV_F0641890CCAM03747P1 | Sugar_Plum | 4 | 1747 | -4240.71 |
| CL9_552583972PSV_F0641890CCAM03747P1 | Sugar_Plum | 5 | 1747 | -4240.71 |
| CL9_552672422PSV_F0641980CCAM02748P1 | Sugar_Loaves | 3 | 1748 | -4239.34 |
| CL9_552672488PSV_F0641980CCAM02748P1 | Sugar_Loaves | 4 | 1748 | -4239.34 |
| CL9_552672553PSV_F0641980CCAM02748P1 | Sugar_Loaves | 5 | 1748 | -4239.34 |
| CL9_552672618PSV_F0641980CCAM02748P1 | Sugar_Loaves | 6 | 1748 | -4239.34 |
| CL9_552672683PSV_F0641980CCAM02748P1 | Sugar_Loaves | 7 | 1748 | -4239.34 |
| CL9_552672748PSV_F0641980CCAM02748P1 | Sugar_Loaves | 8 | 1748 | -4239.34 |
| CL9_552672814PSV_F0641980CCAM02748P1 | Sugar_Loaves | 9 | 1748 | -4239.34 |
| CL9_552673010PSV_F0641980CCAM02748P1 | Sugar_Loaves | 10 | 1748 | -4239.34 |
| CL9_553019658PSV_F0642154CCAM01752P1 | Grogg_Ledge | 1 | 1752 | -4238.5 |
| CL9_553019727PSV_F0642154CCAM01752P1 | Grogg_Ledge | 2 | 1752 | -4238.5 |
| CL9_553019962PSV_F0642154CCAM01752P1 | Grogg_Ledge | 3 | 1752 | -4238.5 |
| CL9_553020031PSV_F0642154CCAM01752P1 | Grogg_Ledge | 4 | 1752 | -4238.5 |
| CL9_553020225PSV_F0642154CCAM01752P1 | Grogg_Ledge | 5 | 1752 | -4238.5 |
| CL9_553115045PSV_F0642238CCAM02753P1 | Foxbird_ccam | 1 | 1753 | -4237.64 |
| CL9_553115113PSV_F0642238CCAM02753P1 | Foxbird_ccam | 2 | 1753 | -4237.64 |
| CL9_553115177PSV_F0642238CCAM02753P1 | Foxbird_ccam | 3 | 1753 | -4237.64 |
| CL9_553115241PSV_F0642238CCAM02753P1 | Foxbird_ccam | 4 | 1753 | -4237.64 |
| CL9_553115435PSV_F0642238CCAM02753P1 | Foxbird_ccam | 5 | 1753 | -4237.64 |
| CL9_553197509PSV_F0642442CCAM01754P1 | Hells_Half_Acre | 1 | 1754 | -4236.2 |

| | | | | |
|---|---|---|---|---|
| CL9_553197585PSV_F0642442CCAM01754P1 | Hells_Half_Acre | 2 | 1754 | -4236.2 |
| CL9_553198982PSV_F0642442CCAM02754P1 | Hockomock_Bay | 1 | 1754 | -4236.2 |
| CL9_553199050PSV_F0642442CCAM02754P1 | Hockomock_Bay | 2 | 1754 | -4236.2 |
| CL9_553199114PSV_F0642442CCAM02754P1 | Hockomock_Bay | 3 | 1754 | -4236.2 |
| CL9_553199178PSV_F0642442CCAM02754P1 | Hockomock_Bay | 4 | 1754 | -4236.2 |
| CL9_553286386PSV_F0642790CCAM01755P1 | Jimmies_Ledge | 1 | 1755 | -4232.98 |
| CL9_553286461PSV_F0642790CCAM01755P1 | Jimmies_Ledge | 2 | 1755 | -4232.98 |
| CL9_553286526PSV_F0642790CCAM01755P1 | Jimmies_Ledge | 3 | 1755 | -4232.98 |
| CL9_553286592PSV_F0642790CCAM01755P1 | Jimmies_Ledge | 4 | 1755 | -4232.98 |
| CL9_553286829PSV_F0642790CCAM01755P1 | Jimmies_Ledge | 5 | 1755 | -4232.98 |
| CL9_553286904PSV_F0642790CCAM01755P1 | Jimmies_Ledge | 6 | 1755 | -4232.98 |
| CL9_553286970PSV_F0642790CCAM01755P1 | Jimmies_Ledge | 7 | 1755 | -4232.98 |
| CL9_553287036PSV_F0642790CCAM01755P1 | Jimmies_Ledge | 8 | 1755 | -4232.98 |
| CL9_553287102PSV_F0642790CCAM01755P1 | Jimmies_Ledge | 9 | 1755 | -4232.98 |
| CL9_553287298PSV_F0642790CCAM01755P1 | Jimmies_Ledge | 10 | 1755 | -4232.98 |
| CL9_553287631PSV_F0642790CCAM02755P1 | Jennys_Nubble | 1 | 1755 | -4232.98 |
| CL9_553287699PSV_F0642790CCAM02755P1 | Jennys_Nubble | 2 | 1755 | -4232.98 |
| CL9_553287763PSV_F0642790CCAM02755P1 | Jennys_Nubble | 3 | 1755 | -4232.98 |
| CL9_553287826PSV_F0642790CCAM02755P1 | Jennys_Nubble | 4 | 1755 | -4232.98 |
| CL9_553287890PSV_F0642790CCAM02755P1 | Jennys_Nubble | 5 | 1755 | -4232.98 |
| CL9_553287954PSV_F0642790CCAM02755P1 | Jennys_Nubble | 6 | 1755 | -4232.98 |
| CL9_553288018PSV_F0642790CCAM02755P1 | Jennys_Nubble | 7 | 1755 | -4232.98 |
| CL9_553288082PSV_F0642790CCAM02755P1 | Jennys_Nubble | 8 | 1755 | -4232.98 |
| CL9_553288146PSV_F0642790CCAM02755P1 | Jennys_Nubble | 9 | 1755 | -4232.98 |
| CL9_553288340PSV_F0642790CCAM02755P1 | Jennys_Nubble | 10 | 1755 | -4232.98 |
| CL9_555507578PSV_F0642790CCAM01780P1 | Huckins_Ledge | 1 | 1780 | -4232.98 |
| CL9_555507647PSV_F0642790CCAM01780P1 | Huckins_Ledge | 2 | 1780 | -4232.98 |
| CL9_555507711PSV_F0642790CCAM01780P1 | Huckins_Ledge | 3 | 1780 | -4232.98 |
| CL9_555507943PSV_F0642790CCAM01780P1 | Huckins_Ledge | 4 | 1780 | -4232.98 |
| CL9_555508008PSV_F0642790CCAM01780P1 | Huckins_Ledge | 5 | 1780 | -4232.98 |
| CL9_555508072PSV_F0642790CCAM01780P1 | Huckins_Ledge | 6 | 1780 | -4232.98 |
| CL9_555508307PSV_F0642790CCAM01780P1 | Huckins_Ledge | 7 | 1780 | -4232.98 |
| CL9_555508372PSV_F0642790CCAM01780P1 | Huckins_Ledge | 8 | 1780 | -4232.98 |
| CL9_555508566PSV_F0642790CCAM01780P1 | Huckins_Ledge | 9 | 1780 | -4232.98 |
| CL9_555508879PSV_F0642790CCAM02780P1 | Mackerel_Ledge | 1 | 1780 | -4232.98 |
| CL9_555508947PSV_F0642790CCAM02780P1 | Mackerel_Ledge | 2 | 1780 | -4232.98 |
| CL9_555509011PSV_F0642790CCAM02780P1 | Mackerel_Ledge | 3 | 1780 | -4232.98 |
| CL9_555509243PSV_F0642790CCAM02780P1 | Mackerel_Ledge | 4 | 1780 | -4232.98 |
| CL9_555509308PSV_F0642790CCAM02780P1 | Mackerel_Ledge | 5 | 1780 | -4232.98 |
| CL9_555509372PSV_F0642790CCAM02780P1 | Mackerel_Ledge | 6 | 1780 | -4232.98 |
| CL9_555688483PSV_F0650000CCAM01782P1 | Thorne | 1 | 1782 | -4227.25 |
| CL9_555688552PSV_F0650000CCAM01782P1 | Thorne | 2 | 1782 | -4227.25 |
| CL9_555688616PSV_F0650000CCAM01782P1 | Thorne | 3 | 1782 | -4227.25 |
| CL9_555688680PSV_F0650000CCAM01782P1 | Thorne | 4 | 1782 | -4227.25 |
| CL9_555688874PSV_F0650000CCAM01782P1 | Thorne | 5 | 1782 | -4227.25 |
| CL9_556130506PSV_F0650550CCAM01787P1 | Deadman_Ledge | 1 | 1787 | -4227.85 |
| CL9_556130574PSV_F0650550CCAM01787P1 | Deadman_Ledge | 2 | 1787 | -4227.85 |
| CL9_556130637PSV_F0650550CCAM01787P1 | Deadman_Ledge | 3 | 1787 | -4227.85 |
| CL9_556130739PSV_F0650550CCAM01787P1 | Deadman_Ledge | 4 | 1787 | -4227.85 |
| CL9_556130802PSV_F0650550CCAM01787P1 | Deadman_Ledge | 5 | 1787 | -4227.85 |
| CL9_556130865PSV_F0650550CCAM01787P1 | Deadman_Ledge | 6 | 1787 | -4227.85 |
| CL9_556130962PSV_F0650550CCAM01787P1 | Deadman_Ledge | 7 | 1787 | -4227.85 |
| CL9_556131025PSV_F0650550CCAM01787P1 | Deadman_Ledge | 8 | 1787 | -4227.85 |
| CL9_556131219PSV_F0650550CCAM01787P1 | Deadman_Ledge | 9 | 1787 | -4227.85 |
| CL9_556220348PSV_F0650646CCAM01788P1 | Dumplings_Island_ccam | 1 | 1788 | -4228.92 |
| CL9_556220417PSV_F0650646CCAM01788P1 | Dumplings_Island_ccam | 2 | 1788 | -4228.92 |
| CL9_556220481PSV_F0650646CCAM01788P1 | Dumplings_Island_ccam | 3 | 1788 | -4228.92 |
| CL9_556220545PSV_F0650646CCAM01788P1 | Dumplings_Island_ccam | 4 | 1788 | -4228.92 |
| CL9_556220706PSV_F0650646CCAM01788P1 | Dumplings_Island_ccam | 5 | 1788 | -4228.92 |

| | | | | |
|---|---|---|---|---|
| CL9_556305669PSV_F0650916CCAM02789P1 | Manana | 1 | 1789 | -4227.54 |
| CL9_556305744PSV_F0650916CCAM02789P1 | Manana | 2 | 1789 | -4227.54 |
| CL9_556305810PSV_F0650916CCAM02789P1 | Manana | 3 | 1789 | -4227.54 |
| CL9_556305876PSV_F0650916CCAM02789P1 | Manana | 4 | 1789 | -4227.54 |
| CL9_556306072PSV_F0650916CCAM02789P1 | Manana | 5 | 1789 | -4227.54 |
| CL9_556392984PSV_F0651174CCAM01790P1 | Zephyr_Ledges | 2 | 1790 | -4225.13 |
| CL9_556393048PSV_F0651174CCAM01790P1 | Zephyr_Ledges | 3 | 1790 | -4225.13 |
| CL9_556393112PSV_F0651174CCAM01790P1 | Zephyr_Ledges | 4 | 1790 | -4225.13 |
| CL9_556393346PSV_F0651174CCAM01790P1 | Zephyr_Ledges | 5 | 1790 | -4225.13 |
| CL9_556393414PSV_F0651174CCAM01790P1 | Zephyr_Ledges | 6 | 1790 | -4225.13 |
| CL9_556393606PSV_F0651174CCAM01790P1 | Zephyr_Ledges | 9 | 1790 | -4225.13 |
| CL9_556394123PSV_F0651174CCAM02790P1 | Wallace_Ledge | 1 | 1790 | -4225.13 |
| CL9_556394198PSV_F0651174CCAM02790P1 | Wallace_Ledge | 2 | 1790 | -4225.13 |
| CL9_556394263PSV_F0651174CCAM02790P1 | Wallace_Ledge | 3 | 1790 | -4225.13 |
| CL9_556394328PSV_F0651174CCAM02790P1 | Wallace_Ledge | 4 | 1790 | -4225.13 |
| CL9_556394564PSV_F0651174CCAM02790P1 | Wallace_Ledge | 5 | 1790 | -4225.13 |
| CL9_556394639PSV_F0651174CCAM02790P1 | Wallace_Ledge | 6 | 1790 | -4225.13 |
| CL9_556394704PSV_F0651174CCAM02790P1 | Wallace_Ledge | 7 | 1790 | -4225.13 |
| CL9_556394769PSV_F0651174CCAM02790P1 | Wallace_Ledge | 8 | 1790 | -4225.13 |
| CL9_556394835PSV_F0651174CCAM02790P1 | Wallace_Ledge | 9 | 1790 | -4225.13 |
| CL9_556747954PSV_F0651438CCAM01794P1 | Boiler | 1 | 1794 | -4224.47 |
| CL9_556748029PSV_F0651438CCAM01794P1 | Boiler | 2 | 1794 | -4224.47 |
| CL9_556748094PSV_F0651438CCAM01794P1 | Boiler | 3 | 1794 | -4224.47 |
| CL9_556748161PSV_F0651438CCAM01794P1 | Boiler | 4 | 1794 | -4224.47 |
| CL9_556748396PSV_F0651438CCAM01794P1 | Boiler | 5 | 1794 | -4224.47 |
| CL9_556748471PSV_F0651438CCAM01794P1 | Boiler | 6 | 1794 | -4224.47 |
| CL9_556748536PSV_F0651438CCAM01794P1 | Boiler | 7 | 1794 | -4224.47 |
| CL9_556748602PSV_F0651438CCAM01794P1 | Boiler | 8 | 1794 | -4224.47 |
| CL9_556748667PSV_F0651438CCAM01794P1 | Boiler | 9 | 1794 | -4224.47 |
| CL9_556748863PSV_F0651438CCAM01794P1 | Boiler | 10 | 1794 | -4224.47 |
| CL9_556749232PSV_F0651438CCAM02794P1 | Biljim | 1 | 1794 | -4224.47 |
| CL9_556749307PSV_F0651438CCAM02794P1 | Biljim | 2 | 1794 | -4224.47 |
| CL9_556749373PSV_F0651438CCAM02794P1 | Biljim | 3 | 1794 | -4224.47 |
| CL9_556749616PSV_F0651438CCAM02794P1 | Biljim | 4 | 1794 | -4224.47 |
| CL9_556749691PSV_F0651438CCAM02794P1 | Biljim | 5 | 1794 | -4224.47 |
| CL9_556749988PSV_F0651438CCAM02794P1 | Biljim | 7 | 1794 | -4224.47 |
| CL9_556750055PSV_F0651438CCAM02794P1 | Biljim | 8 | 1794 | -4224.47 |
| CL9_556842413PSV_F0651642CCAM01795P1 | Agamenticus_CCAM | 1 | 1795 | -4222.52 |
| CL9_556842488PSV_F0651642CCAM01795P1 | Agamenticus_CCAM | 2 | 1795 | -4222.52 |
| CL9_556842553PSV_F0651642CCAM01795P1 | Agamenticus_CCAM | 3 | 1795 | -4222.52 |
| CL9_556842790PSV_F0651642CCAM01795P1 | Agamenticus_CCAM | 4 | 1795 | -4222.52 |
| CL9_556842865PSV_F0651642CCAM01795P1 | Agamenticus_CCAM | 5 | 1795 | -4222.52 |
| CL9_556842932PSV_F0651642CCAM01795P1 | Agamenticus_CCAM | 6 | 1795 | -4222.52 |
| CL9_556843168PSV_F0651642CCAM01795P1 | Agamenticus_CCAM | 7 | 1795 | -4222.52 |
| CL9_556843244PSV_F0651642CCAM01795P1 | Agamenticus_CCAM | 8 | 1795 | -4222.52 |
| CL9_556843309PSV_F0651642CCAM01795P1 | Agamenticus_CCAM | 9 | 1795 | -4222.52 |
| CL9_556843505PSV_F0651642CCAM01795P1 | Agamenticus_CCAM | 10 | 1795 | -4222.52 |
| CL9_556931267PSV_F0651934CCAM01796P1 | Mosquick | 2 | 1796 | -4220.07 |
| CL9_556931332PSV_F0651934CCAM01796P1 | Mosquick | 3 | 1796 | -4220.07 |
| CL9_556931562PSV_F0651934CCAM01796P1 | Mosquick | 5 | 1796 | -4220.07 |
| CL9_556931875PSV_F0651934CCAM02796P1 | Tinker | 1 | 1796 | -4220.07 |
| CL9_556931950PSV_F0651934CCAM02796P1 | Tinker | 2 | 1796 | -4220.07 |
| CL9_556932015PSV_F0651934CCAM02796P1 | Tinker | 3 | 1796 | -4220.07 |
| CL9_556932081PSV_F0651934CCAM02796P1 | Tinker | 4 | 1796 | -4220.07 |
| CL9_556932276PSV_F0651934CCAM02796P1 | Tinker | 5 | 1796 | -4220.07 |
| CL9_557029274PSV_F0652186CCAM05797P1 | Shag_Rock | 1 | 1797 | -4217.47 |
| CL9_557029343PSV_F0652186CCAM05797P1 | Shag_Rock | 2 | 1797 | -4217.47 |
| CL9_557029407PSV_F0652186CCAM05797P1 | Shag_Rock | 3 | 1797 | -4217.47 |
| CL9_557029471PSV_F0652186CCAM05797P1 | Shag_Rock | 4 | 1797 | -4217.47 |

| | | | | |
|---|---|---|---|---|
| CL9_557029677PSV_F0652186CCAM05797P1 | Shag_Rock | 5 | 1797 | -4217.47 |
| CL9_557030050PSV_F0652186CCAM06797P1 | Abagadasset | 1 | 1797 | -4217.47 |
| CL9_557030119PSV_F0652186CCAM06797P1 | Abagadasset | 2 | 1797 | -4217.47 |
| CL9_557030183PSV_F0652186CCAM06797P1 | Abagadasset | 3 | 1797 | -4217.47 |
| CL9_557030426PSV_F0652186CCAM06797P1 | Abagadasset | 4 | 1797 | -4217.47 |
| CL9_557030495PSV_F0652186CCAM06797P1 | Abagadasset | 5 | 1797 | -4217.47 |
| CL9_557030559PSV_F0652186CCAM06797P1 | Abagadasset | 6 | 1797 | -4217.47 |
| CL9_557030802PSV_F0652186CCAM06797P1 | Abagadasset | 7 | 1797 | -4217.47 |
| CL9_557030870PSV_F0652186CCAM06797P1 | Abagadasset | 8 | 1797 | -4217.47 |
| CL9_557287580PSV_F0652456CCAM01800P1 | Bucklin_Rock | 1 | 1800 | -4214.21 |
| CL9_557288056PSV_F0652456CCAM01800P1 | Bucklin_Rock | 5 | 1800 | -4214.21 |
| CL9_557288122PSV_F0652456CCAM01800P1 | Bucklin_Rock | 6 | 1800 | -4214.21 |
| CL9_557288380PSV_F0652456CCAM01800P1 | Bucklin_Rock | 7 | 1800 | -4214.21 |
| CL9_557288456PSV_F0652456CCAM01800P1 | Bucklin_Rock | 8 | 1800 | -4214.21 |
| CL9_557288522PSV_F0652456CCAM01800P1 | Bucklin_Rock | 9 | 1800 | -4214.21 |
| CL9_557289058PSV_F0652456CCAM02800P1 | Gilkey_Harbor | 1 | 1800 | -4214.21 |
| CL9_557289133PSV_F0652456CCAM02800P1 | Gilkey_Harbor | 2 | 1800 | -4214.21 |
| CL9_557289198PSV_F0652456CCAM02800P1 | Gilkey_Harbor | 3 | 1800 | -4214.21 |
| CL9_557289263PSV_F0652456CCAM02800P1 | Gilkey_Harbor | 4 | 1800 | -4214.21 |
| CL9_557289459PSV_F0652456CCAM02800P1 | Gilkey_Harbor | 5 | 1800 | -4214.21 |
| CL9_557463724PSV_F0652720CCAM01802P1 | Tyler_ccam | 1 | 1802 | -4209.11 |
| CL9_557463801PSV_F0652720CCAM01802P1 | Tyler_ccam | 2 | 1802 | -4209.11 |
| CL9_557463866PSV_F0652720CCAM01802P1 | Tyler_ccam | 3 | 1802 | -4209.11 |
| CL9_557463932PSV_F0652720CCAM01802P1 | Tyler_ccam | 4 | 1802 | -4209.11 |
| CL9_557464128PSV_F0652720CCAM01802P1 | Tyler_ccam | 5 | 1802 | -4209.11 |
| CL9_557728387PSV_F0652882CCAM01805P1 | Phoebe_Ledge | 3 | 1805 | -4206.88 |
| CL9_557728453PSV_F0652882CCAM01805P1 | Phoebe_Ledge | 4 | 1805 | -4206.88 |
| CL9_557728663PSV_F0652882CCAM01805P1 | Phoebe_Ledge | 5 | 1805 | -4206.88 |
| CL9_557817431PSV_F0652882CCAM04805P1 | Robinson_Rock_ccam | 1 | 1806 | -4206.88 |
| CL9_557817699PSV_F0652882CCAM04805P1 | Robinson_Rock_ccam | 2 | 1806 | -4206.88 |
| CL9_557818026PSV_F0652882CCAM04805P1 | Robinson_Rock_ccam | 3 | 1806 | -4206.88 |
| CL9_557818355PSV_F0652882CCAM04805P1 | Robinson_Rock_ccam | 4 | 1806 | -4206.88 |
| CL9_558085399PSV_F0653200CCAM01809P1 | Chamberly_CCAM | 1 | 1809 | -4202.55 |
| CL9_558085474PSV_F0653200CCAM01809P1 | Chamberly_CCAM | 2 | 1809 | -4202.55 |
| CL9_558085540PSV_F0653200CCAM01809P1 | Chamberly_CCAM | 3 | 1809 | -4202.55 |
| CL9_558085606PSV_F0653200CCAM01809P1 | Chamberly_CCAM | 4 | 1809 | -4202.55 |
| CL9_558085842PSV_F0653200CCAM01809P1 | Chamberly_CCAM | 5 | 1809 | -4202.55 |
| CL9_558085917PSV_F0653200CCAM01809P1 | Chamberly_CCAM | 6 | 1809 | -4202.55 |
| CL9_558085983PSV_F0653200CCAM01809P1 | Chamberly_CCAM | 7 | 1809 | -4202.55 |
| CL9_558086049PSV_F0653200CCAM01809P1 | Chamberly_CCAM | 8 | 1809 | -4202.55 |
| CL9_558086115PSV_F0653200CCAM01809P1 | Chamberly_CCAM | 9 | 1809 | -4202.55 |
| CL9_558086311PSV_F0653200CCAM01809P1 | Chamberly_CCAM | 10 | 1809 | -4202.55 |
| CL9_558086627PSV_F0653200CCAM02809P1 | Locust_Island_Ledge | 1 | 1809 | -4202.55 |
| CL9_558086702PSV_F0653200CCAM02809P1 | Locust_Island_Ledge | 2 | 1809 | -4202.55 |
| CL9_558086768PSV_F0653200CCAM02809P1 | Locust_Island_Ledge | 3 | 1809 | -4202.55 |
| CL9_558086834PSV_F0653200CCAM02809P1 | Locust_Island_Ledge | 4 | 1809 | -4202.55 |
| CL9_558087030PSV_F0653200CCAM02809P1 | Locust_Island_Ledge | 5 | 1809 | -4202.55 |
| CL9_558087353PSV_F0653200CCAM03809P1 | Thread_of_Life_Ledge | 1 | 1809 | -4202.55 |
| CL9_558087428PSV_F0653200CCAM03809P1 | Thread_of_Life_Ledge | 2 | 1809 | -4202.55 |
| CL9_558087494PSV_F0653200CCAM03809P1 | Thread_of_Life_Ledge | 3 | 1809 | -4202.55 |
| CL9_558087560PSV_F0653200CCAM03809P1 | Thread_of_Life_Ledge | 4 | 1809 | -4202.55 |
| CL9_558087756PSV_F0653200CCAM03809P1 | Thread_of_Life_Ledge | 5 | 1809 | -4202.55 |
| CL9_558348410PSV_F0653308CCAM01811P1 | Kemps_Folly_ccam | 1 | 1810 | -4200.1 |
| CL9_558348485PSV_F0653308CCAM01811P1 | Kemps_Folly_ccam | 2 | 1810 | -4200.1 |
| CL9_558348551PSV_F0653308CCAM01811P1 | Kemps_Folly_ccam | 3 | 1810 | -4200.1 |
| CL9_558348617PSV_F0653308CCAM01811P1 | Kemps_Folly_ccam | 4 | 1810 | -4200.1 |
| CL9_558348813PSV_F0653308CCAM01811P1 | Kemps_Folly_ccam | 5 | 1810 | -4200.1 |
| CL9_558349124PSV_F0653308CCAM02811P1 | Mount_Emphraim | 1 | 1810 | -4200.1 |
| CL9_558349266PSV_F0653308CCAM02811P1 | Mount_Emphraim | 3 | 1810 | -4200.1 |

| | | | | |
|---|---|---|---|---|
| CL9_558349504PSV_F0653308CCAM02811P1 | Mount_Emphraim | 4 | 1810 | -4200.1 |
| CL9_558349580PSV_F0653308CCAM02811P1 | Mount_Emphraim | 5 | 1810 | -4200.1 |
| CL9_558349646PSV_F0653308CCAM02811P1 | Mount_Emphraim | 6 | 1810 | -4200.1 |
| CL9_558349849PSV_F0653308CCAM02811P1 | Mount_Emphraim | 7 | 1810 | -4200.1 |
| CL9_558349988PSV_F0653308CCAM02811P1 | Mount_Emphraim | 9 | 1810 | -4200.1 |
| CL9_558350153PSV_F0653308CCAM02811P1 | Mount_Emphraim | 10 | 1810 | -4200.1 |
| CL9_558350465PSV_F0653308CCAM03811P1 | Sasanoa_ccam | 1 | 1810 | -4200.1 |
| CL9_558350541PSV_F0653308CCAM03811P1 | Sasanoa_ccam | 2 | 1810 | -4200.1 |
| CL9_558350607PSV_F0653308CCAM03811P1 | Sasanoa_ccam | 3 | 1810 | -4200.1 |
| CL9_558350673PSV_F0653308CCAM03811P1 | Sasanoa_ccam | 4 | 1810 | -4200.1 |
| CL9_558350870PSV_F0653308CCAM03811P1 | Sasanoa_ccam | 5 | 1810 | -4200.1 |
| CL9_558351222PSV_F0653308CCAM04811P1 | Mount_Coe | 1 | 1810 | -4200.1 |
| CL9_558351298PSV_F0653308CCAM04811P1 | Mount_Coe | 2 | 1810 | -4200.1 |
| CL9_558351364PSV_F0653308CCAM04811P1 | Mount_Coe | 3 | 1810 | -4200.1 |
| CL9_558351629PSV_F0653308CCAM04811P1 | Mount_Coe | 4 | 1810 | -4200.1 |
| CL9_558351705PSV_F0653308CCAM04811P1 | Mount_Coe | 5 | 1810 | -4200.1 |
| CL9_558351771PSV_F0653308CCAM04811P1 | Mount_Coe | 6 | 1810 | -4200.1 |
| CL9_558352036PSV_F0653308CCAM04811P1 | Mount_Coe | 7 | 1810 | -4200.1 |
| CL9_558352112PSV_F0653308CCAM04811P1 | Mount_Coe | 8 | 1810 | -4200.1 |
| CL9_558352178PSV_F0653308CCAM04811P1 | Mount_Coe | 9 | 1810 | -4200.1 |
| CL9_558352370PSV_F0653308CCAM04811P1 | Mount_Coe | 10 | 1810 | -4200.1 |
| CL9_558529949PSV_F0660000CCAM01814P1 | Pumpkin_Nob_ccam | 1 | 1814 | -4198.86 |
| CL9_558530025PSV_F0660000CCAM01814P1 | Pumpkin_Nob_ccam | 2 | 1814 | -4198.86 |
| CL9_558530091PSV_F0660000CCAM01814P1 | Pumpkin_Nob_ccam | 3 | 1814 | -4198.86 |
| CL9_558530157PSV_F0660000CCAM01814P1 | Pumpkin_Nob_ccam | 4 | 1814 | -4198.86 |
| CL9_558530352PSV_F0660000CCAM01814P1 | Pumpkin_Nob_ccam | 5 | 1814 | -4198.86 |
| CL9_558707030PSV_F0660084CCAM02816P1 | Bragdon | 1 | 1816 | -4196.55 |
| CL9_558707095PSV_F0660084CCAM02816P1 | Bragdon | 2 | 1816 | -4196.55 |
| CL9_558707521PSV_F0660084CCAM02816P1 | Bragdon | 6 | 1816 | -4196.55 |
| CL9_558707756PSV_F0660084CCAM02816P1 | Bragdon | 7 | 1816 | -4196.55 |
| CL9_558708328PSV_F0660084CCAM03816P1 | Graffam | 1 | 1816 | -4196.55 |
| CL9_558708396PSV_F0660084CCAM03816P1 | Graffam | 2 | 1816 | -4196.55 |
| CL9_558708460PSV_F0660084CCAM03816P1 | Graffam | 3 | 1816 | -4196.55 |
| CL9_558708693PSV_F0660084CCAM03816P1 | Graffam | 4 | 1816 | -4196.55 |
| CL9_558708761PSV_F0660084CCAM03816P1 | Graffam | 5 | 1816 | -4196.55 |
| CL9_558708825PSV_F0660084CCAM03816P1 | Graffam | 6 | 1816 | -4196.55 |
| CL9_558709059PSV_F0660084CCAM03816P1 | Graffam | 7 | 1816 | -4196.55 |
| CL9_558709127PSV_F0660084CCAM03816P1 | Graffam | 8 | 1816 | -4196.55 |
| CL9_558709191PSV_F0660084CCAM03816P1 | Graffam | 9 | 1816 | -4196.55 |
| CL9_558709394PSV_F0660084CCAM03816P1 | Graffam | 10 | 1816 | -4196.55 |
| CL9_558891448PSV_F0660084CCAM01818P1 | Medomak | 1 | 1818 | -4196.55 |
| CL9_558891516PSV_F0660084CCAM01818P1 | Medomak | 2 | 1818 | -4196.55 |
| CL9_558891580PSV_F0660084CCAM01818P1 | Medomak | 3 | 1818 | -4196.55 |
| CL9_558891814PSV_F0660084CCAM01818P1 | Medomak | 4 | 1818 | -4196.55 |
| CL9_558891883PSV_F0660084CCAM01818P1 | Medomak | 5 | 1818 | -4196.55 |
| CL9_558891947PSV_F0660084CCAM01818P1 | Medomak | 6 | 1818 | -4196.55 |
| CL9_558892181PSV_F0660084CCAM01818P1 | Medomak | 7 | 1818 | -4196.55 |
| CL9_558892250PSV_F0660084CCAM01818P1 | Medomak | 8 | 1818 | -4196.55 |
| CL9_558892314PSV_F0660084CCAM01818P1 | Medomak | 9 | 1818 | -4196.55 |
| CL9_558892508PSV_F0660084CCAM01818P1 | Medomak | 10 | 1818 | -4196.55 |
| CL9_559146324PSV_F0660246CCAM01820P1 | Passadumkeag_ccam_2 | 1 | 1821 | -4195.37 |
| CL9_559146392PSV_F0660246CCAM01820P1 | Passadumkeag_ccam_2 | 2 | 1821 | -4195.37 |
| CL9_559146455PSV_F0660246CCAM01820P1 | Passadumkeag_ccam_2 | 3 | 1821 | -4195.37 |
| CL9_559146519PSV_F0660246CCAM01820P1 | Passadumkeag_ccam_2 | 4 | 1821 | -4195.37 |
| CL9_559146583PSV_F0660246CCAM01820P1 | Passadumkeag_ccam_2 | 5 | 1821 | -4195.37 |
| CL9_559146646PSV_F0660246CCAM01820P1 | Passadumkeag_ccam_2 | 6 | 1821 | -4195.37 |
| CL9_559146710PSV_F0660246CCAM01820P1 | Passadumkeag_ccam_2 | 7 | 1821 | -4195.37 |
| CL9_559146773PSV_F0660246CCAM01820P1 | Passadumkeag_ccam_2 | 8 | 1821 | -4195.37 |
| CL9_559146837PSV_F0660246CCAM01820P1 | Passadumkeag_ccam_2 | 9 | 1821 | -4195.37 |

| | | | | |
|---|---|---|---|---|
| CL9_559147031PSV_F0660246CCAM01820P1 | Passadumkeag_ccam_2 | 10 | 1821 | -4195.37 |
| CL9_559147353PSV_F0660246CCAM02820P1 | Uncle_Zeke_Island | 1 | 1821 | -4195.37 |
| CL9_559147421PSV_F0660246CCAM02820P1 | Uncle_Zeke_Island | 2 | 1821 | -4195.37 |
| CL9_559147485PSV_F0660246CCAM02820P1 | Uncle_Zeke_Island | 3 | 1821 | -4195.37 |
| CL9_559147720PSV_F0660246CCAM02820P1 | Uncle_Zeke_Island | 4 | 1821 | -4195.37 |
| CL9_559147788PSV_F0660246CCAM02820P1 | Uncle_Zeke_Island | 5 | 1821 | -4195.37 |
| CL9_559147852PSV_F0660246CCAM02820P1 | Uncle_Zeke_Island | 6 | 1821 | -4195.37 |
| CL9_559148087PSV_F0660246CCAM02820P1 | Uncle_Zeke_Island | 7 | 1821 | -4195.37 |
| CL9_559148155PSV_F0660246CCAM02820P1 | Uncle_Zeke_Island | 8 | 1821 | -4195.37 |
| CL9_559148219PSV_F0660246CCAM02820P1 | Uncle_Zeke_Island | 9 | 1821 | -4195.37 |
| CL9_559148413PSV_F0660246CCAM02820P1 | Uncle_Zeke_Island | 10 | 1821 | -4195.37 |
| CL9_559239668PSV_F0660246CCAM02822P1 | Hypocrites_Ledges | 1 | 1822 | -4195.37 |
| CL9_559239736PSV_F0660246CCAM02822P1 | Hypocrites_Ledges | 2 | 1822 | -4195.37 |
| CL9_559239800PSV_F0660246CCAM02822P1 | Hypocrites_Ledges | 3 | 1822 | -4195.37 |
| CL9_559240034PSV_F0660246CCAM02822P1 | Hypocrites_Ledges | 4 | 1822 | -4195.37 |
| CL9_559240102PSV_F0660246CCAM02822P1 | Hypocrites_Ledges | 5 | 1822 | -4195.37 |
| CL9_559240166PSV_F0660246CCAM02822P1 | Hypocrites_Ledges | 6 | 1822 | -4195.37 |
| CL9_559240532PSV_F0660246CCAM02822P1 | Hypocrites_Ledges | 9 | 1822 | -4195.37 |
| CL9_559240694PSV_F0660246CCAM02822P1 | Hypocrites_Ledges | 10 | 1822 | -4195.37 |
| CL9_559678610PSV_F0660384CCAM01827P1 | Troll_Valley_ccam | 1 | 1827 | -4191.61 |
| CL9_559678678PSV_F0660384CCAM01827P1 | Troll_Valley_ccam | 2 | 1827 | -4191.61 |
| CL9_559678742PSV_F0660384CCAM01827P1 | Troll_Valley_ccam | 3 | 1827 | -4191.61 |
| CL9_559678806PSV_F0660384CCAM01827P1 | Troll_Valley_ccam | 4 | 1827 | -4191.61 |
| CL9_559678967PSV_F0660384CCAM01827P1 | Troll_Valley_ccam | 5 | 1827 | -4191.61 |
| CL9_559679278PSV_F0660384CCAM02827P1 | Sherwood_Forest | 1 | 1827 | -4191.61 |
| CL9_559679346PSV_F0660384CCAM02827P1 | Sherwood_Forest | 2 | 1827 | -4191.61 |
| CL9_559679410PSV_F0660384CCAM02827P1 | Sherwood_Forest | 3 | 1827 | -4191.61 |
| CL9_559679474PSV_F0660384CCAM02827P1 | Sherwood_Forest | 4 | 1827 | -4191.61 |
| CL9_559679636PSV_F0660384CCAM02827P1 | Sherwood_Forest | 5 | 1827 | -4191.61 |
| CL9_559679947PSV_F0660384CCAM03827P1 | Tableland | 1 | 1827 | -4191.61 |
| CL9_559680023PSV_F0660384CCAM03827P1 | Tableland | 2 | 1827 | -4191.61 |
| CL9_559680089PSV_F0660384CCAM03827P1 | Tableland | 3 | 1827 | -4191.61 |
| CL9_559680155PSV_F0660384CCAM03827P1 | Tableland | 4 | 1827 | -4191.61 |
| CL9_559680221PSV_F0660384CCAM03827P1 | Tableland | 5 | 1827 | -4191.61 |
| CL9_559680287PSV_F0660384CCAM03827P1 | Tableland | 6 | 1827 | -4191.61 |
| CL9_559680419PSV_F0660384CCAM03827P1 | Tableland | 8 | 1827 | -4191.61 |
| CL9_559680485PSV_F0660384CCAM03827P1 | Tableland | 9 | 1827 | -4191.61 |
| CL9_559680649PSV_F0660384CCAM03827P1 | Tableland | 10 | 1827 | -4191.61 |
| CL9_559769251PSV_F0660450CCAM01828P1 | Bonney_Woods | 1 | 1828 | -4190.84 |
| CL9_559769327PSV_F0660450CCAM01828P1 | Bonney_Woods | 2 | 1828 | -4190.84 |
| CL9_559769459PSV_F0660450CCAM01828P1 | Bonney_Woods | 4 | 1828 | -4190.84 |
| CL9_559769525PSV_F0660450CCAM01828P1 | Bonney_Woods | 5 | 1828 | -4190.84 |
| CL9_559770180PSV_F0660450CCAM02828P1 | Hawk_Mountain | 1 | 1828 | -4190.84 |
| CL9_559770322PSV_F0660450CCAM02828P1 | Hawk_Mountain | 3 | 1828 | -4190.84 |
| CL9_559770586PSV_F0660450CCAM02828P1 | Hawk_Mountain | 5 | 1828 | -4190.84 |
| CL9_559949308PSV_F0660856CCAM01830P1 | Collingham | 1 | 1830 | -4187.56 |
| CL9_559949384PSV_F0660856CCAM01830P1 | Collingham | 2 | 1830 | -4187.56 |
| CL9_559949448PSV_F0660856CCAM01830P1 | Collingham | 3 | 1830 | -4187.56 |
| CL9_559949513PSV_F0660856CCAM01830P1 | Collingham | 4 | 1830 | -4187.56 |
| CL9_559949753PSV_F0660856CCAM01830P1 | Collingham | 5 | 1830 | -4187.56 |
| CL9_559949828PSV_F0660856CCAM01830P1 | Collingham | 6 | 1830 | -4187.56 |
| CL9_559949894PSV_F0660856CCAM01830P1 | Collingham | 7 | 1830 | -4187.56 |
| CL9_559949960PSV_F0660856CCAM01830P1 | Collingham | 8 | 1830 | -4187.56 |
| CL9_559950027PSV_F0660856CCAM01830P1 | Collingham | 9 | 1830 | -4187.56 |
| CL9_559950225PSV_F0660856CCAM01830P1 | Collingham | 10 | 1830 | -4187.56 |
| CL9_560038676PSV_F0660952CCAM01831P1 | Katberg | 1 | 1831 | -4185.06 |
| CL9_560038744PSV_F0660952CCAM01831P1 | Katberg | 2 | 1831 | -4185.06 |
| CL9_560038808PSV_F0660952CCAM01831P1 | Katberg | 3 | 1831 | -4185.06 |
| CL9_560038871PSV_F0660952CCAM01831P1 | Katberg | 4 | 1831 | -4185.06 |

| | | | | |
|---|---|---|---|---|
| CL9_560039065PSV_F0660952CCAM01831P1 | Katberg | 5 | 1831 | -4185.06 |
| CL9_560039130PSV_F0660952CCAM01831P1 | Katberg | 6 | 1831 | -4185.06 |
| CL9_560039194PSV_F0660952CCAM01831P1 | Katberg | 7 | 1831 | -4185.06 |
| CL9_560039258PSV_F0660952CCAM01831P1 | Katberg | 8 | 1831 | -4185.06 |
| CL9_560039322PSV_F0660952CCAM01831P1 | Katberg | 9 | 1831 | -4185.06 |
| CL9_560039516PSV_F0660952CCAM01831P1 | Katberg | 10 | 1831 | -4185.06 |
| CL9_560480929PSV_F0661112CCAM01836P1 | Ecca | 1 | 1836 | -4182.32 |
| CL9_560480997PSV_F0661112CCAM01836P1 | Ecca | 2 | 1836 | -4182.32 |
| CL9_560481061PSV_F0661112CCAM01836P1 | Ecca | 3 | 1836 | -4182.32 |
| CL9_560481125PSV_F0661112CCAM01836P1 | Ecca | 4 | 1836 | -4182.32 |
| CL9_560481253PSV_F0661112CCAM01836P1 | Ecca | 6 | 1836 | -4182.32 |
| CL9_560481317PSV_F0661112CCAM01836P1 | Ecca | 7 | 1836 | -4182.32 |
| CL9_560481381PSV_F0661112CCAM01836P1 | Ecca | 8 | 1836 | -4182.32 |
| CL9_560481445PSV_F0661112CCAM01836P1 | Ecca | 9 | 1836 | -4182.32 |
| CL9_560481639PSV_F0661112CCAM01836P1 | Ecca | 10 | 1836 | -4182.32 |
| CL9_560482058PSV_F0661112CCAM02836P1 | Lucknow | 2 | 1836 | -4182.32 |
| CL9_560482186PSV_F0661112CCAM02836P1 | Lucknow | 4 | 1836 | -4182.32 |
| CL9_560482380PSV_F0661112CCAM02836P1 | Lucknow | 5 | 1836 | -4182.32 |
| CL9_560660340PSV_F0661332CCAM01838P1 | Duitschland | 1 | 1838 | -4180.47 |
| CL9_560660416PSV_F0661332CCAM01838P1 | Duitschland | 2 | 1838 | -4180.47 |
| CL9_560660482PSV_F0661332CCAM01838P1 | Duitschland | 3 | 1838 | -4180.47 |
| CL9_560660713PSV_F0661332CCAM01838P1 | Duitschland | 4 | 1838 | -4180.47 |
| CL9_560660782PSV_F0661332CCAM01838P1 | Duitschland | 5 | 1838 | -4180.47 |
| CL9_560660848PSV_F0661332CCAM01838P1 | Duitschland | 6 | 1838 | -4180.47 |
| CL9_560661079PSV_F0661332CCAM01838P1 | Duitschland | 7 | 1838 | -4180.47 |
| CL9_560661146PSV_F0661332CCAM01838P1 | Duitschland | 8 | 1838 | -4180.47 |
| CL9_560661342PSV_F0661332CCAM01838P1 | Duitschland | 9 | 1838 | -4180.47 |
| CL9_560661691PSV_F0661332CCAM02838P1 | Chesire | 1 | 1838 | -4180.47 |
| CL9_560661766PSV_F0661332CCAM02838P1 | Chesire | 2 | 1838 | -4180.47 |
| CL9_560661832PSV_F0661332CCAM02838P1 | Chesire | 3 | 1838 | -4180.47 |
| CL9_560661898PSV_F0661332CCAM02838P1 | Chesire | 4 | 1838 | -4180.47 |
| CL9_560662094PSV_F0661332CCAM02838P1 | Chesire | 5 | 1838 | -4180.47 |
| CL9_560927580PSV_F0661332CCAM01841P1 | Bokkeveld | 1 | 1842 | -4180.47 |
| CL9_560927655PSV_F0661332CCAM01841P1 | Bokkeveld | 2 | 1842 | -4180.47 |
| CL9_560927720PSV_F0661332CCAM01841P1 | Bokkeveld | 3 | 1842 | -4180.47 |
| CL9_560927785PSV_F0661332CCAM01841P1 | Bokkeveld | 4 | 1842 | -4180.47 |
| CL9_560927949PSV_F0661332CCAM01841P1 | Bokkeveld | 5 | 1842 | -4180.47 |
| CL9_560928014PSV_F0661332CCAM01841P1 | Bokkeveld | 6 | 1842 | -4180.47 |
| CL9_560928079PSV_F0661332CCAM01841P1 | Bokkeveld | 7 | 1842 | -4180.47 |
| CL9_560928144PSV_F0661332CCAM01841P1 | Bokkeveld | 8 | 1842 | -4180.47 |
| CL9_560928209PSV_F0661332CCAM01841P1 | Bokkeveld | 9 | 1842 | -4180.47 |
| CL9_560928373PSV_F0661332CCAM01841P1 | Bokkeveld | 10 | 1842 | -4180.47 |
| CL9_560928761PSV_F0661332CCAM02841P1 | Buffalo_Springs | 2 | 1842 | -4180.47 |
| CL9_560928957PSV_F0661332CCAM02841P1 | Buffalo_Springs | 3 | 1842 | -4180.47 |
| CL9_560929025PSV_F0661332CCAM02841P1 | Buffalo_Springs | 4 | 1842 | -4180.47 |
| CL9_560929188PSV_F0661332CCAM02841P1 | Buffalo_Springs | 5 | 1842 | -4180.47 |
| CL9_561104806PSV_F0661332CCAM01843P1 | Woodlands | 2 | 1843 | -4180.47 |
| CL9_561104871PSV_F0661332CCAM01843P1 | Woodlands | 3 | 1843 | -4180.47 |
| CL9_561104937PSV_F0661332CCAM01843P1 | Woodlands | 4 | 1843 | -4180.47 |
| CL9_561105133PSV_F0661332CCAM01843P1 | Woodlands | 5 | 1843 | -4180.47 |
| CL9_561105201PSV_F0661332CCAM01843P1 | Woodlands | 6 | 1843 | -4180.47 |
| CL9_561105266PSV_F0661332CCAM01843P1 | Woodlands | 7 | 1843 | -4180.47 |
| CL9_561105331PSV_F0661332CCAM01843P1 | Woodlands | 8 | 1843 | -4180.47 |
| CL9_561105396PSV_F0661332CCAM01843P1 | Woodlands | 9 | 1843 | -4180.47 |
| CL9_561105592PSV_F0661332CCAM01843P1 | Woodlands | 10 | 1843 | -4180.47 |
| CL9_561105912PSV_F0661332CCAM02843P1 | Montecristo | 1 | 1843 | -4180.47 |
| CL9_561105988PSV_F0661332CCAM02843P1 | Montecristo | 2 | 1843 | -4180.47 |
| CL9_561106184PSV_F0661332CCAM02843P1 | Montecristo | 3 | 1843 | -4180.47 |
| CL9_561106252PSV_F0661332CCAM02843P1 | Montecristo | 4 | 1843 | -4180.47 |

| | | | | |
|---|---|---|---|---|
| CL9_561106448PSV_F0661332CCAM02843P1 | Montecristo | 5 | 1843 | -4180.47 |
| CL9_561286465PSV_F0661342CCAM01845P1 | Bulawayo | 1 | 1845 | -4180.43 |
| CL9_561286541PSV_F0661342CCAM01845P1 | Bulawayo | 2 | 1845 | -4180.43 |
| CL9_561286707PSV_F0661342CCAM01845P1 | Bulawayo | 3 | 1845 | -4180.43 |
| CL9_561286773PSV_F0661342CCAM01845P1 | Bulawayo | 4 | 1845 | -4180.43 |
| CL9_561286939PSV_F0661342CCAM01845P1 | Bulawayo | 5 | 1845 | -4180.43 |
| CL9_561287264PSV_F0661342CCAM02845P1 | Bushveld | 1 | 1845 | -4180.43 |
| CL9_561287336PSV_F0661342CCAM02845P1 | Bushveld | 2 | 1845 | -4180.43 |
| CL9_561287401PSV_F0661342CCAM02845P1 | Bushveld | 3 | 1845 | -4180.43 |
| CL9_561287466PSV_F0661342CCAM02845P1 | Bushveld | 4 | 1845 | -4180.43 |
| CL9_561287531PSV_F0661342CCAM02845P1 | Bushveld | 5 | 1845 | -4180.43 |
| CL9_561287767PSV_F0661342CCAM02845P1 | Bushveld | 6 | 1845 | -4180.43 |
| CL9_561287842PSV_F0661342CCAM02845P1 | Bushveld | 7 | 1845 | -4180.43 |
| CL9_561287907PSV_F0661342CCAM02845P1 | Bushveld | 8 | 1845 | -4180.43 |
| CL9_561287973PSV_F0661342CCAM02845P1 | Bushveld | 9 | 1845 | -4180.43 |
| CL9_561288136PSV_F0661342CCAM02845P1 | Bushveld | 10 | 1845 | -4180.43 |
| CL9_561556036PSV_F0661516CCAM01848P1 | Boomplaas | 2 | 1848 | -4177.26 |
| CL9_561556100PSV_F0661516CCAM01848P1 | Boomplaas | 3 | 1848 | -4177.26 |
| CL9_561556164PSV_F0661516CCAM01848P1 | Boomplaas | 4 | 1848 | -4177.26 |
| CL9_561556357PSV_F0661516CCAM01848P1 | Boomplaas | 5 | 1848 | -4177.26 |
| CL9_561556669PSV_F0661516CCAM02848P1 | Buck_Reef | 1 | 1848 | -4177.26 |
| CL9_561556802PSV_F0661516CCAM02848P1 | Buck_Reef | 3 | 1848 | -4177.26 |
| CL9_561556866PSV_F0661516CCAM02848P1 | Buck_Reef | 4 | 1848 | -4177.26 |
| CL9_561557060PSV_F0661516CCAM02848P1 | Buck_Reef | 5 | 1848 | -4177.26 |
| CL9_561722238PSV_F0661654CCAM02850P1 | Wolkberg | 1 | 1850 | -4176.74 |
| CL9_561722313PSV_F0661654CCAM02850P1 | Wolkberg | 2 | 1850 | -4176.74 |
| CL9_561722445PSV_F0661654CCAM02850P1 | Wolkberg | 4 | 1850 | -4176.74 |
| CL9_561722641PSV_F0661654CCAM02850P1 | Wolkberg | 5 | 1850 | -4176.74 |
| CL9_561902781PSV_F0661804CCAM01852P1 | Balfour | 1 | 1852 | -4174.11 |
| CL9_561902849PSV_F0661804CCAM01852P1 | Balfour | 2 | 1852 | -4174.11 |
| CL9_561902913PSV_F0661804CCAM01852P1 | Balfour | 3 | 1852 | -4174.11 |
| CL9_561902976PSV_F0661804CCAM01852P1 | Balfour | 4 | 1852 | -4174.11 |
| CL9_561903040PSV_F0661804CCAM01852P1 | Balfour | 5 | 1852 | -4174.11 |
| CL9_561903103PSV_F0661804CCAM01852P1 | Balfour | 6 | 1852 | -4174.11 |
| CL9_561903167PSV_F0661804CCAM01852P1 | Balfour | 7 | 1852 | -4174.11 |
| CL9_561903230PSV_F0661804CCAM01852P1 | Balfour | 8 | 1852 | -4174.11 |
| CL9_561903294PSV_F0661804CCAM01852P1 | Balfour | 9 | 1852 | -4174.11 |
| CL9_561903488PSV_F0661804CCAM01852P1 | Balfour | 10 | 1852 | -4174.11 |
| CL9_561903830PSV_F0661804CCAM02852P1 | Rippon | 1 | 1852 | -4174.11 |
| CL9_561903898PSV_F0661804CCAM02852P1 | Rippon | 2 | 1852 | -4174.11 |
| CL9_561903962PSV_F0661804CCAM02852P1 | Rippon | 3 | 1852 | -4174.11 |
| CL9_561904026PSV_F0661804CCAM02852P1 | Rippon | 4 | 1852 | -4174.11 |
| CL9_561904220PSV_F0661804CCAM02852P1 | Rippon | 5 | 1852 | -4174.11 |
| CL9_562263349PSV_F0661804CCAM01856P1 | Hartley | 5 | 1856 | -4174.11 |
| CL9_562263424PSV_F0661804CCAM01856P1 | Hartley | 6 | 1856 | -4174.11 |
| CL9_562263490PSV_F0661804CCAM01856P1 | Hartley | 7 | 1856 | -4174.11 |
| CL9_562263556PSV_F0661804CCAM01856P1 | Hartley | 8 | 1856 | -4174.11 |
| CL9_562263622PSV_F0661804CCAM01856P1 | Hartley | 9 | 1856 | -4174.11 |
| CL9_562264484PSV_F0661804CCAM02856P1 | Hooggenoeg | 4 | 1856 | -4174.11 |
| CL9_562264719PSV_F0661804CCAM02856P1 | Hooggenoeg | 5 | 1856 | -4174.11 |
| CL9_562790943PSV_F0661804CCAM01861P1 | Schmidtsdrif | 1 | 1862 | -4174.11 |
| CL9_562791011PSV_F0661804CCAM01861P1 | Schmidtsdrif | 2 | 1862 | -4174.11 |
| CL9_562791075PSV_F0661804CCAM01861P1 | Schmidtsdrif | 3 | 1862 | -4174.11 |
| CL9_562791139PSV_F0661804CCAM01861P1 | Schmidtsdrif | 4 | 1862 | -4174.11 |
| CL9_562791333PSV_F0661804CCAM01861P1 | Schmidtsdrif | 5 | 1862 | -4174.11 |
| CL9_562792535PSV_F0661804CCAM03861P1 | Estecourt | 1 | 1862 | -4174.11 |
| CL9_562792611PSV_F0661804CCAM03861P1 | Estecourt | 2 | 1862 | -4174.11 |
| CL9_562792775PSV_F0661804CCAM03861P1 | Estecourt | 3 | 1862 | -4174.11 |
| CL9_562792841PSV_F0661804CCAM03861P1 | Estecourt | 4 | 1862 | -4174.11 |

| | | | | |
|---|---|---|---|---|
| CL9_562793005PSV_F0661804CCAM03861P1 | Estecourt | 5 | 1862 | -4174.11 |
| CL9_563058703PSV_F0661994CCAM01865P1 | Barberton | 1 | 1865 | -4174.66 |
| CL9_563058778PSV_F0661994CCAM01865P1 | Barberton | 2 | 1865 | -4174.66 |
| CL9_563058844PSV_F0661994CCAM01865P1 | Barberton | 3 | 1865 | -4174.66 |
| CL9_563058910PSV_F0661994CCAM01865P1 | Barberton | 4 | 1865 | -4174.66 |
| CL9_563059146PSV_F0661994CCAM01865P1 | Barberton | 5 | 1865 | -4174.66 |
| CL9_563059221PSV_F0661994CCAM01865P1 | Barberton | 6 | 1865 | -4174.66 |
| CL9_563059287PSV_F0661994CCAM01865P1 | Barberton | 7 | 1865 | -4174.66 |
| CL9_563059483PSV_F0661994CCAM01865P1 | Barberton | 8 | 1865 | -4174.66 |
| CL9_563059551PSV_F0661994CCAM01865P1 | Barberton | 9 | 1865 | -4174.66 |
| CL9_563059747PSV_F0661994CCAM01865P1 | Barberton | 10 | 1865 | -4174.66 |
| CL9_563501922PSV_F0662312CCAM01870P1 | Waboomberg | 1 | 1870 | -4170.47 |
| CL9_563501997PSV_F0662312CCAM01870P1 | Waboomberg | 2 | 1870 | -4170.47 |
| CL9_563502063PSV_F0662312CCAM01870P1 | Waboomberg | 3 | 1870 | -4170.47 |
| CL9_563502129PSV_F0662312CCAM01870P1 | Waboomberg | 4 | 1870 | -4170.47 |
| CL9_563589410PSV_F0662312CCAM02870P1 | Platberg_DRT | 1 | 1871 | -4170.47 |
| CL9_563589485PSV_F0662312CCAM02870P1 | Platberg_DRT | 2 | 1871 | -4170.47 |
| CL9_563589551PSV_F0662312CCAM02870P1 | Platberg_DRT | 3 | 1871 | -4170.47 |
| CL9_563589781PSV_F0662312CCAM02870P1 | Platberg_DRT | 4 | 1871 | -4170.47 |
| CL9_563589849PSV_F0662312CCAM02870P1 | Platberg_DRT | 5 | 1871 | -4170.47 |
| CL9_563589915PSV_F0662312CCAM02870P1 | Platberg_DRT | 6 | 1871 | -4170.47 |
| CL9_563590145PSV_F0662312CCAM02870P1 | Platberg_DRT | 7 | 1871 | -4170.47 |
| CL9_563590213PSV_F0662312CCAM02870P1 | Platberg_DRT | 8 | 1871 | -4170.47 |
| CL9_563590409PSV_F0662312CCAM02870P1 | Platberg_DRT | 9 | 1871 | -4170.47 |
| CL9_563678042PSV_F0662414CCAM01872P1 | Fort_Brown | 1 | 1872 | -4168.45 |
| CL9_563678183PSV_F0662414CCAM01872P1 | Fort_Brown | 3 | 1872 | -4168.45 |
| CL9_563678315PSV_F0662414CCAM01872P1 | Fort_Brown | 5 | 1872 | -4168.45 |
| CL9_563678381PSV_F0662414CCAM01872P1 | Fort_Brown | 6 | 1872 | -4168.45 |
| CL9_563678619PSV_F0662414CCAM01872P1 | Fort_Brown | 7 | 1872 | -4168.45 |
| CL9_563678694PSV_F0662414CCAM01872P1 | Fort_Brown | 8 | 1872 | -4168.45 |
| CL9_563678760PSV_F0662414CCAM01872P1 | Fort_Brown | 9 | 1872 | -4168.45 |
| CL9_563678925PSV_F0662414CCAM01872P1 | Fort_Brown | 10 | 1872 | -4168.45 |
| CL9_563680013PSV_F0662414CCAM03872P1 | Fairfield | 2 | 1872 | -4168.45 |
| CL9_563680078PSV_F0662414CCAM03872P1 | Fairfield | 3 | 1872 | -4168.45 |
| CL9_563680315PSV_F0662414CCAM03872P1 | Fairfield | 4 | 1872 | -4168.45 |
| CL9_563680390PSV_F0662414CCAM03872P1 | Fairfield | 5 | 1872 | -4168.45 |
| CL9_563680456PSV_F0662414CCAM03872P1 | Fairfield | 6 | 1872 | -4168.45 |
| CL9_563680767PSV_F0662414CCAM03872P1 | Fairfield | 8 | 1872 | -4168.45 |
| CL9_563680833PSV_F0662414CCAM03872P1 | Fairfield | 9 | 1872 | -4168.45 |
| CL9_563946230PSV_F0662430CCAM01875P1 | Reliance | 1 | 1875 | -4168.34 |
| CL9_563946306PSV_F0662430CCAM01875P1 | Reliance | 2 | 1875 | -4168.34 |
| CL9_563946372PSV_F0662430CCAM01875P1 | Reliance | 3 | 1875 | -4168.34 |
| CL9_563946438PSV_F0662430CCAM01875P1 | Reliance | 4 | 1875 | -4168.34 |
| CL9_563946675PSV_F0662430CCAM01875P1 | Reliance | 5 | 1875 | -4168.34 |
| CL9_563946751PSV_F0662430CCAM01875P1 | Reliance | 6 | 1875 | -4168.34 |
| CL9_563946817PSV_F0662430CCAM01875P1 | Reliance | 7 | 1875 | -4168.34 |
| CL9_563946883PSV_F0662430CCAM01875P1 | Reliance | 8 | 1875 | -4168.34 |
| CL9_563946949PSV_F0662430CCAM01875P1 | Reliance | 9 | 1875 | -4168.34 |
| CL9_563947145PSV_F0662430CCAM01875P1 | Reliance | 10 | 1875 | -4168.34 |
| CL9_563947464PSV_F0662430CCAM02875P1 | Dominion | 1 | 1875 | -4168.34 |
| CL9_563947539PSV_F0662430CCAM02875P1 | Dominion | 2 | 1875 | -4168.34 |
| CL9_563947604PSV_F0662430CCAM02875P1 | Dominion | 3 | 1875 | -4168.34 |
| CL9_563947669PSV_F0662430CCAM02875P1 | Dominion | 4 | 1875 | -4168.34 |
| CL9_563947905PSV_F0662430CCAM02875P1 | Dominion | 5 | 1875 | -4168.34 |
| CL9_563947980PSV_F0662430CCAM02875P1 | Dominion | 6 | 1875 | -4168.34 |
| CL9_563948046PSV_F0662430CCAM02875P1 | Dominion | 7 | 1875 | -4168.34 |
| CL9_563948112PSV_F0662430CCAM02875P1 | Dominion | 8 | 1875 | -4168.34 |
| CL9_563948373PSV_F0662430CCAM02875P1 | Dominion | 10 | 1875 | -4168.34 |
| CL9_564033556PSV_F0662430CCAM04875P1 | Middleton | 1 | 1876 | -4168.34 |

| | | | | |
|---|---|---|---|---|
| CL9_564033624PSV_F0662430CCAM04875P1 | Middleton | 2 | 1876 | -4168.34 |
| CL9_564033688PSV_F0662430CCAM04875P1 | Middleton | 3 | 1876 | -4168.34 |
| CL9_564033752PSV_F0662430CCAM04875P1 | Middleton | 4 | 1876 | -4168.34 |
| CL9_564033946PSV_F0662430CCAM04875P1 | Middleton | 5 | 1876 | -4168.34 |
| CL9_564034011PSV_F0662430CCAM04875P1 | Middleton | 6 | 1876 | -4168.34 |
| CL9_564034075PSV_F0662430CCAM04875P1 | Middleton | 7 | 1876 | -4168.34 |
| CL9_564034139PSV_F0662430CCAM04875P1 | Middleton | 8 | 1876 | -4168.34 |
| CL9_564034203PSV_F0662430CCAM04875P1 | Middleton | 9 | 1876 | -4168.34 |
| CL9_564034397PSV_F0662430CCAM04875P1 | Middleton | 10 | 1876 | -4168.34 |
| CL9_564121462PSV_F0662430CCAM01877P1 | Brenton | 2 | 1877 | -4168.34 |
| CL9_564121528PSV_F0662430CCAM01877P1 | Brenton | 3 | 1877 | -4168.34 |
| CL9_564121764PSV_F0662430CCAM01877P1 | Brenton | 4 | 1877 | -4168.34 |
| CL9_564121839PSV_F0662430CCAM01877P1 | Brenton | 5 | 1877 | -4168.34 |
| CL9_564121905PSV_F0662430CCAM01877P1 | Brenton | 6 | 1877 | -4168.34 |
| CL9_564122140PSV_F0662430CCAM01877P1 | Brenton | 7 | 1877 | -4168.34 |
| CL9_564122215PSV_F0662430CCAM01877P1 | Brenton | 8 | 1877 | -4168.34 |
| CL9_564122281PSV_F0662430CCAM01877P1 | Brenton | 9 | 1877 | -4168.34 |
| CL9_564122444PSV_F0662430CCAM01877P1 | Brenton | 10 | 1877 | -4168.34 |
| CL9_564122833PSV_F0662430CCAM02877P1 | Gamtoos | 2 | 1877 | -4168.34 |
| CL9_564122899PSV_F0662430CCAM02877P1 | Gamtoos | 3 | 1877 | -4168.34 |
| CL9_564122965PSV_F0662430CCAM02877P1 | Gamtoos | 4 | 1877 | -4168.34 |
| CL9_564123161PSV_F0662430CCAM02877P1 | Gamtoos | 5 | 1877 | -4168.34 |
| CL9_564301359PSV_F0670000CCAM04879P1 | Zululand | 1 | 1879 | -4166.6 |
| CL9_564301434PSV_F0670000CCAM04879P1 | Zululand | 2 | 1879 | -4166.6 |
| CL9_564301737PSV_F0670000CCAM04879P1 | Zululand | 4 | 1879 | -4166.6 |
| CL9_564301812PSV_F0670000CCAM04879P1 | Zululand | 5 | 1879 | -4166.6 |
| CL9_564301878PSV_F0670000CCAM04879P1 | Zululand | 6 | 1879 | -4166.6 |
| CL9_564302115PSV_F0670000CCAM04879P1 | Zululand | 7 | 1879 | -4166.6 |
| CL9_564302190PSV_F0670000CCAM04879P1 | Zululand | 8 | 1879 | -4166.6 |
| CL9_564302256PSV_F0670000CCAM04879P1 | Zululand | 9 | 1879 | -4166.6 |
| CL9_564302452PSV_F0670000CCAM04879P1 | Zululand | 10 | 1879 | -4166.6 |
| CL9_564478634PSV_F0670000CCAM06879P1 | Hexriver | 3 | 1881 | -4166.6 |
| CL9_564478871PSV_F0670000CCAM06879P1 | Hexriver | 4 | 1881 | -4166.6 |
| CL9_564478946PSV_F0670000CCAM06879P1 | Hexriver | 5 | 1881 | -4166.6 |
| CL9_564479012PSV_F0670000CCAM06879P1 | Hexriver | 6 | 1881 | -4166.6 |
| CL9_564479249PSV_F0670000CCAM06879P1 | Hexriver | 7 | 1881 | -4166.6 |
| CL9_564479324PSV_F0670000CCAM06879P1 | Hexriver | 8 | 1881 | -4166.6 |
| CL9_564479389PSV_F0670000CCAM06879P1 | Hexriver | 9 | 1881 | -4166.6 |
| CL9_564479585PSV_F0670000CCAM06879P1 | Hexriver | 10 | 1881 | -4166.6 |
| CL9_564829996PSV_F0670000CCAM03882P1 | Klipfonteinheuwel | 1 | 1885 | -4166.6 |
| CL9_564830071PSV_F0670000CCAM03882P1 | Klipfonteinheuwel | 2 | 1885 | -4166.6 |
| CL9_564830137PSV_F0670000CCAM03882P1 | Klipfonteinheuwel | 3 | 1885 | -4166.6 |
| CL9_564830203PSV_F0670000CCAM03882P1 | Klipfonteinheuwel | 4 | 1885 | -4166.6 |
| CL9_564830439PSV_F0670000CCAM03882P1 | Klipfonteinheuwel | 5 | 1885 | -4166.6 |
| CL9_564830514PSV_F0670000CCAM03882P1 | Klipfonteinheuwel | 6 | 1885 | -4166.6 |
| CL9_564830580PSV_F0670000CCAM03882P1 | Klipfonteinheuwel | 7 | 1885 | -4166.6 |
| CL9_564830646PSV_F0670000CCAM03882P1 | Klipfonteinheuwel | 8 | 1885 | -4166.6 |
| CL9_564830712PSV_F0670000CCAM03882P1 | Klipfonteinheuwel | 9 | 1885 | -4166.6 |
| CL9_564830908PSV_F0670000CCAM03882P1 | Klipfonteinheuwel | 10 | 1885 | -4166.6 |
| CL9_564831258PSV_F0670000CCAM04882P1 | Klippan | 1 | 1885 | -4166.6 |
| CL9_564831333PSV_F0670000CCAM04882P1 | Klippan | 2 | 1885 | -4166.6 |
| CL9_564831399PSV_F0670000CCAM04882P1 | Klippan | 3 | 1885 | -4166.6 |
| CL9_564831465PSV_F0670000CCAM04882P1 | Klippan | 4 | 1885 | -4166.6 |
| CL9_564831701PSV_F0670000CCAM04882P1 | Klippan | 5 | 1885 | -4166.6 |
| CL9_564831776PSV_F0670000CCAM04882P1 | Klippan | 6 | 1885 | -4166.6 |
| CL9_564831841PSV_F0670000CCAM04882P1 | Klippan | 7 | 1885 | -4166.6 |
| CL9_564831907PSV_F0670000CCAM04882P1 | Klippan | 8 | 1885 | -4166.6 |
| CL9_564831972PSV_F0670000CCAM04882P1 | Klippan | 9 | 1885 | -4166.6 |
| CL9_564832168PSV_F0670000CCAM04882P1 | Klippan | 10 | 1885 | -4166.6 |

| | | | | |
|---|---|---|---|---|
| CL9_564931420PSV_F0670000CCAM01886P1 | Reivilo | 3 | 1886 | -4166.6 |
| CL9_564931484PSV_F0670000CCAM01886P1 | Reivilo | 4 | 1886 | -4166.6 |
| CL9_565186677PSV_F0670216CCAM01889P1 | Lyttelton | 1 | 1890 | -4163.71 |
| CL9_565186815PSV_F0670216CCAM01889P1 | Lyttelton | 3 | 1890 | -4163.71 |
| CL9_565186879PSV_F0670216CCAM01889P1 | Lyttelton | 4 | 1890 | -4163.71 |
| CL9_565454246PSV_F0670650CCAM01892P1 | Stubenkop | 1 | 1892 | -4157.98 |
| CL9_565454378PSV_F0670650CCAM01892P1 | Stubenkop | 3 | 1892 | -4157.98 |
| CL9_565454442PSV_F0670650CCAM01892P1 | Stubenkop | 4 | 1892 | -4157.98 |
| CL9_565454636PSV_F0670650CCAM01892P1 | Stubenkop | 5 | 1892 | -4157.98 |
| CL9_565454989PSV_F0670650CCAM02892P1 | Drakensberg | 1 | 1892 | -4157.98 |
| CL9_565455064PSV_F0670650CCAM02892P1 | Drakensberg | 2 | 1892 | -4157.98 |
| CL9_565455130PSV_F0670650CCAM02892P1 | Drakensberg | 3 | 1892 | -4157.98 |
| CL9_565455367PSV_F0670650CCAM02892P1 | Drakensberg | 4 | 1892 | -4157.98 |
| CL9_565455442PSV_F0670650CCAM02892P1 | Drakensberg | 5 | 1892 | -4157.98 |
| CL9_565455508PSV_F0670650CCAM02892P1 | Drakensberg | 6 | 1892 | -4157.98 |
| CL9_565455744PSV_F0670650CCAM02892P1 | Drakensberg | 7 | 1892 | -4157.98 |
| CL9_565455819PSV_F0670650CCAM02892P1 | Drakensberg | 8 | 1892 | -4157.98 |
| CL9_565455885PSV_F0670650CCAM02892P1 | Drakensberg | 9 | 1892 | -4157.98 |
| CL9_565456081PSV_F0670650CCAM02892P1 | Drakensberg | 10 | 1892 | -4157.98 |
| CL9_565539988PSV_F0670650CCAM04892P1 | Pongola | 1 | 1893 | -4157.98 |
| CL9_565540056PSV_F0670650CCAM04892P1 | Pongola | 2 | 1893 | -4157.98 |
| CL9_565540120PSV_F0670650CCAM04892P1 | Pongola | 3 | 1893 | -4157.98 |
| CL9_565540184PSV_F0670650CCAM04892P1 | Pongola | 4 | 1893 | -4157.98 |
| CL9_565540418PSV_F0670650CCAM04892P1 | Pongola | 5 | 1893 | -4157.98 |
| CL9_565540486PSV_F0670650CCAM04892P1 | Pongola | 6 | 1893 | -4157.98 |
| CL9_565540550PSV_F0670650CCAM04892P1 | Pongola | 7 | 1893 | -4157.98 |
| CL9_565540614PSV_F0670650CCAM04892P1 | Pongola | 8 | 1893 | -4157.98 |
| CL9_565540677PSV_F0670650CCAM04892P1 | Pongola | 9 | 1893 | -4157.98 |
| CL9_565540912PSV_F0670650CCAM04892P1 | Pongola | 10 | 1893 | -4157.98 |
| CL9_565541050PSV_F0670650CCAM04892P1 | Pongola | 12 | 1893 | -4157.98 |
| CL9_565541244PSV_F0670650CCAM04892P1 | Pongola | 13 | 1893 | -4157.98 |
| CL9_565541308PSV_F0670650CCAM04892P1 | Pongola | 14 | 1893 | -4157.98 |
| CL9_565541372PSV_F0670650CCAM04892P1 | Pongola | 15 | 1893 | -4157.98 |
| CL9_565722290PSV_F0670806CCAM01895P1 | Naute | 1 | 1895 | -4157.16 |
| CL9_565722365PSV_F0670806CCAM01895P1 | Naute | 2 | 1895 | -4157.16 |
| CL9_565722430PSV_F0670806CCAM01895P1 | Naute | 3 | 1895 | -4157.16 |
| CL9_565722495PSV_F0670806CCAM01895P1 | Naute | 4 | 1895 | -4157.16 |
| CL9_565722731PSV_F0670806CCAM01895P1 | Naute | 5 | 1895 | -4157.16 |
| CL9_565722806PSV_F0670806CCAM01895P1 | Naute | 6 | 1895 | -4157.16 |
| CL9_565722871PSV_F0670806CCAM01895P1 | Naute | 7 | 1895 | -4157.16 |
| CL9_565722936PSV_F0670806CCAM01895P1 | Naute | 8 | 1895 | -4157.16 |
| CL9_565723002PSV_F0670806CCAM01895P1 | Naute | 9 | 1895 | -4157.16 |
| CL9_565723198PSV_F0670806CCAM01895P1 | Naute | 10 | 1895 | -4157.16 |
| CL9_565723509PSV_F0670806CCAM02895P1 | Mzamba | 1 | 1895 | -4157.16 |
| CL9_565723577PSV_F0670806CCAM02895P1 | Mzamba | 2 | 1895 | -4157.16 |
| CL9_565723641PSV_F0670806CCAM02895P1 | Mzamba | 3 | 1895 | -4157.16 |
| CL9_565723705PSV_F0670806CCAM02895P1 | Mzamba | 4 | 1895 | -4157.16 |
| CL9_565723939PSV_F0670806CCAM02895P1 | Mzamba | 5 | 1895 | -4157.16 |
| CL9_565724007PSV_F0670806CCAM02895P1 | Mzamba | 6 | 1895 | -4157.16 |
| CL9_565724071PSV_F0670806CCAM02895P1 | Mzamba | 7 | 1895 | -4157.16 |
| CL9_565724135PSV_F0670806CCAM02895P1 | Mzamba | 8 | 1895 | -4157.16 |
| CL9_565724705PSV_F0670806CCAM03895P1 | Nauga | 1 | 1895 | -4157.16 |
| CL9_565724773PSV_F0670806CCAM03895P1 | Nauga | 2 | 1895 | -4157.16 |
| CL9_565724837PSV_F0670806CCAM03895P1 | Nauga | 3 | 1895 | -4157.16 |
| CL9_565724901PSV_F0670806CCAM03895P1 | Nauga | 4 | 1895 | -4157.16 |
| CL9_565725095PSV_F0670806CCAM03895P1 | Nauga | 5 | 1895 | -4157.16 |
| CL9_566074014PSV_F0671016CCAM01899P1 | Muck | 1 | 1899 | -4157.04 |
| CL9_566074089PSV_F0671016CCAM01899P1 | Muck | 2 | 1899 | -4157.04 |
| CL9_566074154PSV_F0671016CCAM01899P1 | Muck | 3 | 1899 | -4157.04 |

| | | | | |
|---|---|---|---|---|
| CL9_566074220PSV_F0671016CCAM01899P1 | Muck | 4 | 1899 | -4157.04 |
| CL9_566074456PSV_F0671016CCAM01899P1 | Muck | 5 | 1899 | -4157.04 |
| CL9_566074531PSV_F0671016CCAM01899P1 | Muck | 6 | 1899 | -4157.04 |
| CL9_566074597PSV_F0671016CCAM01899P1 | Muck | 7 | 1899 | -4157.04 |
| CL9_566074662PSV_F0671016CCAM01899P1 | Muck | 8 | 1899 | -4157.04 |
| CL9_566074727PSV_F0671016CCAM01899P1 | Muck | 9 | 1899 | -4157.04 |
| CL9_566074890PSV_F0671016CCAM01899P1 | Muck | 10 | 1899 | -4157.04 |
| CL9_566075258PSV_F0671016CCAM02899P1 | Gala | 1 | 1899 | -4157.04 |
| CL9_566075334PSV_F0671016CCAM02899P1 | Gala | 2 | 1899 | -4157.04 |
| CL9_566075398PSV_F0671016CCAM02899P1 | Gala | 3 | 1899 | -4157.04 |
| CL9_566075497PSV_F0671016CCAM02899P1 | Gala | 4 | 1899 | -4157.04 |
| CL9_566075563PSV_F0671016CCAM02899P1 | Gala | 5 | 1899 | -4157.04 |
| CL9_566075628PSV_F0671016CCAM02899P1 | Gala | 6 | 1899 | -4157.04 |
| CL9_566075858PSV_F0671016CCAM02899P1 | Gala | 7 | 1899 | -4157.04 |
| CL9_566075927PSV_F0671016CCAM02899P1 | Gala | 8 | 1899 | -4157.04 |
| CL9_566076121PSV_F0671016CCAM02899P1 | Gala | 9 | 1899 | -4157.04 |
| CL9_566076460PSV_F0671016CCAM03899P1 | Lagavulin | 1 | 1899 | -4157.04 |
| CL9_566076535PSV_F0671016CCAM03899P1 | Lagavulin | 2 | 1899 | -4157.04 |
| CL9_566076600PSV_F0671016CCAM03899P1 | Lagavulin | 3 | 1899 | -4157.04 |
| CL9_566076665PSV_F0671016CCAM03899P1 | Lagavulin | 4 | 1899 | -4157.04 |
| CL9_566429159PSV_F0671238CCAM02902P1 | Trotternish | 1 | 1903 | -4154.2 |
| CL9_566429234PSV_F0671238CCAM02902P1 | Trotternish | 2 | 1903 | -4154.2 |
| CL9_566429299PSV_F0671238CCAM02902P1 | Trotternish | 3 | 1903 | -4154.2 |
| CL9_566429365PSV_F0671238CCAM02902P1 | Trotternish | 4 | 1903 | -4154.2 |
| CL9_566429561PSV_F0671238CCAM02902P1 | Trotternish | 5 | 1903 | -4154.2 |
| CL9_566429919PSV_F0671238CCAM03902P1 | Arran | 1 | 1903 | -4154.2 |
| CL9_566429987PSV_F0671238CCAM03902P1 | Arran | 2 | 1903 | -4154.2 |
| CL9_566430051PSV_F0671238CCAM03902P1 | Arran | 3 | 1903 | -4154.2 |
| CL9_566430114PSV_F0671238CCAM03902P1 | Arran | 4 | 1903 | -4154.2 |
| CL9_566430177PSV_F0671238CCAM03902P1 | Arran | 5 | 1903 | -4154.2 |
| CL9_566430241PSV_F0671238CCAM03902P1 | Arran | 6 | 1903 | -4154.2 |
| CL9_566430433PSV_F0671238CCAM03902P1 | Arran | 9 | 1903 | -4154.2 |
| CL9_566430497PSV_F0671238CCAM03902P1 | Arran | 10 | 1903 | -4154.2 |
| CL9_566430561PSV_F0671238CCAM03902P1 | Arran | 11 | 1903 | -4154.2 |
| CL9_566430625PSV_F0671238CCAM03902P1 | Arran | 12 | 1903 | -4154.2 |
| CL9_566430689PSV_F0671238CCAM03902P1 | Arran | 13 | 1903 | -4154.2 |
| CL9_566430753PSV_F0671238CCAM03902P1 | Arran | 14 | 1903 | -4154.2 |
| CL9_566430947PSV_F0671238CCAM03902P1 | Arran | 15 | 1903 | -4154.2 |
| CL9_566518041PSV_F0671358CCAM01904P1 | Oban | 1 | 1904 | -4154.57 |
| CL9_566518173PSV_F0671358CCAM01904P1 | Oban | 3 | 1904 | -4154.57 |
| CL9_566518236PSV_F0671358CCAM01904P1 | Oban | 4 | 1904 | -4154.57 |
| CL9_566518299PSV_F0671358CCAM01904P1 | Oban | 5 | 1904 | -4154.57 |
| CL9_566518363PSV_F0671358CCAM01904P1 | Oban | 6 | 1904 | -4154.57 |
| CL9_566518427PSV_F0671358CCAM01904P1 | Oban | 7 | 1904 | -4154.57 |
| CL9_566518491PSV_F0671358CCAM01904P1 | Oban | 8 | 1904 | -4154.57 |
| CL9_566518555PSV_F0671358CCAM01904P1 | Oban | 9 | 1904 | -4154.57 |
| CL9_566518749PSV_F0671358CCAM01904P1 | Oban | 10 | 1904 | -4154.57 |
| CL9_566519066PSV_F0671358CCAM02904P1 | Talisker | 1 | 1904 | -4154.57 |
| CL9_566519141PSV_F0671358CCAM02904P1 | Talisker | 2 | 1904 | -4154.57 |
| CL9_566519207PSV_F0671358CCAM02904P1 | Talisker | 3 | 1904 | -4154.57 |
| CL9_566519469PSV_F0671358CCAM02904P1 | Talisker | 5 | 1904 | -4154.57 |
| CL9_566519817PSV_F0671358CCAM03904P1 | Laphroaig | 1 | 1904 | -4154.57 |
| CL9_566519885PSV_F0671358CCAM03904P1 | Laphroaig | 2 | 1904 | -4154.57 |
| CL9_566519949PSV_F0671358CCAM03904P1 | Laphroaig | 3 | 1904 | -4154.57 |
| CL9_566520013PSV_F0671358CCAM03904P1 | Laphroaig | 4 | 1904 | -4154.57 |
| CL9_566520207PSV_F0671358CCAM03904P1 | Laphroaig | 5 | 1904 | -4154.57 |
| CL9_566691092PSV_F0671494CCAM01906P1 | Holirood | 2 | 1906 | -4152.91 |
| CL9_566691156PSV_F0671494CCAM01906P1 | Holirood | 3 | 1906 | -4152.91 |
| CL9_566691220PSV_F0671494CCAM01906P1 | Holirood | 4 | 1906 | -4152.91 |

| | | | | |
|---|---|---|---|---|
| CL9_566691284PSV_F0671494CCAM01906P1 | Holirood | 5 | 1906 | -4152.91 |
| CL9_566691348PSV_F0671494CCAM01906P1 | Holirood | 6 | 1906 | -4152.91 |
| CL9_566691412PSV_F0671494CCAM01906P1 | Holirood | 7 | 1906 | -4152.91 |
| CL9_566691476PSV_F0671494CCAM01906P1 | Holirood | 8 | 1906 | -4152.91 |
| CL9_566691734PSV_F0671494CCAM01906P1 | Holirood | 10 | 1906 | -4152.91 |
| CL9_566692077PSV_F0671494CCAM02906P1 | Haddo House | 1 | 1906 | -4152.91 |
| CL9_566692145PSV_F0671494CCAM02906P1 | Haddo House | 2 | 1906 | -4152.91 |
| CL9_566692209PSV_F0671494CCAM02906P1 | Haddo House | 3 | 1906 | -4152.91 |
| CL9_566692273PSV_F0671494CCAM02906P1 | Haddo House | 4 | 1906 | -4152.91 |
| CL9_566692337PSV_F0671494CCAM02906P1 | Haddo House | 5 | 1906 | -4152.91 |
| CL9_566692401PSV_F0671494CCAM02906P1 | Haddo House | 6 | 1906 | -4152.91 |
| CL9_566692465PSV_F0671494CCAM02906P1 | Haddo House | 7 | 1906 | -4152.91 |
| CL9_566692529PSV_F0671494CCAM02906P1 | Haddo House | 8 | 1906 | -4152.91 |
| CL9_566692593PSV_F0671494CCAM02906P1 | Haddo House | 9 | 1906 | -4152.91 |
| CL9_566692787PSV_F0671494CCAM02906P1 | Haddo House | 10 | 1906 | -4152.91 |
| CL9_568023096PSV_F0671762CCAM01921P1 | Haroldswick | 1 | 1921 | -4154.55 |
| CL9_568023227PSV_F0671762CCAM01921P1 | Haroldswick | 3 | 1921 | -4154.55 |
| CL9_568023290PSV_F0671762CCAM01921P1 | Haroldswick | 4 | 1921 | -4154.55 |
| CL9_568023353PSV_F0671762CCAM01921P1 | Haroldswick | 5 | 1921 | -4154.55 |
| CL9_568023416PSV_F0671762CCAM01921P1 | Haroldswick | 6 | 1921 | -4154.55 |
| CL9_568023610PSV_F0671762CCAM01921P1 | Haroldswick | 7 | 1921 | -4154.55 |
| CL9_568023675PSV_F0671762CCAM01921P1 | Haroldswick | 8 | 1921 | -4154.55 |
| CL9_568023738PSV_F0671762CCAM01921P1 | Haroldswick | 9 | 1921 | -4154.55 |
| CL9_568023801PSV_F0671762CCAM01921P1 | Haroldswick | 10 | 1921 | -4154.55 |
| CL9_568023864PSV_F0671762CCAM01921P1 | Haroldswick | 11 | 1921 | -4154.55 |
| CL9_568023927PSV_F0671762CCAM01921P1 | Haroldswick | 12 | 1921 | -4154.55 |
| CL9_568023991PSV_F0671762CCAM01921P1 | Haroldswick | 13 | 1921 | -4154.55 |
| CL9_568024054PSV_F0671762CCAM01921P1 | Haroldswick | 14 | 1921 | -4154.55 |
| CL9_568024117PSV_F0671762CCAM01921P1 | Haroldswick | 15 | 1921 | -4154.55 |
| CL9_568024180PSV_F0671762CCAM01921P1 | Haroldswick | 16 | 1921 | -4154.55 |
| CL9_568024437PSV_F0671762CCAM01921P1 | Haroldswick | 18 | 1921 | -4154.55 |
| CL9_568116741PSV_F0671762CCAM06921P1 | Islay | 1 | 1922 | -4154.55 |
| CL9_568116816PSV_F0671762CCAM06921P1 | Islay | 2 | 1922 | -4154.55 |
| CL9_568116879PSV_F0671762CCAM06921P1 | Islay | 3 | 1922 | -4154.55 |
| CL9_568116943PSV_F0671762CCAM06921P1 | Islay | 4 | 1922 | -4154.55 |
| CL9_568117137PSV_F0671762CCAM06921P1 | Islay | 5 | 1922 | -4154.55 |
| CL9_568117202PSV_F0671762CCAM06921P1 | Islay | 6 | 1922 | -4154.55 |
| CL9_568117265PSV_F0671762CCAM06921P1 | Islay | 7 | 1922 | -4154.55 |
| CL9_568117391PSV_F0671762CCAM06921P1 | Islay | 9 | 1922 | -4154.55 |
| CL9_568117585PSV_F0671762CCAM06921P1 | Islay | 10 | 1922 | -4154.55 |
| CL9_568118305PSV_F0671762CCAM07921P1 | Glen_Coe | 1 | 1922 | -4154.55 |
| CL9_568118438PSV_F0671762CCAM07921P1 | Glen_Coe | 3 | 1922 | -4154.55 |
| CL9_568118502PSV_F0671762CCAM07921P1 | Glen_Coe | 4 | 1922 | -4154.55 |
| CL9_568118566PSV_F0671762CCAM07921P1 | Glen_Coe | 5 | 1922 | -4154.55 |
| CL9_568118630PSV_F0671762CCAM07921P1 | Glen_Coe | 6 | 1922 | -4154.55 |
| CL9_568118694PSV_F0671762CCAM07921P1 | Glen_Coe | 7 | 1922 | -4154.55 |
| CL9_568118758PSV_F0671762CCAM07921P1 | Glen_Coe | 8 | 1922 | -4154.55 |
| CL9_568118822PSV_F0671762CCAM07921P1 | Glen_Coe | 9 | 1922 | -4154.55 |
| CL9_568119057PSV_F0671762CCAM07921P1 | Glen_Coe | 10 | 1922 | -4154.55 |
| CL9_568119125PSV_F0671762CCAM07921P1 | Glen_Coe | 11 | 1922 | -4154.55 |
| CL9_568119189PSV_F0671762CCAM07921P1 | Glen_Coe | 12 | 1922 | -4154.55 |
| CL9_568119253PSV_F0671762CCAM07921P1 | Glen_Coe | 13 | 1922 | -4154.55 |
| CL9_568119317PSV_F0671762CCAM07921P1 | Glen_Coe | 14 | 1922 | -4154.55 |
| CL9_568119381PSV_F0671762CCAM07921P1 | Glen_Coe | 15 | 1922 | -4154.55 |
| CL9_568119445PSV_F0671762CCAM07921P1 | Glen_Coe | 16 | 1922 | -4154.55 |
| CL9_568119509PSV_F0671762CCAM07921P1 | Glen_Coe | 17 | 1922 | -4154.55 |
| CL9_568119573PSV_F0671762CCAM07921P1 | Glen_Coe | 18 | 1922 | -4154.55 |
| CL9_568119637PSV_F0671762CCAM07921P1 | Glen_Coe | 19 | 1922 | -4154.55 |
| CL9_568119831PSV_F0671762CCAM07921P1 | Glen_Coe | 20 | 1922 | -4154.55 |

| | | | | |
|---|---|---|---|---|
| CL9_568378016PSV_F0671846CCAM01925P1 | Barra | 1 | 1925 | -4153.57 |
| CL9_568378091PSV_F0671846CCAM01925P1 | Barra | 2 | 1925 | -4153.57 |
| CL9_568378156PSV_F0671846CCAM01925P1 | Barra | 3 | 1925 | -4153.57 |
| CL9_568378406PSV_F0671846CCAM01925P1 | Barra | 4 | 1925 | -4153.57 |
| CL9_568378481PSV_F0671846CCAM01925P1 | Barra | 5 | 1925 | -4153.57 |
| CL9_568378546PSV_F0671846CCAM01925P1 | Barra | 6 | 1925 | -4153.57 |
| CL9_568378796PSV_F0671846CCAM01925P1 | Barra | 7 | 1925 | -4153.57 |
| CL9_568378871PSV_F0671846CCAM01925P1 | Barra | 8 | 1925 | -4153.57 |
| CL9_568378936PSV_F0671846CCAM01925P1 | Barra | 9 | 1925 | -4153.57 |
| CL9_568379146PSV_F0671846CCAM01925P1 | Barra | 10 | 1925 | -4153.57 |
| CL9_568379500PSV_F0671846CCAM02925P1 | Assynt | 1 | 1925 | -4153.57 |
| CL9_568379575PSV_F0671846CCAM02925P1 | Assynt | 2 | 1925 | -4153.57 |
| CL9_568379641PSV_F0671846CCAM02925P1 | Assynt | 3 | 1925 | -4153.57 |
| CL9_568379878PSV_F0671846CCAM02925P1 | Assynt | 4 | 1925 | -4153.57 |
| CL9_568379953PSV_F0671846CCAM02925P1 | Assynt | 5 | 1925 | -4153.57 |
| CL9_568380256PSV_F0671846CCAM02925P1 | Assynt | 7 | 1925 | -4153.57 |
| CL9_568380397PSV_F0671846CCAM02925P1 | Assynt | 9 | 1925 | -4153.57 |
| CL9_568380593PSV_F0671846CCAM02925P1 | Assynt | 10 | 1925 | -4153.57 |
| CL9_568467235PSV_F0671846CCAM03925P1 | Elgin | 1 | 1925 | -4153.57 |
| CL9_568467310PSV_F0671846CCAM03925P1 | Elgin | 2 | 1925 | -4153.57 |
| CL9_568467375PSV_F0671846CCAM03925P1 | Elgin | 3 | 1925 | -4153.57 |
| CL9_568467440PSV_F0671846CCAM03925P1 | Elgin | 4 | 1925 | -4153.57 |
| CL9_568467677PSV_F0671846CCAM03925P1 | Elgin | 5 | 1925 | -4153.57 |
| CL9_568467752PSV_F0671846CCAM03925P1 | Elgin | 6 | 1925 | -4153.57 |
| CL9_568467882PSV_F0671846CCAM03925P1 | Elgin | 8 | 1925 | -4153.57 |
| CL9_568467947PSV_F0671846CCAM03925P1 | Elgin | 9 | 1925 | -4153.57 |
| CL9_568468143PSV_F0671846CCAM03925P1 | Elgin | 10 | 1925 | -4153.57 |
| CL9_568556726PSV_F0671846CCAM01927P1 | Jura | 1 | 1927 | -4153.57 |
| CL9_568556802PSV_F0671846CCAM01927P1 | Jura | 2 | 1927 | -4153.57 |
| CL9_568556998PSV_F0671846CCAM01927P1 | Jura | 3 | 1927 | -4153.57 |
| CL9_568557066PSV_F0671846CCAM01927P1 | Jura | 4 | 1927 | -4153.57 |
| CL9_568557132PSV_F0671846CCAM01927P1 | Jura | 5 | 1927 | -4153.57 |
| CL9_568557368PSV_F0671846CCAM01927P1 | Jura | 6 | 1927 | -4153.57 |
| CL9_568557444PSV_F0671846CCAM01927P1 | Jura | 7 | 1927 | -4153.57 |
| CL9_568557510PSV_F0671846CCAM01927P1 | Jura | 8 | 1927 | -4153.57 |
| CL9_568557576PSV_F0671846CCAM01927P1 | Jura | 9 | 1927 | -4153.57 |
| CL9_568557772PSV_F0671846CCAM01927P1 | Jura | 10 | 1927 | -4153.57 |
| CL9_568558120PSV_F0671846CCAM02927P1 | Crinan | 1 | 1927 | -4153.57 |
| CL9_568558195PSV_F0671846CCAM02927P1 | Crinan | 2 | 1927 | -4153.57 |
| CL9_568558261PSV_F0671846CCAM02927P1 | Crinan | 3 | 1927 | -4153.57 |
| CL9_568558498PSV_F0671846CCAM02927P1 | Crinan | 4 | 1927 | -4153.57 |
| CL9_568558573PSV_F0671846CCAM02927P1 | Crinan | 5 | 1927 | -4153.57 |
| CL9_568558639PSV_F0671846CCAM02927P1 | Crinan | 6 | 1927 | -4153.57 |
| CL9_568558835PSV_F0671846CCAM02927P1 | Crinan | 7 | 1927 | -4153.57 |
| CL9_568558903PSV_F0671846CCAM02927P1 | Crinan | 8 | 1927 | -4153.57 |
| CL9_568558969PSV_F0671846CCAM02927P1 | Crinan | 9 | 1927 | -4153.57 |
| CL9_568559165PSV_F0671846CCAM02927P1 | Crinan | 10 | 1927 | -4153.57 |
| CL9_568653144PSV_F0671846CCAM05927P1 | Brodick | 2 | 1928 | -4153.57 |
| CL9_568653209PSV_F0671846CCAM05927P1 | Brodick | 3 | 1928 | -4153.57 |
| CL9_568653308PSV_F0671846CCAM05927P1 | Brodick | 4 | 1928 | -4153.57 |
| CL9_568653374PSV_F0671846CCAM05927P1 | Brodick | 5 | 1928 | -4153.57 |
| CL9_568653439PSV_F0671846CCAM05927P1 | Brodick | 6 | 1928 | -4153.57 |
| CL9_568653539PSV_F0671846CCAM05927P1 | Brodick | 7 | 1928 | -4153.57 |
| CL9_568653605PSV_F0671846CCAM05927P1 | Brodick | 8 | 1928 | -4153.57 |
| CL9_568653768PSV_F0671846CCAM05927P1 | Brodick | 9 | 1928 | -4153.57 |
| CL9_568746525PSV_F0672140CCAM01929P1 | Barraclough | 1 | 1929 | -4150.14 |
| CL9_568746593PSV_F0672140CCAM01929P1 | Barraclough | 2 | 1929 | -4150.14 |
| CL9_568746657PSV_F0672140CCAM01929P1 | Barraclough | 3 | 1929 | -4150.14 |
| CL9_568746721PSV_F0672140CCAM01929P1 | Barraclough | 4 | 1929 | -4150.14 |

| | | | | |
|---|---|---|---|---|
| CL9_568746785PSV_F0672140CCAM01929P1 | Barraclough | 5 | 1929 | -4150.14 |
| CL9_568746849PSV_F0672140CCAM01929P1 | Barraclough | 6 | 1929 | -4150.14 |
| CL9_568746913PSV_F0672140CCAM01929P1 | Barraclough | 7 | 1929 | -4150.14 |
| CL9_568746977PSV_F0672140CCAM01929P1 | Barraclough | 8 | 1929 | -4150.14 |
| CL9_568747041PSV_F0672140CCAM01929P1 | Barraclough | 9 | 1929 | -4150.14 |
| CL9_568747235PSV_F0672140CCAM01929P1 | Barraclough | 10 | 1929 | -4150.14 |
| CL9_568747570PSV_F0672140CCAM02929P1 | Banff | 1 | 1929 | -4150.14 |
| CL9_568747645PSV_F0672140CCAM02929P1 | Banff | 2 | 1929 | -4150.14 |
| CL9_568747776PSV_F0672140CCAM02929P1 | Banff | 4 | 1929 | -4150.14 |
| CL9_568748012PSV_F0672140CCAM02929P1 | Banff | 5 | 1929 | -4150.14 |
| CL9_568748087PSV_F0672140CCAM02929P1 | Banff | 6 | 1929 | -4150.14 |
| CL9_568748153PSV_F0672140CCAM02929P1 | Banff | 7 | 1929 | -4150.14 |
| CL9_568748219PSV_F0672140CCAM02929P1 | Banff | 8 | 1929 | -4150.14 |
| CL9_568748285PSV_F0672140CCAM02929P1 | Banff | 9 | 1929 | -4150.14 |
| CL9_568748890PSV_F0672140CCAM03929P1 | Bass_Rock | 2 | 1929 | -4150.14 |
| CL9_568748956PSV_F0672140CCAM03929P1 | Bass_Rock | 3 | 1929 | -4150.14 |
| CL9_568749022PSV_F0672140CCAM03929P1 | Bass_Rock | 4 | 1929 | -4150.14 |
| CL9_568749231PSV_F0672140CCAM03929P1 | Bass_Rock | 5 | 1929 | -4150.14 |
| CL9_569009200PSV_F0672420CCAM01931P1 | Canna | 1 | 1931 | -4149.29 |
| CL9_569009269PSV_F0672420CCAM01931P1 | Canna | 2 | 1931 | -4149.29 |
| CL9_569009333PSV_F0672420CCAM01931P1 | Canna | 3 | 1931 | -4149.29 |
| CL9_569009566PSV_F0672420CCAM01931P1 | Canna | 4 | 1931 | -4149.29 |
| CL9_569009631PSV_F0672420CCAM01931P1 | Canna | 5 | 1931 | -4149.29 |
| CL9_569009928PSV_F0672420CCAM01931P1 | Canna | 7 | 1931 | -4149.29 |
| CL9_569009993PSV_F0672420CCAM01931P1 | Canna | 8 | 1931 | -4149.29 |
| CL9_569010187PSV_F0672420CCAM01931P1 | Canna | 9 | 1931 | -4149.29 |
| CL9_569010534PSV_F0672420CCAM02931P1 | Aberfoyle | 1 | 1931 | -4149.29 |
| CL9_569010602PSV_F0672420CCAM02931P1 | Aberfoyle | 2 | 1931 | -4149.29 |
| CL9_569010666PSV_F0672420CCAM02931P1 | Aberfoyle | 3 | 1931 | -4149.29 |
| CL9_569010730PSV_F0672420CCAM02931P1 | Aberfoyle | 4 | 1931 | -4149.29 |
| CL9_569010924PSV_F0672420CCAM02931P1 | Aberfoyle | 5 | 1931 | -4149.29 |
| CL9_569094693PSV_F0672420CCAM03931P1 | Moar | 1 | 1931 | -4149.29 |
| CL9_569094761PSV_F0672420CCAM03931P1 | Moar | 2 | 1931 | -4149.29 |
| CL9_569094825PSV_F0672420CCAM03931P1 | Moar | 3 | 1931 | -4149.29 |
| CL9_569095055PSV_F0672420CCAM03931P1 | Moar | 4 | 1931 | -4149.29 |
| CL9_569095120PSV_F0672420CCAM03931P1 | Moar | 5 | 1931 | -4149.29 |
| CL9_569095184PSV_F0672420CCAM03931P1 | Moar | 6 | 1931 | -4149.29 |
| CL9_569095481PSV_F0672420CCAM03931P1 | Moar | 8 | 1931 | -4149.29 |
| CL9_569095675PSV_F0672420CCAM03931P1 | Moar | 9 | 1931 | -4149.29 |
| CL9_569096075PSV_F0672420CCAM04931P1 | Unst | 2 | 1931 | -4149.29 |
| CL9_569096139PSV_F0672420CCAM04931P1 | Unst | 3 | 1931 | -4149.29 |
| CL9_569096203PSV_F0672420CCAM04931P1 | Unst | 4 | 1931 | -4149.29 |
| CL9_569096410PSV_F0672420CCAM04931P1 | Unst | 5 | 1931 | -4149.29 |
| CL9_569096777PSV_F0672420CCAM05931P1 | Funzie | 1 | 1931 | -4149.29 |
| CL9_569096845PSV_F0672420CCAM05931P1 | Funzie | 2 | 1931 | -4149.29 |
| CL9_569096909PSV_F0672420CCAM05931P1 | Funzie | 3 | 1931 | -4149.29 |
| CL9_569096973PSV_F0672420CCAM05931P1 | Funzie | 4 | 1931 | -4149.29 |
| CL9_569097208PSV_F0672420CCAM05931P1 | Funzie | 5 | 1931 | -4149.29 |
| CL9_569097277PSV_F0672420CCAM05931P1 | Funzie | 6 | 1931 | -4149.29 |
| CL9_569097341PSV_F0672420CCAM05931P1 | Funzie | 7 | 1931 | -4149.29 |
| CL9_569097405PSV_F0672420CCAM05931P1 | Funzie | 8 | 1931 | -4149.29 |
| CL9_569097663PSV_F0672420CCAM05931P1 | Funzie | 10 | 1931 | -4149.29 |
| CL9_569182000PSV_F0672420CCAM01934P1 | Rhynie | 1 | 1934 | -4149.29 |
| CL9_569182068PSV_F0672420CCAM01934P1 | Rhynie | 2 | 1934 | -4149.29 |
| CL9_569182132PSV_F0672420CCAM01934P1 | Rhynie | 3 | 1934 | -4149.29 |
| CL9_569182365PSV_F0672420CCAM01934P1 | Rhynie | 4 | 1934 | -4149.29 |
| CL9_569182430PSV_F0672420CCAM01934P1 | Rhynie | 5 | 1934 | -4149.29 |
| CL9_569182494PSV_F0672420CCAM01934P1 | Rhynie | 6 | 1934 | -4149.29 |
| CL9_569182727PSV_F0672420CCAM01934P1 | Rhynie | 7 | 1934 | -4149.29 |

| | | | | |
|---|---|---|---|---|
| CL9_569182792PSV_F0672420CCAM01934P1 | Rhynie | 8 | 1934 | -4149.29 |
| CL9_569184549PSV_F0672420CCAM03934P1 | Macleans_Nose | 1 | 1934 | -4149.29 |
| CL9_569184617PSV_F0672420CCAM03934P1 | Macleans_Nose | 2 | 1934 | -4149.29 |
| CL9_569184681PSV_F0672420CCAM03934P1 | Macleans_Nose | 3 | 1934 | -4149.29 |
| CL9_569184916PSV_F0672420CCAM03934P1 | Macleans_Nose | 4 | 1934 | -4149.29 |
| CL9_569185045PSV_F0672420CCAM03934P1 | Macleans_Nose | 6 | 1934 | -4149.29 |
| CL9_569185280PSV_F0672420CCAM03934P1 | Macleans_Nose | 7 | 1934 | -4149.29 |
| CL9_569185345PSV_F0672420CCAM03934P1 | Macleans_Nose | 8 | 1934 | -4149.29 |
| CL9_569185539PSV_F0672420CCAM03934P1 | Macleans_Nose | 9 | 1934 | -4149.29 |
| CL9_569450077PSV_F0672420CCAM01937P1 | Funzie2 | 1 | 1937 | -4149.29 |
| CL9_569450145PSV_F0672420CCAM01937P1 | Funzie2 | 2 | 1937 | -4149.29 |
| CL9_569450208PSV_F0672420CCAM01937P1 | Funzie2 | 3 | 1937 | -4149.29 |
| CL9_569450314PSV_F0672420CCAM01937P1 | Funzie2 | 4 | 1937 | -4149.29 |
| CL9_569450377PSV_F0672420CCAM01937P1 | Funzie2 | 5 | 1937 | -4149.29 |
| CL9_569450440PSV_F0672420CCAM01937P1 | Funzie2 | 6 | 1937 | -4149.29 |
| CL9_569450541PSV_F0672420CCAM01937P1 | Funzie2 | 7 | 1937 | -4149.29 |
| CL9_569450604PSV_F0672420CCAM01937P1 | Funzie2 | 8 | 1937 | -4149.29 |
| CL9_569450798PSV_F0672420CCAM01937P1 | Funzie2 | 9 | 1937 | -4149.29 |
| CL9_569451143PSV_F0672420CCAM02937P1 | Ullapool | 1 | 1937 | -4149.29 |
| CL9_569451348PSV_F0672420CCAM02937P1 | Ullapool | 4 | 1937 | -4149.29 |
| CL9_569451556PSV_F0672420CCAM02937P1 | Ullapool | 5 | 1937 | -4149.29 |
| CL9_569451631PSV_F0672420CCAM02937P1 | Ullapool | 6 | 1937 | -4149.29 |
| CL9_569451697PSV_F0672420CCAM02937P1 | Ullapool | 7 | 1937 | -4149.29 |
| CL9_569451762PSV_F0672420CCAM02937P1 | Ullapool | 8 | 1937 | -4149.29 |
| CL9_569451827PSV_F0672420CCAM02937P1 | Ullapool | 9 | 1937 | -4149.29 |
| CL9_569452023PSV_F0672420CCAM02937P1 | Ullapool | 10 | 1937 | -4149.29 |
| CL9_569452357PSV_F0672420CCAM03937P1 | Macleans_Nose2 | 1 | 1937 | -4149.29 |
| CL9_569452426PSV_F0672420CCAM03937P1 | Macleans_Nose2 | 2 | 1937 | -4149.29 |
| CL9_569452490PSV_F0672420CCAM03937P1 | Macleans_Nose2 | 3 | 1937 | -4149.29 |
| CL9_569452595PSV_F0672420CCAM03937P1 | Macleans_Nose2 | 4 | 1937 | -4149.29 |
| CL9_569452659PSV_F0672420CCAM03937P1 | Macleans_Nose2 | 5 | 1937 | -4149.29 |
| CL9_569452723PSV_F0672420CCAM03937P1 | Macleans_Nose2 | 6 | 1937 | -4149.29 |
| CL9_569452953PSV_F0672420CCAM03937P1 | Macleans_Nose2 | 7 | 1937 | -4149.29 |
| CL9_569453018PSV_F0672420CCAM03937P1 | Macleans_Nose2 | 8 | 1937 | -4149.29 |
| CL9_569453211PSV_F0672420CCAM03937P1 | Macleans_Nose2 | 9 | 1937 | -4149.29 |
| CL9_570604021PSV_F0680000CCAM01950P1 | Killiecrankie | 1 | 1950 | -4146.66 |
| CL9_570604096PSV_F0680000CCAM01950P1 | Killiecrankie | 2 | 1950 | -4146.66 |
| CL9_570604166PSV_F0680000CCAM01950P1 | Killiecrankie | 3 | 1950 | -4146.66 |
| CL9_570604232PSV_F0680000CCAM01950P1 | Killiecrankie | 4 | 1950 | -4146.66 |
| CL9_570604297PSV_F0680000CCAM01950P1 | Killiecrankie | 5 | 1950 | -4146.66 |
| CL9_570604362PSV_F0680000CCAM01950P1 | Killiecrankie | 6 | 1950 | -4146.66 |
| CL9_570605094PSV_F0680000CCAM02950P1 | Bennachie | 1 | 1950 | -4146.66 |
| CL9_570605236PSV_F0680000CCAM02950P1 | Bennachie | 3 | 1950 | -4146.66 |
| CL9_570605302PSV_F0680000CCAM02950P1 | Bennachie | 4 | 1950 | -4146.66 |
| CL9_570605498PSV_F0680000CCAM02950P1 | Bennachie | 5 | 1950 | -4146.66 |
| CL9_571043990PSV_F0680214CCAM01954P1 | GlenRoy | 1 | 1955 | -4146.38 |
| CL9_571044058PSV_F0680214CCAM01954P1 | GlenRoy | 2 | 1955 | -4146.38 |
| CL9_571044122PSV_F0680214CCAM01954P1 | GlenRoy | 3 | 1955 | -4146.38 |
| CL9_571044186PSV_F0680214CCAM01954P1 | GlenRoy | 4 | 1955 | -4146.38 |
| CL9_571044250PSV_F0680214CCAM01954P1 | GlenRoy | 5 | 1955 | -4146.38 |
| CL9_571044314PSV_F0680214CCAM01954P1 | GlenRoy | 6 | 1955 | -4146.38 |
| CL9_571044378PSV_F0680214CCAM01954P1 | GlenRoy | 7 | 1955 | -4146.38 |
| CL9_571044506PSV_F0680214CCAM01954P1 | GlenRoy | 9 | 1955 | -4146.38 |
| CL9_571044667PSV_F0680214CCAM01954P1 | GlenRoy | 10 | 1955 | -4146.38 |
| CL9_571044988PSV_F0680214CCAM02954P1 | HarraEbb | 1 | 1955 | -4146.38 |
| CL9_571045056PSV_F0680214CCAM02954P1 | HarraEbb | 2 | 1955 | -4146.38 |
| CL9_571045120PSV_F0680214CCAM02954P1 | HarraEbb | 3 | 1955 | -4146.38 |
| CL9_571045184PSV_F0680214CCAM02954P1 | HarraEbb | 4 | 1955 | -4146.38 |
| CL9_571045248PSV_F0680214CCAM02954P1 | HarraEbb | 5 | 1955 | -4146.38 |

| | | | | |
|---|---|---|---|---|
| CL9_571045312PSV_F0680214CCAM02954P1 | HarraEbb | 6 | 1955 | -4146.38 |
| CL9_571045385PSV_F0680214CCAM02954P1 | HarraEbb | 7 | 1955 | -4146.38 |
| CL9_571045449PSV_F0680214CCAM02954P1 | HarraEbb | 8 | 1955 | -4146.38 |
| CL9_571045513PSV_F0680214CCAM02954P1 | HarraEbb | 9 | 1955 | -4146.38 |
| CL9_571045674PSV_F0680214CCAM02954P1 | HarraEbb | 10 | 1955 | -4146.38 |
| CL9_571046017PSV_F0680214CCAM03954P1 | Cocksburnpath | 1 | 1955 | -4146.38 |
| CL9_571046085PSV_F0680214CCAM03954P1 | Cocksburnpath | 2 | 1955 | -4146.38 |
| CL9_571046149PSV_F0680214CCAM03954P1 | Cocksburnpath | 3 | 1955 | -4146.38 |
| CL9_571046213PSV_F0680214CCAM03954P1 | Cocksburnpath | 4 | 1955 | -4146.38 |
| CL9_571046448PSV_F0680214CCAM03954P1 | Cocksburnpath | 5 | 1955 | -4146.38 |
| CL9_571046516PSV_F0680214CCAM03954P1 | Cocksburnpath | 6 | 1955 | -4146.38 |
| CL9_571046580PSV_F0680214CCAM03954P1 | Cocksburnpath | 7 | 1955 | -4146.38 |
| CL9_571046644PSV_F0680214CCAM03954P1 | Cocksburnpath | 8 | 1955 | -4146.38 |
| CL9_571222139PSV_F0680214CCAM01957P1 | SouthHarris | 1 | 1957 | -4146.38 |
| CL9_571222207PSV_F0680214CCAM01957P1 | SouthHarris | 2 | 1957 | -4146.38 |
| CL9_571222271PSV_F0680214CCAM01957P1 | SouthHarris | 3 | 1957 | -4146.38 |
| CL9_571222335PSV_F0680214CCAM01957P1 | SouthHarris | 4 | 1957 | -4146.38 |
| CL9_571222399PSV_F0680214CCAM01957P1 | SouthHarris | 5 | 1957 | -4146.38 |
| CL9_571222463PSV_F0680214CCAM01957P1 | SouthHarris | 6 | 1957 | -4146.38 |
| CL9_571222527PSV_F0680214CCAM01957P1 | SouthHarris | 7 | 1957 | -4146.38 |
| CL9_571222591PSV_F0680214CCAM01957P1 | SouthHarris | 8 | 1957 | -4146.38 |
| CL9_571222655PSV_F0680214CCAM01957P1 | SouthHarris | 9 | 1957 | -4146.38 |
| CL9_571222849PSV_F0680214CCAM01957P1 | SouthHarris | 10 | 1957 | -4146.38 |
| CL9_571223207PSV_F0680214CCAM02957P1 | Drummock | 1 | 1957 | -4146.38 |
| CL9_571223706PSV_F0680214CCAM02957P1 | Drummock | 6 | 1957 | -4146.38 |
| CL9_571403266PSV_F0680214CCAM01959P1 | Arnaboll | 1 | 1959 | -4146.38 |
| CL9_571403334PSV_F0680214CCAM01959P1 | Arnaboll | 2 | 1959 | -4146.38 |
| CL9_571403631PSV_F0680214CCAM01959P1 | Arnaboll | 4 | 1959 | -4146.38 |
| CL9_571403696PSV_F0680214CCAM01959P1 | Arnaboll | 5 | 1959 | -4146.38 |
| CL9_571403760PSV_F0680214CCAM01959P1 | Arnaboll | 6 | 1959 | -4146.38 |
| CL9_571403993PSV_F0680214CCAM01959P1 | Arnaboll | 7 | 1959 | -4146.38 |
| CL9_571404058PSV_F0680214CCAM01959P1 | Arnaboll | 8 | 1959 | -4146.38 |
| CL9_571575950PSV_F0680214CCAM03961P1 | Glenfinnan | 1 | 1961 | -4146.38 |
| CL9_571576019PSV_F0680214CCAM03961P1 | Glenfinnan | 2 | 1961 | -4146.38 |
| CL9_571576083PSV_F0680214CCAM03961P1 | Glenfinnan | 3 | 1961 | -4146.38 |
| CL9_571576283PSV_F0680214CCAM03961P1 | Glenfinnan | 4 | 1961 | -4146.38 |
| CL9_571576347PSV_F0680214CCAM03961P1 | Glenfinnan | 5 | 1961 | -4146.38 |
| CL9_571576411PSV_F0680214CCAM03961P1 | Glenfinnan | 6 | 1961 | -4146.38 |
| CL9_571576514PSV_F0680214CCAM03961P1 | Glenfinnan | 7 | 1961 | -4146.38 |
| CL9_571576578PSV_F0680214CCAM03961P1 | Glenfinnan | 8 | 1961 | -4146.38 |
| CL9_571577087PSV_F0680214CCAM04961P1 | SkaraBrae | 1 | 1961 | -4146.38 |
| CL9_571577156PSV_F0680214CCAM04961P1 | SkaraBrae | 2 | 1961 | -4146.38 |
| CL9_571577220PSV_F0680214CCAM04961P1 | SkaraBrae | 3 | 1961 | -4146.38 |
| CL9_571577420PSV_F0680214CCAM04961P1 | SkaraBrae | 4 | 1961 | -4146.38 |
| CL9_571577484PSV_F0680214CCAM04961P1 | SkaraBrae | 5 | 1961 | -4146.38 |
| CL9_571577548PSV_F0680214CCAM04961P1 | SkaraBrae | 6 | 1961 | -4146.38 |
| CL9_571577653PSV_F0680214CCAM04961P1 | SkaraBrae | 7 | 1961 | -4146.38 |
| CL9_571577717PSV_F0680214CCAM04961P1 | SkaraBrae | 8 | 1961 | -4146.38 |
| CL9_571577879PSV_F0680214CCAM04961P1 | SkaraBrae | 9 | 1961 | -4146.38 |
| CL9_571936881PSV_F0680580CCAM01965P1 | Newmachar | 1 | 1965 | -4146.8 |
| CL9_571936957PSV_F0680580CCAM01965P1 | Newmachar | 2 | 1965 | -4146.8 |
| CL9_571937023PSV_F0680580CCAM01965P1 | Newmachar | 3 | 1965 | -4146.8 |
| CL9_571937089PSV_F0680580CCAM01965P1 | Newmachar | 4 | 1965 | -4146.8 |
| CL9_571937189PSV_F0680580CCAM01965P1 | Newmachar | 5 | 1965 | -4146.8 |
| CL9_571937255PSV_F0680580CCAM01965P1 | Newmachar | 6 | 1965 | -4146.8 |
| CL9_571937506PSV_F0680580CCAM01965P1 | Newmachar | 7 | 1965 | -4146.8 |
| CL9_571938007PSV_F0680580CCAM01965P1 | Newmachar | 11 | 1965 | -4146.8 |
| CL9_571938073PSV_F0680580CCAM01965P1 | Newmachar | 12 | 1965 | -4146.8 |
| CL9_571938318PSV_F0680580CCAM01965P1 | Newmachar | 13 | 1965 | -4146.8 |

| | | | | |
|---|---|---|---|---|
| CL9_571938393PSV_F0680580CCAM01965P1 | Newmachar | 14 | 1965 | -4146.8 |
| CL9_571938669PSV_F0680580CCAM01965P1 | Newmachar | 16 | 1965 | -4146.8 |
| CL9_572022761PSV_F0680580CCAM01966P1 | LakeOrcadie | 2 | 1966 | -4146.8 |
| CL9_572022827PSV_F0680580CCAM01966P1 | LakeOrcadie | 3 | 1966 | -4146.8 |
| CL9_572022927PSV_F0680580CCAM01966P1 | LakeOrcadie | 4 | 1966 | -4146.8 |
| CL9_572023059PSV_F0680580CCAM01966P1 | LakeOrcadie | 6 | 1966 | -4146.8 |
| CL9_572023225PSV_F0680580CCAM01966P1 | LakeOrcadie | 8 | 1966 | -4146.8 |
| CL9_572023429PSV_F0680580CCAM01966P1 | LakeOrcadie | 9 | 1966 | -4146.8 |
| CL9_572023760PSV_F0680580CCAM02966P1 | Forties | 1 | 1966 | -4146.8 |
| CL9_572023828PSV_F0680580CCAM02966P1 | Forties | 2 | 1966 | -4146.8 |
| CL9_572023892PSV_F0680580CCAM02966P1 | Forties | 3 | 1966 | -4146.8 |
| CL9_572023956PSV_F0680580CCAM02966P1 | Forties | 4 | 1966 | -4146.8 |
| CL9_572024150PSV_F0680580CCAM02966P1 | Forties | 5 | 1966 | -4146.8 |
| CL9_572112505PSV_F0680580CCAM03966P1 | LochCarron | 1 | 1967 | -4146.8 |
| CL9_572112573PSV_F0680580CCAM03966P1 | LochCarron | 2 | 1967 | -4146.8 |
| CL9_572112637PSV_F0680580CCAM03966P1 | LochCarron | 3 | 1967 | -4146.8 |
| CL9_572112701PSV_F0680580CCAM03966P1 | LochCarron | 4 | 1967 | -4146.8 |
| CL9_572112765PSV_F0680580CCAM03966P1 | LochCarron | 5 | 1967 | -4146.8 |
| CL9_572113020PSV_F0680580CCAM03966P1 | LochCarron | 6 | 1967 | -4146.8 |
| CL9_572113088PSV_F0680580CCAM03966P1 | LochCarron | 7 | 1967 | -4146.8 |
| CL9_572113152PSV_F0680580CCAM03966P1 | LochCarron | 8 | 1967 | -4146.8 |
| CL9_572113216PSV_F0680580CCAM03966P1 | LochCarron | 9 | 1967 | -4146.8 |
| CL9_572113424PSV_F0680580CCAM03966P1 | LochCarron | 10 | 1967 | -4146.8 |
| CL9_572114165PSV_F0680580CCAM04966P1 | VillageBay | 5 | 1967 | -4146.8 |
| CL9_572215337PSV_F0680580CCAM01968P1 | SmooCave | 1 | 1968 | -4146.8 |
| CL9_572215412PSV_F0680580CCAM01968P1 | SmooCave | 2 | 1968 | -4146.8 |
| CL9_572215478PSV_F0680580CCAM01968P1 | SmooCave | 3 | 1968 | -4146.8 |
| CL9_572215578PSV_F0680580CCAM01968P1 | SmooCave | 4 | 1968 | -4146.8 |
| CL9_572215644PSV_F0680580CCAM01968P1 | SmooCave | 5 | 1968 | -4146.8 |
| CL9_572215710PSV_F0680580CCAM01968P1 | SmooCave | 6 | 1968 | -4146.8 |
| CL9_572215810PSV_F0680580CCAM01968P1 | SmooCave | 7 | 1968 | -4146.8 |
| CL9_572215876PSV_F0680580CCAM01968P1 | SmooCave | 8 | 1968 | -4146.8 |
| CL9_572216077PSV_F0680580CCAM01968P1 | SmooCave | 9 | 1968 | -4146.8 |
| CL9_572216416PSV_F0680580CCAM02968P1 | StAndrews | 1 | 1968 | -4146.8 |
| CL9_572216484PSV_F0680580CCAM02968P1 | StAndrews | 2 | 1968 | -4146.8 |
| CL9_572216557PSV_F0680580CCAM02968P1 | StAndrews | 3 | 1968 | -4146.8 |
| CL9_572216872PSV_F0680580CCAM02968P1 | StAndrews | 5 | 1968 | -4146.8 |
| CL9_572216940PSV_F0680580CCAM02968P1 | StAndrews | 6 | 1968 | -4146.8 |
| CL9_572217068PSV_F0680580CCAM02968P1 | StAndrews | 8 | 1968 | -4146.8 |
| CL9_572217132PSV_F0680580CCAM02968P1 | StAndrews | 9 | 1968 | -4146.8 |
| CL9_572217339PSV_F0680580CCAM02968P1 | StAndrews | 10 | 1968 | -4146.8 |
| CL9_572286622PSV_F0680580CCAM03968P1 | Yesnaby | 1 | 1969 | -4146.8 |
| CL9_572286696PSV_F0680580CCAM03968P1 | Yesnaby | 2 | 1969 | -4146.8 |
| CL9_572286761PSV_F0680580CCAM03968P1 | Yesnaby | 3 | 1969 | -4146.8 |
| CL9_572287374PSV_F0680580CCAM04968P1 | Dingwall | 1 | 1969 | -4146.8 |
| CL9_572287442PSV_F0680580CCAM04968P1 | Dingwall | 2 | 1969 | -4146.8 |
| CL9_572287506PSV_F0680580CCAM04968P1 | Dingwall | 3 | 1969 | -4146.8 |
| CL9_572287609PSV_F0680580CCAM04968P1 | Dingwall | 4 | 1969 | -4146.8 |
| CL9_572287673PSV_F0680580CCAM04968P1 | Dingwall | 5 | 1969 | -4146.8 |
| CL9_572287737PSV_F0680580CCAM04968P1 | Dingwall | 6 | 1969 | -4146.8 |
| CL9_572287835PSV_F0680580CCAM04968P1 | Dingwall | 7 | 1969 | -4146.8 |
| CL9_572287899PSV_F0680580CCAM04968P1 | Dingwall | 8 | 1969 | -4146.8 |
| CL9_572288101PSV_F0680580CCAM04968P1 | Dingwall | 9 | 1969 | -4146.8 |
| CL9_572377016PSV_F0680580CCAM06968P1 | Fladda | 1 | 1970 | -4146.8 |
| CL9_572377092PSV_F0680580CCAM06968P1 | Fladda | 2 | 1970 | -4146.8 |
| CL9_572377158PSV_F0680580CCAM06968P1 | Fladda | 3 | 1970 | -4146.8 |
| CL9_572377224PSV_F0680580CCAM06968P1 | Fladda | 4 | 1970 | -4146.8 |
| CL9_572377420PSV_F0680580CCAM06968P1 | Fladda | 5 | 1970 | -4146.8 |
| CL9_572377488PSV_F0680580CCAM06968P1 | Fladda | 6 | 1970 | -4146.8 |

| | | | | |
|---|---|---|---|---|
| CL9_572377554PSV_F0680580CCAM06968P1 | Fladda | 7 | 1970 | -4146.8 |
| CL9_572556420PSV_F0680580CCAM02972P1 | Peterculter | 1 | 1972 | -4146.8 |
| CL9_572556552PSV_F0680580CCAM02972P1 | Peterculter | 3 | 1972 | -4146.8 |
| CL9_572556616PSV_F0680580CCAM02972P1 | Peterculter | 4 | 1972 | -4146.8 |
| CL9_572556744PSV_F0680580CCAM02972P1 | Peterculter | 6 | 1972 | -4146.8 |
| CL9_572556936PSV_F0680580CCAM02972P1 | Peterculter | 9 | 1972 | -4146.8 |
| CL9_572557129PSV_F0680580CCAM02972P1 | Peterculter | 10 | 1972 | -4146.8 |
| CL9_572740363PSV_F0680580CCAM01974P1 | Larbet | 1 | 1974 | -4146.8 |
| CL9_572740438PSV_F0680580CCAM01974P1 | Larbet | 2 | 1974 | -4146.8 |
| CL9_572740504PSV_F0680580CCAM01974P1 | Larbet | 3 | 1974 | -4146.8 |
| CL9_572740604PSV_F0680580CCAM01974P1 | Larbet | 4 | 1974 | -4146.8 |
| CL9_572740800PSV_F0680580CCAM01974P1 | Larbet | 5 | 1974 | -4146.8 |
| CL9_572740868PSV_F0680580CCAM01974P1 | Larbet | 6 | 1974 | -4146.8 |
| CL9_572740968PSV_F0680580CCAM01974P1 | Larbet | 7 | 1974 | -4146.8 |
| CL9_572741034PSV_F0680580CCAM01974P1 | Larbet | 8 | 1974 | -4146.8 |
| CL9_572741230PSV_F0680580CCAM01974P1 | Larbet | 9 | 1974 | -4146.8 |
| CL9_572741550PSV_F0680580CCAM02974P1 | Benbecula | 1 | 1974 | -4146.8 |
| CL9_572741618PSV_F0680580CCAM02974P1 | Benbecula | 2 | 1974 | -4146.8 |
| CL9_572741784PSV_F0680580CCAM02974P1 | Benbecula | 4 | 1974 | -4146.8 |
| CL9_572741977PSV_F0680580CCAM02974P1 | Benbecula | 5 | 1974 | -4146.8 |
| CL9_572742404PSV_F0680580CCAM02974P1 | Benbecula | 9 | 1974 | -4146.8 |
| CL9_572909789PSV_F0680580CCAM03974P1 | Sleat | 1 | 1976 | -4146.8 |
| CL9_572909857PSV_F0680580CCAM03974P1 | Sleat | 2 | 1976 | -4146.8 |
| CL9_572909921PSV_F0680580CCAM03974P1 | Sleat | 3 | 1976 | -4146.8 |
| CL9_572910219PSV_F0680580CCAM03974P1 | Sleat | 5 | 1976 | -4146.8 |
| CL9_572910287PSV_F0680580CCAM03974P1 | Sleat | 6 | 1976 | -4146.8 |
| CL9_572910351PSV_F0680580CCAM03974P1 | Sleat | 7 | 1976 | -4146.8 |
| CL9_572910415PSV_F0680580CCAM03974P1 | Sleat | 8 | 1976 | -4146.8 |
| CL9_573086524PSV_F0680580CCAM04978P1 | UigHills | 1 | 1978 | -4146.8 |
| CL9_573086599PSV_F0680580CCAM04978P1 | UigHills | 2 | 1978 | -4146.8 |
| CL9_573086664PSV_F0680580CCAM04978P1 | UigHills | 3 | 1978 | -4146.8 |
| CL9_573086966PSV_F0680580CCAM04978P1 | UigHills | 5 | 1978 | -4146.8 |
| CL9_573087107PSV_F0680580CCAM04978P1 | UigHills | 7 | 1978 | -4146.8 |
| CL9_573087173PSV_F0680580CCAM04978P1 | UigHills | 8 | 1978 | -4146.8 |
| CL9_573087435PSV_F0680580CCAM04978P1 | UigHills | 10 | 1978 | -4146.8 |
| CL9_573266331PSV_F0680580CCAM01980P1 | LakeOrcadie2 | 1 | 1980 | -4146.8 |
| CL9_573266406PSV_F0680580CCAM01980P1 | LakeOrcadie2 | 2 | 1980 | -4146.8 |
| CL9_573266472PSV_F0680580CCAM01980P1 | LakeOrcadie2 | 3 | 1980 | -4146.8 |
| CL9_573266710PSV_F0680580CCAM01980P1 | LakeOrcadie2 | 4 | 1980 | -4146.8 |
| CL9_573266782PSV_F0680580CCAM01980P1 | LakeOrcadie2 | 5 | 1980 | -4146.8 |
| CL9_573266848PSV_F0680580CCAM01980P1 | LakeOrcadie2 | 6 | 1980 | -4146.8 |
| CL9_573267088PSV_F0680580CCAM01980P1 | LakeOrcadie2 | 7 | 1980 | -4146.8 |
| CL9_573267361PSV_F0680580CCAM01980P1 | LakeOrcadie2 | 9 | 1980 | -4146.8 |
| CL9_573268538PSV_F0680580CCAM03980P1 | StKilda | 1 | 1980 | -4146.8 |
| CL9_573268606PSV_F0680580CCAM03980P1 | StKilda | 2 | 1980 | -4146.8 |
| CL9_573268940PSV_F0680580CCAM03980P1 | StKilda | 5 | 1980 | -4146.8 |
| CL9_573356162PSV_F0680580CCAM01981P1 | portion_drop | 1 | 1981 | -4146.8 |
| CL9_573356237PSV_F0680580CCAM01981P1 | portion_drop | 2 | 1981 | -4146.8 |
| CL9_573356613PSV_F0680580CCAM01981P1 | portion_drop | 5 | 1981 | -4146.8 |
| CL9_573356909PSV_F0680580CCAM01981P1 | portion_drop | 7 | 1981 | -4146.8 |
| CL9_573356976PSV_F0680580CCAM01981P1 | portion_drop | 8 | 1981 | -4146.8 |
| CL9_573357172PSV_F0680580CCAM01981P1 | portion_drop | 9 | 1981 | -4146.8 |
| CL9_573973711PSV_F0681232CCAM01988P1 | NorthHarris | 1 | 1988 | -4150.64 |
| CL9_573973779PSV_F0681232CCAM01988P1 | NorthHarris | 2 | 1988 | -4150.64 |
| CL9_573973843PSV_F0681232CCAM01988P1 | NorthHarris | 3 | 1988 | -4150.64 |
| CL9_573973907PSV_F0681232CCAM01988P1 | NorthHarris | 4 | 1988 | -4150.64 |
| CL9_573974101PSV_F0681232CCAM01988P1 | NorthHarris | 5 | 1988 | -4150.64 |
| CL9_574241832PSV_F0681626CCAM01991P1 | Seaforth_Head | 1 | 1991 | -4152.64 |
| CL9_574241901PSV_F0681626CCAM01991P1 | Seaforth_Head | 2 | 1991 | -4152.64 |

| | | | | |
|---|---|---|---|---|
| CL9_574241965PSV_F0681626CCAM01991P1 | Seaforth_Head | 3 | 1991 | -4152.64 |
| CL9_574242029PSV_F0681626CCAM01991P1 | Seaforth_Head | 4 | 1991 | -4152.64 |
| CL9_574242223PSV_F0681626CCAM01991P1 | Seaforth_Head | 5 | 1991 | -4152.64 |
| CL9_574242554PSV_F0681626CCAM02991P1 | Canisp | 1 | 1991 | -4152.64 |
| CL9_574242629PSV_F0681626CCAM02991P1 | Canisp | 2 | 1991 | -4152.64 |
| CL9_574242695PSV_F0681626CCAM02991P1 | Canisp | 3 | 1991 | -4152.64 |
| CL9_574242761PSV_F0681626CCAM02991P1 | Canisp | 4 | 1991 | -4152.64 |
| CL9_574242957PSV_F0681626CCAM02991P1 | Canisp | 5 | 1991 | -4152.64 |
| CL9_574422260PSV_F0681816CCAM01993P1 | StirlingCastle | 1 | 1993 | -4152.25 |
| CL9_574422324PSV_F0681816CCAM01993P1 | StirlingCastle | 2 | 1993 | -4152.25 |
| CL9_574422388PSV_F0681816CCAM01993P1 | StirlingCastle | 3 | 1993 | -4152.25 |
| CL9_574422622PSV_F0681816CCAM01993P1 | StirlingCastle | 4 | 1993 | -4152.25 |
| CL9_574422690PSV_F0681816CCAM01993P1 | StirlingCastle | 5 | 1993 | -4152.25 |
| CL9_574422754PSV_F0681816CCAM01993P1 | StirlingCastle | 6 | 1993 | -4152.25 |
| CL9_574422818PSV_F0681816CCAM01993P1 | StirlingCastle | 7 | 1993 | -4152.25 |
| CL9_574423076PSV_F0681816CCAM01993P1 | StirlingCastle | 9 | 1993 | -4152.25 |
| CL9_574423416PSV_F0681816CCAM02993P1 | Dunottar | 1 | 1993 | -4152.25 |
| CL9_574423491PSV_F0681816CCAM02993P1 | Dunottar | 2 | 1993 | -4152.25 |
| CL9_574423556PSV_F0681816CCAM02993P1 | Dunottar | 3 | 1993 | -4152.25 |
| CL9_574423903PSV_F0681816CCAM02993P1 | Dunottar | 5 | 1993 | -4152.25 |
| CL9_574423969PSV_F0681816CCAM02993P1 | Dunottar | 6 | 1993 | -4152.25 |
| CL9_574424297PSV_F0681816CCAM02993P1 | Dunottar | 8 | 1993 | -4152.25 |
| CL9_574424362PSV_F0681816CCAM02993P1 | Dunottar | 9 | 1993 | -4152.25 |
| CL9_574424572PSV_F0681816CCAM02993P1 | Dunottar | 10 | 1993 | -4152.25 |
| CL9_574595664PSV_F0682090CCAM01995P1 | Durness | 1 | 1995 | -4154.2 |
| CL9_574595732PSV_F0682090CCAM01995P1 | Durness | 2 | 1995 | -4154.2 |
| CL9_574595796PSV_F0682090CCAM01995P1 | Durness | 3 | 1995 | -4154.2 |
| CL9_574595860PSV_F0682090CCAM01995P1 | Durness | 4 | 1995 | -4154.2 |
| CL9_574596054PSV_F0682090CCAM01995P1 | Durness | 5 | 1995 | -4154.2 |
| CL9_574596119PSV_F0682090CCAM01995P1 | Durness | 6 | 1995 | -4154.2 |
| CL9_574596183PSV_F0682090CCAM01995P1 | Durness | 7 | 1995 | -4154.2 |
| CL9_574596247PSV_F0682090CCAM01995P1 | Durness | 8 | 1995 | -4154.2 |
| CL9_574596311PSV_F0682090CCAM01995P1 | Durness | 9 | 1995 | -4154.2 |
| CL9_574596505PSV_F0682090CCAM01995P1 | Durness | 10 | 1995 | -4154.2 |
| CL9_574596819PSV_F0682090CCAM02995P1 | Paisley | 1 | 1995 | -4154.2 |
| CL9_574596960PSV_F0682090CCAM02995P1 | Paisley | 3 | 1995 | -4154.2 |
| CL9_574597189PSV_F0682090CCAM02995P1 | Paisley | 4 | 1995 | -4154.2 |
| CL9_574597257PSV_F0682090CCAM02995P1 | Paisley | 5 | 1995 | -4154.2 |
| CL9_574597323PSV_F0682090CCAM02995P1 | Paisley | 6 | 1995 | -4154.2 |
| CL9_574597423PSV_F0682090CCAM02995P1 | Paisley | 7 | 1995 | -4154.2 |
| CL9_574597489PSV_F0682090CCAM02995P1 | Paisley | 8 | 1995 | -4154.2 |
| CL9_574597684PSV_F0682090CCAM02995P1 | Paisley | 9 | 1995 | -4154.2 |
| CL9_574598025PSV_F0682090CCAM03995P1 | FingalsCave | 1 | 1995 | -4154.2 |
| CL9_574863373PSV_F0682396CCAM01998P1 | Ochil | 1 | 1998 | -4155.42 |
| CL9_574863441PSV_F0682396CCAM01998P1 | Ochil | 2 | 1998 | -4155.42 |
| CL9_574863505PSV_F0682396CCAM01998P1 | Ochil | 3 | 1998 | -4155.42 |
| CL9_574863569PSV_F0682396CCAM01998P1 | Ochil | 4 | 1998 | -4155.42 |
| CL9_574863764PSV_F0682396CCAM01998P1 | Ochil | 5 | 1998 | -4155.42 |
| CL9_574864091PSV_F0682396CCAM02998P1 | Orval | 1 | 1998 | -4155.42 |
| CL9_574864166PSV_F0682396CCAM02998P1 | Orval | 2 | 1998 | -4155.42 |
| CL9_574864231PSV_F0682396CCAM02998P1 | Orval | 3 | 1998 | -4155.42 |
| CL9_574864297PSV_F0682396CCAM02998P1 | Orval | 4 | 1998 | -4155.42 |
| CL9_574864492PSV_F0682396CCAM02998P1 | Orval | 5 | 1998 | -4155.42 |
| CL9_574948301PSV_F0682484CCAM01999P1 | Mangersta | 1 | 1999 | -4156.99 |
| CL9_574948376PSV_F0682484CCAM01999P1 | Mangersta | 2 | 1999 | -4156.99 |
| CL9_574948507PSV_F0682484CCAM01999P1 | Mangersta | 4 | 1999 | -4156.99 |
| CL9_574948743PSV_F0682484CCAM01999P1 | Mangersta | 5 | 1999 | -4156.99 |
| CL9_574948818PSV_F0682484CCAM01999P1 | Mangersta | 6 | 1999 | -4156.99 |
| CL9_574948883PSV_F0682484CCAM01999P1 | Mangersta | 7 | 1999 | -4156.99 |

| | | | | |
|---|---|---|---|---|
| CL9_574948948PSV_F0682484CCAM01999P1 | Mangersta | 8 | 1999 | -4156.99 |
| CL9_574949178PSV_F0682484CCAM01999P1 | Mangersta | 10 | 1999 | -4156.99 |
| CL9_575035955PSV_F0682626CCAM01000P1 | Brora | 1 | 2000 | -4159.72 |
| CL9_575036031PSV_F0682626CCAM01000P1 | Brora | 2 | 2000 | -4159.72 |
| CL9_575036097PSV_F0682626CCAM01000P1 | Brora | 3 | 2000 | -4159.72 |
| CL9_575036163PSV_F0682626CCAM01000P1 | Brora | 4 | 2000 | -4159.72 |
| CL9_575036359PSV_F0682626CCAM01000P1 | Brora | 5 | 2000 | -4159.72 |
| CL9_575036674PSV_F0682626CCAM02000P1 | Appin | 1 | 2000 | -4159.72 |
| CL9_575036750PSV_F0682626CCAM02000P1 | Appin | 2 | 2000 | -4159.72 |
| CL9_575036816PSV_F0682626CCAM02000P1 | Appin | 3 | 2000 | -4159.72 |
| CL9_575036882PSV_F0682626CCAM02000P1 | Appin | 4 | 2000 | -4159.72 |
| CL9_575037078PSV_F0682626CCAM02000P1 | Appin | 5 | 2000 | -4159.72 |
| CL9_575215613PSV_F0682626CCAM01001P1 | Boddam | 1 | 2002 | -4159.72 |
| CL9_575215689PSV_F0682626CCAM01001P1 | Boddam | 2 | 2002 | -4159.72 |
| CL9_575215755PSV_F0682626CCAM01001P1 | Boddam | 3 | 2002 | -4159.72 |
| CL9_575215993PSV_F0682626CCAM01001P1 | Boddam | 4 | 2002 | -4159.72 |
| CL9_575216069PSV_F0682626CCAM01001P1 | Boddam | 5 | 2002 | -4159.72 |
| CL9_575216135PSV_F0682626CCAM01001P1 | Boddam | 6 | 2002 | -4159.72 |
| CL9_575216373PSV_F0682626CCAM01001P1 | Boddam | 7 | 2002 | -4159.72 |
| CL9_575216515PSV_F0682626CCAM01001P1 | Boddam | 9 | 2002 | -4159.72 |
| CL9_575216711PSV_F0682626CCAM01001P1 | Boddam | 10 | 2002 | -4159.72 |
| CL9_575216992PSV_F0682626CCAM02001P1 | SpurrOfEigg | 1 | 2002 | -4159.72 |
| CL9_575217322PSV_F0682626CCAM02001P1 | SpurrOfEigg | 4 | 2002 | -4159.72 |
| CL9_575217388PSV_F0682626CCAM02001P1 | SpurrOfEigg | 5 | 2002 | -4159.72 |
| CL9_575217454PSV_F0682626CCAM02001P1 | SpurrOfEigg | 6 | 2002 | -4159.72 |
| CL9_575217652PSV_F0682626CCAM02001P1 | SpurrOfEigg | 7 | 2002 | -4159.72 |
| CL9_575217723PSV_F0682626CCAM02001P1 | SpurrOfEigg | 8 | 2002 | -4159.72 |
| CL9_575217887PSV_F0682626CCAM02001P1 | SpurrOfEigg | 9 | 2002 | -4159.72 |
| CL9_575218330PSV_F0682626CCAM03001P1 | Kirkcudbright | 2 | 2002 | -4159.72 |
| CL9_575218458PSV_F0682626CCAM03001P1 | Kirkcudbright | 4 | 2002 | -4159.72 |
| CL9_575390771PSV_F0690000CCAM01004P1 | Mousa | 3 | 2004 | -4159.74 |
| CL9_575390836PSV_F0690000CCAM01004P1 | Mousa | 4 | 2004 | -4159.74 |
| CL9_575391031PSV_F0690000CCAM01004P1 | Mousa | 5 | 2004 | -4159.74 |
| CL9_575483225PSV_F0690408CCAM01005P1 | Straenraer | 1 | 2005 | -4164.62 |
| CL9_575483300PSV_F0690408CCAM01005P1 | Straenraer | 2 | 2005 | -4164.62 |
| CL9_575483366PSV_F0690408CCAM01005P1 | Straenraer | 3 | 2005 | -4164.62 |
| CL9_575483627PSV_F0690408CCAM01005P1 | Straenraer | 5 | 2005 | -4164.62 |
| CL9_575483951PSV_F0690408CCAM02005P1 | PortEllen | 1 | 2005 | -4164.62 |
| CL9_575484019PSV_F0690408CCAM02005P1 | PortEllen | 2 | 2005 | -4164.62 |
| CL9_575484083PSV_F0690408CCAM02005P1 | PortEllen | 3 | 2005 | -4164.62 |
| CL9_575484146PSV_F0690408CCAM02005P1 | PortEllen | 4 | 2005 | -4164.62 |
| CL9_575484339PSV_F0690408CCAM02005P1 | PortEllen | 5 | 2005 | -4164.62 |
| CL9_575569284PSV_F0690408CCAM01006P1 | Burntisland | 3 | 2006 | -4164.62 |
| CL9_575569347PSV_F0690408CCAM01006P1 | Burntisland | 4 | 2006 | -4164.62 |
| CL9_575569554PSV_F0690408CCAM01006P1 | Burntisland | 5 | 2006 | -4164.62 |
| CL9_575569879PSV_F0690408CCAM02006P1 | Burghead | 1 | 2006 | -4164.62 |
| CL9_575569951PSV_F0690408CCAM02006P1 | Burghead | 2 | 2006 | -4164.62 |
| CL9_575570017PSV_F0690408CCAM02006P1 | Burghead | 3 | 2006 | -4164.62 |
| CL9_575570083PSV_F0690408CCAM02006P1 | Burghead | 4 | 2006 | -4164.62 |
| CL9_575570149PSV_F0690408CCAM02006P1 | Burghead | 5 | 2006 | -4164.62 |
| CL9_575570388PSV_F0690408CCAM02006P1 | Burghead | 6 | 2006 | -4164.62 |
| CL9_575570530PSV_F0690408CCAM02006P1 | Burghead | 8 | 2006 | -4164.62 |
| CL9_575570596PSV_F0690408CCAM02006P1 | Burghead | 9 | 2006 | -4164.62 |
| CL9_575570793PSV_F0690408CCAM02006P1 | Burghead | 10 | 2006 | -4164.62 |
| CL9_575658222PSV_F0690408CCAM01007P1 | SullomVoe | 1 | 2007 | -4164.62 |
| CL9_575658517PSV_F0690408CCAM01007P1 | SullomVoe | 2 | 2007 | -4164.62 |
| CL9_575658813PSV_F0690408CCAM01007P1 | SullomVoe | 3 | 2007 | -4164.62 |
| CL9_575659207PSV_F0690408CCAM02007P1 | PapaStour | 2 | 2007 | -4164.62 |
| CL9_575659574PSV_F0690408CCAM02007P1 | PapaStour | 5 | 2007 | -4164.62 |

| | | | | |
|---|---|---|---|---|
| CL9_575659637PSV_F0690408CCAM02007P1 | PapaStour | 6 | 2007 | -4164.62 |
| CL9_575659873PSV_F0690408CCAM02007P1 | PapaStour | 7 | 2007 | -4164.62 |
| CL9_575659938PSV_F0690408CCAM02007P1 | PapaStour | 8 | 2007 | -4164.62 |
| CL9_575660132PSV_F0690408CCAM02007P1 | PapaStour | 9 | 2007 | -4164.62 |
| CL9_575745662PSV_F0690714CCAM01008P1 | BeinnDeargMhor | 1 | 2008 | -4160.97 |
| CL9_575745730PSV_F0690714CCAM01008P1 | BeinnDeargMhor | 2 | 2008 | -4160.97 |
| CL9_575745794PSV_F0690714CCAM01008P1 | BeinnDeargMhor | 3 | 2008 | -4160.97 |
| CL9_575745858PSV_F0690714CCAM01008P1 | BeinnDeargMhor | 4 | 2008 | -4160.97 |
| CL9_575746093PSV_F0690714CCAM01008P1 | BeinnDeargMhor | 5 | 2008 | -4160.97 |
| CL9_575746161PSV_F0690714CCAM01008P1 | BeinnDeargMhor | 6 | 2008 | -4160.97 |
| CL9_575746225PSV_F0690714CCAM01008P1 | BeinnDeargMhor | 7 | 2008 | -4160.97 |
| CL9_575746289PSV_F0690714CCAM01008P1 | BeinnDeargMhor | 8 | 2008 | -4160.97 |
| CL9_575746353PSV_F0690714CCAM01008P1 | BeinnDeargMhor | 9 | 2008 | -4160.97 |
| CL9_575746547PSV_F0690714CCAM01008P1 | BeinnDeargMhor | 10 | 2008 | -4160.97 |
| CL9_575746864PSV_F0690714CCAM02008P1 | DunCaan | 1 | 2008 | -4160.97 |
| CL9_575746996PSV_F0690714CCAM02008P1 | DunCaan | 3 | 2008 | -4160.97 |
| CL9_575747100PSV_F0690714CCAM02008P1 | DunCaan | 4 | 2008 | -4160.97 |
| CL9_575747164PSV_F0690714CCAM02008P1 | DunCaan | 5 | 2008 | -4160.97 |
| CL9_575747228PSV_F0690714CCAM02008P1 | DunCaan | 6 | 2008 | -4160.97 |
| CL9_575747327PSV_F0690714CCAM02008P1 | DunCaan | 7 | 2008 | -4160.97 |
| CL9_575747391PSV_F0690714CCAM02008P1 | DunCaan | 8 | 2008 | -4160.97 |
| CL9_575747585PSV_F0690714CCAM02008P1 | DunCaan | 9 | 2008 | -4160.97 |
| CL9_575747901PSV_F0690714CCAM03008P1 | Dalbeattie | 1 | 2008 | -4160.97 |
| CL9_575747976PSV_F0690714CCAM03008P1 | Dalbeattie | 2 | 2008 | -4160.97 |
| CL9_575748042PSV_F0690714CCAM03008P1 | Dalbeattie | 3 | 2008 | -4160.97 |
| CL9_575748108PSV_F0690714CCAM03008P1 | Dalbeattie | 4 | 2008 | -4160.97 |
| CL9_575748304PSV_F0690714CCAM03008P1 | Dalbeattie | 5 | 2008 | -4160.97 |
| CL9_576018665PSV_F0691072CCAM03011P1 | Morven | 2 | 2011 | -4157.05 |
| CL9_576018731PSV_F0691072CCAM03011P1 | Morven | 3 | 2011 | -4157.05 |
| CL9_576019034PSV_F0691072CCAM03011P1 | Morven | 5 | 2011 | -4157.05 |
| CL9_576019242PSV_F0691072CCAM03011P1 | Morven | 8 | 2011 | -4157.05 |
| CL9_576019308PSV_F0691072CCAM03011P1 | Morven | 9 | 2011 | -4157.05 |
| CL9_576019916PSV_F0691072CCAM04011P1 | Insch | 1 | 2011 | -4157.05 |
| CL9_576019992PSV_F0691072CCAM04011P1 | Insch | 2 | 2011 | -4157.05 |
| CL9_576020057PSV_F0691072CCAM04011P1 | Insch | 3 | 2011 | -4157.05 |
| CL9_576020122PSV_F0691072CCAM04011P1 | Insch | 4 | 2011 | -4157.05 |
| CL9_576020336PSV_F0691072CCAM04011P1 | Insch | 5 | 2011 | -4157.05 |
| CL9_576020411PSV_F0691072CCAM04011P1 | Insch | 6 | 2011 | -4157.05 |
| CL9_576020476PSV_F0691072CCAM04011P1 | Insch | 7 | 2011 | -4157.05 |
| CL9_576020540PSV_F0691072CCAM04011P1 | Insch | 8 | 2011 | -4157.05 |
| CL9_576020604PSV_F0691072CCAM04011P1 | Insch | 9 | 2011 | -4157.05 |
| CL9_576020754PSV_F0691072CCAM04011P1 | Insch | 10 | 2011 | -4157.05 |
| CL9_576021052PSV_F0691072CCAM05011P1 | Pabay | 1 | 2011 | -4157.05 |
| CL9_576021120PSV_F0691072CCAM05011P1 | Pabay | 2 | 2011 | -4157.05 |
| CL9_576021186PSV_F0691072CCAM05011P1 | Pabay | 3 | 2011 | -4157.05 |
| CL9_576021251PSV_F0691072CCAM05011P1 | Pabay | 4 | 2011 | -4157.05 |
| CL9_576021443PSV_F0691072CCAM05011P1 | Pabay | 5 | 2011 | -4157.05 |
| CL9_576021576PSV_F0691072CCAM05011P1 | Pabay | 7 | 2011 | -4157.05 |
| CL9_576021641PSV_F0691072CCAM05011P1 | Pabay | 8 | 2011 | -4157.05 |
| CL9_576021706PSV_F0691072CCAM05011P1 | Pabay | 9 | 2011 | -4157.05 |
| CL9_576021858PSV_F0691072CCAM05011P1 | Pabay | 10 | 2011 | -4157.05 |
| CL9_576103368PSV_F0691072CCAM01012P1 | Colonsay | 7 | 2012 | -4157.05 |
| CL9_576191053PSV_F0691384CCAM01013P1 | Lingarabay | 1 | 2013 | -4155.39 |
| CL9_576191129PSV_F0691384CCAM01013P1 | Lingarabay | 2 | 2013 | -4155.39 |
| CL9_576191195PSV_F0691384CCAM01013P1 | Lingarabay | 3 | 2013 | -4155.39 |
| CL9_576191426PSV_F0691384CCAM01013P1 | Lingarabay | 4 | 2013 | -4155.39 |
| CL9_576191495PSV_F0691384CCAM01013P1 | Lingarabay | 5 | 2013 | -4155.39 |
| CL9_576191561PSV_F0691384CCAM01013P1 | Lingarabay | 6 | 2013 | -4155.39 |
| CL9_576191792PSV_F0691384CCAM01013P1 | Lingarabay | 7 | 2013 | -4155.39 |

| | | | | |
|---|---|---|---|---|
| CL9_576191859PSV_F0691384CCAM01013P1 | Lingarabay | 8 | 2013 | -4155.39 |
| CL9_576192055PSV_F0691384CCAM01013P1 | Lingarabay | 9 | 2013 | -4155.39 |
| CL9_576192430PSV_F0691384CCAM02013P1 | Kinloch | 1 | 2013 | -4155.39 |
| CL9_576192506PSV_F0691384CCAM02013P1 | Kinloch | 2 | 2013 | -4155.39 |
| CL9_577612210PSV_F0692456CCAM01029P1 | Ely | 1 | 2029 | -4165.89 |
| CL9_577612278PSV_F0692456CCAM01029P1 | Ely | 2 | 2029 | -4165.89 |
| CL9_577612342PSV_F0692456CCAM01029P1 | Ely | 3 | 2029 | -4165.89 |
| CL9_577612406PSV_F0692456CCAM01029P1 | Ely | 4 | 2029 | -4165.89 |
| CL9_577612641PSV_F0692456CCAM01029P1 | Ely | 5 | 2029 | -4165.89 |
| CL9_577612709PSV_F0692456CCAM01029P1 | Ely | 6 | 2029 | -4165.89 |
| CL9_577612773PSV_F0692456CCAM01029P1 | Ely | 7 | 2029 | -4165.89 |
| CL9_577612837PSV_F0692456CCAM01029P1 | Ely | 8 | 2029 | -4165.89 |
| CL9_577612901PSV_F0692456CCAM01029P1 | Ely | 9 | 2029 | -4165.89 |
| CL9_577613095PSV_F0692456CCAM01029P1 | Ely | 10 | 2029 | -4165.89 |
| CL9_577613412PSV_F0692456CCAM02029P1 | Babbitt | 1 | 2029 | -4165.89 |
| CL9_577613480PSV_F0692456CCAM02029P1 | Babbitt | 2 | 2029 | -4165.89 |
| CL9_577613544PSV_F0692456CCAM02029P1 | Babbitt | 3 | 2029 | -4165.89 |
| CL9_577613778PSV_F0692456CCAM02029P1 | Babbitt | 4 | 2029 | -4165.89 |
| CL9_577613843PSV_F0692456CCAM02029P1 | Babbitt | 5 | 2029 | -4165.89 |
| CL9_577613907PSV_F0692456CCAM02029P1 | Babbitt | 6 | 2029 | -4165.89 |
| CL9_577614141PSV_F0692456CCAM02029P1 | Babbitt | 7 | 2029 | -4165.89 |
| CL9_577614206PSV_F0692456CCAM02029P1 | Babbitt | 8 | 2029 | -4165.89 |
| CL9_577614400PSV_F0692456CCAM02029P1 | Babbitt | 9 | 2029 | -4165.89 |
| CL9_577614710PSV_F0692456CCAM03029P1 | Hibbing | 1 | 2029 | -4165.89 |
| CL9_577614778PSV_F0692456CCAM03029P1 | Hibbing | 2 | 2029 | -4165.89 |
| CL9_577614842PSV_F0692456CCAM03029P1 | Hibbing | 3 | 2029 | -4165.89 |
| CL9_577614906PSV_F0692456CCAM03029P1 | Hibbing | 4 | 2029 | -4165.89 |
| CL9_577615162PSV_F0692456CCAM03029P1 | Hibbing | 5 | 2029 | -4165.89 |
| CL9_577615294PSV_F0692456CCAM03029P1 | Hibbing | 7 | 2029 | -4165.89 |
| CL9_577615358PSV_F0692456CCAM03029P1 | Hibbing | 8 | 2029 | -4165.89 |
| CL9_577615422PSV_F0692456CCAM03029P1 | Hibbing | 9 | 2029 | -4165.89 |
| CL9_577615616PSV_F0692456CCAM03029P1 | Hibbing | 10 | 2029 | -4165.89 |
| CL9_577883305PSV_F0692594CCAM01032P1 | Pokegama | 1 | 2032 | -4166.28 |
| CL9_577883373PSV_F0692594CCAM01032P1 | Pokegama | 2 | 2032 | -4166.28 |
| CL9_577883437PSV_F0692594CCAM01032P1 | Pokegama | 3 | 2032 | -4166.28 |
| CL9_577883501PSV_F0692594CCAM01032P1 | Pokegama | 4 | 2032 | -4166.28 |
| CL9_577883565PSV_F0692594CCAM01032P1 | Pokegama | 5 | 2032 | -4166.28 |
| CL9_577883629PSV_F0692594CCAM01032P1 | Pokegama | 6 | 2032 | -4166.28 |
| CL9_577883693PSV_F0692594CCAM01032P1 | Pokegama | 7 | 2032 | -4166.28 |
| CL9_577883821PSV_F0692594CCAM01032P1 | Pokegama | 9 | 2032 | -4166.28 |
| CL9_577884015PSV_F0692594CCAM01032P1 | Pokegama | 10 | 2032 | -4166.28 |
| CL9_577884414PSV_F0692594CCAM02032P1 | Kenora | 2 | 2032 | -4166.28 |
| CL9_577884545PSV_F0692594CCAM02032P1 | Kenora | 4 | 2032 | -4166.28 |
| CL9_577884781PSV_F0692594CCAM02032P1 | Kenora | 5 | 2032 | -4166.28 |
| CL9_577884856PSV_F0692594CCAM02032P1 | Kenora | 6 | 2032 | -4166.28 |
| CL9_577884922PSV_F0692594CCAM02032P1 | Kenora | 7 | 2032 | -4166.28 |
| CL9_577884987PSV_F0692594CCAM02032P1 | Kenora | 8 | 2032 | -4166.28 |
| CL9_577885053PSV_F0692594CCAM02032P1 | Kenora | 9 | 2032 | -4166.28 |
| CL9_577885226PSV_F0692594CCAM02032P1 | Kenora | 10 | 2032 | -4166.28 |
| CL9_578053630PSV_F0692766CCAM01034P1 | Mesabi | 3 | 2034 | -4168.18 |
| CL9_578053696PSV_F0692766CCAM01034P1 | Mesabi | 4 | 2034 | -4168.18 |
| CL9_578053953PSV_F0692766CCAM01034P1 | Mesabi | 5 | 2034 | -4168.18 |
| CL9_578054029PSV_F0692766CCAM01034P1 | Mesabi | 6 | 2034 | -4168.18 |
| CL9_578054095PSV_F0692766CCAM01034P1 | Mesabi | 7 | 2034 | -4168.18 |
| CL9_578054161PSV_F0692766CCAM01034P1 | Mesabi | 8 | 2034 | -4168.18 |
| CL9_578054423PSV_F0692766CCAM01034P1 | Mesabi | 10 | 2034 | -4168.18 |
| CL9_578054792PSV_F0692766CCAM02034P1 | WakemupBay | 1 | 2034 | -4168.18 |
| CL9_578054867PSV_F0692766CCAM02034P1 | WakemupBay | 2 | 2034 | -4168.18 |
| CL9_578054932PSV_F0692766CCAM02034P1 | WakemupBay | 3 | 2034 | -4168.18 |

| | | | | |
|---|---|---|---|---|
| CL9_578054997PSV_F0692766CCAM02034P1 | WakemupBay | 4 | 2034 | -4168.18 |
| CL9_578055234PSV_F0692766CCAM02034P1 | WakemupBay | 5 | 2034 | -4168.18 |
| CL9_578055309PSV_F0692766CCAM02034P1 | WakemupBay | 6 | 2034 | -4168.18 |
| CL9_578055374PSV_F0692766CCAM02034P1 | WakemupBay | 7 | 2034 | -4168.18 |
| CL9_578055439PSV_F0692766CCAM02034P1 | WakemupBay | 8 | 2034 | -4168.18 |
| CL9_578055504PSV_F0692766CCAM02034P1 | WakemupBay | 9 | 2034 | -4168.18 |
| CL9_578055699PSV_F0692766CCAM02034P1 | WakemupBay | 10 | 2034 | -4168.18 |
| CL9_578056113PSV_F0692766CCAM03034P1 | Midway | 2 | 2034 | -4168.18 |
| CL9_578056179PSV_F0692766CCAM03034P1 | Midway | 3 | 2034 | -4168.18 |
| CL9_578056245PSV_F0692766CCAM03034P1 | Midway | 4 | 2034 | -4168.18 |
| CL9_578056440PSV_F0692766CCAM03034P1 | Midway | 5 | 2034 | -4168.18 |
| CL9_578236556PSV_F0700000CCAM01036P1 | Virginia | 3 | 2036 | -4174.72 |
| CL9_578236620PSV_F0700000CCAM01036P1 | Virginia | 4 | 2036 | -4174.72 |
| CL9_578236854PSV_F0700000CCAM01036P1 | Virginia | 5 | 2036 | -4174.72 |
| CL9_578236922PSV_F0700000CCAM01036P1 | Virginia | 6 | 2036 | -4174.72 |
| CL9_578236986PSV_F0700000CCAM01036P1 | Virginia | 7 | 2036 | -4174.72 |
| CL9_578237114PSV_F0700000CCAM01036P1 | Virginia | 9 | 2036 | -4174.72 |
| CL9_578237308PSV_F0700000CCAM01036P1 | Virginia | 10 | 2036 | -4174.72 |
| CL9_578237659PSV_F0700000CCAM02036P1 | ShannonLake | 1 | 2036 | -4174.72 |
| CL9_578237727PSV_F0700000CCAM02036P1 | ShannonLake | 2 | 2036 | -4174.72 |
| CL9_578237791PSV_F0700000CCAM02036P1 | ShannonLake | 3 | 2036 | -4174.72 |
| CL9_578237855PSV_F0700000CCAM02036P1 | ShannonLake | 4 | 2036 | -4174.72 |
| CL9_578238090PSV_F0700000CCAM02036P1 | ShannonLake | 5 | 2036 | -4174.72 |
| CL9_578238158PSV_F0700000CCAM02036P1 | ShannonLake | 6 | 2036 | -4174.72 |
| CL9_578238222PSV_F0700000CCAM02036P1 | ShannonLake | 7 | 2036 | -4174.72 |
| CL9_578238285PSV_F0700000CCAM02036P1 | ShannonLake | 8 | 2036 | -4174.72 |
| CL9_578238348PSV_F0700000CCAM02036P1 | ShannonLake | 9 | 2036 | -4174.72 |
| CL9_578238542PSV_F0700000CCAM02036P1 | ShannonLake | 10 | 2036 | -4174.72 |
| CL9_578238853PSV_F0700000CCAM03036P1 | Eveleth | 1 | 2036 | -4174.72 |
| CL9_578238926PSV_F0700000CCAM03036P1 | Eveleth | 2 | 2036 | -4174.72 |
| CL9_578238992PSV_F0700000CCAM03036P1 | Eveleth | 3 | 2036 | -4174.72 |
| CL9_578239229PSV_F0700000CCAM03036P1 | Eveleth | 4 | 2036 | -4174.72 |
| CL9_578239305PSV_F0700000CCAM03036P1 | Eveleth | 5 | 2036 | -4174.72 |
| CL9_578239750PSV_F0700000CCAM03036P1 | Eveleth | 9 | 2036 | -4174.72 |
| CL9_578413296PSV_F0700240CCAM01038P1 | PaulsenLake | 1 | 2038 | -4181.3 |
| CL9_578413371PSV_F0700240CCAM01038P1 | PaulsenLake | 2 | 2038 | -4181.3 |
| CL9_578413437PSV_F0700240CCAM01038P1 | PaulsenLake | 3 | 2038 | -4181.3 |
| CL9_578413685PSV_F0700240CCAM01038P1 | PaulsenLake | 4 | 2038 | -4181.3 |
| CL9_578413760PSV_F0700240CCAM01038P1 | PaulsenLake | 5 | 2038 | -4181.3 |
| CL9_578413826PSV_F0700240CCAM01038P1 | PaulsenLake | 6 | 2038 | -4181.3 |
| CL9_578414075PSV_F0700240CCAM01038P1 | PaulsenLake | 7 | 2038 | -4181.3 |
| CL9_578414150PSV_F0700240CCAM01038P1 | PaulsenLake | 8 | 2038 | -4181.3 |
| CL9_578414814PSV_F0700240CCAM02038P1 | Negaunee | 1 | 2038 | -4181.3 |
| CL9_578414882PSV_F0700240CCAM02038P1 | Negaunee | 2 | 2038 | -4181.3 |
| CL9_578414946PSV_F0700240CCAM02038P1 | Negaunee | 3 | 2038 | -4181.3 |
| CL9_578415010PSV_F0700240CCAM02038P1 | Negaunee | 4 | 2038 | -4181.3 |
| CL9_578415203PSV_F0700240CCAM02038P1 | Negaunee | 5 | 2038 | -4181.3 |
| CL9_578415520PSV_F0700240CCAM03038P1 | Nashwauk | 1 | 2038 | -4181.3 |
| CL9_578415595PSV_F0700240CCAM03038P1 | Nashwauk | 2 | 2038 | -4181.3 |
| CL9_578415662PSV_F0700240CCAM03038P1 | Nashwauk | 3 | 2038 | -4181.3 |
| CL9_578415866PSV_F0700240CCAM03038P1 | Nashwauk | 4 | 2038 | -4181.3 |
| CL9_578415932PSV_F0700240CCAM03038P1 | Nashwauk | 5 | 2038 | -4181.3 |
| CL9_578415998PSV_F0700240CCAM03038P1 | Nashwauk | 6 | 2038 | -4181.3 |
| CL9_578416204PSV_F0700240CCAM03038P1 | Nashwauk | 7 | 2038 | -4181.3 |
| CL9_578416270PSV_F0700240CCAM03038P1 | Nashwauk | 8 | 2038 | -4181.3 |
| CL9_578416445PSV_F0700240CCAM03038P1 | Nashwauk | 9 | 2038 | -4181.3 |
| CL9_578587974PSV_F0700552CCAM01040P1 | PrairieLake | 1 | 2040 | -4179.1 |
| CL9_578588049PSV_F0700552CCAM01040P1 | PrairieLake | 2 | 2040 | -4179.1 |
| CL9_578588115PSV_F0700552CCAM01040P1 | PrairieLake | 3 | 2040 | -4179.1 |

| | | | | |
|---|---|---|---|---|
| CL9_578588181PSV_F0700552CCAM01040P1 | PrairieLake | 4 | 2040 | -4179.1 |
| CL9_578588377PSV_F0700552CCAM01040P1 | PrairieLake | 5 | 2040 | -4179.1 |
| CL9_578588724PSV_F0700552CCAM02040P1 | Gowan | 1 | 2040 | -4179.1 |
| CL9_578588792PSV_F0700552CCAM02040P1 | Gowan | 2 | 2040 | -4179.1 |
| CL9_578588856PSV_F0700552CCAM02040P1 | Gowan | 3 | 2040 | -4179.1 |
| CL9_578588920PSV_F0700552CCAM02040P1 | Gowan | 4 | 2040 | -4179.1 |
| CL9_578588983PSV_F0700552CCAM02040P1 | Gowan | 5 | 2040 | -4179.1 |
| CL9_578589047PSV_F0700552CCAM02040P1 | Gowan | 6 | 2040 | -4179.1 |
| CL9_578589110PSV_F0700552CCAM02040P1 | Gowan | 7 | 2040 | -4179.1 |
| CL9_578589174PSV_F0700552CCAM02040P1 | Gowan | 8 | 2040 | -4179.1 |
| CL9_578589238PSV_F0700552CCAM02040P1 | Gowan | 9 | 2040 | -4179.1 |
| CL9_578589440PSV_F0700552CCAM02040P1 | Gowan | 10 | 2040 | -4179.1 |
| CL9_578676359PSV_F0700886CCAM01041P1 | BartoLake | 6 | 2041 | -4178.56 |
| CL9_578676616PSV_F0700886CCAM01041P1 | BartoLake | 7 | 2041 | -4178.56 |
| CL9_578676692PSV_F0700886CCAM01041P1 | BartoLake | 8 | 2041 | -4178.56 |
| CL9_578676758PSV_F0700886CCAM01041P1 | BartoLake | 9 | 2041 | -4178.56 |
| CL9_578676954PSV_F0700886CCAM01041P1 | BartoLake | 10 | 2041 | -4178.56 |
| CL9_578677311PSV_F0700886CCAM02041P1 | HomerLake | 1 | 2041 | -4178.56 |
| CL9_578677387PSV_F0700886CCAM02041P1 | HomerLake | 2 | 2041 | -4178.56 |
| CL9_578677453PSV_F0700886CCAM02041P1 | HomerLake | 3 | 2041 | -4178.56 |
| CL9_578677702PSV_F0700886CCAM02041P1 | HomerLake | 4 | 2041 | -4178.56 |
| CL9_578677778PSV_F0700886CCAM02041P1 | HomerLake | 5 | 2041 | -4178.56 |
| CL9_578677844PSV_F0700886CCAM02041P1 | HomerLake | 6 | 2041 | -4178.56 |
| CL9_578678094PSV_F0700886CCAM02041P1 | HomerLake | 7 | 2041 | -4178.56 |
| CL9_578678236PSV_F0700886CCAM02041P1 | HomerLake | 9 | 2041 | -4178.56 |
| CL9_578678409PSV_F0700886CCAM02041P1 | HomerLake | 10 | 2041 | -4178.56 |
| CL9_578763987PSV_F0701000CCAM01042P1 | PigeonRiver | 1 | 2042 | -4179.18 |
| CL9_578764063PSV_F0701000CCAM01042P1 | PigeonRiver | 2 | 2042 | -4179.18 |
| CL9_578764129PSV_F0701000CCAM01042P1 | PigeonRiver | 3 | 2042 | -4179.18 |
| CL9_578764368PSV_F0701000CCAM01042P1 | PigeonRiver | 4 | 2042 | -4179.18 |
| CL9_578764441PSV_F0701000CCAM01042P1 | PigeonRiver | 5 | 2042 | -4179.18 |
| CL9_578764747PSV_F0701000CCAM01042P1 | PigeonRiver | 7 | 2042 | -4179.18 |
| CL9_578764819PSV_F0701000CCAM01042P1 | PigeonRiver | 8 | 2042 | -4179.18 |
| CL9_578765023PSV_F0701000CCAM01042P1 | PigeonRiver | 9 | 2042 | -4179.18 |
| CL9_578765357PSV_F0701000CCAM02042P1 | BaldEagleRiver | 1 | 2042 | -4179.18 |
| CL9_578765433PSV_F0701000CCAM02042P1 | BaldEagleRiver | 2 | 2042 | -4179.18 |
| CL9_578765499PSV_F0701000CCAM02042P1 | BaldEagleRiver | 3 | 2042 | -4179.18 |
| CL9_578765738PSV_F0701000CCAM02042P1 | BaldEagleRiver | 4 | 2042 | -4179.18 |
| CL9_578765811PSV_F0701000CCAM02042P1 | BaldEagleRiver | 5 | 2042 | -4179.18 |
| CL9_578765877PSV_F0701000CCAM02042P1 | BaldEagleRiver | 6 | 2042 | -4179.18 |
| CL9_578766117PSV_F0701000CCAM02042P1 | BaldEagleRiver | 7 | 2042 | -4179.18 |
| CL9_578766394PSV_F0701000CCAM02042P1 | BaldEagleRiver | 9 | 2042 | -4179.18 |
| CL9_578766737PSV_F0701000CCAM03042P1 | Arnold | 1 | 2042 | -4179.18 |
| CL9_578766812PSV_F0701000CCAM03042P1 | Arnold | 2 | 2042 | -4179.18 |
| CL9_578766878PSV_F0701000CCAM03042P1 | Arnold | 3 | 2042 | -4179.18 |
| CL9_578766944PSV_F0701000CCAM03042P1 | Arnold | 4 | 2042 | -4179.18 |
| CL9_578767148PSV_F0701000CCAM03042P1 | Arnold | 5 | 2042 | -4179.18 |
| CL9_579032301PSV_F0701138CCAM02045P1 | Coleraine | 1 | 2045 | -4180.64 |
| CL9_579032377PSV_F0701138CCAM02045P1 | Coleraine | 2 | 2045 | -4180.64 |
| CL9_579032443PSV_F0701138CCAM02045P1 | Coleraine | 3 | 2045 | -4180.64 |
| CL9_579032509PSV_F0701138CCAM02045P1 | Coleraine | 4 | 2045 | -4180.64 |
| CL9_579032705PSV_F0701138CCAM02045P1 | Coleraine | 5 | 2045 | -4180.64 |
| CL9_579033064PSV_F0701138CCAM03045P1 | Bovey | 1 | 2045 | -4180.64 |
| CL9_579033139PSV_F0701138CCAM03045P1 | Bovey | 2 | 2045 | -4180.64 |
| CL9_579033205PSV_F0701138CCAM03045P1 | Bovey | 3 | 2045 | -4180.64 |
| CL9_579033270PSV_F0701138CCAM03045P1 | Bovey | 4 | 2045 | -4180.64 |
| CL9_579033474PSV_F0701138CCAM03045P1 | Bovey | 5 | 2045 | -4180.64 |
| CL9_579033807PSV_F0701138CCAM04045P1 | Soudan | 1 | 2045 | -4180.64 |
| CL9_579033883PSV_F0701138CCAM04045P1 | Soudan | 2 | 2045 | -4180.64 |

| | | | | |
|---|---|---|---|---|
| CL9_579033949PSV_F0701138CCAM04045P1 | Soudan | 3 | 2045 | -4180.64 |
| CL9_579034015PSV_F0701138CCAM04045P1 | Soudan | 4 | 2045 | -4180.64 |
| CL9_579034219PSV_F0701138CCAM04045P1 | Soudan | 5 | 2045 | -4180.64 |
| CL9_579122929PSV_F0701430CCAM01046P1 | GrandLake | 1 | 2046 | -4187.41 |
| CL9_579123005PSV_F0701430CCAM01046P1 | GrandLake | 2 | 2046 | -4187.41 |
| CL9_579123071PSV_F0701430CCAM01046P1 | GrandLake | 3 | 2046 | -4187.41 |
| CL9_579123137PSV_F0701430CCAM01046P1 | GrandLake | 4 | 2046 | -4187.41 |
| CL9_579123341PSV_F0701430CCAM01046P1 | GrandLake | 5 | 2046 | -4187.41 |
| CL9_579123668PSV_F0701430CCAM02046P1 | MudLake | 1 | 2046 | -4187.41 |
| CL9_579123743PSV_F0701430CCAM02046P1 | MudLake | 2 | 2046 | -4187.41 |
| CL9_579123808PSV_F0701430CCAM02046P1 | MudLake | 3 | 2046 | -4187.41 |
| CL9_579123874PSV_F0701430CCAM02046P1 | MudLake | 4 | 2046 | -4187.41 |
| CL9_579124068PSV_F0701430CCAM02046P1 | MudLake | 5 | 2046 | -4187.41 |
| CL9_579302455PSV_F0701538CCAM01048P1 | GiantsRange | 1 | 2048 | -4188.74 |
| CL9_579302530PSV_F0701538CCAM01048P1 | GiantsRange | 2 | 2048 | -4188.74 |
| CL9_579302596PSV_F0701538CCAM01048P1 | GiantsRange | 3 | 2048 | -4188.74 |
| CL9_579302694PSV_F0701538CCAM01048P1 | GiantsRange | 4 | 2048 | -4188.74 |
| CL9_579302898PSV_F0701538CCAM01048P1 | GiantsRange | 5 | 2048 | -4188.74 |
| CL9_579302973PSV_F0701538CCAM01048P1 | GiantsRange | 6 | 2048 | -4188.74 |
| CL9_579303074PSV_F0701538CCAM01048P1 | GiantsRange | 7 | 2048 | -4188.74 |
| CL9_579303140PSV_F0701538CCAM01048P1 | GiantsRange | 8 | 2048 | -4188.74 |
| CL9_579303336PSV_F0701538CCAM01048P1 | GiantsRange | 9 | 2048 | -4188.74 |
| CL9_579303663PSV_F0701538CCAM02048P1 | Vermillion | 1 | 2048 | -4188.74 |
| CL9_579303914PSV_F0701538CCAM02048P1 | Vermillion | 3 | 2048 | -4188.74 |
| CL9_579304509PSV_F0701538CCAM03048P1 | LacLaCroix | 1 | 2048 | -4188.74 |
| CL9_579304577PSV_F0701538CCAM03048P1 | LacLaCroix | 2 | 2048 | -4188.74 |
| CL9_579304641PSV_F0701538CCAM03048P1 | LacLaCroix | 3 | 2048 | -4188.74 |
| CL9_579304883PSV_F0701538CCAM03048P1 | LacLaCroix | 4 | 2048 | -4188.74 |
| CL9_579304951PSV_F0701538CCAM03048P1 | LacLaCroix | 5 | 2048 | -4188.74 |
| CL9_579305015PSV_F0701538CCAM03048P1 | LacLaCroix | 6 | 2048 | -4188.74 |
| CL9_579480038PSV_F0701538CCAM05049P1 | MountainIron | 1 | 2050 | -4188.74 |
| CL9_579480114PSV_F0701538CCAM05049P1 | MountainIron | 2 | 2050 | -4188.74 |
| CL9_579480403PSV_F0701538CCAM05049P1 | MountainIron | 4 | 2050 | -4188.74 |
| CL9_579480469PSV_F0701538CCAM05049P1 | MountainIron | 5 | 2050 | -4188.74 |
| CL9_579480535PSV_F0701538CCAM05049P1 | MountainIron | 6 | 2050 | -4188.74 |
| CL9_579480745PSV_F0701538CCAM05049P1 | MountainIron | 7 | 2050 | -4188.74 |
| CL9_579652030PSV_F0701554CCAM01052P1 | Brownell | 1 | 2052 | -4188.79 |
| CL9_579652099PSV_F0701554CCAM01052P1 | Brownell | 2 | 2052 | -4188.79 |
| CL9_579652162PSV_F0701554CCAM01052P1 | Brownell | 3 | 2052 | -4188.79 |
| CL9_579652226PSV_F0701554CCAM01052P1 | Brownell | 4 | 2052 | -4188.79 |
| CL9_579652290PSV_F0701554CCAM01052P1 | Brownell | 5 | 2052 | -4188.79 |
| CL9_579652354PSV_F0701554CCAM01052P1 | Brownell | 6 | 2052 | -4188.79 |
| CL9_579652418PSV_F0701554CCAM01052P1 | Brownell | 7 | 2052 | -4188.79 |
| CL9_579652480PSV_F0701554CCAM01052P1 | Brownell | 8 | 2052 | -4188.79 |
| CL9_579652543PSV_F0701554CCAM01052P1 | Brownell | 9 | 2052 | -4188.79 |
| CL9_579652737PSV_F0701554CCAM01052P1 | Brownell | 10 | 2052 | -4188.79 |
| CL9_579653102PSV_F0701554CCAM02052P1 | Mahtowa | 1 | 2052 | -4188.79 |
| CL9_579653170PSV_F0701554CCAM02052P1 | Mahtowa | 2 | 2052 | -4188.79 |
| CL9_579653234PSV_F0701554CCAM02052P1 | Mahtowa | 3 | 2052 | -4188.79 |
| CL9_579653621PSV_F0701554CCAM02052P1 | Mahtowa | 6 | 2052 | -4188.79 |
| CL9_579653685PSV_F0701554CCAM02052P1 | Mahtowa | 7 | 2052 | -4188.79 |
| CL9_579653749PSV_F0701554CCAM02052P1 | Mahtowa | 8 | 2052 | -4188.79 |
| CL9_579653813PSV_F0701554CCAM02052P1 | Mahtowa | 9 | 2052 | -4188.79 |
| CL9_579654019PSV_F0701554CCAM02052P1 | Mahtowa | 10 | 2052 | -4188.79 |
| CL9_579740817PSV_F0701668CCAM01053P1 | Duluth | 1 | 2053 | -4191.28 |
| CL9_579740892PSV_F0701668CCAM01053P1 | Duluth | 2 | 2053 | -4191.28 |
| CL9_579740958PSV_F0701668CCAM01053P1 | Duluth | 3 | 2053 | -4191.28 |
| CL9_579741195PSV_F0701668CCAM01053P1 | Duluth | 4 | 2053 | -4191.28 |
| CL9_579741267PSV_F0701668CCAM01053P1 | Duluth | 5 | 2053 | -4191.28 |

| | | | | |
|---|---|---|---|---|
| CL9_579741333PSV_F0701668CCAM01053P1 | Duluth | 6 | 2053 | -4191.28 |
| CL9_579741571PSV_F0701668CCAM01053P1 | Duluth | 7 | 2053 | -4191.28 |
| CL9_579741643PSV_F0701668CCAM01053P1 | Duluth | 8 | 2053 | -4191.28 |
| CL9_579741847PSV_F0701668CCAM01053P1 | Duluth | 9 | 2053 | -4191.28 |
| CL9_579742187PSV_F0701668CCAM02053P1 | PineMountain | 1 | 2053 | -4191.28 |
| CL9_579742262PSV_F0701668CCAM02053P1 | PineMountain | 2 | 2053 | -4191.28 |
| CL9_579742328PSV_F0701668CCAM02053P1 | PineMountain | 3 | 2053 | -4191.28 |
| CL9_579742693PSV_F0701668CCAM02053P1 | PineMountain | 6 | 2053 | -4191.28 |
| CL9_579742924PSV_F0701668CCAM02053P1 | PineMountain | 7 | 2053 | -4191.28 |
| CL9_580011256PSV_F0701752CCAM02056P1 | Aitkin | 1 | 2056 | -4192.46 |
| CL9_580011332PSV_F0701752CCAM02056P1 | Aitkin | 2 | 2056 | -4192.46 |
| CL9_580011398PSV_F0701752CCAM02056P1 | Aitkin | 3 | 2056 | -4192.46 |
| CL9_580011464PSV_F0701752CCAM02056P1 | Aitkin | 4 | 2056 | -4192.46 |
| CL9_580011985PSV_F0701752CCAM03056P1 | Chisholm | 1 | 2056 | -4192.46 |
| CL9_580012060PSV_F0701752CCAM03056P1 | Chisholm | 2 | 2056 | -4192.46 |
| CL9_580012126PSV_F0701752CCAM03056P1 | Chisholm | 3 | 2056 | -4192.46 |
| CL9_580012352PSV_F0701752CCAM03056P1 | Chisholm | 4 | 2056 | -4192.46 |
| CL9_580012427PSV_F0701752CCAM03056P1 | Chisholm | 5 | 2056 | -4192.46 |
| CL9_580012493PSV_F0701752CCAM03056P1 | Chisholm | 6 | 2056 | -4192.46 |
| CL9_580012719PSV_F0701752CCAM03056P1 | Chisholm | 7 | 2056 | -4192.46 |
| CL9_580013042PSV_F0701752CCAM03056P1 | Chisholm | 10 | 2056 | -4192.46 |
| CL9_580013415PSV_F0701752CCAM04056P1 | Buhl | 1 | 2056 | -4192.46 |
| CL9_580013491PSV_F0701752CCAM04056P1 | Buhl | 2 | 2056 | -4192.46 |
| CL9_580013557PSV_F0701752CCAM04056P1 | Buhl | 3 | 2056 | -4192.46 |
| CL9_580013622PSV_F0701752CCAM04056P1 | Buhl | 4 | 2056 | -4192.46 |
| CL9_580013849PSV_F0701752CCAM04056P1 | Buhl | 5 | 2056 | -4192.46 |
| CL9_580013924PSV_F0701752CCAM04056P1 | Buhl | 6 | 2056 | -4192.46 |
| CL9_580013989PSV_F0701752CCAM04056P1 | Buhl | 7 | 2056 | -4192.46 |
| CL9_580014054PSV_F0701752CCAM04056P1 | Buhl | 8 | 2056 | -4192.46 |
| CL9_580014120PSV_F0701752CCAM04056P1 | Buhl | 9 | 2056 | -4192.46 |
| CL9_580014302PSV_F0701752CCAM04056P1 | Buhl | 10 | 2056 | -4192.46 |
| CL9_580452813PSV_F0701752CCAM02061P1 | GrandMarais | 1 | 2061 | -4192.46 |
| CL9_580452889PSV_F0701752CCAM02061P1 | GrandMarais | 2 | 2061 | -4192.46 |
| CL9_580452955PSV_F0701752CCAM02061P1 | GrandMarais | 3 | 2061 | -4192.46 |
| CL9_580453208PSV_F0701752CCAM02061P1 | GrandMarais | 4 | 2061 | -4192.46 |
| CL9_580453284PSV_F0701752CCAM02061P1 | GrandMarais | 5 | 2061 | -4192.46 |
| CL9_580453350PSV_F0701752CCAM02061P1 | GrandMarais | 6 | 2061 | -4192.46 |
| CL9_580453608PSV_F0701752CCAM02061P1 | GrandMarais | 7 | 2061 | -4192.46 |
| CL9_580453684PSV_F0701752CCAM02061P1 | GrandMarais | 8 | 2061 | -4192.46 |
| CL9_580453750PSV_F0701752CCAM02061P1 | GrandMarais | 9 | 2061 | -4192.46 |
| CL9_580454280PSV_F0701752CCAM03061P1 | Prosit | 1 | 2061 | -4192.46 |
| CL9_580454348PSV_F0701752CCAM03061P1 | Prosit | 2 | 2061 | -4192.46 |
| CL9_580454412PSV_F0701752CCAM03061P1 | Prosit | 3 | 2061 | -4192.46 |
| CL9_580454476PSV_F0701752CCAM03061P1 | Prosit | 4 | 2061 | -4192.46 |
| CL9_580454539PSV_F0701752CCAM03061P1 | Prosit | 5 | 2061 | -4192.46 |
| CL9_580454602PSV_F0701752CCAM03061P1 | Prosit | 6 | 2061 | -4192.46 |
| CL9_580454665PSV_F0701752CCAM03061P1 | Prosit | 7 | 2061 | -4192.46 |
| CL9_580454729PSV_F0701752CCAM03061P1 | Prosit | 8 | 2061 | -4192.46 |
| CL9_580454793PSV_F0701752CCAM03061P1 | Prosit | 9 | 2061 | -4192.46 |
| CL9_580454977PSV_F0701752CCAM03061P1 | Prosit | 10 | 2061 | -4192.46 |
| CL9_580629410PSV_F0701752CCAM01063P1 | BruleMountain | 1 | 2063 | -4192.46 |
| CL9_580629477PSV_F0701752CCAM01063P1 | BruleMountain | 2 | 2063 | -4192.46 |
| CL9_580629541PSV_F0701752CCAM01063P1 | BruleMountain | 3 | 2063 | -4192.46 |
| CL9_580629645PSV_F0701752CCAM01063P1 | BruleMountain | 4 | 2063 | -4192.46 |
| CL9_580629847PSV_F0701752CCAM01063P1 | BruleMountain | 5 | 2063 | -4192.46 |
| CL9_580629912PSV_F0701752CCAM01063P1 | BruleMountain | 6 | 2063 | -4192.46 |
| CL9_580630016PSV_F0701752CCAM01063P1 | BruleMountain | 7 | 2063 | -4192.46 |
| CL9_580630079PSV_F0701752CCAM01063P1 | BruleMountain | 8 | 2063 | -4192.46 |
| CL9_580630281PSV_F0701752CCAM01063P1 | BruleMountain | 9 | 2063 | -4192.46 |

| | | | | |
|---|---|---|---|---|
| CL9_580630647PSV_F0701752CCAM02063P1 | DevilTrack | 1 | 2063 | -4192.46 |
| CL9_580630723PSV_F0701752CCAM02063P1 | DevilTrack | 2 | 2063 | -4192.46 |
| CL9_580630789PSV_F0701752CCAM02063P1 | DevilTrack | 3 | 2063 | -4192.46 |
| CL9_580630855PSV_F0701752CCAM02063P1 | DevilTrack | 4 | 2063 | -4192.46 |
| CL9_580631063PSV_F0701752CCAM02063P1 | BruleMountain | 10 | 2063 | -4192.46 |
| CL9_580631408PSV_F0701752CCAM03063P1 | DevilfishTower | 1 | 2063 | -4192.46 |
| CL9_580631484PSV_F0701752CCAM03063P1 | DevilfishTower | 2 | 2063 | -4192.46 |
| CL9_580631550PSV_F0701752CCAM03063P1 | DevilfishTower | 3 | 2063 | -4192.46 |
| CL9_580631616PSV_F0701752CCAM03063P1 | DevilfishTower | 4 | 2063 | -4192.46 |
| CL9_580631825PSV_F0701752CCAM03063P1 | DevilfishTower | 5 | 2063 | -4192.46 |
| CL9_580994371PSV_F0701752CCAM01067P1 | SawtoothBluff | 1 | 2067 | -4192.46 |
| CL9_580994443PSV_F0701752CCAM01067P1 | SawtoothBluff | 2 | 2067 | -4192.46 |
| CL9_580994508PSV_F0701752CCAM01067P1 | SawtoothBluff | 3 | 2067 | -4192.46 |
| CL9_580994574PSV_F0701752CCAM01067P1 | SawtoothBluff | 4 | 2067 | -4192.46 |
| CL9_580994747PSV_F0701752CCAM01067P1 | SawtoothBluff | 5 | 2067 | -4192.46 |
| CL9_580995058PSV_F0701752CCAM02067P1 | Gary | 1 | 2067 | -4192.46 |
| CL9_580995133PSV_F0701752CCAM02067P1 | Gary | 2 | 2067 | -4192.46 |
| CL9_580995198PSV_F0701752CCAM02067P1 | Gary | 3 | 2067 | -4192.46 |
| CL9_580995263PSV_F0701752CCAM02067P1 | Gary | 4 | 2067 | -4192.46 |
| CL9_580995477PSV_F0701752CCAM02067P1 | Gary | 5 | 2067 | -4192.46 |
| CL9_580995552PSV_F0701752CCAM02067P1 | Gary | 6 | 2067 | -4192.46 |
| CL9_580995617PSV_F0701752CCAM02067P1 | Gary | 7 | 2067 | -4192.46 |
| CL9_580995682PSV_F0701752CCAM02067P1 | Gary | 8 | 2067 | -4192.46 |
| CL9_580995747PSV_F0701752CCAM02067P1 | Gary | 9 | 2067 | -4192.46 |
| CL9_580995921PSV_F0701752CCAM02067P1 | Gary | 10 | 2067 | -4192.46 |
| CL9_581070367PSV_F0701752CCAM01068P1 | Bassett | 1 | 2068 | -4192.46 |
| CL9_581070442PSV_F0701752CCAM01068P1 | Bassett | 2 | 2068 | -4192.46 |
| CL9_581070507PSV_F0701752CCAM01068P1 | Bassett | 3 | 2068 | -4192.46 |
| CL9_581070573PSV_F0701752CCAM01068P1 | Bassett | 4 | 2068 | -4192.46 |
| CL9_581070822PSV_F0701752CCAM01068P1 | Bassett | 5 | 2068 | -4192.46 |
| CL9_581070897PSV_F0701752CCAM01068P1 | Bassett | 6 | 2068 | -4192.46 |
| CL9_581070963PSV_F0701752CCAM01068P1 | Bassett | 7 | 2068 | -4192.46 |
| CL9_581071029PSV_F0701752CCAM01068P1 | Bassett | 8 | 2068 | -4192.46 |
| CL9_581071095PSV_F0701752CCAM01068P1 | Bassett | 9 | 2068 | -4192.46 |
| CL9_581071299PSV_F0701752CCAM01068P1 | Bassett | 10 | 2068 | -4192.46 |
| CL9_581261039PSV_F0701752CCAM01070P1 | LittleMarais | 1 | 2070 | -4192.46 |
| CL9_581261168PSV_F0701752CCAM01070P1 | LittleMarais | 3 | 2070 | -4192.46 |
| CL9_581261232PSV_F0701752CCAM01070P1 | LittleMarais | 4 | 2070 | -4192.46 |
| CL9_581261403PSV_F0701752CCAM01070P1 | LittleMarais | 5 | 2070 | -4192.46 |
| CL9_581437720PSV_F0701752CCAM01072P1 | Barrett2 | 1 | 2070 | -4192.46 |
| CL9_581437796PSV_F0701752CCAM01072P1 | Barrett2 | 2 | 2070 | -4192.46 |
| CL9_581437862PSV_F0701752CCAM01072P1 | Barrett2 | 3 | 2070 | -4192.46 |
| CL9_581437928PSV_F0701752CCAM01072P1 | Barrett2 | 4 | 2072 | -4192.46 |
| CL9_581438103PSV_F0701752CCAM01072P1 | Barrett2 | 5 | 2072 | -4192.46 |
| CL9_581618700PSV_F0701752CCAM02074P1 | Independence | 1 | 2072 | -4192.46 |
| CL9_581618772PSV_F0701752CCAM02074P1 | Independence | 2 | 2072 | -4192.46 |
| CL9_581618838PSV_F0701752CCAM02074P1 | Independence | 3 | 2072 | -4192.46 |
| CL9_581618904PSV_F0701752CCAM02074P1 | Independence | 4 | 2074 | -4192.46 |
| CL9_581619079PSV_F0701752CCAM02074P1 | Independence | 5 | 2074 | -4192.46 |
| CL9_594216473PSV_F0730000CCAM01216P1 | Grange | 1 | 2216 | -4150.11 |
| CL9_594216541PSV_F0730000CCAM01216P1 | Grange | 2 | 2216 | -4150.11 |
| CL9_594308747PSV_F0730000CCAM01217P1 | Clune | 1 | 2216 | -4150.11 |
| CL9_594308815PSV_F0730000CCAM01217P1 | Clune | 2 | 2217 | -4150.11 |
| CL9_594308879PSV_F0730000CCAM01217P1 | Clune | 3 | 2217 | -4150.11 |
| CL9_594309114PSV_F0730000CCAM01217P1 | Clune | 4 | 2217 | -4150.11 |
| CL9_594309182PSV_F0730000CCAM01217P1 | Clune | 5 | 2217 | -4150.11 |
| CL9_594309246PSV_F0730000CCAM01217P1 | Clune | 6 | 2217 | -4150.11 |
| CL9_594309549PSV_F0730000CCAM01217P1 | Clune | 8 | 2217 | -4150.11 |
| CL9_594309613PSV_F0730000CCAM01217P1 | Clune | 9 | 2217 | -4150.11 |

| | | | | |
|---|---|---|---|---|
| CL9_594309807PSV_F0730000CCAM01217P1 | Clune | 10 | 2217 | -4150.11 |
| CL9_594397488PSV_F0730000CCAM02218P1 | Grange | 1 | 2217 | -4150.11 |
| CL9_594396273PSV_F0730000CCAM01218P1 | MiltonNess | 1 | 2218 | -4150.11 |
| CL9_594396341PSV_F0730000CCAM01218P1 | MiltonNess | 2 | 2218 | -4150.11 |
| CL9_594396751PSV_F0730000CCAM01218P1 | MiltonNess | 6 | 2218 | -4150.11 |
| CL9_594396814PSV_F0730000CCAM01218P1 | MiltonNess | 7 | 2218 | -4150.11 |
| CL9_594396877PSV_F0730000CCAM01218P1 | MiltonNess | 8 | 2218 | -4150.11 |
| CL9_594396940PSV_F0730000CCAM01218P1 | MiltonNess | 9 | 2218 | -4150.11 |
| CL9_594397147PSV_F0730000CCAM01218P1 | MiltonNess | 10 | 2218 | -4150.11 |
| CL9_594397556PSV_F0730000CCAM02218P1 | Grange | 2 | 2218 | -4150.11 |
| CL9_594481775PSV_F0730046CCAM01219P1 | Dryden | 1 | 2218 | -4150.72 |
| CL9_594481915PSV_F0730046CCAM01219P1 | Dryden | 3 | 2219 | -4150.72 |
| CL9_594481980PSV_F0730046CCAM01219P1 | Dryden | 4 | 2219 | -4150.72 |
| CL9_594482224PSV_F0730046CCAM01219P1 | Dryden | 5 | 2219 | -4150.72 |
| CL9_594482364PSV_F0730046CCAM01219P1 | Dryden | 7 | 2219 | -4150.72 |
| CL9_594482429PSV_F0730046CCAM01219P1 | Dryden | 8 | 2219 | -4150.72 |
| CL9_594482495PSV_F0730046CCAM01219P1 | Dryden | 9 | 2219 | -4150.72 |
| CL9_594482691PSV_F0730046CCAM01219P1 | Dryden | 10 | 2219 | -4150.72 |
| CL9_594483008PSV_F0730046CCAM02219P1 | Kirkness | 1 | 2219 | -4150.72 |
| CL9_594483083PSV_F0730046CCAM02219P1 | Kirkness | 2 | 2219 | -4150.72 |
| CL9_594483148PSV_F0730046CCAM02219P1 | Kirkness | 3 | 2219 | -4150.72 |
| CL9_594483213PSV_F0730046CCAM02219P1 | Kirkness | 4 | 2219 | -4150.72 |
| CL9_594483450PSV_F0730046CCAM02219P1 | Kirkness | 5 | 2219 | -4150.72 |
| CL9_594483525PSV_F0730046CCAM02219P1 | Kirkness | 6 | 2219 | -4150.72 |
| CL9_594483590PSV_F0730046CCAM02219P1 | Kirkness | 7 | 2219 | -4150.72 |
| CL9_594483655PSV_F0730046CCAM02219P1 | Kirkness | 8 | 2219 | -4150.72 |
| CL9_594483720PSV_F0730046CCAM02219P1 | Kirkness | 9 | 2219 | -4150.72 |
| CL9_594483915PSV_F0730046CCAM02219P1 | Kirkness | 10 | 2219 | -4150.72 |
| CL9_594745930PSV_F0730448CCAM01222P1 | Flotta | 1 | 2219 | -4146.67 |
| CL9_594746005PSV_F0730448CCAM01222P1 | Flotta | 2 | 2222 | -4146.67 |
| CL9_594746071PSV_F0730448CCAM01222P1 | Flotta | 3 | 2222 | -4146.67 |
| CL9_594746135PSV_F0730448CCAM01222P1 | Flotta | 4 | 2222 | -4146.67 |
| CL9_594746202PSV_F0730448CCAM01222P1 | Flotta | 5 | 2222 | -4146.67 |
| CL9_594746577PSV_F0730448CCAM01222P1 | Flotta | 8 | 2222 | -4146.67 |
| CL9_594746643PSV_F0730448CCAM01222P1 | Flotta | 9 | 2222 | -4146.67 |
| CL9_594746840PSV_F0730448CCAM01222P1 | Flotta | 10 | 2222 | -4146.67 |
| CL9_595191774PSV_F0730550CCAM01226P1 | Rosebrae | 1 | 2226 | -4146.95 |
| CL9_595191842PSV_F0730550CCAM01226P1 | Rosebrae | 2 | 2226 | -4146.95 |
| CL9_595191906PSV_F0730550CCAM01226P1 | Rosebrae | 3 | 2226 | -4146.95 |
| CL9_595192292PSV_F0730550CCAM01226P1 | Rosebrae | 6 | 2226 | -4146.95 |
| CL9_595192356PSV_F0730550CCAM01226P1 | Rosebrae | 7 | 2226 | -4146.95 |
| CL9_595192420PSV_F0730550CCAM01226P1 | Rosebrae | 8 | 2226 | -4146.95 |
| CL9_595192484PSV_F0730550CCAM01226P1 | Rosebrae | 9 | 2226 | -4146.95 |
| CL9_595194686PSV_F0730550CCAM03226P1 | Cullen | 4 | 2226 | -4146.95 |
| CL9_595195010PSV_F0730550CCAM03226P1 | Cullen | 6 | 2226 | -4146.95 |
| CL9_595195073PSV_F0730550CCAM03226P1 | Cullen | 7 | 2226 | -4146.95 |
| CL9_595195408PSV_F0730550CCAM03226P1 | Cullen | 10 | 2226 | -4146.95 |
| CL9_595367865PSV_F0730550CCAM04229P1 | FlandersMoss | 1 | 2226 | -4146.95 |
| CL9_595365340PSV_F0730550CCAM01229P1 | FraserCastle | 1 | 2229 | -4146.95 |
| CL9_595365408PSV_F0730550CCAM01229P1 | FraserCastle | 2 | 2229 | -4146.95 |
| CL9_595365472PSV_F0730550CCAM01229P1 | FraserCastle | 3 | 2229 | -4146.95 |
| CL9_595365574PSV_F0730550CCAM01229P1 | FraserCastle | 4 | 2229 | -4146.95 |
| CL9_595365638PSV_F0730550CCAM01229P1 | FraserCastle | 5 | 2229 | -4146.95 |
| CL9_595365701PSV_F0730550CCAM01229P1 | FraserCastle | 6 | 2229 | -4146.95 |
| CL9_595365863PSV_F0730550CCAM01229P1 | FraserCastle | 8 | 2229 | -4146.95 |
| CL9_595366025PSV_F0730550CCAM01229P1 | FraserCastle | 9 | 2229 | -4146.95 |
| CL9_595367933PSV_F0730550CCAM04229P1 | FlandersMoss | 2 | 2229 | -4146.95 |
| CL9_595367997PSV_F0730550CCAM04229P1 | FlandersMoss | 3 | 2229 | -4146.95 |
| CL9_595368060PSV_F0730550CCAM04229P1 | FlandersMoss | 4 | 2229 | -4146.95 |

| | | | | |
|---|---|---|---|---|
| CL9_595368377PSV_F0730550CCAM04229P1 | FlandersMoss | 9 | 2229 | -4146.95 |
| CL9_595368583PSV_F0730550CCAM04229P1 | FlandersMoss | 10 | 2229 | -4146.95 |
| CL9_595636541PSV_F0730550CCAM02231P1 | FraserCastle2 | 1 | 2232 | -4146.95 |
| CL9_595636609PSV_F0730550CCAM02231P1 | FraserCastle2 | 2 | 2232 | -4146.95 |
| CL9_595636885PSV_F0730550CCAM02231P1 | FraserCastle2 | 4 | 2232 | -4146.95 |
| CL9_595636949PSV_F0730550CCAM02231P1 | FraserCastle2 | 5 | 2232 | -4146.95 |
| CL9_595637011PSV_F0730550CCAM02231P1 | FraserCastle2 | 6 | 2232 | -4146.95 |
| CL9_595637461PSV_F0730550CCAM02231P1 | FraserCastle2 | 9 | 2232 | -4146.95 |
| CL9_595637977PSV_F0730550CCAM03231P1 | BridgeofDon | 3 | 2232 | -4146.95 |
| CL9_595638041PSV_F0730550CCAM03231P1 | BridgeofDon | 4 | 2232 | -4146.95 |
| CL9_595724490PSV_F0730550CCAM01233P1 | DunCarloway | 2 | 2233 | -4146.95 |
| CL9_595724554PSV_F0730550CCAM01233P1 | DunCarloway | 3 | 2233 | -4146.95 |
| CL9_595724819PSV_F0730550CCAM01233P1 | DunCarloway | 5 | 2233 | -4146.95 |
| CL9_595724883PSV_F0730550CCAM01233P1 | DunCarloway | 6 | 2233 | -4146.95 |
| CL9_595725084PSV_F0730550CCAM01233P1 | DunCarloway | 7 | 2233 | -4146.95 |
| CL9_595725148PSV_F0730550CCAM01233P1 | DunCarloway | 8 | 2233 | -4146.95 |
| CL9_595725309PSV_F0730550CCAM01233P1 | DunCarloway | 9 | 2233 | -4146.95 |
| CL9_595725663PSV_F0730550CCAM02233P1 | St.AbbsHead | 1 | 2233 | -4146.95 |
| CL9_595725739PSV_F0730550CCAM02233P1 | St.AbbsHead | 2 | 2233 | -4146.95 |
| CL9_595725805PSV_F0730550CCAM02233P1 | St.AbbsHead | 3 | 2233 | -4146.95 |
| CL9_595726126PSV_F0730550CCAM02233P1 | St.AbbsHead | 5 | 2233 | -4146.95 |
| CL9_595726404PSV_F0730550CCAM02233P1 | St.AbbsHead | 7 | 2233 | -4146.95 |
| CL9_595726480PSV_F0730550CCAM02233P1 | St.AbbsHead | 8 | 2233 | -4146.95 |
| CL9_596614965PSV_F0730550CCAM01243P1 | Kingseat | 1 | 2242 | -4146.95 |
| CL9_596615033PSV_F0730550CCAM01243P1 | Kingseat | 2 | 2243 | -4146.95 |
| CL9_596615097PSV_F0730550CCAM01243P1 | Kingseat | 3 | 2243 | -4146.95 |
| CL9_596615161PSV_F0730550CCAM01243P1 | Kingseat | 4 | 2243 | -4146.95 |
| CL9_596615403PSV_F0730550CCAM01243P1 | Kingseat | 5 | 2243 | -4146.95 |
| CL9_596615471PSV_F0730550CCAM01243P1 | Kingseat | 6 | 2243 | -4146.95 |
| CL9_596615535PSV_F0730550CCAM01243P1 | Kingseat | 7 | 2243 | -4146.95 |
| CL9_596615599PSV_F0730550CCAM01243P1 | Kingseat | 8 | 2243 | -4146.95 |
| CL9_596615663PSV_F0730550CCAM01243P1 | Kingseat | 9 | 2243 | -4146.95 |
| CL9_596615865PSV_F0730550CCAM01243P1 | Kingseat | 10 | 2243 | -4146.95 |
| CL9_596702094PSV_F0730550CCAM03243P1 | Grampian | 1 | 2243 | -4146.95 |
| CL9_596702163PSV_F0730550CCAM03243P1 | Grampian | 2 | 2244 | -4146.95 |
| CL9_596702227PSV_F0730550CCAM03243P1 | Grampian | 3 | 2244 | -4146.95 |
| CL9_596702291PSV_F0730550CCAM03243P1 | Grampian | 4 | 2244 | -4146.95 |
| CL9_596702529PSV_F0730550CCAM03243P1 | Grampian | 5 | 2244 | -4146.95 |
| CL9_596702597PSV_F0730550CCAM03243P1 | Grampian | 6 | 2244 | -4146.95 |
| CL9_596702790PSV_F0730550CCAM03243P1 | Grampian | 7 | 2244 | -4146.95 |
| CL9_596702855PSV_F0730550CCAM03243P1 | Grampian | 8 | 2244 | -4146.95 |
| CL9_596702919PSV_F0730550CCAM03243P1 | Grampian | 9 | 2244 | -4146.95 |
| CL9_596703125PSV_F0730550CCAM03243P1 | Grampian | 10 | 2244 | -4146.95 |
| CL9_596882260PSV_F0730550CCAM05245P1 | Eildon | 1 | 2245 | -4146.95 |
| CL9_596881038PSV_F0730550CCAM04245P1 | Forres | 1 | 2246 | -4146.95 |
| CL9_596881106PSV_F0730550CCAM04245P1 | Forres | 2 | 2246 | -4146.95 |
| CL9_596881170PSV_F0730550CCAM04245P1 | Forres | 3 | 2246 | -4146.95 |
| CL9_596881234PSV_F0730550CCAM04245P1 | Forres | 4 | 2246 | -4146.95 |
| CL9_596881469PSV_F0730550CCAM04245P1 | Forres | 5 | 2246 | -4146.95 |
| CL9_596881537PSV_F0730550CCAM04245P1 | Forres | 6 | 2246 | -4146.95 |
| CL9_596881665PSV_F0730550CCAM04245P1 | Forres | 8 | 2246 | -4146.95 |
| CL9_596881729PSV_F0730550CCAM04245P1 | Forres | 9 | 2246 | -4146.95 |
| CL9_596882329PSV_F0730550CCAM05245P1 | Eildon | 2 | 2246 | -4146.95 |
| CL9_596882393PSV_F0730550CCAM05245P1 | Eildon | 3 | 2246 | -4146.95 |
| CL9_596882457PSV_F0730550CCAM05245P1 | Eildon | 4 | 2246 | -4146.95 |
| CL9_596882768PSV_F0730550CCAM05245P1 | Eildon | 6 | 2246 | -4146.95 |
| CL9_596882896PSV_F0730550CCAM05245P1 | Eildon | 8 | 2246 | -4146.95 |
| CL9_596882960PSV_F0730550CCAM05245P1 | Eildon | 9 | 2246 | -4146.95 |
| CL9_596883166PSV_F0730550CCAM05245P1 | Eildon | 10 | 2246 | -4146.95 |

| | | | | |
|---|---|---|---|---|
| CL9_596967595PSV_F0730550CCAM01247P1 | QuarryHaven | 1 | 2247 | -4146.95 |
| CL9_596967663PSV_F0730550CCAM01247P1 | QuarryHaven | 2 | 2247 | -4146.95 |
| CL9_596968169PSV_F0730550CCAM01247P1 | QuarryHaven | 7 | 2247 | -4146.95 |
| CL9_597411980PSV_F0730722CCAM03252P1 | ConanMains | 1 | 2250 | -4145.77 |
| CL9_597409746PSV_F0730722CCAM01252P1 | Knochan_Crag | 1 | 2252 | -4145.77 |
| CL9_597409821PSV_F0730722CCAM01252P1 | Knochan_Crag | 2 | 2252 | -4145.77 |
| CL9_597409886PSV_F0730722CCAM01252P1 | Knochan_Crag | 3 | 2252 | -4145.77 |
| CL9_597409951PSV_F0730722CCAM01252P1 | Knochan_Crag | 4 | 2252 | -4145.77 |
| CL9_597410016PSV_F0730722CCAM01252P1 | Knochan_Crag | 5 | 2252 | -4145.77 |
| CL9_597410081PSV_F0730722CCAM01252P1 | Knochan_Crag | 6 | 2252 | -4145.77 |
| CL9_597410146PSV_F0730722CCAM01252P1 | Knochan_Crag | 7 | 2252 | -4145.77 |
| CL9_597410211PSV_F0730722CCAM01252P1 | Knochan_Crag | 8 | 2252 | -4145.77 |
| CL9_597410276PSV_F0730722CCAM01252P1 | Knochan_Crag | 9 | 2252 | -4145.77 |
| CL9_597410439PSV_F0730722CCAM01252P1 | Knochan_Crag | 10 | 2252 | -4145.77 |
| CL9_597410783PSV_F0730722CCAM02252P1 | Skatie_Shore | 1 | 2252 | -4145.77 |
| CL9_597410858PSV_F0730722CCAM02252P1 | Skatie_Shore | 2 | 2252 | -4145.77 |
| CL9_597410923PSV_F0730722CCAM02252P1 | Skatie_Shore | 3 | 2252 | -4145.77 |
| CL9_597410989PSV_F0730722CCAM02252P1 | Skatie_Shore | 4 | 2252 | -4145.77 |
| CL9_597411054PSV_F0730722CCAM02252P1 | Skatie_Shore | 5 | 2252 | -4145.77 |
| CL9_597411290PSV_F0730722CCAM02252P1 | Skatie_Shore | 6 | 2252 | -4145.77 |
| CL9_597411365PSV_F0730722CCAM02252P1 | Skatie_Shore | 7 | 2252 | -4145.77 |
| CL9_597411430PSV_F0730722CCAM02252P1 | Skatie_Shore | 8 | 2252 | -4145.77 |
| CL9_597411495PSV_F0730722CCAM02252P1 | Skatie_Shore | 9 | 2252 | -4145.77 |
| CL9_597411659PSV_F0730722CCAM02252P1 | Skatie_Shore | 10 | 2252 | -4145.77 |
| CL9_597412055PSV_F0730722CCAM03252P1 | ConanMains | 2 | 2252 | -4145.77 |
| CL9_597412120PSV_F0730722CCAM03252P1 | ConanMains | 3 | 2252 | -4145.77 |
| CL9_597412185PSV_F0730722CCAM03252P1 | ConanMains | 4 | 2252 | -4145.77 |
| CL9_597412250PSV_F0730722CCAM03252P1 | ConanMains | 5 | 2252 | -4145.77 |
| CL9_597412487PSV_F0730722CCAM03252P1 | ConanMains | 6 | 2252 | -4145.77 |
| CL9_597412562PSV_F0730722CCAM03252P1 | ConanMains | 7 | 2252 | -4145.77 |
| CL9_597412627PSV_F0730722CCAM03252P1 | ConanMains | 8 | 2252 | -4145.77 |
| CL9_597412692PSV_F0730722CCAM03252P1 | ConanMains | 9 | 2252 | -4145.77 |
| CL9_597412887PSV_F0730722CCAM03252P1 | ConanMains | 10 | 2252 | -4145.77 |
| CL9_597587560PSV_F0730800CCAM03254P1 | BlackfortHill | 1 | 2252 | -4144.95 |
| CL9_597585484PSV_F0730800CCAM01254P1 | Woodhill | 1 | 2254 | -4144.95 |
| CL9_597585559PSV_F0730800CCAM01254P1 | Woodhill | 2 | 2254 | -4144.95 |
| CL9_597585625PSV_F0730800CCAM01254P1 | Woodhill | 3 | 2254 | -4144.95 |
| CL9_597585721PSV_F0730800CCAM01254P1 | Woodhill | 4 | 2254 | -4144.95 |
| CL9_597585786PSV_F0730800CCAM01254P1 | Woodhill | 5 | 2254 | -4144.95 |
| CL9_597585851PSV_F0730800CCAM01254P1 | Woodhill | 6 | 2254 | -4144.95 |
| CL9_597585948PSV_F0730800CCAM01254P1 | Woodhill | 7 | 2254 | -4144.95 |
| CL9_597586013PSV_F0730800CCAM01254P1 | Woodhill | 8 | 2254 | -4144.95 |
| CL9_597586209PSV_F0730800CCAM01254P1 | Woodhill | 9 | 2254 | -4144.95 |
| CL9_597586522PSV_F0730800CCAM02254P1 | Dalziel | 1 | 2254 | -4144.95 |
| CL9_597586597PSV_F0730800CCAM02254P1 | Dalziel | 2 | 2254 | -4144.95 |
| CL9_597586662PSV_F0730800CCAM02254P1 | Dalziel | 3 | 2254 | -4144.95 |
| CL9_597586727PSV_F0730800CCAM02254P1 | Dalziel | 4 | 2254 | -4144.95 |
| CL9_597586792PSV_F0730800CCAM02254P1 | Dalziel | 5 | 2254 | -4144.95 |
| CL9_597586857PSV_F0730800CCAM02254P1 | Dalziel | 6 | 2254 | -4144.95 |
| CL9_597586922PSV_F0730800CCAM02254P1 | Dalziel | 7 | 2254 | -4144.95 |
| CL9_597586987PSV_F0730800CCAM02254P1 | Dalziel | 8 | 2254 | -4144.95 |
| CL9_597587053PSV_F0730800CCAM02254P1 | Dalziel | 9 | 2254 | -4144.95 |
| CL9_597587249PSV_F0730800CCAM02254P1 | Dalziel | 10 | 2254 | -4144.95 |
| CL9_597587635PSV_F0730800CCAM03254P1 | BlackfortHill | 2 | 2254 | -4144.95 |
| CL9_597587961PSV_F0730800CCAM03254P1 | BlackfortHill | 5 | 2254 | -4144.95 |
| CL9_597768054PSV_F0731010CCAM01256P1 | Tarness_Haven | 1 | 2254 | -4144 |
| CL9_597768129PSV_F0731010CCAM01256P1 | Tarness_Haven | 2 | 2256 | -4144 |
| CL9_597768195PSV_F0731010CCAM01256P1 | Tarness_Haven | 3 | 2256 | -4144 |
| CL9_597768261PSV_F0731010CCAM01256P1 | Tarness_Haven | 4 | 2256 | -4144 |

| | | | | |
|---|---|---|---|---|
| CL9_597768457PSV_F0731010CCAM01256P1 | Tarness_Haven | 5 | 2256 | -4144 |
| CL9_597850454PSV_F0731104CCAM01257P1 | RockHall1 | 1 | 2256 | -4143.6 |
| CL9_597850518PSV_F0731104CCAM01257P1 | RockHall1 | 2 | 2257 | -4143.6 |
| CL9_597850582PSV_F0731104CCAM01257P1 | RockHall1 | 3 | 2257 | -4143.6 |
| CL9_597850646PSV_F0731104CCAM01257P1 | RockHall1 | 4 | 2257 | -4143.6 |
| CL9_597850808PSV_F0731104CCAM01257P1 | RockHall1 | 5 | 2257 | -4143.6 |
| CL9_597851118PSV_F0731104CCAM02257P1 | RockHall2 | 1 | 2257 | -4143.6 |
| CL9_597851186PSV_F0731104CCAM02257P1 | RockHall2 | 2 | 2257 | -4143.6 |
| CL9_597851250PSV_F0731104CCAM02257P1 | RockHall2 | 3 | 2257 | -4143.6 |
| CL9_597851314PSV_F0731104CCAM02257P1 | RockHall2 | 4 | 2257 | -4143.6 |
| CL9_597851508PSV_F0731104CCAM02257P1 | RockHall2 | 5 | 2257 | -4143.6 |
| CL9_597851821PSV_F0731104CCAM03257P1 | RockHall3 | 1 | 2257 | -4143.6 |
| CL9_597851889PSV_F0731104CCAM03257P1 | RockHall3 | 2 | 2257 | -4143.6 |
| CL9_597851953PSV_F0731104CCAM03257P1 | RockHall3 | 3 | 2257 | -4143.6 |
| CL9_597852017PSV_F0731104CCAM03257P1 | RockHall3 | 4 | 2257 | -4143.6 |
| CL9_597852211PSV_F0731104CCAM03257P1 | RockHall3 | 5 | 2257 | -4143.6 |
| CL9_598036404PSV_F0731206CCAM02258P1 | RockHall4 | 1 | 2259 | -4143.6 |
| CL9_598036478PSV_F0731206CCAM02258P1 | RockHall4 | 2 | 2259 | -4143.6 |
| CL9_598036542PSV_F0731206CCAM02258P1 | RockHall4 | 3 | 2259 | -4143.6 |
| CL9_598036777PSV_F0731206CCAM02258P1 | RockHall4 | 4 | 2259 | -4143.6 |
| CL9_598036845PSV_F0731206CCAM02258P1 | RockHall4 | 5 | 2259 | -4143.6 |
| CL9_598036909PSV_F0731206CCAM02258P1 | RockHall4 | 6 | 2259 | -4143.6 |
| CL9_598037144PSV_F0731206CCAM02258P1 | RockHall4 | 7 | 2259 | -4143.6 |
| CL9_598037212PSV_F0731206CCAM02258P1 | RockHall4 | 8 | 2259 | -4143.6 |
| CL9_598037276PSV_F0731206CCAM02258P1 | RockHall4 | 9 | 2259 | -4143.6 |
| CL9_598037469PSV_F0731206CCAM02258P1 | RockHall4 | 10 | 2259 | -4143.6 |
| CL9_598038583PSV_F0731206CCAM03258P1 | ClunyHill | 8 | 2259 | -4143.6 |
| CL9_598038647PSV_F0731206CCAM03258P1 | ClunyHill | 9 | 2259 | -4143.6 |
| CL9_598038841PSV_F0731206CCAM03258P1 | ClunyHill | 10 | 2259 | -4143.6 |
| CL9_598390311PSV_F0731206CCAM03263P1 | Arnage | 1 | 2259 | -4143.6 |
| CL9_598390379PSV_F0731206CCAM03263P1 | Arnage | 2 | 2263 | -4143.6 |
| CL9_598390443PSV_F0731206CCAM03263P1 | Arnage | 3 | 2263 | -4143.6 |
| CL9_598390507PSV_F0731206CCAM03263P1 | Arnage | 4 | 2263 | -4143.6 |
| CL9_598390571PSV_F0731206CCAM03263P1 | Arnage | 5 | 2263 | -4143.6 |
| CL9_598390806PSV_F0731206CCAM03263P1 | Arnage | 6 | 2263 | -4143.6 |
| CL9_598390874PSV_F0731206CCAM03263P1 | Arnage | 7 | 2263 | -4143.6 |
| CL9_598390938PSV_F0731206CCAM03263P1 | Arnage | 8 | 2263 | -4143.6 |
| CL9_598391002PSV_F0731206CCAM03263P1 | Arnage | 9 | 2263 | -4143.6 |
| CL9_598391196PSV_F0731206CCAM03263P1 | Arnage | 10 | 2263 | -4143.6 |
| CL9_599548611PSV_F0731206CCAM02276P1 | Auchenheath | 6 | 2276 | -4143.79 |
| CL9_599548674PSV_F0731206CCAM02276P1 | Auchenheath | 7 | 2276 | -4143.79 |
| CL9_599548737PSV_F0731206CCAM02276P1 | Auchenheath | 8 | 2276 | -4143.79 |
| CL9_599548801PSV_F0731206CCAM02276P1 | Auchenheath | 9 | 2276 | -4143.79 |
| CL9_599548995PSV_F0731206CCAM02276P1 | Auchenheath | 10 | 2276 | -4143.79 |
| CL9_599549309PSV_F0731206CCAM03276P1 | FirthofForth | 1 | 2276 | -4143.79 |
| CL9_599549374PSV_F0731206CCAM03276P1 | FirthofForth | 2 | 2276 | -4143.79 |
| CL9_599549438PSV_F0731206CCAM03276P1 | FirthofForth | 3 | 2276 | -4143.79 |
| CL9_599549502PSV_F0731206CCAM03276P1 | FirthofForth | 4 | 2276 | -4143.79 |
| CL9_599549737PSV_F0731206CCAM03276P1 | FirthofForth | 5 | 2276 | -4143.79 |
| CL9_599549805PSV_F0731206CCAM03276P1 | FirthofForth | 6 | 2276 | -4143.79 |
| CL9_599549869PSV_F0731206CCAM03276P1 | FirthofForth | 7 | 2276 | -4143.79 |
| CL9_599549933PSV_F0731206CCAM03276P1 | FirthofForth | 8 | 2276 | -4143.79 |
| CL9_599549997PSV_F0731206CCAM03276P1 | FirthofForth | 9 | 2276 | -4143.79 |
| CL9_599550190PSV_F0731206CCAM03276P1 | FirthofForth | 10 | 2276 | -4143.79 |
| CL9_599813360PSV_F0731206CCAM02279P1 | Portnockie | 1 | 2279 | -4143.79 |
| CL9_599813428PSV_F0731206CCAM02279P1 | Portnockie | 2 | 2279 | -4143.79 |
| CL9_599813492PSV_F0731206CCAM02279P1 | Portnockie | 3 | 2279 | -4143.79 |
| CL9_599813594PSV_F0731206CCAM02279P1 | Portnockie | 4 | 2279 | -4143.79 |
| CL9_599813658PSV_F0731206CCAM02279P1 | Portnockie | 5 | 2279 | -4143.79 |

| | | | | |
|---|---|---|---|---|
| CL9_599813722PSV_F0731206CCAM02279P1 | Portnockie | 6 | 2279 | -4143.79 |
| CL9_599813819PSV_F0731206CCAM02279P1 | Portnockie | 7 | 2279 | -4143.79 |
| CL9_599813882PSV_F0731206CCAM02279P1 | Portnockie | 8 | 2279 | -4143.79 |
| CL9_599814076PSV_F0731206CCAM02279P1 | Portnockie | 9 | 2279 | -4143.79 |
| CL9_599815102PSV_F0731206CCAM04279P1 | Bothwell | 1 | 2279 | -4143.79 |
| CL9_599815170PSV_F0731206CCAM04279P1 | Bothwell | 2 | 2279 | -4143.79 |
| CL9_599815234PSV_F0731206CCAM04279P1 | Bothwell | 3 | 2279 | -4143.79 |
| CL9_599815467PSV_F0731206CCAM04279P1 | Bothwell | 4 | 2279 | -4143.79 |
| CL9_599815532PSV_F0731206CCAM04279P1 | Bothwell | 5 | 2279 | -4143.79 |
| CL9_599815596PSV_F0731206CCAM04279P1 | Bothwell | 6 | 2279 | -4143.79 |
| CL9_599815829PSV_F0731206CCAM04279P1 | Bothwell | 7 | 2279 | -4143.79 |
| CL9_599815894PSV_F0731206CCAM04279P1 | Bothwell | 8 | 2279 | -4143.79 |
| CL9_599816088PSV_F0731206CCAM04279P1 | Bothwell | 9 | 2279 | -4143.79 |
| CL9_600157204PSV_F0731206CCAM02281P1 | StCyrus | 2 | 2283 | -4143.79 |
| CL9_600157268PSV_F0731206CCAM02281P1 | StCyrus | 3 | 2283 | -4143.79 |
| CL9_600157332PSV_F0731206CCAM02281P1 | StCyrus | 4 | 2283 | -4143.79 |
| CL9_600157566PSV_F0731206CCAM02281P1 | StCyrus | 5 | 2283 | -4143.79 |
| CL9_600157634PSV_F0731206CCAM02281P1 | StCyrus | 6 | 2283 | -4143.79 |
| CL9_600157698PSV_F0731206CCAM02281P1 | StCyrus | 7 | 2283 | -4143.79 |
| CL9_600157762PSV_F0731206CCAM02281P1 | StCyrus | 8 | 2283 | -4143.79 |
| CL9_600157826PSV_F0731206CCAM02281P1 | StCyrus | 9 | 2283 | -4143.79 |
| CL9_600261647PSV_F0731206CCAM01284P1 | Pitscurrie | 1 | 2284 | -4143.79 |
| CL9_600261715PSV_F0731206CCAM01284P1 | Pitscurrie | 2 | 2284 | -4143.79 |
| CL9_600261778PSV_F0731206CCAM01284P1 | Pitscurrie | 3 | 2284 | -4143.79 |
| CL9_600261841PSV_F0731206CCAM01284P1 | Pitscurrie | 4 | 2284 | -4143.79 |
| CL9_600261904PSV_F0731206CCAM01284P1 | Pitscurrie | 5 | 2284 | -4143.79 |
| CL9_600261967PSV_F0731206CCAM01284P1 | Pitscurrie | 6 | 2284 | -4143.79 |
| CL9_600262030PSV_F0731206CCAM01284P1 | Pitscurrie | 7 | 2284 | -4143.79 |
| CL9_600262094PSV_F0731206CCAM01284P1 | Pitscurrie | 8 | 2284 | -4143.79 |
| CL9_600262157PSV_F0731206CCAM01284P1 | Pitscurrie | 9 | 2284 | -4143.79 |
| CL9_600262340PSV_F0731206CCAM01284P1 | Pitscurrie | 10 | 2284 | -4143.79 |
| CL9_600262652PSV_F0731206CCAM02284P1 | Coatbridge | 1 | 2284 | -4143.79 |
| CL9_600262717PSV_F0731206CCAM02284P1 | Coatbridge | 2 | 2284 | -4143.79 |
| CL9_600262781PSV_F0731206CCAM02284P1 | Coatbridge | 3 | 2284 | -4143.79 |
| CL9_600262845PSV_F0731206CCAM02284P1 | Coatbridge | 4 | 2284 | -4143.79 |
| CL9_600263058PSV_F0731206CCAM02284P1 | Coatbridge | 5 | 2284 | -4143.79 |
| CL9_600263126PSV_F0731206CCAM02284P1 | Coatbridge | 6 | 2284 | -4143.79 |
| CL9_600263190PSV_F0731206CCAM02284P1 | Coatbridge | 7 | 2284 | -4143.79 |
| CL9_600263254PSV_F0731206CCAM02284P1 | Coatbridge | 8 | 2284 | -4143.79 |
| CL9_600263318PSV_F0731206CCAM02284P1 | Coatbridge | 9 | 2284 | -4143.79 |
| CL9_600263490PSV_F0731206CCAM02284P1 | Coatbridge | 10 | 2284 | -4143.79 |
| CL9_600523322PSV_F0731206CCAM02286P1 | Burra | 1 | 2287 | -4143.79 |
| CL9_600523390PSV_F0731206CCAM02286P1 | Burra | 2 | 2287 | -4143.79 |
| CL9_600523454PSV_F0731206CCAM02286P1 | Burra | 3 | 2287 | -4143.79 |
| CL9_600523518PSV_F0731206CCAM02286P1 | Burra | 4 | 2287 | -4143.79 |
| CL9_600523712PSV_F0731206CCAM02286P1 | Burra | 5 | 2287 | -4143.79 |
| CL9_600524098PSV_F0731206CCAM03286P1 | Braeriach | 2 | 2287 | -4143.79 |
| CL9_600524162PSV_F0731206CCAM03286P1 | Braeriach | 3 | 2287 | -4143.79 |
| CL9_600524397PSV_F0731206CCAM03286P1 | Braeriach | 4 | 2287 | -4143.79 |
| CL9_600524465PSV_F0731206CCAM03286P1 | Braeriach | 5 | 2287 | -4143.79 |
| CL9_600524529PSV_F0731206CCAM03286P1 | Braeriach | 6 | 2287 | -4143.79 |
| CL9_600524763PSV_F0731206CCAM03286P1 | Braeriach | 7 | 2287 | -4143.79 |
| CL9_600524831PSV_F0731206CCAM03286P1 | Braeriach | 8 | 2287 | -4143.79 |
| CL9_600524895PSV_F0731206CCAM03286P1 | Braeriach | 9 | 2287 | -4143.79 |
| CL9_600525089PSV_F0731206CCAM03286P1 | Braeriach | 10 | 2287 | -4143.79 |
| CL9_600525431PSV_F0731206CCAM04286P1 | Deveron | 1 | 2287 | -4143.79 |
| CL9_600525499PSV_F0731206CCAM04286P1 | Deveron | 2 | 2287 | -4143.79 |
| CL9_600525563PSV_F0731206CCAM04286P1 | Deveron | 3 | 2287 | -4143.79 |
| CL9_600525796PSV_F0731206CCAM04286P1 | Deveron | 4 | 2287 | -4143.79 |

| | | | | |
|---|---|---|---|---|
| CL9_600525861PSV_F0731206CCAM04286P1 | Deveron | 5 | 2287 | -4143.79 |
| CL9_600525925PSV_F0731206CCAM04286P1 | Deveron | 6 | 2287 | -4143.79 |
| CL9_600526160PSV_F0731206CCAM04286P1 | Deveron | 7 | 2287 | -4143.79 |
| CL9_600526225PSV_F0731206CCAM04286P1 | Deveron | 8 | 2287 | -4143.79 |
| CL9_600526429PSV_F0731206CCAM04286P1 | Deveron | 9 | 2287 | -4143.79 |
| CL9_600788130PSV_F0731206CCAM05288P1 | Dufftown | 1 | 2290 | -4143.79 |
| CL9_600788198PSV_F0731206CCAM05288P1 | Dufftown | 2 | 2290 | -4143.79 |
| CL9_600788262PSV_F0731206CCAM05288P1 | Dufftown | 3 | 2290 | -4143.79 |
| CL9_600788326PSV_F0731206CCAM05288P1 | Dufftown | 4 | 2290 | -4143.79 |
| CL9_600788560PSV_F0731206CCAM05288P1 | Dufftown | 5 | 2290 | -4143.79 |
| CL9_600788628PSV_F0731206CCAM05288P1 | Dufftown | 6 | 2290 | -4143.79 |
| CL9_600788692PSV_F0731206CCAM05288P1 | Dufftown | 7 | 2290 | -4143.79 |
| CL9_600788756PSV_F0731206CCAM05288P1 | Dufftown | 8 | 2290 | -4143.79 |
| CL9_600788820PSV_F0731206CCAM05288P1 | Dufftown | 9 | 2290 | -4143.79 |
| CL9_600789014PSV_F0731206CCAM05288P1 | Dufftown | 10 | 2290 | -4143.79 |
| CL9_600789337PSV_F0731206CCAM06288P1 | Lairg | 1 | 2290 | -4143.79 |
| CL9_600789405PSV_F0731206CCAM06288P1 | Lairg | 2 | 2290 | -4143.79 |
| CL9_600789469PSV_F0731206CCAM06288P1 | Lairg | 3 | 2290 | -4143.79 |
| CL9_600789533PSV_F0731206CCAM06288P1 | Lairg | 4 | 2290 | -4143.79 |
| CL9_600789768PSV_F0731206CCAM06288P1 | Lairg | 5 | 2290 | -4143.79 |
| CL9_600789842PSV_F0731206CCAM06288P1 | Lairg | 6 | 2290 | -4143.79 |
| CL9_600789906PSV_F0731206CCAM06288P1 | Lairg | 7 | 2290 | -4143.79 |
| CL9_600789970PSV_F0731206CCAM06288P1 | Lairg | 8 | 2290 | -4143.79 |
| CL9_600790034PSV_F0731206CCAM06288P1 | Lairg | 9 | 2290 | -4143.79 |
| CL9_600790227PSV_F0731206CCAM06288P1 | Lairg | 10 | 2290 | -4143.79 |
| CL9_600968272PSV_F0731206CCAM03292P1 | StCyrus_2 | 1 | 2292 | -4143.79 |
| CL9_600968340PSV_F0731206CCAM03292P1 | StCyrus_2 | 2 | 2292 | -4143.79 |
| CL9_600968404PSV_F0731206CCAM03292P1 | StCyrus_2 | 3 | 2292 | -4143.79 |
| CL9_600968506PSV_F0731206CCAM03292P1 | StCyrus_2 | 4 | 2292 | -4143.79 |
| CL9_600968570PSV_F0731206CCAM03292P1 | StCyrus_2 | 5 | 2292 | -4143.79 |
| CL9_600968634PSV_F0731206CCAM03292P1 | StCyrus_2 | 6 | 2292 | -4143.79 |
| CL9_600968731PSV_F0731206CCAM03292P1 | StCyrus_2 | 7 | 2292 | -4143.79 |
| CL9_600968795PSV_F0731206CCAM03292P1 | StCyrus_2 | 8 | 2292 | -4143.79 |
| CL9_600968988PSV_F0731206CCAM03292P1 | StCyrus_2 | 9 | 2292 | -4143.79 |
| CL9_601223778PSV_F0731206CCAM01295P1 | StCyrus3 | 1 | 2295 | -4143.79 |
| CL9_601223845PSV_F0731206CCAM01295P1 | StCyrus3 | 2 | 2295 | -4143.79 |
| CL9_601223909PSV_F0731206CCAM01295P1 | StCyrus3 | 3 | 2295 | -4143.79 |
| CL9_601223973PSV_F0731206CCAM01295P1 | StCyrus3 | 4 | 2295 | -4143.79 |
| CL9_601224076PSV_F0731206CCAM01295P1 | StCyrus3 | 5 | 2295 | -4143.79 |
| CL9_601224139PSV_F0731206CCAM01295P1 | StCyrus3 | 6 | 2295 | -4143.79 |
| CL9_601224203PSV_F0731206CCAM01295P1 | StCyrus3 | 7 | 2295 | -4143.79 |
| CL9_601224267PSV_F0731206CCAM01295P1 | StCyrus3 | 8 | 2295 | -4143.79 |
| CL9_601224499PSV_F0731206CCAM01295P1 | StCyrus3 | 9 | 2295 | -4143.79 |
| CL9_601224564PSV_F0731206CCAM01295P1 | StCyrus3 | 10 | 2295 | -4143.79 |
| CL9_601224628PSV_F0731206CCAM01295P1 | StCyrus3 | 11 | 2295 | -4143.79 |
| CL9_601224693PSV_F0731206CCAM01295P1 | StCyrus3 | 12 | 2295 | -4143.79 |
| CL9_601224796PSV_F0731206CCAM01295P1 | StCyrus3 | 13 | 2295 | -4143.79 |
| CL9_601224860PSV_F0731206CCAM01295P1 | StCyrus3 | 14 | 2295 | -4143.79 |
| CL9_601224924PSV_F0731206CCAM01295P1 | StCyrus3 | 15 | 2295 | -4143.79 |
| CL9_601225118PSV_F0731206CCAM01295P1 | StCyrus3 | 16 | 2295 | -4143.79 |
| CL9_601319419PSV_F0731206CCAM03295P1 | StNiniansTombolo | 1 | 2296 | -4143.79 |
| CL9_601319487PSV_F0731206CCAM03295P1 | StNiniansTombolo | 2 | 2296 | -4143.79 |
| CL9_601319551PSV_F0731206CCAM03295P1 | StNiniansTombolo | 3 | 2296 | -4143.79 |
| CL9_601319615PSV_F0731206CCAM03295P1 | StNiniansTombolo | 4 | 2296 | -4143.79 |
| CL9_601319679PSV_F0731206CCAM03295P1 | StNiniansTombolo | 5 | 2296 | -4143.79 |
| CL9_601319742PSV_F0731206CCAM03295P1 | StNiniansTombolo | 6 | 2296 | -4143.79 |
| CL9_601319806PSV_F0731206CCAM03295P1 | StNiniansTombolo | 7 | 2296 | -4143.79 |
| CL9_601319870PSV_F0731206CCAM03295P1 | StNiniansTombolo | 8 | 2296 | -4143.79 |
| CL9_601319934PSV_F0731206CCAM03295P1 | StNiniansTombolo | 9 | 2296 | -4143.79 |

| | | | | |
|---|---|---|---|---|
| CL9_601320128PSV_F0731206CCAM03295P1 | StNiniansTombolo | 10 | 2296 | -4143.79 |
| CL9_601320441PSV_F0731206CCAM04295P1 | StacPollaidh | 1 | 2296 | -4143.79 |
| CL9_601320509PSV_F0731206CCAM04295P1 | StacPollaidh | 2 | 2296 | -4143.79 |
| CL9_601320573PSV_F0731206CCAM04295P1 | StacPollaidh | 3 | 2296 | -4143.79 |
| CL9_601320637PSV_F0731206CCAM04295P1 | StacPollaidh | 4 | 2296 | -4143.79 |
| CL9_601320872PSV_F0731206CCAM04295P1 | StacPollaidh | 5 | 2296 | -4143.79 |
| CL9_601320940PSV_F0731206CCAM04295P1 | StacPollaidh | 6 | 2296 | -4143.79 |
| CL9_601321004PSV_F0731206CCAM04295P1 | StacPollaidh | 7 | 2296 | -4143.79 |
| CL9_601321068PSV_F0731206CCAM04295P1 | StacPollaidh | 8 | 2296 | -4143.79 |
| CL9_601321132PSV_F0731206CCAM04295P1 | StacPollaidh | 9 | 2296 | -4143.79 |
| CL9_601321326PSV_F0731206CCAM04295P1 | StacPollaidh | 10 | 2296 | -4143.79 |
| CL9_601495661PSV_F0731482CCAM01298P1 | Oldmeldrum | 1 | 2298 | -4142.77 |
| CL9_601495729PSV_F0731482CCAM01298P1 | Oldmeldrum | 2 | 2298 | -4142.77 |
| CL9_601495793PSV_F0731482CCAM01298P1 | Oldmeldrum | 3 | 2298 | -4142.77 |
| CL9_601495857PSV_F0731482CCAM01298P1 | Oldmeldrum | 4 | 2298 | -4142.77 |
| CL9_601496051PSV_F0731482CCAM01298P1 | Oldmeldrum | 5 | 2298 | -4142.77 |
| CL9_601496370PSV_F0731482CCAM02298P1 | Dyce | 1 | 2298 | -4142.77 |
| CL9_601496445PSV_F0731482CCAM02298P1 | Dyce | 2 | 2298 | -4142.77 |
| CL9_601496510PSV_F0731482CCAM02298P1 | Dyce | 3 | 2298 | -4142.77 |
| CL9_601496747PSV_F0731482CCAM02298P1 | Dyce | 4 | 2298 | -4142.77 |
| CL9_601496822PSV_F0731482CCAM02298P1 | Dyce | 5 | 2298 | -4142.77 |
| CL9_601496887PSV_F0731482CCAM02298P1 | Dyce | 6 | 2298 | -4142.77 |
| CL9_601497124PSV_F0731482CCAM02298P1 | Dyce | 7 | 2298 | -4142.77 |
| CL9_601497199PSV_F0731482CCAM02298P1 | Dyce | 8 | 2298 | -4142.77 |
| CL9_601497264PSV_F0731482CCAM02298P1 | Dyce | 9 | 2298 | -4142.77 |
| CL9_601497460PSV_F0731482CCAM02298P1 | Dyce | 10 | 2298 | -4142.77 |
| CL9_601497788PSV_F0731482CCAM03298P1 | Banchory | 1 | 2298 | -4142.77 |
| CL9_601497856PSV_F0731482CCAM03298P1 | Banchory | 2 | 2298 | -4142.77 |
| CL9_601497920PSV_F0731482CCAM03298P1 | Banchory | 3 | 2298 | -4142.77 |
| CL9_601498154PSV_F0731482CCAM03298P1 | Banchory | 4 | 2298 | -4142.77 |
| CL9_601498222PSV_F0731482CCAM03298P1 | Banchory | 5 | 2298 | -4142.77 |
| CL9_601498286PSV_F0731482CCAM03298P1 | Banchory | 6 | 2298 | -4142.77 |
| CL9_601498520PSV_F0731482CCAM03298P1 | Banchory | 7 | 2298 | -4142.77 |
| CL9_601498588PSV_F0731482CCAM03298P1 | Banchory | 8 | 2298 | -4142.77 |
| CL9_601498652PSV_F0731482CCAM03298P1 | Banchory | 9 | 2298 | -4142.77 |
| CL9_601498846PSV_F0731482CCAM03298P1 | Banchory | 10 | 2298 | -4142.77 |
| CL9_601500341PSV_F0731482CCAM05298P1 | Elrick | 2 | 2298 | -4142.77 |
| CL9_601500405PSV_F0731482CCAM05298P1 | Elrick | 3 | 2298 | -4142.77 |
| CL9_601500654PSV_F0731482CCAM05298P1 | Elrick | 4 | 2298 | -4142.77 |
| CL9_601500722PSV_F0731482CCAM05298P1 | Elrick | 5 | 2298 | -4142.77 |
| CL9_601500786PSV_F0731482CCAM05298P1 | Elrick | 6 | 2298 | -4142.77 |
| CL9_601501035PSV_F0731482CCAM05298P1 | Elrick | 7 | 2298 | -4142.77 |
| CL9_601501103PSV_F0731482CCAM05298P1 | Elrick | 8 | 2298 | -4142.77 |
| CL9_601501167PSV_F0731482CCAM05298P1 | Elrick | 9 | 2298 | -4142.77 |
| CL9_601501353PSV_F0731482CCAM05298P1 | Elrick | 10 | 2298 | -4142.77 |
| CL9_601586992PSV_F0731692CCAM01299P1 | Achanarraas | 1 | 2299 | -4141.05 |
| CL9_601587067PSV_F0731692CCAM01299P1 | Achanarraas | 2 | 2299 | -4141.05 |
| CL9_601587132PSV_F0731692CCAM01299P1 | Achanarraas | 3 | 2299 | -4141.05 |
| CL9_601587197PSV_F0731692CCAM01299P1 | Achanarraas | 4 | 2299 | -4141.05 |
| CL9_601587393PSV_F0731692CCAM01299P1 | Achanarraas | 5 | 2299 | -4141.05 |
| CL9_601587720PSV_F0731692CCAM02299P1 | Melrose | 1 | 2299 | -4141.05 |
| CL9_601587789PSV_F0731692CCAM02299P1 | Melrose | 2 | 2299 | -4141.05 |
| CL9_601587853PSV_F0731692CCAM02299P1 | Melrose | 3 | 2299 | -4141.05 |
| CL9_601587917PSV_F0731692CCAM02299P1 | Melrose | 4 | 2299 | -4141.05 |
| CL9_601588121PSV_F0731692CCAM02299P1 | Melrose | 5 | 2299 | -4141.05 |
| CL9_601673641PSV_F0731944CCAM01300P1 | Stornoway | 1 | 2300 | -4139.85 |
| CL9_601673709PSV_F0731944CCAM01300P1 | Stornoway | 2 | 2300 | -4139.85 |
| CL9_601673772PSV_F0731944CCAM01300P1 | Stornoway | 3 | 2300 | -4139.85 |
| CL9_601673837PSV_F0731944CCAM01300P1 | Stornoway | 4 | 2300 | -4139.85 |

| | | | | |
|---|---|---|---|---|
| CL9_601674071PSV_F0731944CCAM01300P1 | Stornoway | 5 | 2300 | -4139.85 |
| CL9_601674138PSV_F0731944CCAM01300P1 | Stornoway | 6 | 2300 | -4139.85 |
| CL9_601674202PSV_F0731944CCAM01300P1 | Stornoway | 7 | 2300 | -4139.85 |
| CL9_601674266PSV_F0731944CCAM01300P1 | Stornoway | 8 | 2300 | -4139.85 |
| CL9_601674331PSV_F0731944CCAM01300P1 | Stornoway | 9 | 2300 | -4139.85 |
| CL9_601674525PSV_F0731944CCAM01300P1 | Stornoway | 10 | 2300 | -4139.85 |
| CL9_601674839PSV_F0731944CCAM02300P1 | Stoneywood | 1 | 2300 | -4139.85 |
| CL9_601674908PSV_F0731944CCAM02300P1 | Stoneywood | 2 | 2300 | -4139.85 |
| CL9_601674972PSV_F0731944CCAM02300P1 | Stoneywood | 3 | 2300 | -4139.85 |
| CL9_601675035PSV_F0731944CCAM02300P1 | Stoneywood | 4 | 2300 | -4139.85 |
| CL9_601675099PSV_F0731944CCAM02300P1 | Stoneywood | 5 | 2300 | -4139.85 |
| CL9_601675163PSV_F0731944CCAM02300P1 | Stoneywood | 6 | 2300 | -4139.85 |
| CL9_601675226PSV_F0731944CCAM02300P1 | Stoneywood | 7 | 2300 | -4139.85 |
| CL9_601675290PSV_F0731944CCAM02300P1 | Stoneywood | 8 | 2300 | -4139.85 |
| CL9_601675353PSV_F0731944CCAM02300P1 | Stoneywood | 9 | 2300 | -4139.85 |
| CL9_601675547PSV_F0731944CCAM02300P1 | Stoneywood | 10 | 2300 | -4139.85 |
| CL9_601757065PSV_F0732112CCAM01301P1 | LochNess | 1 | 2301 | -4140.06 |
| CL9_601757133PSV_F0732112CCAM01301P1 | LochNess | 2 | 2301 | -4140.06 |
| CL9_601757197PSV_F0732112CCAM01301P1 | LochNess | 3 | 2301 | -4140.06 |
| CL9_601757431PSV_F0732112CCAM01301P1 | LochNess | 4 | 2301 | -4140.06 |
| CL9_601757499PSV_F0732112CCAM01301P1 | LochNess | 5 | 2301 | -4140.06 |
| CL9_601757563PSV_F0732112CCAM01301P1 | LochNess | 6 | 2301 | -4140.06 |
| CL9_601757798PSV_F0732112CCAM01301P1 | LochNess | 7 | 2301 | -4140.06 |
| CL9_601757867PSV_F0732112CCAM01301P1 | LochNess | 8 | 2301 | -4140.06 |
| CL9_601757931PSV_F0732112CCAM01301P1 | LochNess | 9 | 2301 | -4140.06 |
| CL9_601758124PSV_F0732112CCAM01301P1 | LochNess | 10 | 2301 | -4140.06 |
| CL9_601758459PSV_F0732112CCAM02301P1 | LochSkeen | 1 | 2301 | -4140.06 |
| CL9_601758527PSV_F0732112CCAM02301P1 | LochSkeen | 2 | 2301 | -4140.06 |
| CL9_601758825PSV_F0732112CCAM02301P1 | LochSkeen | 4 | 2301 | -4140.06 |
| CL9_601758893PSV_F0732112CCAM02301P1 | LochSkeen | 5 | 2301 | -4140.06 |
| CL9_601758957PSV_F0732112CCAM02301P1 | LochSkeen | 6 | 2301 | -4140.06 |
| CL9_601759191PSV_F0732112CCAM02301P1 | LochSkeen | 7 | 2301 | -4140.06 |
| CL9_601759259PSV_F0732112CCAM02301P1 | LochSkeen | 8 | 2301 | -4140.06 |
| CL9_601759323PSV_F0732112CCAM02301P1 | LochSkeen | 9 | 2301 | -4140.06 |
| CL9_601759517PSV_F0732112CCAM02301P1 | LochSkeen | 10 | 2301 | -4140.06 |
| CL9_602030215PSV_F0732346CCAM01304P1 | Cyrus | 1 | 2304 | -4141.97 |
| CL9_602030283PSV_F0732346CCAM01304P1 | Cyrus | 2 | 2304 | -4141.97 |
| CL9_602030347PSV_F0732346CCAM01304P1 | Cyrus | 3 | 2304 | -4141.97 |
| CL9_602030411PSV_F0732346CCAM01304P1 | Cyrus | 4 | 2304 | -4141.97 |
| CL9_602030475PSV_F0732346CCAM01304P1 | Cyrus | 5 | 2304 | -4141.97 |
| CL9_602030539PSV_F0732346CCAM01304P1 | Cyrus | 6 | 2304 | -4141.97 |
| CL9_602030603PSV_F0732346CCAM01304P1 | Cyrus | 7 | 2304 | -4141.97 |
| CL9_602030667PSV_F0732346CCAM01304P1 | Cyrus | 8 | 2304 | -4141.97 |
| CL9_602030731PSV_F0732346CCAM01304P1 | Cyrus | 9 | 2304 | -4141.97 |
| CL9_602030924PSV_F0732346CCAM01304P1 | Cyrus | 10 | 2304 | -4141.97 |
| CL9_602207577PSV_F0732394CCAM01306P1 | Fladday | 1 | 2306 | -4141.65 |
| CL9_602207708PSV_F0732394CCAM01306P1 | Fladday | 3 | 2306 | -4141.65 |
| CL9_602207771PSV_F0732394CCAM01306P1 | Fladday | 4 | 2306 | -4141.65 |
| CL9_602208005PSV_F0732394CCAM01306P1 | Fladday | 5 | 2306 | -4141.65 |
| CL9_602208073PSV_F0732394CCAM01306P1 | Fladday | 6 | 2306 | -4141.65 |
| CL9_602208136PSV_F0732394CCAM01306P1 | Fladday | 7 | 2306 | -4141.65 |
| CL9_602208199PSV_F0732394CCAM01306P1 | Fladday | 8 | 2306 | -4141.65 |
| CL9_602208457PSV_F0732394CCAM01306P1 | Fladday | 10 | 2306 | -4141.65 |
| CL9_602386426PSV_F0732502CCAM01308P1 | Brent | 1 | 2308 | -4141.14 |
| CL9_602386494PSV_F0732502CCAM01308P1 | Brent | 2 | 2308 | -4141.14 |
| CL9_602386558PSV_F0732502CCAM01308P1 | Brent | 3 | 2308 | -4141.14 |
| CL9_602386622PSV_F0732502CCAM01308P1 | Brent | 4 | 2308 | -4141.14 |
| CL9_602386816PSV_F0732502CCAM01308P1 | Brent | 5 | 2308 | -4141.14 |
| CL9_602387139PSV_F0732502CCAM02308P1 | Carluke | 1 | 2308 | -4141.14 |

| | | | | |
|---|---|---|---|---|
| CL9_602387215PSV_F0732502CCAM02308P1 | Carluke | 2 | 2308 | -4141.14 |
| CL9_602387281PSV_F0732502CCAM02308P1 | Carluke | 3 | 2308 | -4141.14 |
| CL9_602387348PSV_F0732502CCAM02308P1 | Carluke | 4 | 2308 | -4141.14 |
| CL9_602387544PSV_F0732502CCAM02308P1 | Carluke | 5 | 2308 | -4141.14 |
| CL9_602653562PSV_F0732676CCAM01311P1 | Isbister | 3 | 2311 | -4143 |
| CL9_602653628PSV_F0732676CCAM01311P1 | Isbister | 4 | 2311 | -4143 |
| CL9_602653792PSV_F0732676CCAM01311P1 | Isbister | 5 | 2311 | -4143 |
| CL9_602828231PSV_F0732958CCAM02313P1 | Luing | 1 | 2313 | -4145.4 |
| CL9_602828363PSV_F0732958CCAM02313P1 | Luing | 3 | 2313 | -4145.4 |
| CL9_603000359PSV_F0740000CCAM02315P1 | Dauntless | 1 | 2315 | -4147.44 |
| CL9_603000500PSV_F0740000CCAM02315P1 | Dauntless | 3 | 2315 | -4147.44 |
| CL9_603000566PSV_F0740000CCAM02315P1 | Dauntless | 4 | 2315 | -4147.44 |
| CL9_603000762PSV_F0740000CCAM02315P1 | Dauntless | 5 | 2315 | -4147.44 |
| CL9_603092415PSV_F0740000CCAM03315P1 | Thistle | 1 | 2316 | -4147.44 |
| CL9_603092490PSV_F0740000CCAM03315P1 | Thistle | 2 | 2316 | -4147.44 |
| CL9_603092556PSV_F0740000CCAM03315P1 | Thistle | 3 | 2316 | -4147.44 |
| CL9_603092621PSV_F0740000CCAM03315P1 | Thistle | 4 | 2316 | -4147.44 |
| CL9_603092818PSV_F0740000CCAM03315P1 | Thistle | 5 | 2316 | -4147.44 |
| CL9_603093208PSV_F0740000CCAM04315P1 | Otter | 2 | 2316 | -4147.44 |
| CL9_603093273PSV_F0740000CCAM04315P1 | Otter | 3 | 2316 | -4147.44 |
| CL9_603093339PSV_F0740000CCAM04315P1 | Otter | 4 | 2316 | -4147.44 |
| CL9_603093535PSV_F0740000CCAM04315P1 | Otter | 5 | 2316 | -4147.44 |
| CL9_603272071PSV_F0740210CCAM01318P1 | Southerness | 1 | 2318 | -4148.29 |
| CL9_603272139PSV_F0740210CCAM01318P1 | Southerness | 2 | 2318 | -4148.29 |
| CL9_603272202PSV_F0740210CCAM01318P1 | Southerness | 3 | 2318 | -4148.29 |
| CL9_603272266PSV_F0740210CCAM01318P1 | Southerness | 4 | 2318 | -4148.29 |
| CL9_603272329PSV_F0740210CCAM01318P1 | Southerness | 5 | 2318 | -4148.29 |
| CL9_603272392PSV_F0740210CCAM01318P1 | Southerness | 6 | 2318 | -4148.29 |
| CL9_603272455PSV_F0740210CCAM01318P1 | Southerness | 7 | 2318 | -4148.29 |
| CL9_603272518PSV_F0740210CCAM01318P1 | Southerness | 8 | 2318 | -4148.29 |
| CL9_603272581PSV_F0740210CCAM01318P1 | Southerness | 9 | 2318 | -4148.29 |
| CL9_603272775PSV_F0740210CCAM01318P1 | Southerness | 10 | 2318 | -4148.29 |
| CL9_603273087PSV_F0740210CCAM02318P1 | Ladyburn | 1 | 2318 | -4148.29 |
| CL9_603273155PSV_F0740210CCAM02318P1 | Ladyburn | 2 | 2318 | -4148.29 |
| CL9_603273218PSV_F0740210CCAM02318P1 | Ladyburn | 3 | 2318 | -4148.29 |
| CL9_603273281PSV_F0740210CCAM02318P1 | Ladyburn | 4 | 2318 | -4148.29 |
| CL9_603273345PSV_F0740210CCAM02318P1 | Ladyburn | 5 | 2318 | -4148.29 |
| CL9_603273408PSV_F0740210CCAM02318P1 | Ladyburn | 6 | 2318 | -4148.29 |
| CL9_603273471PSV_F0740210CCAM02318P1 | Ladyburn | 7 | 2318 | -4148.29 |
| CL9_603273534PSV_F0740210CCAM02318P1 | Ladyburn | 8 | 2318 | -4148.29 |
| CL9_603273597PSV_F0740210CCAM02318P1 | Ladyburn | 9 | 2318 | -4148.29 |
| CL9_603273791PSV_F0740210CCAM02318P1 | Ladyburn | 10 | 2318 | -4148.29 |
| CL9_603274117PSV_F0740210CCAM03318P1 | Gannet | 1 | 2318 | -4148.29 |
| CL9_603274185PSV_F0740210CCAM03318P1 | Gannet | 2 | 2318 | -4148.29 |
| CL9_603274249PSV_F0740210CCAM03318P1 | Gannet | 3 | 2318 | -4148.29 |
| CL9_603274313PSV_F0740210CCAM03318P1 | Gannet | 4 | 2318 | -4148.29 |
| CL9_603274376PSV_F0740210CCAM03318P1 | Gannet | 5 | 2318 | -4148.29 |
| CL9_603274439PSV_F0740210CCAM03318P1 | Gannet | 6 | 2318 | -4148.29 |
| CL9_603274502PSV_F0740210CCAM03318P1 | Gannet | 7 | 2318 | -4148.29 |
| CL9_603274565PSV_F0740210CCAM03318P1 | Gannet | 8 | 2318 | -4148.29 |
| CL9_603274628PSV_F0740210CCAM03318P1 | Gannet | 9 | 2318 | -4148.29 |
| CL9_603274822PSV_F0740210CCAM03318P1 | Gannet | 10 | 2318 | -4148.29 |
| CL9_603359716PSV_F0740210CCAM04318P1 | Beryl | 1 | 2319 | -4148.29 |
| CL9_603359784PSV_F0740210CCAM04318P1 | Beryl | 2 | 2319 | -4148.29 |
| CL9_603359847PSV_F0740210CCAM04318P1 | Beryl | 3 | 2319 | -4148.29 |
| CL9_603359910PSV_F0740210CCAM04318P1 | Beryl | 4 | 2319 | -4148.29 |
| CL9_603359973PSV_F0740210CCAM04318P1 | Beryl | 5 | 2319 | -4148.29 |
| CL9_603360036PSV_F0740210CCAM04318P1 | Beryl | 6 | 2319 | -4148.29 |
| CL9_603360099PSV_F0740210CCAM04318P1 | Beryl | 7 | 2319 | -4148.29 |

| | | | | |
|---|---|---|---|---|
| CL9_603360163PSV_F0740210CCAM04318P1 | Beryl | 8 | 2319 | -4148.29 |
| CL9_603360227PSV_F0740210CCAM04318P1 | Beryl | 9 | 2319 | -4148.29 |
| CL9_603360420PSV_F0740210CCAM04318P1 | Beryl | 10 | 2319 | -4148.29 |
| CL9_603360748PSV_F0740210CCAM05318P1 | Clyde | 1 | 2319 | -4148.29 |
| CL9_603360816PSV_F0740210CCAM05318P1 | Clyde | 2 | 2319 | -4148.29 |
| CL9_603360880PSV_F0740210CCAM05318P1 | Clyde | 3 | 2319 | -4148.29 |
| CL9_603360944PSV_F0740210CCAM05318P1 | Clyde | 4 | 2319 | -4148.29 |
| CL9_603361138PSV_F0740210CCAM05318P1 | Clyde | 5 | 2319 | -4148.29 |
| CL9_603448754PSV_F0740210CCAM01320P1 | Fyvie | 1 | 2320 | -4148.29 |
| CL9_603448822PSV_F0740210CCAM01320P1 | Fyvie | 2 | 2320 | -4148.29 |
| CL9_603448886PSV_F0740210CCAM01320P1 | Fyvie | 3 | 2320 | -4148.29 |
| CL9_603449472PSV_F0740210CCAM02320P1 | LadderHills | 1 | 2320 | -4148.29 |
| CL9_603449540PSV_F0740210CCAM02320P1 | LadderHills | 2 | 2320 | -4148.29 |
| CL9_603449604PSV_F0740210CCAM02320P1 | LadderHills | 3 | 2320 | -4148.29 |
| CL9_603449668PSV_F0740210CCAM02320P1 | LadderHills | 4 | 2320 | -4148.29 |
| CL9_603449872PSV_F0740210CCAM02320P1 | LadderHills | 5 | 2320 | -4148.29 |
| CL9_604602298PSV_F0740540CCAM01333P1 | Auchterarder | 1 | 2333 | -4150.33 |
| CL9_604602366PSV_F0740540CCAM01333P1 | Auchterarder | 2 | 2333 | -4150.33 |
| CL9_604602430PSV_F0740540CCAM01333P1 | Auchterarder | 3 | 2333 | -4150.33 |
| CL9_604602664PSV_F0740540CCAM01333P1 | Auchterarder | 4 | 2333 | -4150.33 |
| CL9_604602732PSV_F0740540CCAM01333P1 | Auchterarder | 5 | 2333 | -4150.33 |
| CL9_604602796PSV_F0740540CCAM01333P1 | Auchterarder | 6 | 2333 | -4150.33 |
| CL9_604603031PSV_F0740540CCAM01333P1 | Auchterarder | 7 | 2333 | -4150.33 |
| CL9_604603099PSV_F0740540CCAM01333P1 | Auchterarder | 8 | 2333 | -4150.33 |
| CL9_604603163PSV_F0740540CCAM01333P1 | Auchterarder | 9 | 2333 | -4150.33 |
| CL9_604603324PSV_F0740540CCAM01333P1 | Auchterarder | 10 | 2333 | -4150.33 |
| CL9_604603643PSV_F0740540CCAM02333P1 | Alloa | 1 | 2333 | -4150.33 |
| CL9_604603712PSV_F0740540CCAM02333P1 | Alloa | 2 | 2333 | -4150.33 |
| CL9_604603776PSV_F0740540CCAM02333P1 | Alloa | 3 | 2333 | -4150.33 |
| CL9_604603976PSV_F0740540CCAM02333P1 | Alloa | 4 | 2333 | -4150.33 |
| CL9_604604040PSV_F0740540CCAM02333P1 | Alloa | 5 | 2333 | -4150.33 |
| CL9_604604104PSV_F0740540CCAM02333P1 | Alloa | 6 | 2333 | -4150.33 |
| CL9_604604304PSV_F0740540CCAM02333P1 | Alloa | 7 | 2333 | -4150.33 |
| CL9_604604368PSV_F0740540CCAM02333P1 | Alloa | 8 | 2333 | -4150.33 |
| CL9_604604530PSV_F0740540CCAM02333P1 | Alloa | 9 | 2333 | -4150.33 |
| CL9_604604856PSV_F0740540CCAM03333P1 | Auchterarder | 11 | 2333 | -4150.33 |
| CL9_604604924PSV_F0740540CCAM03333P1 | Cathres | 1 | 2333 | -4150.33 |
| CL9_604604988PSV_F0740540CCAM03333P1 | Cathres | 2 | 2333 | -4150.33 |
| CL9_604605243PSV_F0740540CCAM03333P1 | Cathres | 3 | 2333 | -4150.33 |
| CL9_604605317PSV_F0740540CCAM03333P1 | Cathres | 4 | 2333 | -4150.33 |
| CL9_604605381PSV_F0740540CCAM03333P1 | Cathres | 5 | 2333 | -4150.33 |
| CL9_604605636PSV_F0740540CCAM03333P1 | Cathres | 6 | 2333 | -4150.33 |
| CL9_604605704PSV_F0740540CCAM03333P1 | Cathres | 7 | 2333 | -4150.33 |
| CL9_604605768PSV_F0740540CCAM03333P1 | Cathres | 8 | 2333 | -4150.33 |
| CL9_604605962PSV_F0740540CCAM03333P1 | Cathres | 9 | 2333 | -4150.33 |
| CL9_605043016PSV_F0740540CCAM01338P1 | Cathres | 10 | 2333 | -4150.33 |
| CL9_605043084PSV_F0740540CCAM01338P1 | CuttyHill | 1 | 2338 | -4150.33 |
| CL9_605043147PSV_F0740540CCAM01338P1 | CuttyHill | 2 | 2338 | -4150.33 |
| CL9_605043211PSV_F0740540CCAM01338P1 | CuttyHill | 3 | 2338 | -4150.33 |
| CL9_605043274PSV_F0740540CCAM01338P1 | CuttyHill | 4 | 2338 | -4150.33 |
| CL9_605043337PSV_F0740540CCAM01338P1 | CuttyHill | 5 | 2338 | -4150.33 |
| CL9_605043400PSV_F0740540CCAM01338P1 | CuttyHill | 6 | 2338 | -4150.33 |
| CL9_605043463PSV_F0740540CCAM01338P1 | CuttyHill | 7 | 2338 | -4150.33 |
| CL9_605043527PSV_F0740540CCAM01338P1 | CuttyHill | 8 | 2338 | -4150.33 |
| CL9_605043721PSV_F0740540CCAM01338P1 | CuttyHill | 9 | 2338 | -4150.33 |
| CL9_605044058PSV_F0740540CCAM02338P1 | CuttyHill | 10 | 2338 | -4150.33 |
| CL9_605044379PSV_F0740540CCAM02338P1 | Miller | 5 | 2338 | -4150.33 |
| CL9_605044442PSV_F0740540CCAM02338P1 | Miller | 6 | 2338 | -4150.33 |
| CL9_605044505PSV_F0740540CCAM02338P1 | Miller | 7 | 2338 | -4150.33 |

| | | | | |
|---|---|---|---|---|
| CL9_605044771PSV_F0740540CCAM02338P1 | Miller | 9 | 2338 | -4150.33 |
| CL9_605135647PSV_F0740762CCAM01339P1 | Miller | 10 | 2338 | -4150.62 |
| CL9_605135715PSV_F0740762CCAM01339P1 | Arbuthnott | 1 | 2339 | -4150.62 |
| CL9_605135779PSV_F0740762CCAM01339P1 | Arbuthnott | 2 | 2339 | -4150.62 |
| CL9_605135842PSV_F0740762CCAM01339P1 | Arbuthnott | 3 | 2339 | -4150.62 |
| CL9_605135905PSV_F0740762CCAM01339P1 | Arbuthnott | 4 | 2339 | -4150.62 |
| CL9_605135968PSV_F0740762CCAM01339P1 | Arbuthnott | 5 | 2339 | -4150.62 |
| CL9_605136031PSV_F0740762CCAM01339P1 | Arbuthnott | 6 | 2339 | -4150.62 |
| CL9_605136094PSV_F0740762CCAM01339P1 | Arbuthnott | 7 | 2339 | -4150.62 |
| CL9_605136158PSV_F0740762CCAM01339P1 | Arbuthnott | 8 | 2339 | -4150.62 |
| CL9_605136352PSV_F0740762CCAM01339P1 | Arbuthnott | 9 | 2339 | -4150.62 |
| CL9_605136665PSV_F0740762CCAM02339P1 | Arbuthnott | 10 | 2339 | -4150.62 |
| CL9_605136733PSV_F0740762CCAM02339P1 | Clair | 1 | 2339 | -4150.62 |
| CL9_605136797PSV_F0740762CCAM02339P1 | Clair | 2 | 2339 | -4150.62 |
| CL9_605136861PSV_F0740762CCAM02339P1 | Clair | 3 | 2339 | -4150.62 |
| CL9_605136989PSV_F0740762CCAM02339P1 | Clair | 4 | 2339 | -4150.62 |
| CL9_605137053PSV_F0740762CCAM02339P1 | Clair | 5 | 2339 | -4150.62 |
| CL9_605137117PSV_F0740762CCAM02339P1 | Clair | 6 | 2339 | -4150.62 |
| CL9_605137181PSV_F0740762CCAM02339P1 | Clair | 7 | 2339 | -4150.62 |
| CL9_605137375PSV_F0740762CCAM02339P1 | Clair | 8 | 2339 | -4150.62 |
| CL9_605137722PSV_F0740762CCAM03339P1 | Clair | 9 | 2339 | -4150.62 |
| CL9_605137790PSV_F0740762CCAM03339P1 | Clair | 10 | 2339 | -4150.62 |
| CL9_605137854PSV_F0740762CCAM03339P1 | Fife | 1 | 2339 | -4150.62 |
| CL9_605137927PSV_F0740762CCAM03339P1 | Fife | 2 | 2339 | -4150.62 |
| CL9_605138120PSV_F0740762CCAM03339P1 | Fife | 3 | 2339 | -4150.62 |
| CL9_605138185PSV_F0740762CCAM03339P1 | Fife | 4 | 2339 | -4150.62 |
| CL9_605138249PSV_F0740762CCAM03339P1 | Fife | 5 | 2339 | -4150.62 |
| CL9_605138312PSV_F0740762CCAM03339P1 | Fife | 6 | 2339 | -4150.62 |
| CL9_605138375PSV_F0740762CCAM03339P1 | Fife | 7 | 2339 | -4150.62 |
| CL9_605138568PSV_F0740762CCAM03339P1 | Fife | 8 | 2339 | -4150.62 |
| CL9_605849522PSV_F0740762CCAM01347P1 | Fife | 9 | 2339 | -4150.62 |
| CL9_605849590PSV_F0740762CCAM01347P1 | Fife | 10 | 2339 | -4150.62 |
| CL9_605849663PSV_F0740762CCAM01347P1 | Conival | 1 | 2347 | -4150.62 |
| CL9_605849727PSV_F0740762CCAM01347P1 | Conival | 2 | 2347 | -4150.62 |
| CL9_605849921PSV_F0740762CCAM01347P1 | Conival | 3 | 2347 | -4150.62 |
| CL9_605850231PSV_F0740762CCAM02347P1 | Fulmar | 9 | 2347 | -4150.62 |
| CL9_605850300PSV_F0740762CCAM02347P1 | Fulmar | 10 | 2347 | -4150.62 |
| CL9_605850951PSV_F0740762CCAM03347P1 | Conival | 4 | 2347 | -4150.62 |
| CL9_605851019PSV_F0740762CCAM03347P1 | Conival | 5 | 2347 | -4150.62 |
| CL9_605851083PSV_F0740762CCAM03347P1 | Fulmar | 1 | 2347 | -4150.62 |
| CL9_605851146PSV_F0740762CCAM03347P1 | Fulmar | 2 | 2347 | -4150.62 |
| CL9_605851209PSV_F0740762CCAM03347P1 | Fulmar | 3 | 2347 | -4150.62 |
| CL9_605851272PSV_F0740762CCAM03347P1 | Fulmar | 4 | 2347 | -4150.62 |
| CL9_605851335PSV_F0740762CCAM03347P1 | Fulmar | 5 | 2347 | -4150.62 |
| CL9_605851398PSV_F0740762CCAM03347P1 | Fulmar | 6 | 2347 | -4150.62 |
| CL9_605851461PSV_F0740762CCAM03347P1 | Fulmar | 7 | 2347 | -4150.62 |
| CL9_605851655PSV_F0740762CCAM03347P1 | Fulmar | 8 | 2347 | -4150.62 |
| CL9_606022000PSV_F0750000CCAM01349P1 | Gardenstown | 3 | 2349 | -4150.62 |
| CL9_606022068PSV_F0750000CCAM01349P1 | Gardenstown | 4 | 2349 | -4150.62 |
| CL9_606022132PSV_F0750000CCAM01349P1 | Gardenstown | 5 | 2349 | -4150.62 |
| CL9_606022196PSV_F0750000CCAM01349P1 | Gardenstown | 6 | 2349 | -4150.62 |
| CL9_606022260PSV_F0750000CCAM01349P1 | Gardenstown | 7 | 2349 | -4150.62 |
| CL9_606022454PSV_F0750000CCAM01349P1 | Gardenstown | 8 | 2349 | -4150.62 |
| CL9_606295263PSV_F0750060CCAM04352P1 | Gardenstown | 10 | 2349 | -4150.45 |
| CL9_606292140PSV_F0750060CCAM01352P1 | Crail | 9 | 2352 | -4150.45 |
| CL9_606292204PSV_F0750060CCAM01352P1 | Crieff | 1 | 2352 | -4150.45 |
| CL9_606292438PSV_F0750060CCAM01352P1 | Crieff | 2 | 2352 | -4150.45 |
| CL9_606292503PSV_F0750060CCAM01352P1 | Crieff | 3 | 2352 | -4150.45 |
| CL9_606292567PSV_F0750060CCAM01352P1 | Crieff | 4 | 2352 | -4150.45 |

| | | | | |
|---|---|---|---|---|
| CL9_606292802PSV_F0750060CCAM01352P1 | Crieff | 5 | 2352 | -4150.45 |
| CL9_606292867PSV_F0750060CCAM01352P1 | Crieff | 6 | 2352 | -4150.45 |
| CL9_606293061PSV_F0750060CCAM01352P1 | Crieff | 7 | 2352 | -4150.45 |
| CL9_606293390PSV_F0750060CCAM02352P1 | Crieff | 8 | 2352 | -4150.45 |
| CL9_606293458PSV_F0750060CCAM02352P1 | Crieff | 9 | 2352 | -4150.45 |
| CL9_606293522PSV_F0750060CCAM02352P1 | James | 1 | 2352 | -4150.45 |
| CL9_606293630PSV_F0750060CCAM02352P1 | James | 2 | 2352 | -4150.45 |
| CL9_606293694PSV_F0750060CCAM02352P1 | James | 3 | 2352 | -4150.45 |
| CL9_606293758PSV_F0750060CCAM02352P1 | James | 4 | 2352 | -4150.45 |
| CL9_606293858PSV_F0750060CCAM02352P1 | James | 5 | 2352 | -4150.45 |
| CL9_606293922PSV_F0750060CCAM02352P1 | James | 6 | 2352 | -4150.45 |
| CL9_606294116PSV_F0750060CCAM02352P1 | James | 7 | 2352 | -4150.45 |
| CL9_606294460PSV_F0750060CCAM03352P1 | James | 8 | 2352 | -4150.45 |
| CL9_606294528PSV_F0750060CCAM03352P1 | James | 9 | 2352 | -4150.45 |
| CL9_606294592PSV_F0750060CCAM03352P1 | Kilmarnock | 1 | 2352 | -4150.45 |
| CL9_606295327PSV_F0750060CCAM04352P1 | Crail | 1 | 2352 | -4150.45 |
| CL9_606295562PSV_F0750060CCAM04352P1 | Crail | 2 | 2352 | -4150.45 |
| CL9_606295631PSV_F0750060CCAM04352P1 | Crail | 3 | 2352 | -4150.45 |
| CL9_606295929PSV_F0750060CCAM04352P1 | Crail | 5 | 2352 | -4150.45 |
| CL9_606295994PSV_F0750060CCAM04352P1 | Crail | 6 | 2352 | -4150.45 |
| CL9_606296188PSV_F0750060CCAM04352P1 | Crail | 7 | 2352 | -4150.45 |
| CL9_606468653PSV_F0750264CCAM01354P1 | Kilmarnock | 4 | 2352 | -4150.84 |
| CL9_606468721PSV_F0750264CCAM01354P1 | Kilmarnock | 5 | 2352 | -4150.84 |
| CL9_606468784PSV_F0750264CCAM01354P1 | Greenock | 1 | 2354 | -4150.84 |
| CL9_606468847PSV_F0750264CCAM01354P1 | Greenock | 2 | 2354 | -4150.84 |
| CL9_606468910PSV_F0750264CCAM01354P1 | Greenock | 3 | 2354 | -4150.84 |
| CL9_606468973PSV_F0750264CCAM01354P1 | Greenock | 4 | 2354 | -4150.84 |
| CL9_606469036PSV_F0750264CCAM01354P1 | Greenock | 5 | 2354 | -4150.84 |
| CL9_606469099PSV_F0750264CCAM01354P1 | Greenock | 6 | 2354 | -4150.84 |
| CL9_606469162PSV_F0750264CCAM01354P1 | Greenock | 7 | 2354 | -4150.84 |
| CL9_606469356PSV_F0750264CCAM01354P1 | Greenock | 8 | 2354 | -4150.84 |
| CL9_606469670PSV_F0750264CCAM02354P1 | Greenock | 9 | 2354 | -4150.84 |
| CL9_606469735PSV_F0750264CCAM02354P1 | Greenock | 10 | 2354 | -4150.84 |
| CL9_606469798PSV_F0750264CCAM02354P1 | Greenvoe | 1 | 2354 | -4150.84 |
| CL9_606469861PSV_F0750264CCAM02354P1 | Greenvoe | 2 | 2354 | -4150.84 |
| CL9_606469924PSV_F0750264CCAM02354P1 | Greenvoe | 3 | 2354 | -4150.84 |
| CL9_606469987PSV_F0750264CCAM02354P1 | Greenvoe | 4 | 2354 | -4150.84 |
| CL9_606470060PSV_F0750264CCAM02354P1 | Greenvoe | 5 | 2354 | -4150.84 |
| CL9_606470123PSV_F0750264CCAM02354P1 | Greenvoe | 6 | 2354 | -4150.84 |
| CL9_606470186PSV_F0750264CCAM02354P1 | Greenvoe | 7 | 2354 | -4150.84 |
| CL9_606470380PSV_F0750264CCAM02354P1 | Greenvoe | 8 | 2354 | -4150.84 |
| CL9_606645020PSV_F0750456CCAM02356P1 | Greenvoe | 9 | 2354 | -4152.26 |
| CL9_606645088PSV_F0750456CCAM02356P1 | Greenvoe | 10 | 2354 | -4152.26 |
| CL9_606644000PSV_F0750456CCAM01356P1 | Flodigarry | 5 | 2356 | -4152.26 |
| CL9_606644068PSV_F0750456CCAM01356P1 | Snorre | 1 | 2356 | -4152.26 |
| CL9_606644131PSV_F0750456CCAM01356P1 | Snorre | 2 | 2356 | -4152.26 |
| CL9_606644194PSV_F0750456CCAM01356P1 | Snorre | 3 | 2356 | -4152.26 |
| CL9_606644257PSV_F0750456CCAM01356P1 | Snorre | 4 | 2356 | -4152.26 |
| CL9_606644321PSV_F0750456CCAM01356P1 | Snorre | 5 | 2356 | -4152.26 |
| CL9_606644384PSV_F0750456CCAM01356P1 | Snorre | 6 | 2356 | -4152.26 |
| CL9_606644448PSV_F0750456CCAM01356P1 | Snorre | 7 | 2356 | -4152.26 |
| CL9_606644511PSV_F0750456CCAM01356P1 | Snorre | 8 | 2356 | -4152.26 |
| CL9_606644705PSV_F0750456CCAM01356P1 | Snorre | 9 | 2356 | -4152.26 |
| CL9_606646119PSV_F0750456CCAM03356P1 | Flodigarry | 1 | 2356 | -4152.26 |
| CL9_606646183PSV_F0750456CCAM03356P1 | Flodigarry | 2 | 2356 | -4152.26 |
| CL9_606646247PSV_F0750456CCAM03356P1 | Flodigarry | 3 | 2356 | -4152.26 |
| CL9_606646441PSV_F0750456CCAM03356P1 | Flodigarry | 4 | 2356 | -4152.26 |
